# Spermine Regulates Immune and Signal Transduction Dysfunction in Diabetic Cardiomyopathy

**DOI:** 10.3389/fendo.2021.740493

**Published:** 2022-01-31

**Authors:** Can Wei, Mengting Sun, Xiao Liang, Bingbing Che, Ningning Wang, Lili Shi, Ying Fan

**Affiliations:** ^1^Department of Pathophysiology, Harbin Medical University, Harbin, China; ^2^Department of Surgery, The Second Affiliated Hospital of Harbin Medical University, Harbin, China; ^3^Department of Cardiovascular, The First Affiliated Hospital of Harbin Medical University, Harbin, China; ^4^Department of Cadre Ward, The First Affiliated Hospital of Harbin Medical University, Harbin, China

**Keywords:** diabetic cardiomyopathy, spermine, RNA sequencing, immune system, signal transduction

## Abstract

**Background:**

Diabetic cardiomyopathy (DCM) is a specific form of cardiomyopathy that is independent of coronary artery disease and hypertension. Exploring the transcriptomics of DCM is of great significance for understanding the biology of the disease and for guiding new therapeutic targets for the potential therapeutic effect of spermine (SPM).

**Methods and Results:**

By using a mouse DCM model, we analyzed the transcriptome of the myocardium, before/after treatment with SPM. Using RNA sequencing (RNA-seq), we identified 1,318 differentially expressed genes (DEGs), with 636 being upregulated and 682 being downregulated in DCM compared to control check (CK). We then identified 1,393 DEGs, with 887 being upregulated and 506 being downregulated in SPM compared to DCM. Kyoto Encyclopedia of Genes And Genomes (KEGG) analysis demonstrated that the DEGs were significantly enriched in the immune system and signal transduction-related pathways. UpSet Venn analysis showed that 174 DEGs in DCM could be reversed by SPM, with 45 candidates related to immune system and related signal transduction pathways. Trend analysis demonstrated the dynamic changes in gene levels in DCM and SPM treatment, shown as 49 immune and signal transduction-related candidates were significantly enriched in some classical pathways, such as complement and coagulation cascades and phosphatidylinositol-4,5-bisphosphate 3-kinase (PI3K)-protein kinase B (Akt) signaling pathway. To further reveal the protective mechanism of SPM to DCM, we predicted 14 overlapped transcription factors (TFs) and their co-factors involved in gene transcription regulation and showed gene interaction with Cytoscape.

**Conclusion:**

The biomarkers and canonical pathways identified in this study may hold the key to understanding the mechanisms of DCM pathobiology and providing new targets for the therapeutic effect of SPM against DCM by targeting abnormal immune response and signal transduction.

## Introduction

Diabetic cardiomyopathy (DCM) is closely associated with type 1 diabetes (T1D), which is characterized by hyperglycemia and results in low-grade systemic inflammation (1). Studies have shown that immune and its related signal transduction pathways, oxidative stress, fibrosis, coronary endothelial cells dysregulation, exosomes, abnormal mitochondrial calcium handling, and some other factors play a potentially important role in the pathogenesis of diabetes and dilated cardiomyopathy, but the exact mechanism is not yet fully understood (1–4). The development of high-throughput genomics techniques improves the classical studies over large gene datasets; thus, we can better understand the molecular mechanism of DCM (5).

Spermine (SPM), a natural product of cellular metabolism, is a small polyamine with positively charged alkylamines (6). SPM has many biological functions and is crucial for diverse physiological processes including immunity and transcriptional regulation (7, 8). We have previously found that the intracellular content of SPM was decreased significantly in cardiomyocytes of T1D rats, resulting in myocardial energy metabolism disorder, oxidative stress, and myocardial fibrosis, which can be alleviated by exogenous SPM treatment (9–12). However, the effect of SPM on DCM, especially on immunity and its related signal transduction pathways, needs to be further explored.

Here, we describe the dynamic changes occurring throughout non-treated and SPM-treated myocardium of diabetic mice from the omics perspectives. The information from this study may be utilized in identifying novel candidate targets and pathways for SPM treatment of DCM.

## Materials and Methods

### Animal Use and Experimental Design

Male C57BL/6J mice (aged 6–8 weeks and weighted 22–24 g) were supplied by the Laboratory Animal Center of Harbin Medical University (Harbin, China). Mice were housed in a controlled environment (24 ± 1°C; 60 ± 10% relative humidity; fixed 12/12 h light/dark cycle) with food and water *ad libitum* and were randomized into three groups (n = 24 per group): (1) control check group (CK), 0.1 mol/L sterile citrate buffer (pH 4.5) was injected intraperitoneally; (2) diabetic cardiomyopathy model group (DCM), a single intraperitoneal injection of streptozotocin (STZ, 180 mg/kg, dissolved in 0.1 mol/L, pH 4.5 citric acid–citrate sodium buffer) was used to establish type 1 diabetes (T1D) model with normal diet fed for 12 weeks; and (3) spermine group (SPM), an injection of SPM (1 mg/kg/day) every day for 2 weeks prior to STZ injection; after which SPM was injected every other day for 12 weeks. The experimental mice were fasted 12 h prior to the STZ administration; immediately thereafter, 10% sucrose was supplemented in the drinking water for 24 h to avoid sudden hypoglycemia due to insulin hyper-secretion. The body weight and blood glucose levels were monitored once weekly after 6 h fasting. Additional details may be found in our previous study (9). Animal safety protocols were followed by the investigators and the Animal Laboratories personnel according to the guidelines of the Harbin Medical University.

After 12 weeks, the mice were fasted overnight and then anesthetized with pentobarbital sodium for tissue collection. The animal study was reviewed and approved by Harbin Medical University. The transthoracic two-dimensional (2D) echocardiography (Vevo 2100) ultrasound system was used to detect heart function. Left ventricular ejection fraction (LVEF), left ventricular fractional shortening (LVFS), left ventricular internal diameter systolic (LVIDs), and left ventricular internal diameter diastolic (LVIDd) were monitored. Images were obtained from two-dimensional, M-mode, pulsed-wave Doppler and tissue Doppler imaging. All measurements were calculated from the average of five consecutive cardiac cycles. The hearts of the mice were carefully dissected from the surrounding tissues and stored at −80°C for further experiments.

### Histological Analysis

The collected heart tissues were subjected to fixation in 4% paraformaldehyde (PFA) and made into paraffin-embedded 5-μm slices. To observe tissue damage and fibrosis, the samples were stained by hematoxylin and eosin (H&E) and collagen I immunohistochemistry, and photographed under a microscope (Leica, Germany).

### RNA Sequencing Analysis

Total RNA was isolated from the collected heart tissues from three mice in each group with Trizol Reagent (Invitrogen, Life Technologies Corporation). RNA quality was assessed on an Agilent 2100 Bioanalyzer (Agilent Technologies, CA, USA) and checked using RNase free agarose gel electrophoresis. Extracted mRNA was enriched by Oligo (dT) beads, with removal of rRNA using a Ribo-Zero Magnetic Kit (Epicentre, WI, USA). Then, the enriched mRNA was fragmented into short fragments and reverse transcribed into cDNA with random primers. After synthesis of second-strand cDNA, fragments were then purified, end repaired, polyadenylated, and ligated to Illumina sequencing adapters. Ligation products were size selected by agarose gel electrophoresis, PCR amplified, and sequenced using Illumina HiSeq 2500 by Gene Denovo Biotechnology Co. (Guangzhou, China) (13). Clean reads were further filtered by fastp (version 0.18.0) (14). We used DESeq2 software to identify RNA differential expression between two different groups (15). The genes/transcripts with the parameter of false discovery rate (FDR) <0.05 and absolute fold change ≥1.5 were considered differentially expressed genes/transcripts.

KEGG enrichment analysis was then used to identify the significantly enriched metabolic or signal transduction pathways in differentially expressed genes (DEGs) comparing with the whole genome background (16). The calculated p-value went through FDR correction, taking FDR <0.05 as a threshold. Briefly, we input gene expression matrix and ranked genes by SinaltoNoise normalization method. Enrichment scores and p-value were calculated in default parameters.

### Statistical Analysis

The calculated p-value went through false discovery rate (FDR)  correction, taking FDR <0.05 as a threshold, and absolute fold change ≥1.5 was considered differentially expressed genes/transcripts. For the myocardial tissue used for RNA-seq analysis, 18 mouse hearts were randomly divided into three parts, that is, six hearts each for the mixed samples. For all experiments, analyses were done at least in biological triplicate.

## Results

### Gene Expression Profile and Enrichment Analysis of Myocardial Tissue in CK, DCM, and SPM Mice

**Figure 1:**
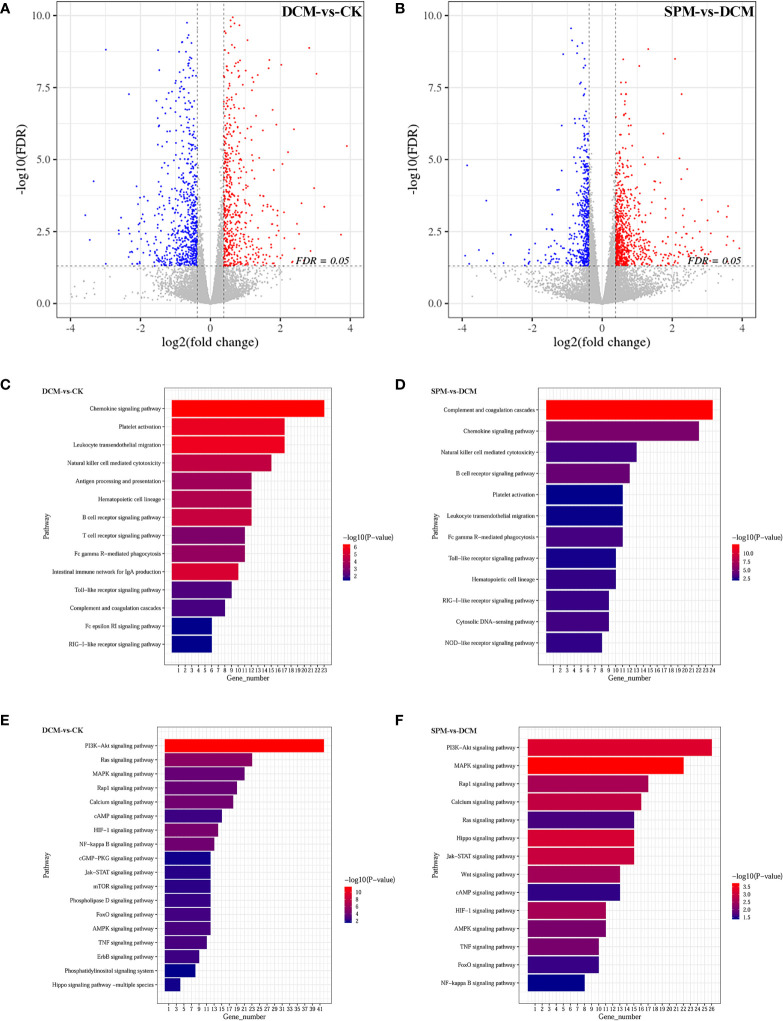
The gene expression profiling and enrichment for myocardium of CK, DCM, and SPM mice. Volcano plot indicates upregulated (red) and downregulated (blue) differentially expressed genes (DEGs) in **(A)** DCM compared to CK group and **(B)** SPM compared to DCM group. Each point represents a gene. KEGG column charts shows DEGs enriched in immunity-related pathway of **(C)** DCM compared to CK group and **(D)** SPM compared to DCM group. KEGG column charts shows the DEGs enriched in signal transduction pathway of **(E)** DCM compared to CK group and **(F)** SPM compared to DCM group. Numbers and p-values of DEGs in each pathway are shown.

The systemic and cardiac data and diastolic dysfunction were validated in the model (**Supplementary Figure S1**). Differentially expressed genes (DEGs) in the myocardium evaluated by RNA-seq analysis showed that 636 genes were upregulated, while 682 genes were downregulated by at least 1.5-fold in the diabetic cardiomyopathy (DCM) model group compared to control check (CK) group, while 887 genes were upregulated while 506 genes were downregulated in the spermine (SPM) group compared to DCM. We used a volcano plot to indicate the above changes. Each point stands for a gene, with color red representing upregulated genes and blue representing downregulated genes (**Figures 1A, B**, **Supplementary Tables S1**, **S2**).

Next, we performed Kyoto Encyclopedia of Genes and Genomes (KEGG) enrichment analysis on DEGs of the three groups and noticed that many genes were enriched in the immune system and signal transduction pathways. When comparing DCM to CK group, DEGs were enriched in some immune-related pathways, such as chemokine signaling pathway, platelet activation, B-cell signaling pathway, T-cell receptor signaling pathway, and Toll-like receptor signaling pathway (**Figure 1C**). They were also enriched in signal transduction pathways, such as phosphatidylinositol-4,5-bisphosphate 3-kinase (PI3K)-protein kinase B (Akt) signaling pathway, Ras signaling pathway, hypoxia-inducible factor-1 (HIF-1) signaling pathway, nuclear factor (NF)-kappa B signaling pathway, mitogen-activated protein kinases (MAPKs) signaling pathway, and AMP-activated protein kinase (AMPK) signaling pathway (**Figure 1E**). When comparing SPM to DCM group, DEGs are significantly enriched in immune-system-related signaling pathways, for example, complement and coagulation cascades, chemokine signaling pathway, natural killer cell-mediated cytotoxicity, B-cell receptor signaling pathway, Toll-like receptor signaling pathway, and RIG-I-like receptor signaling pathway (**Figure 1D**). They may also be enriched in signal transduction pathways, for example, MAPK signaling pathway, PI3K-Akt signaling pathway, Jak-STAT signaling pathway, HIF-1 signaling pathway, Wnt signaling pathway, and AMPK signaling pathway (**Figure 1F**). The annotation of KEGG pathways suggests that the occurrence of DCM and the therapeutic effect of SPM may be closely related to the immune system and signal transduction pathways.

**Figure 2:**
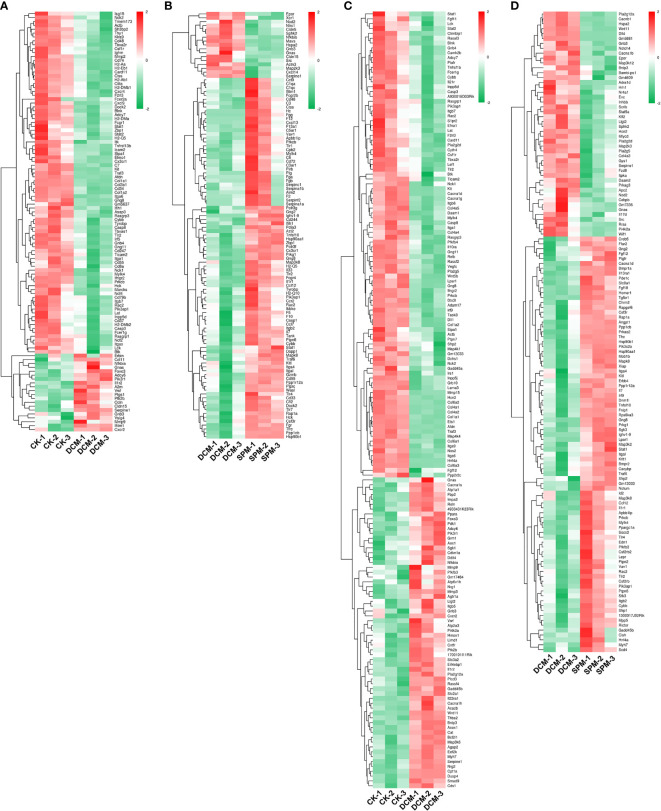
Differential gene expressions in immune and signal-transduction-related pathways. Heat map shows the hierarchical clustering of DEGs in immunity-related pathways of **(A)** DCM compared to CK group and **(B)** SPM compared to DCM group. Heat map shows the hierarchical clustering of DEGs in signal transduction pathways of **(C)** DCM compared to CK group and **(D)** SPM compared to DCM group. In clustering analysis, up- and downregulated genes are colored in red and green, respectively.

In order to intuitively capture the DEGs and KEGG analysis among the three groups, we used heatmap to depict the hierarchical clustering of DEGs that enriched in the immune system and signal transduction pathway in DCM mice before/after SPM treatment. Compared with CK group, 108 DEGs were enriched in immune-system-related pathways (**Figure 2A** and **Supplementary Table S3**), and 160 DEGs were involved in signal transduction in DCM group (**Figure 2C** and **Supplementary Table S5**). Compared with DCM group, 110 DEGs were enriched in immune-system-related pathways (**Figure 2B** and **Supplementary Table S4**), and 131 DEGs were involved in signal transduction in SPM group (**Figure 2D** and **Supplementary Table S6**). The up- and downregulated genes are colored in red and green, respectively.

### SPM Reduces DCM Myocardial Damage by Regulating Immunity and Signal Transduction-Related Pathways

**Figure 3:**
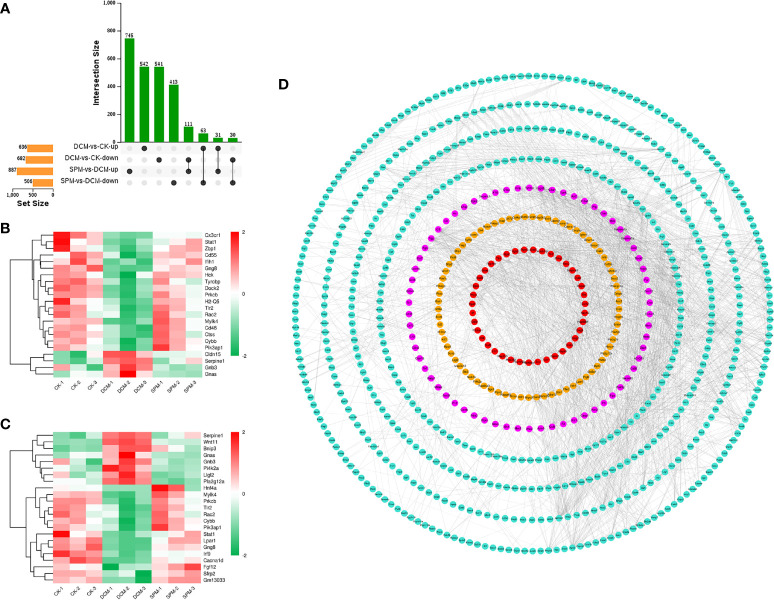
SPM reduces DCM myocardial damage by regulating immunity and signal-transduction-related pathways. **(A)** UpSet Venn diagram shows the number of overlap genes that up- or down-regulated in DCM and reversed by SPM. Heat map shows the hierarchical clustering of overlap genes in **(B)** immunity-related pathways or **(C)** signal transduction pathway. Red color shows the genes with significantly elevated transcription levels, and green color shows the genes with significantly reduced transcription levels. **(D)** Gene interaction network analysis of immunity and signal transduction pathways related candidates between SPM and DCM. Immunity and signal transduction pathways related candidates are colored in pink, yellow, or red, and other genes are colored in turquoise.

To explore the pathogenic process of DCM and the molecular differences of SPM treatment, we cross-matched these differentially expressed candidates from DCM compared to CK group and SPM compared to DCM group. Among these candidates, 63 RNAs were upregulated, and 111 were downregulated in DCM group but reversed by SPM treatment (**Figure 3A**). That is, SPM may play a role in DCM by regulating the expression of these 174 candidates. Subsequently, we screened overlapped genes belonging to immune or signal transduction pathways and plotted a heat map, respectively, with color red standing for elevated transcription levels and green representing downregulated transcription levels (**Figures 3B, C** and **Supplementary Table S7**).

Next, we performed a gene interaction network analysis on the related candidates in immune or signal transduction pathways between SPM and DCM group. The results confirmed that the candidates involved in the two pathways were at the core, reflecting that SPM could protect DCM by regulating immune or signal transduction process, but future in-depth studies are required (**Figure 3D**).

**Figure 4:**
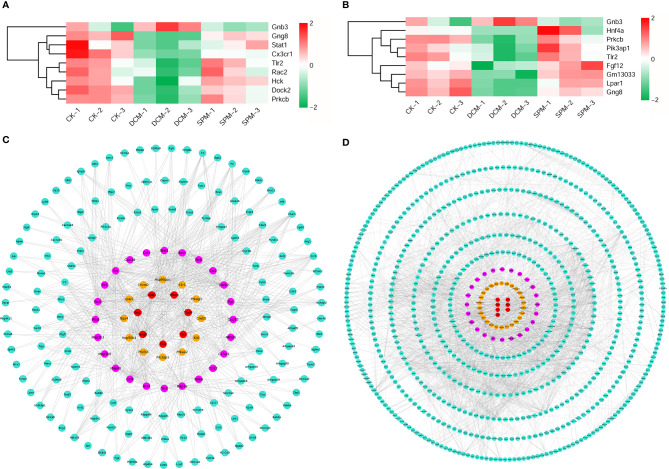
Analysis of the candidates of classical immunity and signal transduction pathways involved in DCM. **(A)** Heat map shows the hierarchical clustering of DEGs in chemokine signaling pathway, B-cell receptor signaling pathway, and Toll-like receptor signaling pathway. **(B)** Gene interaction network analysis of the above candidates between SPM and DCM. The candidates in chemokine signaling pathway, B-cell receptor signaling pathway, and Toll-like receptor signaling pathway are colored in pink, yellow, and red, respectively, and other genes are colored in turquoise. **(C)** Heat map shows the hierarchical clustering of DEGs in the PI3K-Akt signaling pathway, NF-kappa B signaling pathway, and AMPK signaling pathway. **(D)** Gene interaction network analysis of the above candidates between SPM and DCM. The candidates in PI3K-Akt signaling pathway, NF-kappa B signaling pathway, and AMPK signaling pathway are colored in yellow, pink, and red, respectively, and other genes are colored in turquoise.

In addition, we analyzed the related candidate genes of several classic immune system and signal transduction pathways during DCM occurrence. Chemokine signaling pathway, B-cell receptor signaling pathway, and Toll-like receptor signaling pathway are classical pathways closely related to immune response; PI3K-Akt signaling pathway, NF-kappa B signaling pathway, and AMPK signaling pathway are involved in signal transduction of many immune and inflammatory diseases. Heat maps of overlapping genes were performed to show the candidates involved in the classical immune response pathways (**Figure 4A**) and signal transduction pathways (**Figure 4C**).

At the same time, we respectively performed the gene interaction network analysis on the two types of related candidates between the SPM group and the DCM group. The candidates in the chemokine signaling pathway, B-cell receptor signaling pathway, and Toll-like receptor signaling pathway are colored in pink, yellow, and red, respectively, and other genes are colored in turquoise (**Figure 4B**), while candidates in the PI3K-Akt signaling pathway, NF-kappa B signaling pathway, and AMPK signaling pathway are colored in yellow, pink, and red, respectively, and other genes are colored in turquoise (**Figure 4D**). These results confirm that the candidates involved in the above six classic pathways could be the core factors of SPM in protecting diabetic cardiomyopathy.

### Dynamic Differential Expression of Genes in DCM Mice Heart Before/After SPM Treatment

**Figure 5:**
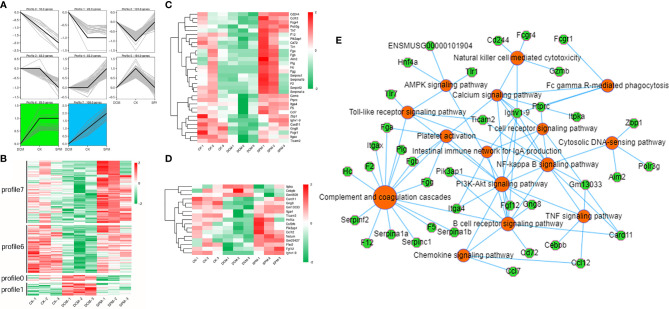
Dynamic differential expression of genes in DCM mice heart before/after SPM treatment. **(A)** Trend analysis of DCM with or without SPM treatment. **(B)** Heat map shows the hierarchical clustering of genes in the profiles 0, 1, 6, and 7. The up- and downregulated genes are colored in red and green, respectively. Heat map shows the hierarchical clustering of the above genes in **(C)** immunity-related pathways or **(D)** signal transduction pathway. **(E)** KEGG network diagram shows the genes in the clusters 0, 1, 6, and 7 that are enriched in immunity and signal transduction pathways. Color orange and green represent pathways and genes, respectively.

We obtained a more comprehensive picture of the dynamic changes in myocardial transcription level of DCM occurrence with or without SPM treatment through trend analysis. All of the genes we analyzed were clustered into seven expression patterns, with clusters 0, 1, 6, and 7 being our concerns (**Figure 5A**). Clusters 0, 1, 6, and 7 contained 10, 22, 83, and 108 genes, respectively, and we revealed the abovementioned genes using heat map (**Figure 5B**). In these four expression patterns, we screened the genes related to the immune system (**Figure 5C** and **Supplementary Table S8**) or signal transduction (**Figure 5D** and **Supplementary Table S8**) and cluster them in the heat map. Red indicates an increased transcription level, while green indicates a decreased transcription level. Furthermore, we enriched the genes of these four expression patterns by KEGG analysis. KEGG network diagram shows the genes in the clusters 0, 1, 6, and 7 that are enriched in immunity and signal transduction pathways, such as complement and coagulation cascades, chemokine signaling pathway, Toll-like receptor signaling pathway, PI3K-Akt signaling pathway, and AMPK signaling pathway. Color orange indicated the related pathways, and green represented these genes (**Figure 5E**).

### Prediction of Transcription Factors and RNA Binding Proteins Target RNAs

**Figure 6:**
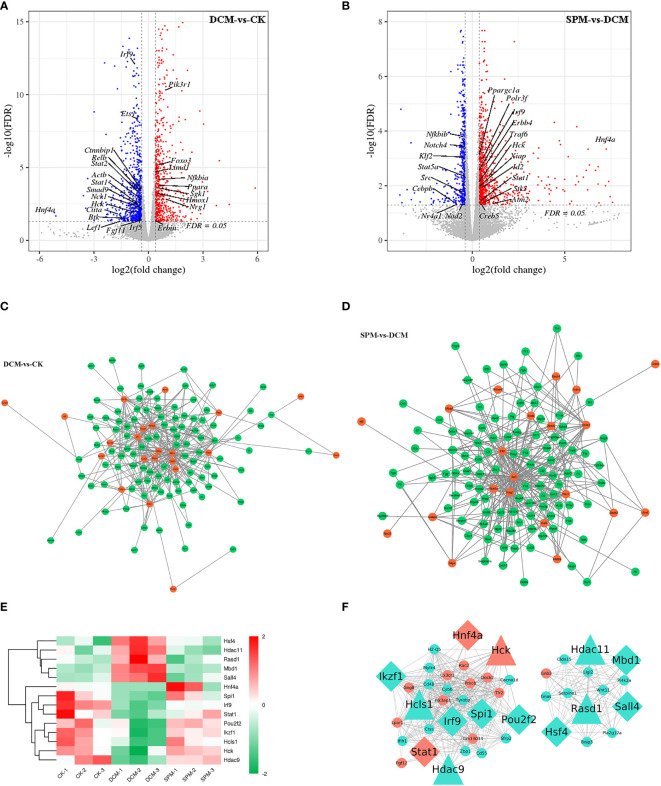
Prediction of transcription factors and its co-factors target RNAs. Tagging the differentially expressed transcription factors (TFs) and its co-factors of **(A, B)** DCM compared to CK group and SPM compared to DCM group on volcano plot. Network diagram suggests the TFs and its co-factors with their predicted target genes of **(C)** DCM compared to CK group and **(D)** SPM compared to DCM group. **(E)** Heat map shows the hierarchical clustering of the TFs and its co-factors that were up- or downregulated in DCM and reversed by SPM. Color red and green represent up- and downregulated genes, respectively. **(F)** Cytoscape diagram shows the correlation between the TFs and its co-factors and the DEGs in immunity and signal transduction pathways. Diamond represents TFs, triangle represents TFs co-factors, and circle represents other genes in the immunity and signal transduction pathways. Color orange represents genes in our concerned pathways, such as PI3K-Akt signaling pathway, and chemokine signaling pathway. Color turquoise represents other genes.

In order to clarify the interaction between gene transcription, we predicted the transcription factors (TFs) and TFs co-factors that target RNAs. Among 1,318 DEGs between DCM and control group, 136 TFs or their co-factors were screened. We also found 171 TFs or their co-factors from the 1,393 DEGs between SPM and CK group. Some TFs or their co-factors were labeled on the volcanic map for DCM compared to CK and for SPM compared to DCM (**Figures 6A, B**).

Next, we selected some TFs and their co-factors in DCM group (compared with CK) and SPM group (compared with DCM), respectively, predicted their target genes, and drew the gene network diagram, indicating that TFs and their co-factors may participate in the pathogenesis of DCM through interaction with the other genes (**Figures 6C, D**).

Venn analysis was used to find the 14 overlapping TFs or TFs co-factors, which up- or downregulated in DCM but reversed by SPM treatment. Heat map showed the hierarchical clustering of these genes with upregulated TFs or their co-factors shown in red and downregulated TFs or their co-factors represented in green (**Figure 6E** and **Table S9**). Then, we selected the above 14 overlapping TFs and TFs co-factors and immune and signal-transduction-related genes and take the interaction gene with the correlation >0.6 to display using Cytoscape (**Figure 6F**). The above results may suggest that the protective effect of SPM on DCM may be mediated by TFs or TF co-factors through harnessing regulatory RNAs to control transcription.

## Discussion

The prevalence of diabetes mellitus in the world has increased in the past 20 years and is currently estimated at 9% in the adult population (17). Cardiovascular complications, including DCM, account for more than 80% of diabetic deaths (18). DCM is associated with changes in systolic and diastolic function without vascular diseases (3, 19). Diabetes affects the heart through a variety of mechanisms such as inflammation and immune-system disorders; thus, researchers begin to focus on the close crosstalk between the immune system and the cardiovascular system (2, 18, 20, 21). However, the pathogenesis of DCM is not clear, especially the mechanisms of complex communication among cardiomyocytes, fibroblasts, and immune cells mediated by immune-related molecules in diabetes. It is still an urgent public health need to clarify the molecular mechanism of DCM and explore novel therapeutic targets to slow or abate diabetes (18).

In this study, we established a T1D mouse model by intraperitoneal injection of STZ. The experimental mice showed hyperglycemia, hyperinsulinemia, weight loss, and abnormal cardiac function and structure (data not shown). Spermine (SPM) is one of the important products of polyamine metabolism in mammals, which has been proven to have many myocardial protective effects, for example, anti-fibrosis, inhibition of oxidative stress and endoplasmic reticulum stress, promotion of autophagy, and recovery of energy metabolism (9–11, 22–24). However, the effects of immune response and related signal transduction process on the DCM model of C57BL/6J mice, and the regulatory role of SPM, a potential modulator for myocardial injury in DCM, are not clear.

We comprehensively analyzed the characteristics and differential gene expressions of DCM myocardial tissue before/after SPM treatment using RNA sequencing (25). Thousands of significantly different genes were identified in DCM group when comparing to CK group and SPM when comparing to DCM group, respectively. KEGG analysis showed that many of these DEGs could be enriched in immune system and related signal transduction pathways. These findings preliminarily indicated that immune response and related signal transduction process are involved in the pathogenesis of DCM and could be regulated by SPM.

Numerous experimental studies have suggested that both innate and adaptive immune responses are activated in the heart in response to inflammation-related tissue injury that results from the pathogenesis of DCM, which in turn cause deleterious cardiac remodeling and left ventricular dysfunction (20, 26, 27). Meanwhile, multiple signal transduction pathways, including the components of these pathways, are essential to regulate the overall immune response and identify potential drug targets (28). One hundred seventy-four genes were found in the DCM but reversed by SPM, suggesting that SPM may play a role in DCM by regulating these 174 DEGs expression. Subsequently, we further screened the genes related to immune response or signal transduction, respectively, among the overlapping genes and found that the above-mentioned molecules can establish extensive regulatory links with other genes.

Among the KEGG-enriched pathways, we focused on three immune system pathways, like chemokine signaling pathway, B-cell receptor signaling pathway, and Toll-like receptor signaling pathway, and three classical signal transduction pathways, like PI3K-Akt signaling pathway, NF-kappa B signaling pathway, and AMPK signaling pathway, and then clustered the related factors in these pathways. Based on the interaction network, we found that these 18 candidates were in the center of the interaction network. Collectively, this discovery strongly suggests that DCM progress may be related to the immune system and related signal transduction dysfunction, and SPM protects against DCM by adjusting the expression of these genes related to these pathways. It has been reported that spermine relieves diabetic cardiomyopathy by inhibiting ROS/p53-mediated repression of calcium-sensitive receptor (9). Spermine suppresses myocardial fibrosis by attenuating canonical Wnt signaling pathway and endoplasmic reticulum stress in diabetic cardiomyopathy (11). However, the association of spermine with TLR signaling has not been reported previously.

TFs are the point of convergence of multiple signaling pathways within eukaryotic cells (27, 29). By modulating the expression or degradation of TFs and their co-factors, protein/protein interactions could be blocked, which can achieve the purpose of disease treatment, ranging from diabetes, inflammatory disorders, and cardiovascular disease to many cancers (30, 31). We identified 14 TFs or their co-factors from the DEGs overlapped among CK, DCM, and SPM groups. Among them, signal transducer and activator of transcription 1 (Stat1, a TF), hepatic nuclear factor 4 alpha (Hnf4a, a TF), and hemopoietic cell kinase (Hck, a TF co-factor) may regulate the RNAs transcription and protein–protein interaction in the above-mentioned six classical immune and signal transduction pathways. In other words, Stat1, Hnf4a, and Hck are expected to be an effective target for SPM to prevent and treat DCM that needs to be verified by animal experiments.

The development of DCM may have a very complicated regulatory network, and this study provides only a preliminary exploration. However, our comprehensive study has revealed the differences between DCM and SPM treatment at the transcriptome level, which lays a foundation for SPM to improve DCM through gene regulation. Meanwhile, there are some limitations in this study. For example, the half-life of spermine should be confirmed, and whether the need to change the dosing regimen is unclear, which needs to be explored in future investigations.

## Conclusion

DCM has remained the leading cause of mortality and morbidity in individuals with diabetes. Animal studies have preliminarily captured the molecular mechanism of DCM and the potential therapeutic target of SPM by RNA-seq. That is, SPM has a protective effect on DCM through the regulation of abnormal gene transcription of immune system and related signal transduction.

## Data Availability Statement

Sequencing data reported here are available at GEO, under the superseries, GSE161052 (https://www.ncbi.nlm.nih.gov/geo/query/acc.cgi?acc=GSE161052).

## Ethics Statement

The animal study was reviewed and approved by Harbin Medical University.

## Author Contributions

CW, LS, and YF designed the research and drafted the manuscript. MS and XL processed the data curation. BC and NW completed the experiment. All authors contributed to the article and approved the submitted version.

## Funding

This research was supported by the National Natural Science Foundation of China (Nos. 82170268, 81800260, and 81900760), University Nursing Program for Young Scholars with Creative Talents in Heilongjiang Province (UNPYSCT-2020165), the Natural Science Foundation of Heilongjiang Province (No. YQ2021H004), and Heilongjiang Postdoctoral Research Starting Fund (No. LBH-Q21024).

## Conflict of Interest

The authors declare that the research was conducted in the absence of any commercial or financial relationships that could be construed as a potential conflict of interest.

## Publisher’s Note

All claims expressed in this article are solely those of the authors and do not necessarily represent those of their affiliated organizations, or those of the publisher, the editors and the reviewers. Any product that may be evaluated in this article, or claim that may be made by its manufacturer, is not guaranteed or endorsed by the publisher.

## Supplementary Material

Supplementary Figure 1 The validation of the cardiac function in the model. **(A–E)** The cardiac function was validated in the CK, DCM, and SPM group. **(A)** The cardiac injury and fibrosis were analyzed using HE staining and Collagen I immunohistochemistry. The EF **(B)**, FS **(C)**, LVIDd **(D)**, and LVIDs **(E)** were analyzed.
