# Supplementary figures and images for: Spermine Regulates Immune and Signal Transduction Dysfunction in Diabetic Cardiomyopathy
Source: Front Endocrinol (Lausanne). 2022 Jan 31;12:740493. doi: 10.3389/fendo.2021.740493 (PMC8842652; doi:10.3389/fendo.2021.740493)

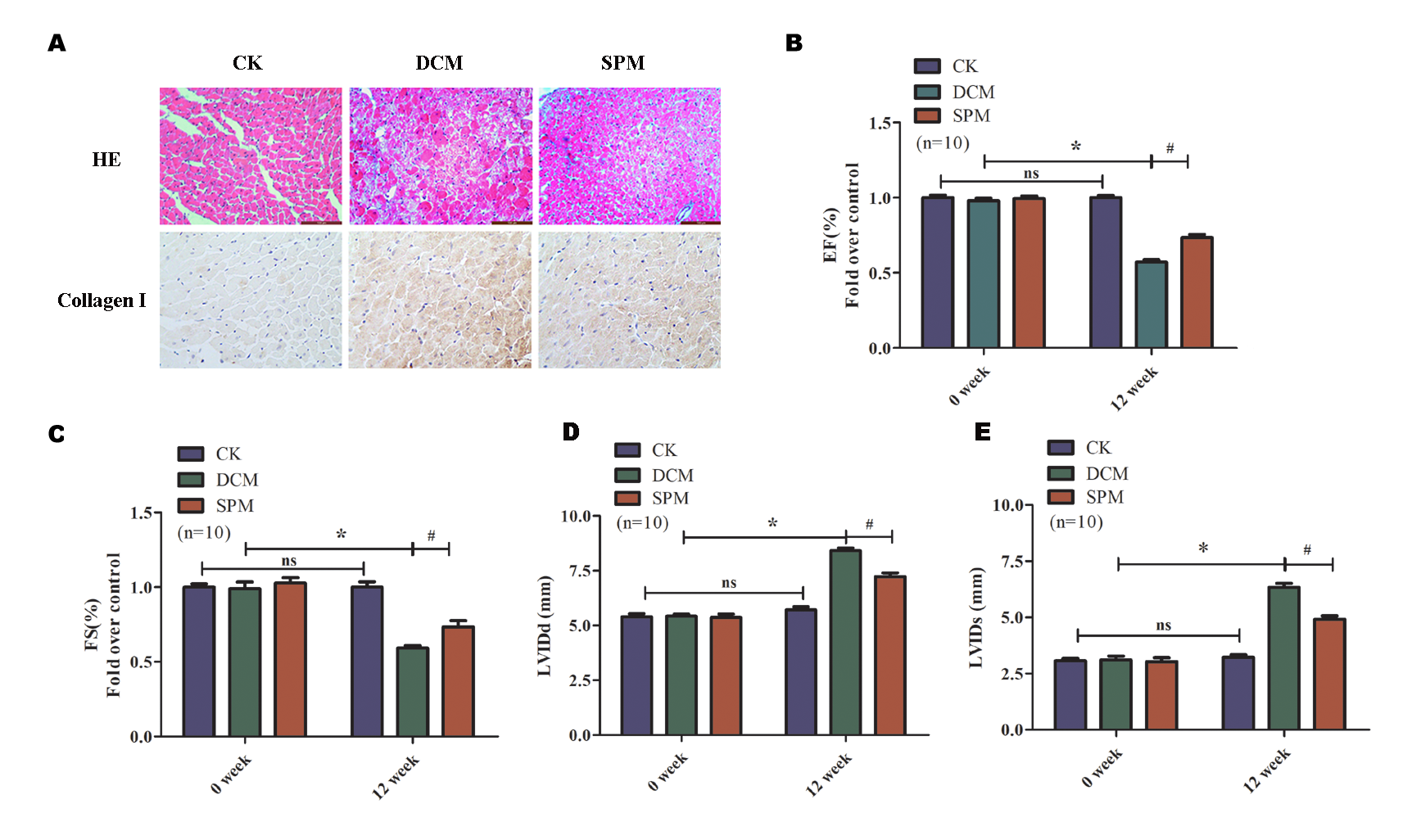

Supplement: Supplementary Figure 1 — The validation of the cardiac function in the model. (A–E) The cardiac function was validated in the CK, DCM, and SPM group. (A) The cardiac injury and fibrosis were analyzed using HE staining and Collagen I immunohistochemistry. The EF (B), FS (C), LVIDd (D), and LVIDs (E) were analyzed. [file Image_1.tif]
