# Supplementary material for: Spermine Regulates Immune and Signal Transduction Dysfunction in Diabetic Cardiomyopathy
Source: Front Endocrinol (Lausanne). 2022 Jan 31;12:740493. doi: 10.3389/fendo.2021.740493 (PMC8842652; doi:10.3389/fendo.2021.740493)
Supplement: Supplementary file 2 [file DataSheet_1.docx]

**Table SI. RNA-seq identified 1318 genes that differentially expressed in DCM compared to CK.**

| Gene_ID | DCM_FPKM | CK_FPKM | logFC | FDR | KEGG_ID | symbol | ncbi_descritption |
| --- | --- | --- | --- | --- | --- | --- | --- |
| ENSMUSG00000000031 | 45.649 | 24.056 | 1.011 | 0.000 |  | H19 | H19, imprinted maternally expressed transcript |
| ENSMUSG00000000296 | 10.432 | 8.216 | 0.436 | 0.012 | None | Tpd52l1 | tumor protein D52-like 1 |
| ENSMUSG00000000317 | 9.268 | 14.980 | -0.599 | 0.000 | None | Bcl6b | B cell CLL/lymphoma 6, member B |
| ENSMUSG00000000320 | 5.922 | 3.864 | 0.709 | 0.000 | K00458 | Alox12 | arachidonate 12-lipoxygenase |
| ENSMUSG00000000326 | 4.001 | 2.465 | 0.794 | 0.001 | K00545 | Comt | catechol-O-methyltransferase |
| ENSMUSG00000000386 | 0.262 | 0.605 | -1.108 | 0.038 | None | Mx1 | MX dynamin-like GTPase 1 |
| ENSMUSG00000000409 | 0.403 | 1.265 | -1.551 | 0.006 | K05856 | Lck | lymphocyte protein tyrosine kinase |
| ENSMUSG00000000682 | 6.022 | 17.076 | -1.411 | 0.000 | K06488 | Cd52 | CD52 antigen |
| ENSMUSG00000000805 | 68.195 | 48.536 | 0.584 | 0.000 | K18246 | Car4 | carbonic anhydrase 4 |
| ENSMUSG00000000958 | 2.664 | 4.890 | -0.782 | 0.000 | K13867 | Slc7a7 | solute carrier family 7 (cationic amino acid transporter, y+ system), member 7 |
| ENSMUSG00000001029 | 20.917 | 32.117 | -0.525 | 0.000 | K06523 | Icam2 | intercellular adhesion molecule 2 |
| ENSMUSG00000001119 | 30.729 | 49.124 | -0.584 | 0.000 | K06238 | Col6a1 | collagen, type VI, alpha 1 |
| ENSMUSG00000001123 | 27.761 | 41.556 | -0.490 | 0.000 | K10093 | Lgals9 | lectin, galactose binding, soluble 9 |
| ENSMUSG00000001227 | 9.824 | 6.113 | 0.779 | 0.000 | K06842 | Sema6b | sema domain, transmembrane domain (TM), and cytoplasmic domain, (semaphorin) 6B |
| ENSMUSG00000001228 | 0.300 | 0.875 | -1.453 | 0.000 | K10638 | Uhrf1 | ubiquitin-like, containing PHD and RING finger domains, 1 |
| ENSMUSG00000001247 | 2.114 | 1.379 | 0.707 | 0.035 | None | Lsr | lipolysis stimulated lipoprotein receptor |
| ENSMUSG00000001270 | 18.613 | 28.408 | -0.517 | 0.000 | K00933 | Ckb | creatine kinase, brain |
| ENSMUSG00000001281 | 1.065 | 1.910 | -0.747 | 0.044 | K06590 | Itgb7 | integrin beta 7 |
| ENSMUSG00000001334 | 90.880 | 139.646 | -0.526 | 0.000 | None | Fndc5 | fibronectin type III domain containing 5 |
| ENSMUSG00000001493 | 5.296 | 10.540 | -0.900 | 0.000 | K09322 | Meox1 | mesenchyme homeobox 1 |
| ENSMUSG00000001506 | 18.192 | 40.373 | -1.058 | 0.000 | K06236 | Col1a1 | collagen, type I, alpha 1 |
| ENSMUSG00000001555 | 13.808 | 8.991 | 0.712 | 0.000 | K09575 | Fkbp10 | FK506 binding protein 10 |
| ENSMUSG00000001739 | 10.599 | 7.106 | 0.669 | 0.000 | K06087 | Cldn15 | claudin 15 |
| ENSMUSG00000001774 | 1.926 | 3.008 | -0.552 | 0.007 | K16729 | Chordc1 | cysteine and histidine-rich domain (CHORD)-containing, zinc-binding protein 1 |
| ENSMUSG00000001930 | 50.554 | 31.045 | 0.793 | 0.000 | K03900 | Vwf | Von Willebrand factor |
| ENSMUSG00000001985 | 0.212 | 0.079 | 1.522 | 0.000 | K05203 | Grik3 | glutamate receptor, ionotropic, kainate 3 |
| ENSMUSG00000002111 | 4.321 | 7.604 | -0.722 | 0.005 | K09438 | Spi1 | spleen focus forming virus (SFFV) proviral integration oncogene |
| ENSMUSG00000002289 | 22.377 | 39.913 | -0.743 | 0.038 | K08767 | Angptl4 | angiopoietin-like 4 |
| ENSMUSG00000002325 | 6.868 | 12.495 | -0.770 | 0.000 | K04693 | Irf9 | interferon regulatory factor 9 |
| ENSMUSG00000002668 | 0.572 | 1.133 | -0.890 | 0.019 | K20160 | Dennd1c | DENN/MADD domain containing 1C |
| ENSMUSG00000002831 | 85.007 | 54.881 | 0.726 | 0.000 | K20254 | Plin4 | perilipin 4 |
| ENSMUSG00000002983 | 3.737 | 5.683 | -0.512 | 0.000 | K09253 | Relb | avian reticuloendotheliosis viral (v-rel) oncogene related B |
| ENSMUSG00000003206 | 0.285 | 1.286 | -2.056 | 0.002 | None | Ebi3 | Epstein-Barr virus induced gene 3 |
| ENSMUSG00000003283 | 1.562 | 2.946 | -0.819 | 0.011 | K08893 | Hck | hemopoietic cell kinase |
| ENSMUSG00000003418 | 0.606 | 0.412 | 0.649 | 0.010 | K06615 | St8sia6 | ST8 alpha-N-acetyl-neuraminide alpha-2,8-sialyltransferase 6 |
| ENSMUSG00000003477 | 80.366 | 27.493 | 1.640 | 0.000 | K00562 | Inmt | indolethylamine N-methyltransferase |
| ENSMUSG00000003549 | 25.791 | 20.671 | 0.412 | 0.001 | K10849 | Ercc1 | excision repair cross-complementing rodent repair deficiency, complementation group 1 |
| ENSMUSG00000003617 | 20.819 | 16.454 | 0.432 | 0.000 | K13624 | Cp | ceruloplasmin |
| ENSMUSG00000003779 | 0.411 | 0.799 | -0.862 | 0.048 | K10402 | Kif20a | kinesin family member 20A |
| ENSMUSG00000003849 | 51.666 | 34.053 | 0.693 | 0.000 | K00355 | Nqo1 | NAD(P)H dehydrogenase, quinone 1 |
| ENSMUSG00000003934 | 42.051 | 25.222 | 0.831 | 0.000 | K05463 | Efnb3 | ephrin B3 |
| ENSMUSG00000003949 | 4.359 | 3.187 | 0.545 | 0.000 | K09057 | Hlf | hepatic leukemia factor |
| ENSMUSG00000004098 | 6.590 | 9.789 | -0.478 | 0.003 | K19721 | Col5a3 | collagen, type V, alpha 3 |
| ENSMUSG00000004263 | 0.561 | 0.333 | 0.844 | 0.036 | K05626 | Atn1 | atrophin 1 |
| ENSMUSG00000004328 | 1.497 | 0.434 | 1.883 | 0.000 | K09096 | Hif3a | hypoxia inducible factor 3, alpha subunit |
| ENSMUSG00000004415 | 0.127 | 0.535 | -1.982 | 0.006 | None | Col26a1 | collagen, type XXVI, alpha 1 |
| ENSMUSG00000004698 | 2.313 | 3.524 | -0.515 | 0.001 | K11409 | Hdac9 | histone deacetylase 9 |
| ENSMUSG00000004730 | 5.120 | 10.415 | -0.932 | 0.011 | K04591 | Adgre1 | adhesion G protein-coupled receptor E1 |
| ENSMUSG00000004891 | 5.580 | 10.962 | -0.880 | 0.000 | K07609 | Nes | nestin |
| ENSMUSG00000005413 | 17.584 | 13.095 | 0.518 | 0.001 | K00510 | Hmox1 | heme oxygenase 1 |
| ENSMUSG00000005514 | 36.356 | 29.373 | 0.401 | 0.000 | K00327 | Por | P450 (cytochrome) oxidoreductase |
| ENSMUSG00000005540 | 0.231 | 0.826 | -1.739 | 0.021 | K06468 | Fcer2a | Fc receptor, IgE, low affinity II, alpha polypeptide |
| ENSMUSG00000005547 | 0.000 | 0.937 | -8.372 | 0.047 | K07411 | Cyp2a5 | cytochrome P450, family 2, subfamily a, polypeptide 5 |
| ENSMUSG00000005672 | 0.938 | 2.442 | -1.287 | 0.000 | K05091 | Kit | KIT proto-oncogene receptor tyrosine kinase |
| ENSMUSG00000005763 | 0.971 | 2.083 | -1.007 | 0.001 | K06453 | Cd247 | CD247 antigen |
| ENSMUSG00000005951 | 2.352 | 1.628 | 0.623 | 0.014 | K11214 | Shpk | sedoheptulokinase |
| ENSMUSG00000005958 | 3.840 | 2.941 | 0.478 | 0.004 | K05112 | Ephb3 | Eph receptor B3 |
| ENSMUSG00000006014 | 5.623 | 4.374 | 0.455 | 0.000 | None | Prg4 | proteoglycan 4 (megakaryocyte stimulating factor, articular superficial zone protein) |
| ENSMUSG00000006221 | 851.648 | 660.349 | 0.461 | 0.000 | K09546 | Hspb7 | heat shock protein family, member 7 (cardiovascular) |
| ENSMUSG00000006344 | 5.715 | 3.568 | 0.774 | 0.000 | K18592 | Ggt5 | gamma-glutamyltransferase 5 |
| ENSMUSG00000006403 | 0.681 | 1.267 | -0.800 | 0.023 | K07764 | Adamts4 | a disintegrin-like and metallopeptidase (reprolysin type) with thrombospondin type 1 motif, 4 |
| ENSMUSG00000006411 | 0.115 | 0.353 | -1.520 | 0.022 | K06593 | Nectin4 | nectin cell adhesion molecule 4 |
| ENSMUSG00000006462 | 1.896 | 1.204 | 0.751 | 0.043 |  | A530013C23Rik | RIKEN cDNA A530013C23 gene |
| ENSMUSG00000006476 | 5.997 | 8.584 | -0.424 | 0.000 | None | Nsmf | NMDA receptor synaptonuclear signaling and neuronal migration factor |
| ENSMUSG00000006494 | 53.679 | 41.761 | 0.456 | 0.000 | K12077 | Pdk1 | pyruvate dehydrogenase kinase, isoenzyme 1 |
| ENSMUSG00000006642 | 3.640 | 1.693 | 1.199 | 0.000 | None | Tcf23 | transcription factor 23 |
| ENSMUSG00000006784 | 0.141 | 0.028 | 2.406 | 0.012 | None | Ttc25 | tetratricopeptide repeat domain 25 |
| ENSMUSG00000007122 | 1.272 | 4.655 | -1.773 | 0.000 | None | Casq1 | calsequestrin 1 |
| ENSMUSG00000007279 | 4.122 | 5.785 | -0.397 | 0.022 | None | Scube2 | signal peptide, CUB domain, EGF-like 2 |
| ENSMUSG00000007659 | 47.327 | 26.775 | 0.916 | 0.000 | K04570 | Bcl2l1 | BCL2-like 1 |
| ENSMUSG00000007682 | 0.171 | 0.408 | -1.163 | 0.010 | K17904 | Dio2 | deiodinase, iodothyronine, type II |
| ENSMUSG00000007877 | 1902.512 | 1310.851 | 0.629 | 0.000 | K19879 | Tcap | titin-cap |
| ENSMUSG00000008193 | 0.199 | 0.864 | -2.023 | 0.002 | K09439 | Spib | Spi-B transcription factor (Spi-1/PU.1 related) |
| ENSMUSG00000008496 | 0.112 | 0.297 | -1.307 | 0.047 | K09364 | Pou2f2 | POU domain, class 2, transcription factor 2 |
| ENSMUSG00000008540 | 55.308 | 41.957 | 0.492 | 0.000 | K00799 | Mgst1 | microsomal glutathione S-transferase 1 |
| ENSMUSG00000008730 | 5.333 | 4.210 | 0.435 | 0.007 | K08826 | Hipk1 | homeodomain interacting protein kinase 1 |
| ENSMUSG00000008845 | 14.177 | 11.113 | 0.445 | 0.000 | K06545 | Cd163 | CD163 antigen |
| ENSMUSG00000008874 | 2.089 | 5.371 | -1.267 | 0.016 | K17519 | Clec3a | C-type lectin domain family 3, member a |
| ENSMUSG00000009013 | 12.610 | 19.845 | -0.562 | 0.000 | K10418 | Dynll1 | dynein light chain LC8-type 1 |
| ENSMUSG00000009739 | 6.076 | 4.866 | 0.413 | 0.000 | K09368 | Pou6f1 | POU domain, class 6, transcription factor 1 |
| ENSMUSG00000010095 | 28.261 | 21.663 | 0.476 | 0.000 | K06519 | Slc3a2 | solute carrier family 3 (activators of dibasic and neutral amino acid transport), member 2 |
| ENSMUSG00000010142 | 0.611 | 1.238 | -0.926 | 0.012 | K05150 | Tnfrsf13b | tumor necrosis factor receptor superfamily, member 13b |
| ENSMUSG00000010554 | 1.043 | 1.572 | -0.500 | 0.004 | K11393 | Mettl16 | methyltransferase like 16 |
| ENSMUSG00000010830 | 4.778 | 7.838 | -0.622 | 0.016 | K10949 | Kdelr3 | KDEL (Lys-Asp-Glu-Leu) endoplasmic reticulum protein retention receptor 3 |
| ENSMUSG00000011179 | 59.061 | 47.370 | 0.411 | 0.000 | K01581 | Odc1 | ornithine decarboxylase, structural 1 |
| ENSMUSG00000011256 | 3.751 | 6.842 | -0.773 | 0.000 | K08608 | Adam19 | a disintegrin and metallopeptidase domain 19 (meltrin beta) |
| ENSMUSG00000011305 | 154.567 | 108.245 | 0.608 | 0.000 | K20255 | Plin5 | perilipin 5 |
| ENSMUSG00000012076 | 4.493 | 6.412 | -0.421 | 0.010 | K19196 | Brms1l | breast cancer metastasis-suppressor 1-like |
| ENSMUSG00000013089 | 20.033 | 12.903 | 0.728 | 0.000 | K15593 | Etv5 | ets variant 5 |
| ENSMUSG00000013275 | 42.090 | 27.865 | 0.688 | 0.000 | K15122 | Slc41a1 | solute carrier family 41, member 1 |
| ENSMUSG00000013584 | 2.849 | 5.153 | -0.764 | 0.003 | K07249 | Aldh1a2 | aldehyde dehydrogenase family 1, subfamily A2 |
| ENSMUSG00000013698 | 53.454 | 82.470 | -0.533 | 0.000 | None | Pea15a | phosphoprotein enriched in astrocytes 15A |
| ENSMUSG00000013707 | 3.012 | 5.616 | -0.805 | 0.003 | None | Tnfaip8l2 | tumor necrosis factor, alpha-induced protein 8-like 2 |
| ENSMUSG00000014030 | 0.101 | 0.329 | -1.608 | 0.037 | K09383 | Pax5 | paired box 5 |
| ENSMUSG00000014503 | 5.089 | 7.566 | -0.480 | 0.006 | K04991 | Pkd2l2 | polycystic kidney disease 2-like 2 |
| ENSMUSG00000014773 | 2.738 | 4.294 | -0.556 | 0.002 | K06051 | Dll1 | delta like canonical Notch ligand 1 |
| ENSMUSG00000014813 | 1.562 | 1.046 | 0.670 | 0.032 | None | Stc1 | stanniocalcin 1 |
| ENSMUSG00000014846 | 23.026 | 34.199 | -0.477 | 0.000 | None | Tppp3 | tubulin polymerization-promoting protein family member 3 |
| ENSMUSG00000015090 | 375.001 | 199.879 | 1.004 | 0.002 | K01830 | Ptgds | prostaglandin D2 synthase (brain) |
| ENSMUSG00000015312 | 12.267 | 7.004 | 0.901 | 0.001 | K04402 | Gadd45b | growth arrest and DNA-damage-inducible 45 beta |
| ENSMUSG00000015340 | 3.375 | 5.732 | -0.672 | 0.000 | K08008 | Cybb | cytochrome b-245, beta polypeptide |
| ENSMUSG00000015355 | 2.566 | 4.813 | -0.813 | 0.006 | K06479 | Cd48 | CD48 antigen |
| ENSMUSG00000015365 | 17.614 | 12.264 | 0.616 | 0.000 | K13983 | Mov10l1 | Moloney leukemia virus 10-like 1 |
| ENSMUSG00000015396 | 4.559 | 7.349 | -0.596 | 0.003 | K06510 | Cd83 | CD83 antigen |
| ENSMUSG00000015766 | 9.270 | 6.490 | 0.608 | 0.000 | K17277 | Eps8 | epidermal growth factor receptor pathway substrate 8 |
| ENSMUSG00000015852 | 2.447 | 7.366 | -1.496 | 0.000 | K06727 | Fcrls | Fc receptor-like S, scavenger receptor |
| ENSMUSG00000015947 | 1.097 | 2.647 | -1.176 | 0.000 | K06498 | Fcgr1 | Fc receptor, IgG, high affinity I |
| ENSMUSG00000015957 | 6.130 | 3.881 | 0.752 | 0.000 | K01384 | Wnt11 | wingless-type MMTV integration site family, member 11 |
| ENSMUSG00000015968 | 0.368 | 0.733 | -0.902 | 0.000 | K04851 | Cacna1d | calcium channel, voltage-dependent, L type, alpha 1D subunit |
| ENSMUSG00000016262 | 1.304 | 2.324 | -0.741 | 0.038 | None | Sertad4 | SERTA domain containing 4 |
| ENSMUSG00000016494 | 15.162 | 23.034 | -0.511 | 0.000 | K06474 | Cd34 | CD34 antigen |
| ENSMUSG00000016984 | 0.314 | 0.631 | -0.916 | 0.022 | None | Etaa1 | Ewing tumor-associated antigen 1 |
| ENSMUSG00000017707 | 184.125 | 146.873 | 0.420 | 0.000 | None | Serinc3 | serine incorporator 3 |
| ENSMUSG00000017737 | 1.661 | 1.034 | 0.776 | 0.023 | K01403 | Mmp9 | matrix metallopeptidase 9 |
| ENSMUSG00000017897 | 2.871 | 2.091 | 0.549 | 0.019 | K17620 | Eya2 | EYA transcriptional coactivator and phosphatase 2 |
| ENSMUSG00000017950 | 0.000 | 0.047 | -5.178 | 0.012 | K07292 | Hnf4a | hepatic nuclear factor 4, alpha |
| ENSMUSG00000018008 | 4.534 | 8.832 | -0.870 | 0.024 | K18441 | Cyth4 | cytohesin 4 |
| ENSMUSG00000018168 | 0.228 | 0.544 | -1.156 | 0.035 | K09220 | Ikzf3 | IKAROS family zinc finger 3 |
| ENSMUSG00000018415 | 10.678 | 15.991 | -0.489 | 0.000 | None | Gid4 | GID complex subunit 4, VID24 homolog |
| ENSMUSG00000018427 | 4.646 | 9.044 | -0.867 | 0.000 | None | Ypel2 | yippee like 2 |
| ENSMUSG00000018574 | 403.310 | 310.089 | 0.472 | 0.000 | K09479 | Acadvl | acyl-Coenzyme A dehydrogenase, very long chain |
| ENSMUSG00000018593 | 172.351 | 368.303 | -1.003 | 0.000 | None | Sparc | secreted acidic cysteine rich glycoprotein |
| ENSMUSG00000018654 | 0.716 | 1.416 | -0.889 | 0.006 | K09220 | Ikzf1 | IKAROS family zinc finger 1 |
| ENSMUSG00000018740 | 0.881 | 1.720 | -0.873 | 0.000 | K15117 | Slc25a35 | solute carrier family 25, member 35 |
| ENSMUSG00000018809 | 4.868 | 3.715 | 0.483 | 0.003 | None | Smyd4 | SET and MYND domain containing 4 |
| ENSMUSG00000018899 | 14.367 | 21.156 | -0.467 | 0.040 | K09444 | Irf1 | interferon regulatory factor 1 |
| ENSMUSG00000018923 | 6.435 | 10.920 | -0.668 | 0.001 | K15131 | Med11 | mediator complex subunit 11 |
| ENSMUSG00000018924 | 0.019 | 0.228 | -3.451 | 0.006 | K00460 | Alox15 | arachidonate 15-lipoxygenase |
| ENSMUSG00000018931 | 5.240 | 7.289 | -0.383 | 0.007 | K06975 | Natd1 | N-acetyltransferase domain containing 1 |
| ENSMUSG00000018986 | 0.241 | 0.640 | -1.328 | 0.034 | None | Slfn3 | schlafen 3 |
| ENSMUSG00000019055 | 25.299 | 38.855 | -0.524 | 0.000 | K00473 | Plod1 | procollagen-lysine, 2-oxoglutarate 5-dioxygenase 1 |
| ENSMUSG00000019066 | 15.308 | 11.138 | 0.553 | 0.000 | K07884 | Rab3d | RAB3D, member RAS oncogene family |
| ENSMUSG00000019124 | 3.240 | 5.405 | -0.646 | 0.000 | K14358 | Scrn1 | secernin 1 |
| ENSMUSG00000019139 | 22.766 | 17.939 | 0.437 | 0.000 | K01858 | Isyna1 | myo-inositol 1-phosphate synthase A1 |
| ENSMUSG00000019235 | 1.596 | 3.408 | -1.002 | 0.000 | None | Rps6kl1 | ribosomal protein S6 kinase-like 1 |
| ENSMUSG00000019539 | 17.103 | 24.180 | -0.407 | 0.001 | None | Rcn3 | reticulocalbin 3, EF-hand calcium binding domain |
| ENSMUSG00000019577 | 627.105 | 907.267 | -0.439 | 0.002 | K00898 | Pdk4 | pyruvate dehydrogenase kinase, isoenzyme 4 |
| ENSMUSG00000019647 | 4.722 | 6.581 | -0.386 | 0.018 | K06842 | Sema6a | sema domain, transmembrane domain (TM), and cytoplasmic domain, (semaphorin) 6A |
| ENSMUSG00000019789 | 3.449 | 2.466 | 0.576 | 0.002 | K09091 | Hey2 | hairy/enhancer-of-split related with YRPW motif 2 |
| ENSMUSG00000019828 | 4.189 | 2.992 | 0.579 | 0.000 | K04603 | Grm1 | glutamate receptor, metabotropic 1 |
| ENSMUSG00000019851 | 89.993 | 68.946 | 0.479 | 0.000 | K10136 | Perp | PERP, TP53 apoptosis effector |
| ENSMUSG00000019916 | 6.701 | 10.725 | -0.585 | 0.000 | K00472 | P4ha1 | procollagen-proline, 2-oxoglutarate 4-dioxygenase (proline 4-hydroxylase), alpha 1 polypeptide |
| ENSMUSG00000019923 | 31.567 | 24.418 | 0.463 | 0.000 | K19943 | Zwint | ZW10 interactor |
| ENSMUSG00000019933 | 0.387 | 2.027 | -2.310 | 0.004 | None | Mrln | myoregulin |
| ENSMUSG00000019970 | 59.585 | 43.770 | 0.541 | 0.000 | K13302 | Sgk1 | serum/glucocorticoid regulated kinase 1 |
| ENSMUSG00000019990 | 2.801 | 4.158 | -0.477 | 0.012 | K18436 | Pde7b | phosphodiesterase 7B |
| ENSMUSG00000020077 | 18.970 | 26.306 | -0.379 | 0.004 | K06849 | Srgn | serglycin |
| ENSMUSG00000020100 | 1.738 | 2.559 | -0.465 | 0.007 | K15014 | Slc29a3 | solute carrier family 29 (nucleoside transporters), member 3 |
| ENSMUSG00000020108 | 136.117 | 75.717 | 0.942 | 0.004 | K08270 | Ddit4 | DNA-damage-inducible transcript 4 |
| ENSMUSG00000020143 | 1.091 | 2.040 | -0.809 | 0.000 | K12367 | Dock2 | dedicator of cyto-kinesis 2 |
| ENSMUSG00000020176 | 15.351 | 21.434 | -0.388 | 0.000 | K20064 | Grb10 | growth factor receptor bound protein 10 |
| ENSMUSG00000020181 | 0.952 | 1.513 | -0.574 | 0.004 | None | Nav3 | neuron navigator 3 |
| ENSMUSG00000020241 | 20.158 | 37.638 | -0.807 | 0.000 | K06238 | Col6a2 | collagen, type VI, alpha 2 |
| ENSMUSG00000020256 | 1.390 | 2.221 | -0.584 | 0.011 | K00289 | Aldh1l2 | aldehyde dehydrogenase 1 family, member L2 |
| ENSMUSG00000020264 | 7.536 | 11.947 | -0.573 | 0.000 | K14209 | Slc36a2 | solute carrier family 36 (proton/amino acid symporter), member 2 |
| ENSMUSG00000020272 | 5.350 | 7.662 | -0.425 | 0.000 | K08837 | Stk10 | serine/threonine kinase 10 |
| ENSMUSG00000020331 | 8.502 | 13.822 | -0.608 | 0.000 | K04955 | Hcn2 | hyperpolarization-activated, cyclic nucleotide-gated K+ 2 |
| ENSMUSG00000020374 | 0.440 | 0.222 | 1.074 | 0.044 | None | Rasgef1c | RasGEF domain family, member 1C |
| ENSMUSG00000020387 | 11.107 | 6.441 | 0.879 | 0.000 | None | Jade2 | jade family PHD finger 2 |
| ENSMUSG00000020395 | 0.091 | 0.294 | -1.610 | 0.008 | K07363 | Itk | IL2 inducible T cell kinase |
| ENSMUSG00000020407 | 10.821 | 7.908 | 0.548 | 0.011 | K00757 | Upp1 | uridine phosphorylase 1 |
| ENSMUSG00000020424 | 9.385 | 6.879 | 0.543 | 0.004 | None | Gatsl3 | GATS protein-like 3 |
| ENSMUSG00000020427 | 33.288 | 21.131 | 0.748 | 0.000 | K10138 | Igfbp3 | insulin-like growth factor binding protein 3 |
| ENSMUSG00000020437 | 0.907 | 1.485 | -0.618 | 0.047 | K10356 | Myo1g | myosin IG |
| ENSMUSG00000020453 | 11.876 | 7.411 | 0.773 | 0.000 | None | Patz1 | POZ (BTB) and AT hook containing zinc finger 1 |
| ENSMUSG00000020513 | 2.612 | 3.811 | -0.451 | 0.048 | K10390 | Tubd1 | tubulin, delta 1 |
| ENSMUSG00000020520 | 5.185 | 7.705 | -0.478 | 0.000 | K00710 | Galnt10 | polypeptide N-acetylgalactosaminyltransferase 10 |
| ENSMUSG00000020534 | 7.275 | 5.881 | 0.399 | 0.006 | K00600 | Shmt1 | serine hydroxymethyltransferase 1 (soluble) |
| ENSMUSG00000020542 | 10.223 | 8.326 | 0.388 | 0.001 | None | Myocd | myocardin |
| ENSMUSG00000020546 | 0.695 | 1.108 | -0.580 | 0.022 | K15302 | Stxbp4 | syntaxin binding protein 4 |
| ENSMUSG00000020577 | 34.071 | 51.015 | -0.489 | 0.000 | K17356 | Tspan13 | tetraspanin 13 |
| ENSMUSG00000020604 | 1.556 | 0.932 | 0.835 | 0.001 | K12381 | Arsg | arylsulfatase G |
| ENSMUSG00000020620 | 1.884 | 2.643 | -0.395 | 0.035 | K05650 | Abca8b | ATP-binding cassette, sub-family A (ABC1), member 8b |
| ENSMUSG00000020638 | 4.865 | 8.132 | -0.648 | 0.000 | K13809 | Cmpk2 | cytidine monophosphate (UMP-CMP) kinase 2, mitochondrial |
| ENSMUSG00000020641 | 6.861 | 10.259 | -0.488 | 0.004 | K15045 | Rsad2 | radical S-adenosyl methionine domain containing 2 |
| ENSMUSG00000020642 | 9.008 | 13.144 | -0.452 | 0.000 | K11975 | Rnf144a | ring finger protein 144A |
| ENSMUSG00000020656 | 0.282 | 0.116 | 1.372 | 0.025 | K09275 | Grhl1 | grainyhead like transcription factor 1 |
| ENSMUSG00000020674 | 18.079 | 27.242 | -0.499 | 0.000 | K19511 | Pxdn | peroxidasin |
| ENSMUSG00000020676 | 3.108 | 1.623 | 1.027 | 0.025 | K16597 | Ccl11 | chemokine (C-C motif) ligand 11 |
| ENSMUSG00000020682 | 5.110 | 3.952 | 0.464 | 0.006 | K08006 | Mmp28 | matrix metallopeptidase 28 (epilysin) |
| ENSMUSG00000020695 | 5.797 | 9.011 | -0.543 | 0.000 | K06560 | Mrc2 | mannose receptor, C type 2 |
| ENSMUSG00000020737 | 65.322 | 51.430 | 0.438 | 0.000 | None | Jpt1 | Jupiter microtubule associated homolog 1 |
| ENSMUSG00000020776 | 4.032 | 5.612 | -0.384 | 0.006 | K16471 | Fbf1 | Fas (TNFRSF6) binding factor 1 |
| ENSMUSG00000020777 | 153.978 | 118.741 | 0.469 | 0.000 | K00232 | Acox1 | acyl-Coenzyme A oxidase 1, palmitoyl |
| ENSMUSG00000020782 | 9.084 | 6.973 | 0.476 | 0.002 | K06094 | Llgl2 | LLGL2 scribble cell polarity complex component |
| ENSMUSG00000020788 | 16.252 | 11.917 | 0.540 | 0.000 | K05853 | Atp2a3 | ATPase, Ca++ transporting, ubiquitous |
| ENSMUSG00000020814 | 15.118 | 22.364 | -0.472 | 0.000 | None | Mxra7 | matrix-remodelling associated 7 |
| ENSMUSG00000020826 | 5.006 | 7.617 | -0.512 | 0.001 | K13241 | Nos2 | nitric oxide synthase 2, inducible |
| ENSMUSG00000020846 | 14.687 | 32.392 | -1.047 | 0.000 | None | Rflnb | refilin B |
| ENSMUSG00000020848 | 0.662 | 0.170 | 2.061 | 0.000 | K19917 | Doc2b | double C2, beta |
| ENSMUSG00000020866 | 1.993 | 3.311 | -0.640 | 0.000 | K04854 | Cacna1g | calcium channel, voltage-dependent, T type, alpha 1G subunit |
| ENSMUSG00000020887 | 0.331 | 0.085 | 2.056 | 0.021 |  | A230052G05Rik | RIKEN cDNA A230052G05 gene |
| ENSMUSG00000020889 | 40.202 | 25.713 | 0.738 | 0.000 | K03728 | Nr1d1 | nuclear receptor subfamily 1, group D, member 1 |
| ENSMUSG00000020893 | 29.099 | 15.844 | 0.969 | 0.004 | K02633 | Per1 | period circadian clock 1 |
| ENSMUSG00000020900 | 7.414 | 10.442 | -0.401 | 0.000 | K10352 | Myh10 | myosin, heavy polypeptide 10, non-muscle |
| ENSMUSG00000020902 | 56.641 | 31.283 | 0.950 | 0.000 | K06843 | Ntn1 | netrin 1 |
| ENSMUSG00000020914 | 0.467 | 0.873 | -0.808 | 0.041 | K03164 | Top2a | topoisomerase (DNA) II alpha |
| ENSMUSG00000020937 | 8.829 | 6.395 | 0.559 | 0.000 | K05857 | Plcd3 | phospholipase C, delta 3 |
| ENSMUSG00000020990 | 13.023 | 9.462 | 0.554 | 0.000 | K08824 | Cdkl1 | cyclin-dependent kinase-like 1 (CDC2-related kinase) |
| ENSMUSG00000021025 | 65.986 | 46.418 | 0.603 | 0.000 | K04734 | Nfkbia | nuclear factor of kappa light polypeptide gene enhancer in B cells inhibitor, alpha |
| ENSMUSG00000021057 | 0.169 | 0.504 | -1.482 | 0.000 | K16522 | Akap5 | A kinase (PRKA) anchor protein 5 |
| ENSMUSG00000021091 | 15.830 | 7.645 | 1.141 | 0.010 | K04525 | Serpina3n | serine (or cysteine) peptidase inhibitor, clade A, member 3N |
| ENSMUSG00000021134 | 52.846 | 37.746 | 0.577 | 0.000 | K12893 | Srsf5 | serine/arginine-rich splicing factor 5 |
| ENSMUSG00000021135 | 0.025 | 0.219 | -2.993 | 0.042 | K14341 | Slc10a1 | solute carrier family 10 (sodium/bile acid cotransporter family), member 1 |
| ENSMUSG00000021136 | 9.979 | 7.774 | 0.450 | 0.016 | None | Smoc1 | SPARC related modular calcium binding 1 |
| ENSMUSG00000021182 | 2.527 | 3.695 | -0.456 | 0.000 | None | Ccdc88c | coiled-coil domain containing 88C |
| ENSMUSG00000021219 | 2.521 | 3.980 | -0.566 | 0.000 | K16449 | Rgs6 | regulator of G-protein signaling 6 |
| ENSMUSG00000021223 | 4.869 | 8.056 | -0.633 | 0.000 | None | Papln | papilin, proteoglycan-like sulfated glycoprotein |
| ENSMUSG00000021226 | 101.191 | 70.697 | 0.611 | 0.000 | K01068 | Acot2 | acyl-CoA thioesterase 2 |
| ENSMUSG00000021236 | 45.458 | 36.633 | 0.403 | 0.000 | K01511 | Entpd5 | ectonucleoside triphosphate diphosphohydrolase 5 |
| ENSMUSG00000021255 | 13.146 | 10.323 | 0.442 | 0.000 | K08553 | Esrrb | estrogen related receptor, beta |
| ENSMUSG00000021256 | 4.466 | 10.537 | -1.146 | 0.000 | None | Vash1 | vasohibin 1 |
| ENSMUSG00000021260 | 1.055 | 0.497 | 1.178 | 0.000 | None | Hhipl1 | hedgehog interacting protein-like 1 |
| ENSMUSG00000021262 | 3.912 | 6.728 | -0.688 | 0.000 | None | Evl | Ena-vasodilator stimulated phosphoprotein |
| ENSMUSG00000021277 | 3.099 | 4.626 | -0.485 | 0.001 | K03174 | Traf3 | TNF receptor-associated factor 3 |
| ENSMUSG00000021280 | 2.275 | 1.625 | 0.578 | 0.049 | None | Exoc3l4 | exocyst complex component 3-like 4 |
| ENSMUSG00000021281 | 6.171 | 8.651 | -0.395 | 0.044 | K19989 | Tnfaip2 | tumor necrosis factor, alpha-induced protein 2 |
| ENSMUSG00000021338 | 1.550 | 1.089 | 0.600 | 0.004 | K20493 | Carmil1 | capping protein regulator and myosin 1 linker 1 |
| ENSMUSG00000021417 | 22.369 | 17.603 | 0.439 | 0.000 | K13239 | Eci2 | enoyl-Coenzyme A delta isomerase 2 |
| ENSMUSG00000021423 | 1.535 | 3.795 | -1.212 | 0.000 | None | Ly86 | lymphocyte antigen 86 |
| ENSMUSG00000021456 | 50.981 | 31.796 | 0.774 | 0.000 | K03841 | Fbp2 | fructose bisphosphatase 2 |
| ENSMUSG00000021481 | 15.179 | 12.298 | 0.396 | 0.000 | None | Zfp346 | zinc finger protein 346 |
| ENSMUSG00000021575 | 1.360 | 1.003 | 0.529 | 0.018 | K09094 | Ahrr | aryl-hydrocarbon receptor repressor |
| ENSMUSG00000021604 | 18.302 | 14.306 | 0.449 | 0.000 | None | Irx4 | Iroquois homeobox 4 |
| ENSMUSG00000021608 | 4.454 | 6.335 | -0.415 | 0.007 | K13510 | Lpcat1 | lysophosphatidylcholine acyltransferase 1 |
| ENSMUSG00000021614 | 0.961 | 1.587 | -0.631 | 0.000 | K06793 | Vcan | versican |
| ENSMUSG00000021638 | 0.464 | 0.244 | 1.019 | 0.044 | K06088 | Ocln | occludin |
| ENSMUSG00000021709 | 6.155 | 5.020 | 0.385 | 0.045 | K12796 | Erbin | Erbb2 interacting protein |
| ENSMUSG00000021750 | 10.097 | 3.048 | 1.824 | 0.000 | None | Fam107a | family with sequence similarity 107, member A |
| ENSMUSG00000021880 | 0.288 | 0.822 | -1.424 | 0.008 | K01172 | Rnase6 | ribonuclease, RNase A family, 6 |
| ENSMUSG00000021903 | 25.745 | 14.527 | 0.919 | 0.000 | K00710 | Galnt15 | polypeptide N-acetylgalactosaminyltransferase 15 |
| ENSMUSG00000021904 | 22.525 | 17.891 | 0.426 | 0.000 | K06840 | Sema3g | sema domain, immunoglobulin domain (Ig), short basic domain, secreted, (semaphorin) 3G |
| ENSMUSG00000021943 | 1.191 | 0.807 | 0.657 | 0.004 | K05496 | Gdf10 | growth differentiation factor 10 |
| ENSMUSG00000021998 | 7.787 | 13.837 | -0.737 | 0.000 | K17276 | Lcp1 | lymphocyte cytosolic protein 1 |
| ENSMUSG00000022014 | 1.249 | 2.594 | -0.961 | 0.004 | None | Epsti1 | epithelial stromal interaction 1 (breast) |
| ENSMUSG00000022018 | 129.200 | 95.380 | 0.533 | 0.000 | None | Rgcc | regulator of cell cycle |
| ENSMUSG00000022025 | 4.254 | 1.606 | 1.501 | 0.000 | None | Cnmd | chondromodulin |
| ENSMUSG00000022040 | 267.049 | 186.188 | 0.614 | 0.000 | K08726 | Ephx2 | epoxide hydrolase 2, cytoplasmic |
| ENSMUSG00000022041 | 3.662 | 2.541 | 0.620 | 0.005 | K04804 | Chrna2 | cholinergic receptor, nicotinic, alpha polypeptide 2 (neuronal) |
| ENSMUSG00000022096 | 2.674 | 5.160 | -0.856 | 0.000 | K00478 | Hr | hairless |
| ENSMUSG00000022098 | 6.896 | 10.164 | -0.467 | 0.000 | K05502 | Bmp1 | bone morphogenetic protein 1 |
| ENSMUSG00000022102 | 1.846 | 3.400 | -0.786 | 0.011 | K20234 | Dok2 | docking protein 2 |
| ENSMUSG00000022103 | 0.526 | 1.191 | -1.086 | 0.000 | None | Gfra2 | glial cell line derived neurotrophic factor family receptor alpha 2 |
| ENSMUSG00000022106 | 4.500 | 6.402 | -0.417 | 0.001 | K11494 | Rcbtb2 | regulator of chromosome condensation (RCC1) and BTB (POZ) domain containing protein 2 |
| ENSMUSG00000022148 | 0.466 | 0.918 | -0.883 | 0.030 | K17698 | Fyb | FYN binding protein |
| ENSMUSG00000022186 | 168.000 | 271.718 | -0.599 | 0.000 | K01027 | Oxct1 | 3-oxoacid CoA transferase 1 |
| ENSMUSG00000022210 | 92.404 | 74.587 | 0.403 | 0.000 | K11147 | Dhrs4 | dehydrogenase/reductase (SDR family) member 4 |
| ENSMUSG00000022211 | 1.512 | 1.173 | 0.460 | 0.022 | K20493 | Carmil3 | capping protein regulator and myosin 1 linker 3 |
| ENSMUSG00000022231 | 3.234 | 1.673 | 1.044 | 0.000 | K06841 | Sema5a | sema domain, seven thrombospondin repeats (type 1 and type 1-like), transmembrane domain (TM) and short cytoplasmic domain, (semaphorin) 5A |
| ENSMUSG00000022324 | 7.575 | 10.789 | -0.417 | 0.003 | None | Matn2 | matrilin 2 |
| ENSMUSG00000022364 | 0.894 | 1.700 | -0.832 | 0.000 | None | Tbc1d31 | TBC1 domain family, member 31 |
| ENSMUSG00000022371 | 3.740 | 6.136 | -0.622 | 0.000 | K08133 | Col14a1 | collagen, type XIV, alpha 1 |
| ENSMUSG00000022383 | 11.243 | 7.843 | 0.611 | 0.000 | K07294 | Ppara | peroxisome proliferator activated receptor alpha |
| ENSMUSG00000022389 | 49.637 | 33.305 | 0.668 | 0.000 | K09058 | Tef | thyrotroph embryonic factor |
| ENSMUSG00000022421 | 1.695 | 1.192 | 0.602 | 0.006 | None | Nptxr | neuronal pentraxin receptor |
| ENSMUSG00000022425 | 9.694 | 7.752 | 0.415 | 0.001 | K01122 | Enpp2 | ectonucleotide pyrophosphatase/phosphodiesterase 2 |
| ENSMUSG00000022440 | 3.924 | 7.640 | -0.867 | 0.000 | K19470 | C1qtnf6 | C1q and tumor necrosis factor related protein 6 |
| ENSMUSG00000022443 | 30.266 | 44.524 | -0.463 | 0.000 | K10352 | Myh9 | myosin, heavy polypeptide 9, non-muscle |
| ENSMUSG00000022488 | 2.557 | 4.997 | -0.872 | 0.000 | K05750 | Nckap1l | NCK associated protein 1 like |
| ENSMUSG00000022504 | 0.057 | 0.204 | -1.721 | 0.015 | K08060 | Ciita | class II transactivator |
| ENSMUSG00000022510 | 0.361 | 0.167 | 1.203 | 0.006 | K10149 | Trp63 | transformation related protein 63 |
| ENSMUSG00000022519 | 131.607 | 188.404 | -0.424 | 0.000 | None | Srl | sarcalumenin |
| ENSMUSG00000022523 | 0.600 | 1.138 | -0.830 | 0.043 | K04358 | Fgf12 | fibroblast growth factor 12 |
| ENSMUSG00000022548 | 11.323 | 6.670 | 0.858 | 0.026 | K03098 | Apod | apolipoprotein D |
| ENSMUSG00000022579 | 118.117 | 95.546 | 0.400 | 0.002 | K20001 | Gpihbp1 | GPI-anchored HDL-binding protein 1 |
| ENSMUSG00000022754 | 1.027 | 1.835 | -0.742 | 0.010 | None | Tmem45a | transmembrane protein 45a |
| ENSMUSG00000022769 | 4.053 | 7.016 | -0.699 | 0.010 | None | Sdf2l1 | stromal cell-derived factor 2-like 1 |
| ENSMUSG00000022774 | 8.293 | 11.935 | -0.433 | 0.002 | K12883 | Ncbp2 | nuclear cap binding protein subunit 2 |
| ENSMUSG00000022803 | 293.849 | 228.290 | 0.458 | 0.000 | None | Popdc2 | popeye domain containing 2 |
| ENSMUSG00000022816 | 26.308 | 42.673 | -0.606 | 0.000 | None | Fstl1 | follistatin-like 1 |
| ENSMUSG00000022817 | 94.674 | 77.371 | 0.385 | 0.000 | K06588 | Itgb5 | integrin beta 5 |
| ENSMUSG00000022831 | 7.830 | 11.947 | -0.517 | 0.005 | K06106 | Hcls1 | hematopoietic cell specific Lyn substrate 1 |
| ENSMUSG00000022844 | 3.275 | 4.944 | -0.499 | 0.014 | K09583 | Pdia5 | protein disulfide isomerase associated 5 |
| ENSMUSG00000022856 | 13.606 | 10.921 | 0.410 | 0.019 | None | Tmem41a | transmembrane protein 41a |
| ENSMUSG00000022863 | 6.271 | 4.242 | 0.657 | 0.000 | K14443 | Btg3 | B cell translocation gene 3 |
| ENSMUSG00000022868 | 18.644 | 7.934 | 1.324 | 0.000 | None | Ahsg | alpha-2-HS-glycoprotein |
| ENSMUSG00000022906 | 3.170 | 4.810 | -0.509 | 0.001 | K15260 | Parp9 | poly (ADP-ribose) polymerase family, member 9 |
| ENSMUSG00000022952 | 0.393 | 0.691 | -0.717 | 0.022 | K08367 | Runx1 | runt related transcription factor 1 |
| ENSMUSG00000022965 | 36.202 | 56.268 | -0.544 | 0.000 | K05133 | Ifngr2 | interferon gamma receptor 2 |
| ENSMUSG00000022982 | 91.234 | 74.751 | 0.382 | 0.000 | K04565 | Sod1 | superoxide dismutase 1, soluble |
| ENSMUSG00000022994 | 57.658 | 43.626 | 0.496 | 0.000 | K08046 | Adcy6 | adenylate cyclase 6 |
| ENSMUSG00000023015 | 0.652 | 1.198 | -0.785 | 0.020 | K16733 | Racgap1 | Rac GTPase-activating protein 1 |
| ENSMUSG00000023019 | 2.914 | 1.888 | 0.717 | 0.002 | K00006 | Gpd1 | glycerol-3-phosphate dehydrogenase 1 (soluble) |
| ENSMUSG00000023067 | 64.620 | 34.208 | 1.012 | 0.000 | K06625 | Cdkn1a | cyclin-dependent kinase inhibitor 1A (P21) |
| ENSMUSG00000023087 | 2.174 | 1.609 | 0.528 | 0.005 | K18764 | Noct | nocturnin |
| ENSMUSG00000023132 | 0.409 | 1.504 | -1.770 | 0.009 | K01352 | Gzma | granzyme A |
| ENSMUSG00000023147 | 4.022 | 5.680 | -0.404 | 0.007 | None | Wrb | tryptophan rich basic protein |
| ENSMUSG00000023336 | 4.876 | 10.287 | -0.985 | 0.000 | None | Wfdc1 | WAP four-disulfide core domain 1 |
| ENSMUSG00000023439 | 13.456 | 9.741 | 0.563 | 0.007 | K07825 | Gnb3 | guanine nucleotide binding protein (G protein), beta 3 |
| ENSMUSG00000023826 | 2.150 | 1.632 | 0.490 | 0.014 | K04556 | Park2 | Parkinson disease (autosomal recessive, juvenile) 2, parkin |
| ENSMUSG00000023829 | 0.709 | 1.565 | -1.048 | 0.001 | K08198 | Slc22a1 | solute carrier family 22 (organic cation transporter), member 1 |
| ENSMUSG00000023885 | 16.677 | 8.837 | 1.011 | 0.000 | K04659 | Thbs2 | thrombospondin 2 |
| ENSMUSG00000023886 | 21.706 | 30.575 | -0.401 | 0.000 | None | Smoc2 | SPARC related modular calcium binding 2 |
| ENSMUSG00000023999 | 1.069 | 1.796 | -0.658 | 0.032 | K10397 | Kif6 | kinesin family member 6 |
| ENSMUSG00000024011 | 13.669 | 24.980 | -0.776 | 0.000 | K20412 | Pi16 | peptidase inhibitor 16 |
| ENSMUSG00000024013 | 1.731 | 3.231 | -0.810 | 0.027 | K05721 | Fgd2 | FYVE, RhoGEF and PH domain containing 2 |
| ENSMUSG00000024066 | 35.554 | 27.126 | 0.485 | 0.000 | K00106 | Xdh | xanthine dehydrogenase |
| ENSMUSG00000024076 | 5.533 | 4.021 | 0.552 | 0.000 | None | Vit | vitrin |
| ENSMUSG00000024087 | 5.823 | 2.896 | 1.101 | 0.000 | K07410 | Cyp1b1 | cytochrome P450, family 1, subfamily b, polypeptide 1 |
| ENSMUSG00000024112 | 6.449 | 4.507 | 0.612 | 0.000 | K04855 | Cacna1h | calcium channel, voltage-dependent, T type, alpha 1H subunit |
| ENSMUSG00000024160 | 5.358 | 4.098 | 0.479 | 0.007 | K10345 | Spsb3 | splA/ryanodine receptor domain and SOCS box containing 3 |
| ENSMUSG00000024180 | 4.665 | 3.159 | 0.655 | 0.000 | None | Tmem8 | transmembrane protein 8 (five membrane-spanning domains) |
| ENSMUSG00000024197 | 92.845 | 69.896 | 0.504 | 0.000 | K20287 | Plin3 | perilipin 3 |
| ENSMUSG00000024222 | 30.195 | 9.022 | 1.838 | 0.000 | K09571 | Fkbp5 | FK506 binding protein 5 |
| ENSMUSG00000024270 | 2.108 | 1.658 | 0.440 | 0.047 | K14712 | Slc39a6 | solute carrier family 39 (metal ion transporter), member 6 |
| ENSMUSG00000024300 | 2.018 | 3.146 | -0.547 | 0.007 | K10356 | Myo1f | myosin IF |
| ENSMUSG00000024338 | 2.889 | 4.984 | -0.692 | 0.002 | K02740 | Psmb8 | proteasome (prosome, macropain) subunit, beta type 8 (large multifunctional peptidase 7) |
| ENSMUSG00000024349 | 3.523 | 5.442 | -0.534 | 0.010 | K12654 | Tmem173 | transmembrane protein 173 |
| ENSMUSG00000024378 | 1.972 | 3.106 | -0.561 | 0.001 | None | Stard4 | StAR-related lipid transfer (START) domain containing 4 |
| ENSMUSG00000024397 | 2.070 | 5.041 | -1.189 | 0.006 | K18617 | Aif1 | allograft inflammatory factor 1 |
| ENSMUSG00000024421 | 0.366 | 0.636 | -0.707 | 0.003 | K06240 | Lama3 | laminin, alpha 3 |
| ENSMUSG00000024440 | 7.315 | 15.472 | -0.988 | 0.000 | K16499 | Pcdh12 | protocadherin 12 |
| ENSMUSG00000024481 | 2.067 | 0.919 | 1.260 | 0.000 | K13724 | Lvrn | laeverin |
| ENSMUSG00000024525 | 35.908 | 26.017 | 0.558 | 0.000 | K01092 | Impa2 | inositol (myo)-1(or 4)-monophosphatase 2 |
| ENSMUSG00000024526 | 31.328 | 21.769 | 0.619 | 0.000 | None | Cidea | cell death-inducing DNA fragmentation factor, alpha subunit-like effector A |
| ENSMUSG00000024529 | 3.365 | 2.008 | 0.836 | 0.000 | K00277 | Lox | lysyl oxidase |
| ENSMUSG00000024538 | 15.951 | 29.991 | -0.818 | 0.000 | K09563 | Ppic | peptidylprolyl isomerase C |
| ENSMUSG00000024558 | 3.110 | 1.231 | 1.431 | 0.000 | K06855 | Mapk4 | mitogen-activated protein kinase 4 |
| ENSMUSG00000024561 | 21.464 | 15.526 | 0.561 | 0.000 | K11589 | Mbd1 | methyl-CpG binding domain protein 1 |
| ENSMUSG00000024590 | 2.926 | 4.176 | -0.420 | 0.026 | K07611 | Lmnb1 | lamin B1 |
| ENSMUSG00000024610 | 96.131 | 264.284 | -1.371 | 0.000 | K06505 | Cd74 | CD74 antigen (invariant polypeptide of major histocompatibility complex, class II antigen-associated) |
| ENSMUSG00000024621 | 19.446 | 29.006 | -0.484 | 0.001 | K05090 | Csf1r | colony stimulating factor 1 receptor |
| ENSMUSG00000024640 | 3.852 | 2.230 | 0.884 | 0.000 | K00831 | Psat1 | phosphoserine aminotransferase 1 |
| ENSMUSG00000024736 | 1.543 | 2.707 | -0.719 | 0.003 | K17599 | Tmem132a | transmembrane protein 132A |
| ENSMUSG00000024737 | 1.297 | 1.950 | -0.494 | 0.041 | K14638 | Slc15a3 | solute carrier family 15, member 3 |
| ENSMUSG00000024766 | 1.493 | 2.279 | -0.518 | 0.033 | None | Lipo3 | lipase, member O3 |
| ENSMUSG00000024827 | 0.468 | 0.174 | 1.514 | 0.000 | K00281 | Gldc | glycine decarboxylase |
| ENSMUSG00000024835 | 2.668 | 0.919 | 1.632 | 0.000 | K13886 | Coro1b | coronin, actin binding protein 1B |
| ENSMUSG00000024843 | 2.205 | 1.694 | 0.473 | 0.014 | K14156 | Chka | choline kinase alpha |
| ENSMUSG00000024900 | 47.204 | 33.738 | 0.577 | 0.000 | K08765 | Cpt1a | carnitine palmitoyltransferase 1a, liver |
| ENSMUSG00000024901 | 2.423 | 1.539 | 0.747 | 0.011 | K11964 | Peli3 | pellino 3 |
| ENSMUSG00000024903 | 0.268 | 0.004 | 5.868 | 0.000 | K03334 | Lao1 | L-amino acid oxidase 1 |
| ENSMUSG00000024910 | 0.383 | 1.071 | -1.378 | 0.027 | K08569 | Ctsw | cathepsin W |
| ENSMUSG00000025017 | 1.794 | 3.061 | -0.677 | 0.034 | K12230 | Pik3ap1 | phosphoinositide-3-kinase adaptor protein 1 |
| ENSMUSG00000025089 | 5.155 | 3.990 | 0.463 | 0.000 | K19895 | Gfra1 | glial cell line derived neurotrophic factor family receptor alpha 1 |
| ENSMUSG00000025178 | 14.584 | 11.301 | 0.461 | 0.000 | K13711 | Pi4k2a | phosphatidylinositol 4-kinase type 2 alpha |
| ENSMUSG00000025184 | 2.008 | 2.913 | -0.445 | 0.046 | None | R3hcc1l | R3H domain and coiled-coil containing 1 like |
| ENSMUSG00000025190 | 509.764 | 383.091 | 0.505 | 0.000 | K14454 | Got1 | glutamic-oxaloacetic transaminase 1, soluble |
| ENSMUSG00000025223 | 41.973 | 33.702 | 0.410 | 0.000 | K15617 | Ldb1 | LIM domain binding 1 |
| ENSMUSG00000025229 | 0.992 | 0.450 | 1.234 | 0.035 | K09357 | Pitx3 | paired-like homeodomain transcription factor 3 |
| ENSMUSG00000025239 | 26.811 | 21.948 | 0.382 | 0.000 | K16682 | Limd1 | LIM domains containing 1 |
| ENSMUSG00000025279 | 0.037 | 0.230 | -2.555 | 0.001 | K11995 | Dnase1l3 | deoxyribonuclease 1-like 3 |
| ENSMUSG00000025351 | 54.805 | 44.764 | 0.384 | 0.000 | K06497 | Cd63 | CD63 antigen |
| ENSMUSG00000025372 | 5.170 | 4.229 | 0.384 | 0.037 | K05627 | Baiap2 | brain-specific angiogenesis inhibitor 1-associated protein 2 |
| ENSMUSG00000025422 | 3.205 | 1.896 | 0.852 | 0.000 | K17848 | Agap2 | ArfGAP with GTPase domain, ankyrin repeat and PH domain 2 |
| ENSMUSG00000025428 | 54.171 | 44.224 | 0.387 | 0.002 | K02132 | Atp5a1 | ATP synthase, H+ transporting, mitochondrial F1 complex, alpha subunit 1 |
| ENSMUSG00000025491 | 8.162 | 5.123 | 0.765 | 0.021 | K19831 | Ifitm1 | interferon induced transmembrane protein 1 |
| ENSMUSG00000025509 | 454.846 | 299.261 | 0.697 | 0.000 | K16816 | Pnpla2 | patatin-like phospholipase domain containing 2 |
| ENSMUSG00000025511 | 54.578 | 35.003 | 0.733 | 0.000 | K17294 | Tspan4 | tetraspanin 4 |
| ENSMUSG00000025512 | 3.021 | 4.240 | -0.395 | 0.006 | K17525 | Chid1 | chitinase domain containing 1 |
| ENSMUSG00000025586 | 7.567 | 5.008 | 0.689 | 0.000 | K02602 | Cpeb1 | cytoplasmic polyadenylation element binding protein 1 |
| ENSMUSG00000025648 | 2.112 | 3.077 | -0.449 | 0.025 | K19030 | Pfkfb4 | 6-phosphofructo-2-kinase/fructose-2,6-biphosphatase 4 |
| ENSMUSG00000025716 | 0.861 | 1.348 | -0.554 | 0.018 | K08834 | Myo3a | myosin IIIA |
| ENSMUSG00000025745 | 643.603 | 512.538 | 0.422 | 0.000 | K07515 | Hadha | hydroxyacyl-Coenzyme A dehydrogenase/3-ketoacyl-Coenzyme A thiolase/enoyl-Coenzyme A hydratase (trifunctional protein), alpha subunit |
| ENSMUSG00000025780 | 1.931 | 3.461 | -0.749 | 0.000 | None | Itih5 | inter-alpha (globulin) inhibitor H5 |
| ENSMUSG00000025792 | 13.007 | 8.705 | 0.672 | 0.000 | K13577 | Slc25a10 | solute carrier family 25 (mitochondrial carrier, dicarboxylate transporter), member 10 |
| ENSMUSG00000025815 | 1.119 | 0.840 | 0.504 | 0.048 | K15791 | Dhtkd1 | dehydrogenase E1 and transketolase domain containing 1 |
| ENSMUSG00000025823 | 10.091 | 14.222 | -0.402 | 0.002 | K09582 | Pdia4 | protein disulfide isomerase associated 4 |
| ENSMUSG00000025825 | 100.244 | 81.916 | 0.384 | 0.000 | None | Iscu | iron-sulfur cluster assembly enzyme |
| ENSMUSG00000025909 | 0.811 | 0.530 | 0.705 | 0.044 | None | Sntg1 | syntrophin, gamma 1 |
| ENSMUSG00000025969 | 9.321 | 15.315 | -0.623 | 0.000 | K06819 | Nrp2 | neuropilin 2 |
| ENSMUSG00000026003 | 748.744 | 544.425 | 0.553 | 0.000 | K00255 | Acadl | acyl-Coenzyme A dehydrogenase, long-chain |
| ENSMUSG00000026014 | 7.270 | 5.609 | 0.467 | 0.000 | None | Raph1 | Ras association (RalGDS/AF-6) and pleckstrin homology domains 1 |
| ENSMUSG00000026029 | 4.384 | 6.324 | -0.436 | 0.004 | K04398 | Casp8 | caspase 8 |
| ENSMUSG00000026042 | 6.612 | 12.512 | -0.827 | 0.000 | K19721 | Col5a2 | collagen, type V, alpha 2 |
| ENSMUSG00000026043 | 30.025 | 93.084 | -1.541 | 0.000 | K19720 | Col3a1 | collagen, type III, alpha 1 |
| ENSMUSG00000026073 | 0.306 | 0.015 | 4.446 | 0.003 | K04387 | Il1r2 | interleukin 1 receptor, type II |
| ENSMUSG00000026074 | 30.091 | 42.684 | -0.411 | 0.000 | K04407 | Map4k4 | mitogen-activated protein kinase kinase kinase kinase 4 |
| ENSMUSG00000026104 | 2.788 | 4.698 | -0.659 | 0.002 | K11220 | Stat1 | signal transducer and activator of transcription 1 |
| ENSMUSG00000026177 | 2.637 | 4.118 | -0.549 | 0.014 | K12347 | Slc11a1 | solute carrier family 11 (proton-coupled divalent metal ion transporters), member 1 |
| ENSMUSG00000026180 | 0.325 | 0.115 | 1.605 | 0.040 | K05050 | Cxcr2 | chemokine (C-X-C motif) receptor 2 |
| ENSMUSG00000026204 | 5.083 | 2.600 | 1.062 | 0.000 | K07817 | Ptprn | protein tyrosine phosphatase, receptor type, N |
| ENSMUSG00000026288 | 3.226 | 4.852 | -0.496 | 0.020 | K03084 | Inpp5d | inositol polyphosphate-5-phosphatase D |
| ENSMUSG00000026303 | 0.582 | 0.266 | 1.226 | 0.013 | None | Mlph | melanophilin |
| ENSMUSG00000026380 | 0.410 | 0.099 | 2.146 | 0.000 | K09275 | Tfcp2l1 | transcription factor CP2-like 1 |
| ENSMUSG00000026389 | 6.922 | 9.998 | -0.437 | 0.000 | K10142 | Steap3 | STEAP family member 3 |
| ENSMUSG00000026398 | 0.136 | 0.326 | -1.163 | 0.030 | K08027 | Nr5a2 | nuclear receptor subfamily 5, group A, member 2 |
| ENSMUSG00000026399 | 2.622 | 4.082 | -0.546 | 0.009 | K04006 | Cd55 | CD55 molecule, decay accelerating factor for complement |
| ENSMUSG00000026407 | 3.739 | 2.673 | 0.577 | 0.000 | K04857 | Cacna1s | calcium channel, voltage-dependent, L type, alpha 1S subunit |
| ENSMUSG00000026429 | 0.661 | 1.126 | -0.677 | 0.037 | K13960 | Ube2t | ubiquitin-conjugating enzyme E2T |
| ENSMUSG00000026433 | 7.841 | 11.309 | -0.436 | 0.005 | K07916 | Rab29 | RAB29, member RAS oncogene family |
| ENSMUSG00000026471 | 5.724 | 4.357 | 0.487 | 0.001 | None | Mr1 | major histocompatibility complex, class I-related |
| ENSMUSG00000026473 | 297.978 | 228.645 | 0.477 | 0.000 | K01915 | Glul | glutamate-ammonia ligase (glutamine synthetase) |
| ENSMUSG00000026480 | 1.860 | 2.931 | -0.564 | 0.011 | K08010 | Ncf2 | neutrophil cytosolic factor 2 |
| ENSMUSG00000026547 | 51.552 | 72.786 | -0.406 | 0.000 | K20526 | Tagln2 | transgelin 2 |
| ENSMUSG00000026548 | 3.094 | 7.671 | -1.217 | 0.000 | None | Slamf9 | SLAM family member 9 |
| ENSMUSG00000026603 | 51.712 | 40.254 | 0.454 | 0.000 | K11426 | Smyd2 | SET and MYND domain containing 2 |
| ENSMUSG00000026655 | 2.237 | 3.271 | -0.455 | 0.032 | None | Fam107b | family with sequence similarity 107, member B |
| ENSMUSG00000026674 | 3.028 | 4.526 | -0.488 | 0.000 | K05125 | Ddr2 | discoidin domain receptor family, member 2 |
| ENSMUSG00000026675 | 3.846 | 3.035 | 0.434 | 0.005 | K13373 | Hsd17b7 | hydroxysteroid (17-beta) dehydrogenase 7 |
| ENSMUSG00000026687 | 23.465 | 18.434 | 0.441 | 0.000 | K00149 | Aldh9a1 | aldehyde dehydrogenase 9, subfamily A1 |
| ENSMUSG00000026688 | 448.781 | 345.719 | 0.470 | 0.000 | K00799 | Mgst3 | microsomal glutathione S-transferase 3 |
| ENSMUSG00000026707 | 1.534 | 0.967 | 0.759 | 0.009 | None | Nsun6 | NOL1/NOP2/Sun domain family member 6 |
| ENSMUSG00000026743 | 3.789 | 2.840 | 0.508 | 0.001 | None | Mllt10 | myeloid/lymphoid or mixed-lineage leukemia; translocated to, 10 |
| ENSMUSG00000026773 | 7.613 | 5.467 | 0.570 | 0.000 | K01103 | Pfkfb3 | 6-phosphofructo-2-kinase/fructose-2,6-biphosphatase 3 |
| ENSMUSG00000026799 | 6.328 | 8.985 | -0.412 | 0.024 | K15170 | Med27 | mediator complex subunit 27 |
| ENSMUSG00000026822 | 5.521 | 2.307 | 1.354 | 0.000 | None | Lcn2 | lipocalin 2 |
| ENSMUSG00000026837 | 5.912 | 10.243 | -0.701 | 0.000 | K19721 | Col5a1 | collagen, type V, alpha 1 |
| ENSMUSG00000026853 | 215.730 | 174.525 | 0.399 | 0.000 | K00624 | Crat | carnitine acetyltransferase |
| ENSMUSG00000026854 | 16.802 | 12.544 | 0.515 | 0.000 | K11848 | Usp20 | ubiquitin specific peptidase 20 |
| ENSMUSG00000026885 | 4.203 | 1.914 | 1.227 | 0.000 | K16604 | Ttll11 | tubulin tyrosine ligase-like family, member 11 |
| ENSMUSG00000026896 | 1.807 | 2.566 | -0.414 | 0.019 | K12647 | Ifih1 | interferon induced with helicase C domain 1 |
| ENSMUSG00000026971 | 0.982 | 2.916 | -1.478 | 0.000 | K06589 | Itgb6 | integrin beta 6 |
| ENSMUSG00000026979 | 0.143 | 0.377 | -1.306 | 0.001 | K12494 | Psd4 | pleckstrin and Sec7 domain containing 4 |
| ENSMUSG00000026991 | 58.427 | 46.277 | 0.431 | 0.000 | None | Pkp4 | plakophilin 4 |
| ENSMUSG00000027074 | 71.935 | 49.115 | 0.644 | 0.000 | K08230 | Slc43a3 | solute carrier family 43, member 3 |
| ENSMUSG00000027075 | 1.973 | 1.252 | 0.748 | 0.002 | K08228 | Slc43a1 | solute carrier family 43, member 1 |
| ENSMUSG00000027111 | 17.347 | 30.386 | -0.716 | 0.000 | K06485 | Itga6 | integrin alpha 6 |
| ENSMUSG00000027173 | 0.476 | 1.132 | -1.159 | 0.018 | None | Depdc7 | DEP domain containing 7 |
| ENSMUSG00000027187 | 164.125 | 132.025 | 0.407 | 0.000 | K03781 | Cat | catalase |
| ENSMUSG00000027204 | 11.254 | 17.896 | -0.576 | 0.000 | K06825 | Fbn1 | fibrillin 1 |
| ENSMUSG00000027217 | 6.531 | 11.426 | -0.714 | 0.000 | K17353 | Tspan18 | tetraspanin 18 |
| ENSMUSG00000027219 | 11.793 | 17.024 | -0.436 | 0.000 | K11536 | Slc28a2 | solute carrier family 28 (sodium-coupled nucleoside transporter), member 2 |
| ENSMUSG00000027227 | 192.475 | 143.575 | 0.515 | 0.000 | K00008 | Sord | sorbitol dehydrogenase |
| ENSMUSG00000027233 | 1.265 | 2.297 | -0.766 | 0.027 | None | Patl2 | protein associated with topoisomerase II homolog 2 (yeast) |
| ENSMUSG00000027253 | 2.984 | 2.343 | 0.442 | 0.013 | K20051 | Lrp4 | low density lipoprotein receptor-related protein 4 |
| ENSMUSG00000027274 | 2.475 | 3.820 | -0.534 | 0.012 | K09492 | Mkks | McKusick-Kaufman syndrome |
| ENSMUSG00000027293 | 199.737 | 159.801 | 0.415 | 0.000 | K12477 | Ehd4 | EH-domain containing 4 |
| ENSMUSG00000027322 | 2.057 | 3.092 | -0.494 | 0.009 | K06548 | Siglec1 | sialic acid binding Ig-like lectin 1, sialoadhesin |
| ENSMUSG00000027339 | 1.827 | 2.944 | -0.596 | 0.000 | K09851 | Rassf2 | Ras association (RalGDS/AF-6) domain family member 2 |
| ENSMUSG00000027347 | 0.177 | 0.427 | -1.170 | 0.047 | K04350 | Rasgrp1 | RAS guanyl releasing protein 1 |
| ENSMUSG00000027375 | 7.826 | 4.864 | 0.778 | 0.000 | None | Mal | myelin and lymphocyte protein, T cell differentiation protein |
| ENSMUSG00000027377 | 4.148 | 6.862 | -0.632 | 0.001 | None | Mall | mal, T cell differentiation protein-like |
| ENSMUSG00000027408 | 5.091 | 8.556 | -0.655 | 0.000 | K08638 | Cpxm1 | carboxypeptidase X 1 (M14 family) |
| ENSMUSG00000027435 | 23.061 | 33.829 | -0.460 | 0.000 | K06702 | Cd93 | CD93 antigen |
| ENSMUSG00000027469 | 0.316 | 0.695 | -1.042 | 0.003 | K16812 | Tpx2 | TPX2, microtubule-associated |
| ENSMUSG00000027488 | 65.719 | 53.919 | 0.379 | 0.002 | None | Snta1 | syntrophin, acidic 1 |
| ENSMUSG00000027500 | 1.975 | 2.960 | -0.489 | 0.026 | None | Stmn2 | stathmin-like 2 |
| ENSMUSG00000027509 | 21.838 | 17.564 | 0.408 | 0.002 | K14298 | Rae1 | ribonucleic acid export 1 |
| ENSMUSG00000027514 | 0.810 | 1.745 | -1.011 | 0.004 | K12965 | Zbp1 | Z-DNA binding protein 1 |
| ENSMUSG00000027523 | 7.361 | 4.592 | 0.778 | 0.001 | K04632 | Gnas | GNAS (guanine nucleotide binding protein, alpha stimulating) complex locus |
| ENSMUSG00000027524 | 0.505 | 0.244 | 1.146 | 0.027 | K05227 | Edn3 | endothelin 3 |
| ENSMUSG00000027525 | 0.163 | 0.050 | 1.814 | 0.040 | K17594 | Phactr3 | phosphatase and actin regulator 3 |
| ENSMUSG00000027547 | 0.610 | 0.366 | 0.829 | 0.015 | K19871 | Sall4 | spalt like transcription factor 4 |
| ENSMUSG00000027639 | 12.361 | 17.672 | -0.422 | 0.000 | None | Samhd1 | SAM domain and HD domain, 1 |
| ENSMUSG00000027669 | 3.836 | 5.362 | -0.390 | 0.002 | K04538 | Gnb4 | guanine nucleotide binding protein (G protein), beta 4 |
| ENSMUSG00000027750 | 5.934 | 10.090 | -0.674 | 0.000 | None | Postn | periostin, osteoblast specific factor |
| ENSMUSG00000027796 | 1.359 | 0.949 | 0.612 | 0.003 | K16791 | Smad9 | SMAD family member 9 |
| ENSMUSG00000027834 | 1.309 | 1.952 | -0.485 | 0.035 | None | Serpini1 | serine (or cysteine) peptidase inhibitor, clade I, member 1 |
| ENSMUSG00000027845 | 1.003 | 0.536 | 0.997 | 0.000 | K15341 | Dclre1b | DNA cross-link repair 1B |
| ENSMUSG00000027864 | 10.763 | 17.645 | -0.620 | 0.000 | K06729 | Ptgfrn | prostaglandin F2 receptor negative regulator |
| ENSMUSG00000027875 | 93.809 | 31.989 | 1.646 | 0.000 | K01641 | Hmgcs2 | 3-hydroxy-3-methylglutaryl-Coenzyme A synthase 2 |
| ENSMUSG00000027890 | 11.260 | 9.092 | 0.401 | 0.004 | K00799 | Gstm4 | glutathione S-transferase, mu 4 |
| ENSMUSG00000027907 | 58.854 | 83.313 | -0.410 | 0.010 | None | S100a11 | S100 calcium binding protein A11 |
| ENSMUSG00000027954 | 21.930 | 31.039 | -0.409 | 0.029 | K05462 | Efna1 | ephrin A1 |
| ENSMUSG00000027955 | 4.477 | 7.324 | -0.618 | 0.000 | None | Fam198b | family with sequence similarity 198, member B |
| ENSMUSG00000027956 | 14.018 | 10.840 | 0.465 | 0.000 | None | Tmem144 | transmembrane protein 144 |
| ENSMUSG00000027966 | 0.027 | 0.135 | -2.235 | 0.042 | K19721 | Col11a1 | collagen, type XI, alpha 1 |
| ENSMUSG00000027985 | 0.085 | 0.275 | -1.591 | 0.050 | K04492 | Lef1 | lymphoid enhancer binding factor 1 |
| ENSMUSG00000027994 | 1.871 | 3.516 | -0.813 | 0.014 | None | Mcub | mitochondrial calcium uniporter dominant negative beta subunit |
| ENSMUSG00000027995 | 1.870 | 3.087 | -0.629 | 0.011 | K10159 | Tlr2 | toll-like receptor 2 |
| ENSMUSG00000027996 | 3.919 | 5.860 | -0.488 | 0.042 | K02176 | Sfrp2 | secreted frizzled-related protein 2 |
| ENSMUSG00000027999 | 21.398 | 17.101 | 0.417 | 0.000 | K01047 | Pla2g12a | phospholipase A2, group XIIA |
| ENSMUSG00000028024 | 3.267 | 5.143 | -0.562 | 0.000 | K11141 | Enpep | glutamyl aminopeptidase |
| ENSMUSG00000028037 | 1.017 | 1.631 | -0.587 | 0.040 | None | Ifi44 | interferon-induced protein 44 |
| ENSMUSG00000028064 | 2.560 | 4.241 | -0.634 | 0.001 | K06521 | Sema4a | sema domain, immunoglobulin domain (Ig), transmembrane domain (TM)  and short cytoplasmic domain, (semaphorin) 4A |
| ENSMUSG00000028088 | 6.781 | 5.022 | 0.527 | 0.000 | K00485 | Fmo5 | flavin containing monooxygenase 5 |
| ENSMUSG00000028158 | 3.141 | 1.894 | 0.823 | 0.000 | K14463 | Mttp | microsomal triglyceride transfer protein |
| ENSMUSG00000028164 | 7.318 | 10.943 | -0.487 | 0.000 | K01192 | Manba | mannosidase, beta A, lysosomal |
| ENSMUSG00000028211 | 3.476 | 2.712 | 0.450 | 0.013 | K15310 | Trp53inp1 | transformation related protein 53 inducible nuclear protein 1 |
| ENSMUSG00000028223 | 193.507 | 135.000 | 0.612 | 0.000 | K13236 | Decr1 | 2,4-dienoyl CoA reductase 1, mitochondrial |
| ENSMUSG00000028268 | 3.811 | 8.386 | -1.044 | 0.000 | None | Gbp3 | guanylate binding protein 3 |
| ENSMUSG00000028270 | 8.636 | 16.396 | -0.833 | 0.000 | None | Gbp2 | guanylate binding protein 2 |
| ENSMUSG00000028329 | 11.456 | 9.237 | 0.403 | 0.022 | K10847 | Xpa | xeroderma pigmentosum, complementation group A |
| ENSMUSG00000028339 | 11.150 | 32.738 | -1.462 | 0.000 | K08135 | Col15a1 | collagen, type XV, alpha 1 |
| ENSMUSG00000028378 | 0.823 | 1.659 | -0.919 | 0.003 | K13948 | Ptgr1 | prostaglandin reductase 1 |
| ENSMUSG00000028383 | 116.555 | 95.501 | 0.380 | 0.000 | None | Hsdl2 | hydroxysteroid dehydrogenase like 2 |
| ENSMUSG00000028396 | 18.341 | 14.454 | 0.437 | 0.001 | None | 2310002L09Rik | RIKEN cDNA 2310002L09 gene |
| ENSMUSG00000028410 | 6.344 | 9.030 | -0.418 | 0.002 | K09502 | Dnaja1 | DnaJ heat shock protein family (Hsp40) member A1 |
| ENSMUSG00000028420 | 11.167 | 8.842 | 0.429 | 0.001 | None | Tmem38b | transmembrane protein 38B |
| ENSMUSG00000028444 | 4.931 | 3.458 | 0.605 | 0.020 | K05059 | Cntfr | ciliary neurotrophic factor receptor |
| ENSMUSG00000028480 | 2.765 | 4.504 | -0.610 | 0.014 | None | Glipr2 | GLI pathogenesis-related 2 |
| ENSMUSG00000028497 | 2.248 | 3.262 | -0.445 | 0.018 | K10703 | Hacd4 | 3-hydroxyacyl-CoA dehydratase 4 |
| ENSMUSG00000028581 | 12.247 | 20.991 | -0.685 | 0.000 | K12387 | Laptm5 | lysosomal-associated protein transmembrane 5 |
| ENSMUSG00000028599 | 5.372 | 8.283 | -0.532 | 0.001 | K05141 | Tnfrsf1b | tumor necrosis factor receptor superfamily, member 1b |
| ENSMUSG00000028630 | 3.415 | 5.376 | -0.562 | 0.000 | K18669 | Dyrk2 | dual-specificity tyrosine-(Y)-phosphorylation regulated kinase 2 |
| ENSMUSG00000028645 | 3.549 | 2.590 | 0.548 | 0.001 | K07299 | Slc2a1 | solute carrier family 2 (facilitated glucose transporter), member 1 |
| ENSMUSG00000028773 | 5732.348 | 4582.568 | 0.417 | 0.000 | K08752 | Fabp3 | fatty acid binding protein 3, muscle and heart |
| ENSMUSG00000028780 | 1.232 | 2.171 | -0.726 | 0.006 | K06840 | Sema3c | sema domain, immunoglobulin domain (Ig), short basic domain, secreted, (semaphorin) 3C |
| ENSMUSG00000028825 | 8.815 | 4.949 | 0.927 | 0.000 | K06579 | Rhd | Rh blood group, D antigen |
| ENSMUSG00000028841 | 7.789 | 5.231 | 0.670 | 0.009 | None | Cnksr1 | connector enhancer of kinase suppressor of Ras 1 |
| ENSMUSG00000028843 | 31.299 | 47.439 | -0.508 | 0.006 | None | Sh3bgrl3 | SH3 domain binding glutamic acid-rich protein-like 3 |
| ENSMUSG00000028862 | 10.279 | 4.671 | 1.232 | 0.000 | K04425 | Map3k6 | mitogen-activated protein kinase kinase kinase 6 |
| ENSMUSG00000028896 | 2.855 | 4.205 | -0.465 | 0.015 | K11493 | Rcc1 | regulator of chromosome condensation 1 |
| ENSMUSG00000028906 | 23.263 | 17.859 | 0.474 | 0.000 | K06107 | Epb41 | erythrocyte membrane protein band 4.1 |
| ENSMUSG00000028909 | 4.198 | 2.044 | 1.132 | 0.000 | K16662 | Ptpru | protein tyrosine phosphatase, receptor type, U |
| ENSMUSG00000028919 | 10.033 | 15.083 | -0.495 | 0.002 | None | Arhgef19 | Rho guanine nucleotide exchange factor (GEF) 19 |
| ENSMUSG00000028937 | 78.234 | 63.997 | 0.383 | 0.000 | K17360 | Acot7 | acyl-CoA thioesterase 7 |
| ENSMUSG00000028952 | 3.465 | 2.761 | 0.420 | 0.049 | K10519 | Zbtb48 | zinc finger and BTB domain containing 48 |
| ENSMUSG00000028986 | 17.535 | 14.361 | 0.381 | 0.000 | K10445 | Klhl7 | kelch-like 7 |
| ENSMUSG00000028988 | 11.089 | 15.945 | -0.430 | 0.000 | K04493 | Ctnnbip1 | catenin beta interacting protein 1 |
| ENSMUSG00000029001 | 13.586 | 10.824 | 0.421 | 0.002 | K10103 | Fbxo44 | F-box protein 44 |
| ENSMUSG00000029095 | 14.380 | 11.766 | 0.382 | 0.000 | K07520 | Ablim2 | actin-binding LIM protein 2 |
| ENSMUSG00000029120 | 0.464 | 1.272 | -1.364 | 0.001 | K04354 | Ppp2r2c | protein phosphatase 2, regulatory subunit B, gamma |
| ENSMUSG00000029123 | 1.520 | 0.816 | 0.989 | 0.000 | K08793 | Stk32b | serine/threonine kinase 32B |
| ENSMUSG00000029161 | 2.505 | 1.351 | 0.982 | 0.008 | None | Cgref1 | cell growth regulator with EF hand domain 1 |
| ENSMUSG00000029162 | 20.395 | 15.477 | 0.490 | 0.000 | K00846 | Khk | ketohexokinase |
| ENSMUSG00000029185 | 6.105 | 8.657 | -0.411 | 0.003 | None | Fam114a1 | family with sequence similarity 114, member A1 |
| ENSMUSG00000029202 | 2.674 | 3.762 | -0.401 | 0.021 | K11267 | Pds5a | PDS5 cohesin associated factor A |
| ENSMUSG00000029223 | 12.238 | 9.821 | 0.409 | 0.013 | K05611 | Uchl1 | ubiquitin carboxy-terminal hydrolase L1 |
| ENSMUSG00000029228 | 5.710 | 4.610 | 0.401 | 0.012 | K10692 | Lnx1 | ligand of numb-protein X 1 |
| ENSMUSG00000029311 | 49.745 | 32.323 | 0.714 | 0.000 | None | Hsd17b11 | hydroxysteroid (17-beta) dehydrogenase 11 |
| ENSMUSG00000029321 | 8.391 | 3.482 | 1.364 | 0.000 | K14346 | Slc10a6 | solute carrier family 10 (sodium/bile acid cotransporter family), member 6 |
| ENSMUSG00000029322 | 0.937 | 2.151 | -1.098 | 0.004 | None | Plac8 | placenta-specific 8 |
| ENSMUSG00000029330 | 2.424 | 1.467 | 0.819 | 0.000 | K00981 | Cds1 | CDP-diacylglycerol synthase 1 |
| ENSMUSG00000029343 | 0.489 | 2.002 | -1.936 | 0.001 | None | Crybb1 | crystallin, beta B1 |
| ENSMUSG00000029359 | 171.287 | 122.765 | 0.574 | 0.000 | K17612 | Tesc | tescalcin |
| ENSMUSG00000029368 | 0.148 | 5.461 | -5.098 | 0.021 | K16141 | Alb | albumin |
| ENSMUSG00000029417 | 1.294 | 4.515 | -1.708 | 0.000 | K05416 | Cxcl9 | chemokine (C-X-C motif) ligand 9 |
| ENSMUSG00000029426 | 66.836 | 53.664 | 0.410 | 0.000 | K12384 | Scarb2 | scavenger receptor class B, member 2 |
| ENSMUSG00000029469 | 7.791 | 11.416 | -0.460 | 0.013 | K19677 | Ift81 | intraflagellar transport 81 |
| ENSMUSG00000029521 | 1.828 | 3.069 | -0.654 | 0.019 | K06641 | Chek2 | checkpoint kinase 2 |
| ENSMUSG00000029544 | 3.204 | 4.519 | -0.404 | 0.011 | None | Cabp1 | calcium binding protein 1 |
| ENSMUSG00000029561 | 6.086 | 10.039 | -0.631 | 0.003 | K14608 | Oasl2 | 2'-5' oligoadenylate synthetase-like 2 |
| ENSMUSG00000029580 | 209.973 | 292.337 | -0.384 | 0.002 | K05692 | Actb | actin, beta |
| ENSMUSG00000029581 | 16.874 | 29.648 | -0.719 | 0.000 | K17455 | Fscn1 | fascin actin-bundling protein 1 |
| ENSMUSG00000029591 | 3.936 | 6.540 | -0.640 | 0.000 | K03648 | Ung | uracil DNA glycosylase |
| ENSMUSG00000029657 | 4.921 | 7.697 | -0.552 | 0.004 | K09485 | Hsph1 | heat shock 105kDa/110kDa protein 1 |
| ENSMUSG00000029661 | 32.232 | 60.213 | -0.811 | 0.000 | K06236 | Col1a2 | collagen, type I, alpha 2 |
| ENSMUSG00000029669 | 7.158 | 9.975 | -0.386 | 0.005 | K17355 | Tspan12 | tetraspanin 12 |
| ENSMUSG00000029673 | 0.135 | 0.372 | -1.378 | 0.024 | None | Auts2 | autism susceptibility candidate 2 |
| ENSMUSG00000029675 | 27.597 | 44.652 | -0.603 | 0.000 | K14211 | Eln | elastin |
| ENSMUSG00000029718 | 19.417 | 28.840 | -0.478 | 0.000 | None | Pcolce | procollagen C-endopeptidase enhancer protein |
| ENSMUSG00000029722 | 11.872 | 7.295 | 0.797 | 0.000 | None | Agfg2 | ArfGAP with FG repeats 2 |
| ENSMUSG00000029771 | 3.068 | 4.538 | -0.471 | 0.027 | K09446 | Irf5 | interferon regulatory factor 5 |
| ENSMUSG00000029802 | 7.971 | 6.063 | 0.488 | 0.001 | K05681 | Abcg2 | ATP binding cassette subfamily G member 2 (Junior blood group) |
| ENSMUSG00000029816 | 2.243 | 1.049 | 1.188 | 0.000 | None | Gpnmb | glycoprotein (transmembrane) nmb |
| ENSMUSG00000029838 | 1.842 | 2.796 | -0.507 | 0.037 | K16642 | Ptn | pleiotrophin |
| ENSMUSG00000029875 | 1.114 | 0.535 | 1.146 | 0.001 | None | Ccdc184 | coiled-coil domain containing 184 |
| ENSMUSG00000029925 | 0.877 | 1.403 | -0.584 | 0.022 | K01832 | Tbxas1 | thromboxane A synthase 1, platelet |
| ENSMUSG00000030022 | 2.709 | 4.608 | -0.673 | 0.000 | K08624 | Adamts9 | a disintegrin-like and metallopeptidase (reprolysin type) with thrombospondin type 1 motif, 9 |
| ENSMUSG00000030036 | 4.017 | 5.867 | -0.453 | 0.013 | K01228 | Mogs | mannosyl-oligosaccharide glucosidase |
| ENSMUSG00000030087 | 48.625 | 36.737 | 0.498 | 0.000 | K09210 | Klf15 | Kruppel-like factor 15 |
| ENSMUSG00000030096 | 42.702 | 32.433 | 0.490 | 0.000 | K05039 | Slc6a6 | solute carrier family 6 (neurotransmitter transporter, taurine), member 6 |
| ENSMUSG00000030110 | 0.739 | 1.737 | -1.139 | 0.000 | K05126 | Ret | ret proto-oncogene |
| ENSMUSG00000030111 | 1.196 | 0.743 | 0.779 | 0.003 | K03910 | A2m | alpha-2-macroglobulin |
| ENSMUSG00000030123 | 26.034 | 41.354 | -0.574 | 0.000 | K06822 | Plxnd1 | plexin D1 |
| ENSMUSG00000030134 | 0.474 | 0.130 | 1.951 | 0.003 | None | Rasgef1a | RasGEF domain family, member 1A |
| ENSMUSG00000030142 | 0.084 | 0.007 | 3.734 | 0.004 | K10059 | Clec4e | C-type lectin domain family 4, member e |
| ENSMUSG00000030170 | 4.272 | 6.202 | -0.445 | 0.014 | K00444 | Wnt5b | wingless-type MMTV integration site family, member 5B |
| ENSMUSG00000030208 | 18.590 | 28.859 | -0.541 | 0.000 | None | Emp1 | epithelial membrane protein 1 |
| ENSMUSG00000030245 | 4.024 | 5.978 | -0.479 | 0.000 | None | Golt1b | golgi transport 1B |
| ENSMUSG00000030256 | 2.174 | 1.301 | 0.834 | 0.000 | K03730 | Bhlhe41 | basic helix-loop-helix family, member e41 |
| ENSMUSG00000030263 | 1.239 | 2.529 | -0.934 | 0.004 | None | Lrmp | lymphoid-restricted membrane protein |
| ENSMUSG00000030306 | 37.779 | 28.492 | 0.500 | 0.000 | None | Tmtc1 | transmembrane and tetratricopeptide repeat containing 1 |
| ENSMUSG00000030315 | 12.637 | 18.954 | -0.492 | 0.000 | None | Vgll4 | vestigial like family member 4 |
| ENSMUSG00000030323 | 3.047 | 5.736 | -0.819 | 0.000 | K19656 | Ift122 | intraflagellar transport 122 |
| ENSMUSG00000030346 | 0.223 | 0.629 | -1.398 | 0.046 | None | Rad51ap1 | RAD51 associated protein 1 |
| ENSMUSG00000030474 | 0.874 | 1.713 | -0.875 | 0.044 | None | Siglece | sialic acid binding Ig-like lectin E |
| ENSMUSG00000030483 | 0.970 | 0.241 | 2.105 | 0.001 | K07412 | Cyp2b10 | cytochrome P450, family 2, subfamily b, polypeptide 10 |
| ENSMUSG00000030522 | 2.128 | 1.708 | 0.410 | 0.044 | K18085 | Mtmr10 | myotubularin related protein 10 |
| ENSMUSG00000030541 | 1834.039 | 1469.574 | 0.413 | 0.000 | K00031 | Idh2 | isocitrate dehydrogenase 2 (NADP+), mitochondrial |
| ENSMUSG00000030560 | 11.014 | 17.229 | -0.553 | 0.000 | K01275 | Ctsc | cathepsin C |
| ENSMUSG00000030579 | 20.768 | 37.453 | -0.758 | 0.000 | K07992 | Tyrobp | TYRO protein tyrosine kinase binding protein |
| ENSMUSG00000030650 | 0.111 | 0.329 | -1.471 | 0.029 | None | Tmc5 | transmembrane channel-like gene family 5 |
| ENSMUSG00000030683 | 0.942 | 2.363 | -1.234 | 0.000 | None | Sez6l2 | seizure related 6 homolog like 2 |
| ENSMUSG00000030688 | 21.483 | 30.400 | -0.406 | 0.009 | None | Stard10 | START domain containing 10 |
| ENSMUSG00000030707 | 8.368 | 17.204 | -0.953 | 0.033 | K13882 | Coro1a | coronin, actin binding protein 1A |
| ENSMUSG00000030711 | 65.780 | 38.016 | 0.884 | 0.000 | K01014 | Sult1a1 | sulfotransferase family 1A, phenol-preferring, member 1 |
| ENSMUSG00000030737 | 10.601 | 8.552 | 0.403 | 0.000 | K14352 | Slco2b1 | solute carrier organic anion transporter family, member 2b1 |
| ENSMUSG00000030742 | 0.259 | 0.810 | -1.543 | 0.021 | K07362 | Lat | linker for activation of T cells |
| ENSMUSG00000030745 | 0.384 | 1.173 | -1.511 | 0.001 | K05075 | Il21r | interleukin 21 receptor |
| ENSMUSG00000030787 | 11.918 | 6.802 | 0.901 | 0.000 | K19012 | Lyve1 | lymphatic vessel endothelial hyaluronan receptor 1 |
| ENSMUSG00000030789 | 0.297 | 0.617 | -0.955 | 0.030 | K06462 | Itgax | integrin alpha X |
| ENSMUSG00000030795 | 42.238 | 34.521 | 0.383 | 0.000 | K13098 | Fus | fused in sarcoma |
| ENSMUSG00000030798 | 2.130 | 3.866 | -0.767 | 0.029 | K06475 | Cd37 | CD37 antigen |
| ENSMUSG00000030811 | 1.967 | 3.017 | -0.524 | 0.005 | K10285 | Fbxl19 | F-box and leucine-rich repeat protein 19 |
| ENSMUSG00000030844 | 7.061 | 13.658 | -0.859 | 0.001 | K16449 | Rgs10 | regulator of G-protein signalling 10 |
| ENSMUSG00000030878 | 3.612 | 5.796 | -0.589 | 0.000 | None | Cdr2 | cerebellar degeneration-related 2 |
| ENSMUSG00000030889 | 6.744 | 4.751 | 0.599 | 0.001 | None | Vwa3a | von Willebrand factor A domain containing 3A |
| ENSMUSG00000030921 | 2.855 | 4.256 | -0.483 | 0.012 | None | Trim30a | tripartite motif-containing 30A |
| ENSMUSG00000030972 | 4.238 | 2.701 | 0.743 | 0.004 | K01896 | Acsm5 | acyl-CoA synthetase medium-chain family member 5 |
| ENSMUSG00000030990 | 11.546 | 17.940 | -0.542 | 0.000 | None | Pgap2 | post-GPI attachment to proteins 2 |
| ENSMUSG00000031026 | 0.152 | 0.070 | 1.215 | 0.020 | K12032 | Trim66 | tripartite motif-containing 66 |
| ENSMUSG00000031101 | 1.119 | 2.272 | -0.927 | 0.003 | None | Sash3 | SAM and SH3 domain containing 3 |
| ENSMUSG00000031103 | 3.145 | 4.472 | -0.415 | 0.007 | K09428 | Elf4 | E74-like factor 4 (ets domain transcription factor) |
| ENSMUSG00000031111 | 3.618 | 5.640 | -0.547 | 0.002 | None | Igsf1 | immunoglobulin superfamily, member 1 |
| ENSMUSG00000031134 | 10.200 | 8.348 | 0.381 | 0.006 | K12885 | Rbmx | RNA binding motif protein, X chromosome |
| ENSMUSG00000031147 | 12.458 | 17.284 | -0.380 | 0.002 | None | Magix | MAGI family member, X-linked |
| ENSMUSG00000031155 | 2.455 | 1.774 | 0.561 | 0.040 | K08806 | Pim2 | proviral integration site 2 |
| ENSMUSG00000031161 | 5.760 | 4.529 | 0.441 | 0.002 | K11407 | Hdac6 | histone deacetylase 6 |
| ENSMUSG00000031167 | 27.871 | 21.103 | 0.493 | 0.001 | K13186 | Rbm3 | RNA binding motif (RNP1, RRM) protein 3 |
| ENSMUSG00000031239 | 9.893 | 14.188 | -0.428 | 0.006 | K18241 | Itm2a | integral membrane protein 2A |
| ENSMUSG00000031264 | 0.584 | 1.465 | -1.233 | 0.030 | K07370 | Btk | Bruton agammaglobulinemia tyrosine kinase |
| ENSMUSG00000031274 | 5.064 | 7.387 | -0.452 | 0.000 | K06237 | Col4a5 | collagen, type IV, alpha 5 |
| ENSMUSG00000031340 | 0.618 | 0.307 | 1.102 | 0.007 | K05185 | Gabre | gamma-aminobutyric acid (GABA) A receptor, subunit epsilon |
| ENSMUSG00000031343 | 1.882 | 1.126 | 0.833 | 0.002 | K05175 | Gabra3 | gamma-aminobutyric acid (GABA) A receptor, subunit alpha 3 |
| ENSMUSG00000031389 | 0.259 | 0.529 | -0.931 | 0.037 | K20122 | Arhgap4 | Rho GTPase activating protein 4 |
| ENSMUSG00000031398 | 0.111 | 0.330 | -1.478 | 0.001 | K06820 | Plxna3 | plexin A3 |
| ENSMUSG00000031461 | 775.171 | 537.608 | 0.623 | 0.000 | None | Myom2 | myomesin 2 |
| ENSMUSG00000031490 | 112.653 | 78.753 | 0.610 | 0.000 | K07205 | Eif4ebp1 | eukaryotic translation initiation factor 4E binding protein 1 |
| ENSMUSG00000031502 | 59.556 | 117.976 | -0.892 | 0.000 | K06237 | Col4a1 | collagen, type IV, alpha 1 |
| ENSMUSG00000031503 | 58.974 | 100.150 | -0.670 | 0.000 | K06237 | Col4a2 | collagen, type IV, alpha 2 |
| ENSMUSG00000031506 | 0.396 | 0.778 | -0.881 | 0.041 | K18019 | Ptpn7 | protein tyrosine phosphatase, non-receptor type 7 |
| ENSMUSG00000031520 | 4.965 | 6.956 | -0.394 | 0.047 | K05449 | Vegfc | vascular endothelial growth factor C |
| ENSMUSG00000031530 | 15.505 | 2.343 | 2.819 | 0.000 | K04459 | Dusp4 | dual specificity phosphatase 4 |
| ENSMUSG00000031543 | 2.432 | 1.948 | 0.414 | 0.017 | K10380 | Ank1 | ankyrin 1, erythroid |
| ENSMUSG00000031558 | 1.256 | 0.832 | 0.685 | 0.000 | K06839 | Slit2 | slit guidance ligand 2 |
| ENSMUSG00000031586 | 112.892 | 86.755 | 0.473 | 0.000 | None | Rbpms | RNA binding protein gene with multiple splicing |
| ENSMUSG00000031628 | 1.172 | 2.161 | -0.786 | 0.011 | K02187 | Casp3 | caspase 3 |
| ENSMUSG00000031659 | 2.528 | 4.619 | -0.776 | 0.000 | K08047 | Adcy7 | adenylate cyclase 7 |
| ENSMUSG00000031709 | 1.214 | 2.022 | -0.643 | 0.003 | K19951 | Tbc1d9 | TBC1 domain family, member 9 |
| ENSMUSG00000031734 | 19.292 | 10.332 | 0.994 | 0.000 | None | Irx3 | Iroquois related homeobox 3 |
| ENSMUSG00000031736 | 6.011 | 3.879 | 0.726 | 0.001 |  | Crnde | colorectal neoplasia differentially expressed (non-protein coding) |
| ENSMUSG00000031737 | 6.057 | 4.614 | 0.486 | 0.003 | None | Irx5 | Iroquois homeobox 5 |
| ENSMUSG00000031740 | 37.199 | 56.712 | -0.516 | 0.000 | K01398 | Mmp2 | matrix metallopeptidase 2 |
| ENSMUSG00000031755 | 7.957 | 6.294 | 0.431 | 0.002 | K16747 | Bbs2 | Bardet-Biedl syndrome 2 (human) |
| ENSMUSG00000031756 | 0.200 | 0.522 | -1.296 | 0.027 | K11506 | Cenpn | centromere protein N |
| ENSMUSG00000031762 | 3.723 | 1.412 | 1.490 | 0.001 | K14739 | Mt2 | metallothionein 2 |
| ENSMUSG00000031765 | 233.482 | 129.973 | 0.938 | 0.000 | K14739 | Mt1 | metallothionein 1 |
| ENSMUSG00000031770 | 138.124 | 100.738 | 0.550 | 0.000 | K14027 | Herpud1 | homocysteine-inducible, endoplasmic reticulum stress-inducible, ubiquitin-like domain member 1 |
| ENSMUSG00000031785 | 45.736 | 32.631 | 0.582 | 0.000 | K08450 | Adgrg1 | adhesion G protein-coupled receptor G1 |
| ENSMUSG00000031790 | 20.181 | 34.269 | -0.670 | 0.000 | K07995 | Mmp15 | matrix metallopeptidase 15 |
| ENSMUSG00000031799 | 9.372 | 13.733 | -0.459 | 0.000 | K10375 | Tpm4 | tropomyosin 4 |
| ENSMUSG00000031808 | 154.676 | 101.266 | 0.705 | 0.000 | K08745 | Slc27a1 | solute carrier family 27 (fatty acid transporter), member 1 |
| ENSMUSG00000031827 | 9.598 | 15.639 | -0.612 | 0.012 | None | Cotl1 | coactosin-like 1 (Dictyostelium) |
| ENSMUSG00000031840 | 1.149 | 0.083 | 3.902 | 0.000 | K07882 | Rab3a | RAB3A, member RAS oncogene family |
| ENSMUSG00000031875 | 13.806 | 22.592 | -0.617 | 0.000 | None | Cmtm3 | CKLF-like MARVEL transmembrane domain containing 3 |
| ENSMUSG00000031903 | 6.213 | 8.620 | -0.379 | 0.029 | K06129 | Pla2g15 | phospholipase A2, group XV |
| ENSMUSG00000031910 | 0.469 | 0.918 | -0.875 | 0.010 | K00752 | Has3 | hyaluronan synthase 3 |
| ENSMUSG00000031980 | 5.359 | 2.834 | 1.011 | 0.000 | K09821 | Agt | angiotensinogen (serpin peptidase inhibitor, clade A, member 8) |
| ENSMUSG00000032011 | 5.789 | 9.726 | -0.656 | 0.000 | K06514 | Thy1 | thymus cell antigen 1, theta |
| ENSMUSG00000032012 | 0.739 | 1.270 | -0.689 | 0.005 | K06081 | Nectin1 | nectin cell adhesion molecule 1 |
| ENSMUSG00000032035 | 17.891 | 29.222 | -0.615 | 0.000 | K02678 | Ets1 | E26 avian leukemia oncogene 1, 5' domain |
| ENSMUSG00000032089 | 2.552 | 4.605 | -0.758 | 0.000 | K05134 | Il10ra | interleukin 10 receptor, alpha |
| ENSMUSG00000032115 | 8.115 | 11.341 | -0.390 | 0.001 | K09486 | Hyou1 | hypoxia up-regulated 1 |
| ENSMUSG00000032122 | 0.966 | 1.471 | -0.515 | 0.016 | K13783 | Slc37a2 | solute carrier family 37 (glycerol-3-phosphate transporter), member 2 |
| ENSMUSG00000032172 | 0.676 | 0.274 | 1.389 | 0.013 | None | Olfm2 | olfactomedin 2 |
| ENSMUSG00000032202 | 2.926 | 2.302 | 0.439 | 0.036 | K07885 | Rab27a | RAB27A, member RAS oncogene family |
| ENSMUSG00000032220 | 3.328 | 5.446 | -0.618 | 0.000 | K10356 | Myo1e | myosin IE |
| ENSMUSG00000032224 | 1.508 | 2.997 | -0.899 | 0.000 | None | Fam81a | family with sequence similarity 81, member A |
| ENSMUSG00000032231 | 36.597 | 53.000 | -0.442 | 0.000 | K17092 | Anxa2 | annexin A2 |
| ENSMUSG00000032265 | 3.732 | 2.844 | 0.483 | 0.006 | None | Fam46a | family with sequence similarity 46, member A |
| ENSMUSG00000032271 | 2.687 | 4.224 | -0.558 | 0.044 | K00541 | Nnmt | nicotinamide N-methyltransferase |
| ENSMUSG00000032315 | 9.551 | 1.246 | 3.034 | 0.000 | K07408 | Cyp1a1 | cytochrome P450, family 1, subfamily a, polypeptide 1 |
| ENSMUSG00000032334 | 18.395 | 28.925 | -0.560 | 0.000 | K14678 | Loxl1 | lysyl oxidase-like 1 |
| ENSMUSG00000032350 | 6.161 | 5.014 | 0.389 | 0.006 | K11204 | Gclc | glutamate-cysteine ligase, catalytic subunit |
| ENSMUSG00000032372 | 18.199 | 14.490 | 0.421 | 0.028 | None | Plscr2 | phospholipid scramblase 2 |
| ENSMUSG00000032374 | 4.289 | 6.250 | -0.451 | 0.003 | K13645 | Plod2 | procollagen lysine, 2-oxoglutarate 5-dioxygenase 2 |
| ENSMUSG00000032411 | 19.091 | 11.679 | 0.801 | 0.000 | K09392 | Tfdp2 | transcription factor Dp 2 |
| ENSMUSG00000032420 | 8.300 | 6.074 | 0.544 | 0.000 | K19970 | Nt5e | 5' nucleotidase, ecto |
| ENSMUSG00000032456 | 15.950 | 13.019 | 0.385 | 0.001 | K06210 | Nmnat3 | nicotinamide nucleotide adenylyltransferase 3 |
| ENSMUSG00000032475 | 5.009 | 6.969 | -0.384 | 0.006 | K07365 | Nck1 | non-catalytic region of tyrosine kinase adaptor protein 1 |
| ENSMUSG00000032492 | 16.764 | 5.923 | 1.595 | 0.000 | K04585 | Pth1r | parathyroid hormone 1 receptor |
| ENSMUSG00000032511 | 47.466 | 33.856 | 0.582 | 0.000 | K04838 | Scn5a | sodium channel, voltage-gated, type V, alpha |
| ENSMUSG00000032565 | 6.057 | 4.625 | 0.483 | 0.007 | K16855 | Nudt16 | nudix (nucleoside diphosphate linked moiety X)-type motif 16 |
| ENSMUSG00000032596 | 6.036 | 9.673 | -0.587 | 0.000 | K10698 | Uba7 | ubiquitin-like modifier activating enzyme 7 |
| ENSMUSG00000032602 | 117.300 | 86.636 | 0.531 | 0.000 | K15109 | Slc25a20 | solute carrier family 25 (mitochondrial carnitine/acylcarnitine translocase), member 20 |
| ENSMUSG00000032625 | 1.957 | 1.184 | 0.817 | 0.000 | None | Thsd7a | thrombospondin, type I, domain containing 7A |
| ENSMUSG00000032649 | 3.216 | 4.535 | -0.404 | 0.008 | K11703 | Colgalt2 | collagen beta(1-O)galactosyltransferase 2 |
| ENSMUSG00000032690 | 3.535 | 5.219 | -0.469 | 0.004 | K14216 | Oas2 | 2'-5' oligoadenylate synthetase 2 |
| ENSMUSG00000032705 | 3.449 | 5.129 | -0.480 | 0.001 | None | Exd2 | exonuclease 3'-5' domain containing 2 |
| ENSMUSG00000032712 | 2.053 | 1.433 | 0.610 | 0.027 | None | 2810474O19Rik | RIKEN cDNA 2810474O19 gene |
| ENSMUSG00000032763 | 16.615 | 12.905 | 0.458 | 0.000 | K11259 | Ilvbl | ilvB (bacterial acetolactate synthase)-like |
| ENSMUSG00000032766 | 31.944 | 53.661 | -0.655 | 0.000 | K04546 | Gng11 | guanine nucleotide binding protein (G protein), gamma 11 |
| ENSMUSG00000032860 | 17.276 | 14.047 | 0.392 | 0.003 | K04269 | P2ry2 | purinergic receptor P2Y, G-protein coupled 2 |
| ENSMUSG00000032898 | 32.517 | 25.115 | 0.466 | 0.000 | K10301 | Fbxo21 | F-box protein 21 |
| ENSMUSG00000032915 | 0.097 | 0.425 | -2.012 | 0.000 | K08445 | Adgre4 | adhesion G protein-coupled receptor E4 |
| ENSMUSG00000032942 | 45.786 | 74.462 | -0.608 | 0.000 | K15103 | Ucp3 | uncoupling protein 3 (mitochondrial, proton carrier) |
| ENSMUSG00000033007 | 0.468 | 0.132 | 1.914 | 0.002 | K04831 | Asic4 | acid-sensing (proton-gated) ion channel family member 4 |
| ENSMUSG00000033024 | 0.276 | 1.244 | -2.070 | 0.000 | None | Klra9 | killer cell lectin-like receptor subfamily A, member 9 |
| ENSMUSG00000033033 | 2.174 | 3.335 | -0.524 | 0.032 | K19739 | Calhm2 | calcium homeostasis modulator 2 |
| ENSMUSG00000033066 | 0.246 | 0.426 | -0.695 | 0.046 | K18618 | Gas7 | growth arrest specific 7 |
| ENSMUSG00000033082 | 2.537 | 1.871 | 0.531 | 0.003 | K10069 | Clec1a | C-type lectin domain family 1, member a |
| ENSMUSG00000033083 | 15.681 | 22.302 | -0.415 | 0.002 | K17902 | Tbc1d4 | TBC1 domain family, member 4 |
| ENSMUSG00000033161 | 302.262 | 245.022 | 0.396 | 0.000 | K01539 | Atp1a1 | ATPase, Na+/K+ transporting, alpha 1 polypeptide |
| ENSMUSG00000033220 | 3.230 | 5.160 | -0.584 | 0.034 | K07860 | Rac2 | RAS-related C3 botulinum substrate 2 |
| ENSMUSG00000033249 | 5.126 | 3.364 | 0.699 | 0.001 | K09417 | Hsf4 | heat shock transcription factor 4 |
| ENSMUSG00000033287 | 15.392 | 25.516 | -0.636 | 0.000 | None | Kctd17 | potassium channel tetramerisation domain containing 17 |
| ENSMUSG00000033307 | 200.245 | 154.972 | 0.464 | 0.000 | K07253 | Mif | macrophage migration inhibitory factor (glycosylation-inhibiting factor) |
| ENSMUSG00000033308 | 3.565 | 2.848 | 0.416 | 0.012 | K00207 | Dpyd | dihydropyrimidine dehydrogenase |
| ENSMUSG00000033318 | 7.801 | 6.268 | 0.408 | 0.035 | K00799 | Gstt2 | glutathione S-transferase, theta 2 |
| ENSMUSG00000033355 | 5.531 | 10.137 | -0.781 | 0.000 | None | Rtp4 | receptor transporter protein 4 |
| ENSMUSG00000033361 | 3.073 | 4.319 | -0.399 | 0.001 | None | Prrg3 | proline rich Gla (G-carboxyglutamic acid) 3 (transmembrane) |
| ENSMUSG00000033508 | 0.235 | 0.034 | 2.864 | 0.015 | None | Asprv1 | aspartic peptidase, retroviral-like 1 |
| ENSMUSG00000033578 | 1.402 | 2.914 | -0.960 | 0.000 | None | Tmem35a | transmembrane protein 35A |
| ENSMUSG00000033684 | 21.771 | 17.401 | 0.417 | 0.000 | K10758 | Qsox1 | quiescin Q6 sulfhydryl oxidase 1 |
| ENSMUSG00000033793 | 6.189 | 5.033 | 0.390 | 0.003 | K02144 | Atp6v1h | ATPase, H+ transporting, lysosomal V1 subunit H |
| ENSMUSG00000033826 | 0.017 | 0.070 | -1.918 | 0.017 | K10408 | Dnah8 | dynein, axonemal, heavy chain 8 |
| ENSMUSG00000033863 | 38.451 | 28.625 | 0.516 | 0.001 | K09208 | Klf9 | Kruppel-like factor 9 |
| ENSMUSG00000033871 | 8.179 | 6.289 | 0.472 | 0.000 | K17962 | Ppargc1b | peroxisome proliferative activated receptor, gamma, coactivator 1 beta |
| ENSMUSG00000034009 | 0.070 | 0.294 | -1.971 | 0.001 | K04306 | Rxfp1 | relaxin/insulin-like family peptide receptor 1 |
| ENSMUSG00000034158 | 5.901 | 4.493 | 0.485 | 0.000 | None | Lrrc58 | leucine rich repeat containing 58 |
| ENSMUSG00000034173 | 3.093 | 2.342 | 0.493 | 0.050 | None | Zbed5 | zinc finger, BED type containing 5 |
| ENSMUSG00000034201 | 6.060 | 8.710 | -0.431 | 0.002 | None | Gas2l1 | growth arrest-specific 2 like 1 |
| ENSMUSG00000034205 | 2.292 | 4.264 | -0.802 | 0.000 | K00280 | Loxl2 | lysyl oxidase-like 2 |
| ENSMUSG00000034206 | 0.439 | 0.744 | -0.670 | 0.032 | K02349 | Polq | polymerase (DNA directed), theta |
| ENSMUSG00000034245 | 14.622 | 11.538 | 0.436 | 0.001 | K11418 | Hdac11 | histone deacetylase 11 |
| ENSMUSG00000034258 | 3.997 | 2.751 | 0.632 | 0.000 | K08220 | Flvcr2 | feline leukemia virus subgroup C cellular receptor 2 |
| ENSMUSG00000034297 | 0.837 | 0.624 | 0.516 | 0.017 | K15164 | Med13 | mediator complex subunit 13 |
| ENSMUSG00000034377 | 7.823 | 6.402 | 0.383 | 0.001 | None | Tulp4 | tubby like protein 4 |
| ENSMUSG00000034422 | 5.037 | 8.505 | -0.663 | 0.000 | K15261 | Parp14 | poly (ADP-ribose) polymerase family, member 14 |
| ENSMUSG00000034459 | 2.982 | 4.420 | -0.474 | 0.016 | K14217 | Ifit1 | interferon-induced protein with tetratricopeptide repeats 1 |
| ENSMUSG00000034472 | 7.410 | 2.947 | 1.424 | 0.000 | K07844 | Rasd2 | RASD family, member 2 |
| ENSMUSG00000034480 | 2.352 | 1.870 | 0.423 | 0.010 | K05741 | Diaph2 | diaphanous related formin 2 |
| ENSMUSG00000034570 | 3.329 | 4.708 | -0.407 | 0.033 | K01106 | Inpp5j | inositol polyphosphate 5-phosphatase J |
| ENSMUSG00000034574 | 10.867 | 15.619 | -0.430 | 0.000 | K04512 | Daam1 | dishevelled associated activator of morphogenesis 1 |
| ENSMUSG00000034623 | 0.148 | 0.953 | -2.615 | 0.004 | None | Prss55 | protease, serine 55 |
| ENSMUSG00000034648 | 0.081 | 0.371 | -2.089 | 0.013 | None | Lrrn1 | leucine rich repeat protein 1, neuronal |
| ENSMUSG00000034675 | 3.769 | 5.707 | -0.505 | 0.007 | None | Dbn1 | drebrin 1 |
| ENSMUSG00000034687 | 0.099 | 0.301 | -1.511 | 0.002 | None | Fras1 | Fraser extracellular matrix complex subunit 1 |
| ENSMUSG00000034777 | 1.700 | 0.911 | 0.992 | 0.024 | K09318 | Vax2 | ventral anterior homeobox 2 |
| ENSMUSG00000034842 | 286.144 | 218.834 | 0.481 | 0.000 | K00775 | Art3 | ADP-ribosyltransferase 3 |
| ENSMUSG00000034853 | 2.189 | 1.658 | 0.495 | 0.034 | K12417 | Acot11 | acyl-CoA thioesterase 11 |
| ENSMUSG00000034858 | 14.883 | 12.175 | 0.384 | 0.012 | None | Fam214a | family with sequence similarity 214, member A |
| ENSMUSG00000034881 | 1.826 | 3.142 | -0.689 | 0.019 | K04264 | Tbxa2r | thromboxane A2 receptor |
| ENSMUSG00000035064 | 16.367 | 10.329 | 0.758 | 0.000 | K08292 | Eef2k | eukaryotic elongation factor-2 kinase |
| ENSMUSG00000035121 | 1.288 | 0.764 | 0.847 | 0.007 | K10568 | Neil2 | nei like 2 (E. coli) |
| ENSMUSG00000035184 | 4.825 | 3.702 | 0.475 | 0.015 | None | Fam124a | family with sequence similarity 124, member A |
| ENSMUSG00000035279 | 1.764 | 2.689 | -0.514 | 0.038 | None | Ssc5d | scavenger receptor cysteine rich family, 5 domains |
| ENSMUSG00000035372 | 3.378 | 5.021 | -0.479 | 0.021 | None | 1810055G02Rik | RIKEN cDNA 1810055G02 gene |
| ENSMUSG00000035441 | 5.084 | 7.205 | -0.410 | 0.006 | K10356 | Myo1d | myosin ID |
| ENSMUSG00000035566 | 0.512 | 0.994 | -0.865 | 0.000 | K16499 | Pcdh17 | protocadherin 17 |
| ENSMUSG00000035606 | 2.507 | 3.866 | -0.533 | 0.000 | None | Ky | kyphoscoliosis peptidase |
| ENSMUSG00000035621 | 35.798 | 25.499 | 0.583 | 0.000 | None | Midn | midnolin |
| ENSMUSG00000035692 | 9.942 | 17.259 | -0.702 | 0.010 | K12159 | Isg15 | ISG15 ubiquitin-like modifier |
| ENSMUSG00000035697 | 3.795 | 6.416 | -0.664 | 0.000 | None | Arhgap45 | Rho GTPase activating protein 45 |
| ENSMUSG00000035722 | 5.378 | 4.317 | 0.410 | 0.000 | K05645 | Abca7 | ATP-binding cassette, sub-family A (ABC1), member 7 |
| ENSMUSG00000035824 | 11.375 | 8.644 | 0.489 | 0.000 | K00857 | Tk2 | thymidine kinase 2, mitochondrial |
| ENSMUSG00000035828 | 98.155 | 72.189 | 0.536 | 0.000 | K08807 | Pim3 | proviral integration site 3 |
| ENSMUSG00000035849 | 3.283 | 7.365 | -1.074 | 0.000 | None | Krt222 | keratin 222 |
| ENSMUSG00000035860 | 0.538 | 0.236 | 1.281 | 0.029 | K16503 | Cdhr3 | cadherin-related family member 3 |
| ENSMUSG00000035873 | 3.079 | 1.536 | 1.096 | 0.000 | None | Pawr | PRKC, apoptosis, WT1, regulator |
| ENSMUSG00000035896 | 1.525 | 3.000 | -0.883 | 0.025 | K01168 | Rnase1 | ribonuclease, RNase A family, 1 (pancreatic) |
| ENSMUSG00000036006 | 4.195 | 3.271 | 0.452 | 0.000 | None | Ripor2 | RHO family interacting cell polarization regulator 2 |
| ENSMUSG00000036022 | 2.015 | 1.181 | 0.863 | 0.000 | None | Fam122b | family with sequence similarity 122, member B |
| ENSMUSG00000036144 | 4.532 | 6.402 | -0.406 | 0.039 | K09322 | Meox2 | mesenchyme homeobox 2 |
| ENSMUSG00000036181 | 286.445 | 148.389 | 1.041 | 0.000 | K11275 | Hist1h1c | histone cluster 1, H1c |
| ENSMUSG00000036246 | 1.637 | 2.436 | -0.480 | 0.018 | None | Gmip | Gem-interacting protein |
| ENSMUSG00000036264 | 1.703 | 0.572 | 1.668 | 0.000 | None | Fstl4 | follistatin-like 4 |
| ENSMUSG00000036306 | 1.604 | 1.065 | 0.686 | 0.020 | None | Lzts1 | leucine zipper, putative tumor suppressor 1 |
| ENSMUSG00000036334 | 0.484 | 0.810 | -0.650 | 0.002 | None | Igsf10 | immunoglobulin superfamily, member 10 |
| ENSMUSG00000036390 | 3.201 | 5.899 | -0.790 | 0.003 | K04402 | Gadd45a | growth arrest and DNA-damage-inducible 45 alpha |
| ENSMUSG00000036412 | 0.325 | 1.501 | -2.113 | 0.000 | K12375 | Arsi | arylsulfatase i |
| ENSMUSG00000036446 | 27.488 | 41.058 | -0.487 | 0.000 | K08122 | Lum | lumican |
| ENSMUSG00000036492 | 4.910 | 3.007 | 0.801 | 0.001 | None | Rnf39 | ring finger protein 39 |
| ENSMUSG00000036501 | 2.793 | 4.484 | -0.591 | 0.001 | None | Fam13b | family with sequence similarity 13, member B |
| ENSMUSG00000036526 | 0.166 | 0.396 | -1.158 | 0.041 | K07367 | Card11 | caspase recruitment domain family, member 11 |
| ENSMUSG00000036545 | 5.696 | 8.196 | -0.432 | 0.000 | K08618 | Adamts2 | a disintegrin-like and metallopeptidase (reprolysin type) with thrombospondin type 1 motif, 2 |
| ENSMUSG00000036594 | 56.372 | 165.253 | -1.465 | 0.000 | K06752 | H2-Aa | histocompatibility 2, class II antigen A, alpha |
| ENSMUSG00000036611 | 10.704 | 14.979 | -0.392 | 0.000 | None | Eepd1 | endonuclease/exonuclease/phosphatase family domain containing 1 |
| ENSMUSG00000036745 | 1.579 | 0.939 | 0.843 | 0.001 | K16583 | Ttll7 | tubulin tyrosine ligase-like family, member 7 |
| ENSMUSG00000036820 | 10.190 | 8.265 | 0.395 | 0.010 | K01443 | Amdhd2 | amidohydrolase domain containing 2 |
| ENSMUSG00000036862 | 2.324 | 3.536 | -0.512 | 0.002 | K16507 | Dchs1 | dachsous cadherin related 1 |
| ENSMUSG00000036863 | 4.039 | 3.178 | 0.438 | 0.012 | None | Syde2 | synapse defective 1, Rho GTPase, homolog 2 (C. elegans) |
| ENSMUSG00000036880 | 1074.149 | 790.025 | 0.537 | 0.000 | K07508 | Acaa2 | acetyl-Coenzyme A acyltransferase 2 (mitochondrial 3-oxoacyl-Coenzyme A thiolase) |
| ENSMUSG00000036908 | 16.837 | 28.060 | -0.644 | 0.000 | None | Unc93b1 | unc-93 homolog B1 (C. elegans) |
| ENSMUSG00000036995 | 3.393 | 5.124 | -0.503 | 0.001 | K12488 | Asap3 | ArfGAP with SH3 domain, ankyrin repeat and PH domain 3 |
| ENSMUSG00000037003 | 79.187 | 53.881 | 0.649 | 0.000 | K18080 | Tns2 | tensin 2 |
| ENSMUSG00000037010 | 2.022 | 5.717 | -1.407 | 0.000 | K05225 | Apln | apelin |
| ENSMUSG00000037031 | 5.363 | 8.008 | -0.485 | 0.001 | K17297 | Tspan15 | tetraspanin 15 |
| ENSMUSG00000037095 | 91.256 | 49.972 | 0.962 | 0.000 | None | Lrg1 | leucine-rich alpha-2-glycoprotein 1 |
| ENSMUSG00000037139 | 1.274 | 0.779 | 0.805 | 0.000 | None | Myom3 | myomesin family, member 3 |
| ENSMUSG00000037145 | 0.499 | 1.975 | -1.887 | 0.001 | None | 2210407C18Rik | RIKEN cDNA 2210407C18 gene |
| ENSMUSG00000037157 | 0.652 | 0.224 | 1.630 | 0.000 | K05138 | Il22ra1 | interleukin 22 receptor, alpha 1 |
| ENSMUSG00000037169 | 0.664 | 1.956 | -1.466 | 0.000 | K09109 | Mycn | v-myc avian myelocytomatosis viral related oncogene, neuroblastoma derived |
| ENSMUSG00000037204 | 27.216 | 20.957 | 0.470 | 0.001 | K19730 | Atg101 | autophagy related 101 |
| ENSMUSG00000037337 | 0.526 | 1.175 | -1.070 | 0.012 | K04408 | Map4k1 | mitogen-activated protein kinase kinase kinase kinase 1 |
| ENSMUSG00000037347 | 3.240 | 5.319 | -0.622 | 0.004 | K04743 | Chst7 | carbohydrate (N-acetylglucosamino) sulfotransferase 7 |
| ENSMUSG00000037355 | 1.291 | 0.954 | 0.527 | 0.030 | None | Uvssa | UV stimulated scaffold protein A |
| ENSMUSG00000037379 | 4.213 | 7.984 | -0.832 | 0.003 | None | Spon2 | spondin 2, extracellular matrix protein |
| ENSMUSG00000037411 | 33.232 | 18.160 | 0.964 | 0.000 | K03982 | Serpine1 | serine (or cysteine) peptidase inhibitor, clade E, member 1 |
| ENSMUSG00000037461 | 2.978 | 4.375 | -0.461 | 0.016 | K13144 | Ints7 | integrator complex subunit 7 |
| ENSMUSG00000037548 | 0.650 | 1.733 | -1.318 | 0.047 | K06752 | H2-DMb2 | histocompatibility 2, class II, locus Mb2 |
| ENSMUSG00000037583 | 13.528 | 10.590 | 0.446 | 0.006 | K08563 | Nr0b2 | nuclear receptor subfamily 0, group B, member 2 |
| ENSMUSG00000037649 | 3.116 | 10.195 | -1.615 | 0.000 | K06752 | H2-DMa | histocompatibility 2, class II, locus DMa |
| ENSMUSG00000037661 | 4.347 | 1.978 | 1.233 | 0.000 | K08438 | Gpr160 | G protein-coupled receptor 160 |
| ENSMUSG00000037679 | 14.197 | 10.584 | 0.519 | 0.000 | None | Inf2 | inverted formin, FH2 and WH2 domain containing |
| ENSMUSG00000037686 | 0.798 | 0.329 | 1.370 | 0.006 | K13278 | Aspg | asparaginase |
| ENSMUSG00000037731 | 2.173 | 3.779 | -0.704 | 0.002 | None | Themis2 | thymocyte selection associated family member 2 |
| ENSMUSG00000037754 | 4.129 | 5.838 | -0.406 | 0.008 | K17459 | Ppp1r16b | protein phosphatase 1, regulatory (inhibitor) subunit 16B |
| ENSMUSG00000037762 | 2.838 | 2.000 | 0.597 | 0.009 | K08186 | Slc16a9 | solute carrier family 16 (monocarboxylic acid transporters), member 9 |
| ENSMUSG00000037813 | 2.854 | 4.757 | -0.644 | 0.000 | None | D630003M21Rik | RIKEN cDNA D630003M21 gene |
| ENSMUSG00000037892 | 1.520 | 2.340 | -0.529 | 0.012 | K16499 | Pcdh18 | protocadherin 18 |
| ENSMUSG00000037902 | 10.762 | 15.123 | -0.398 | 0.011 | K06551 | Sirpa | signal-regulatory protein alpha |
| ENSMUSG00000037904 | 28.959 | 19.276 | 0.682 | 0.000 | None | Ankrd9 | ankyrin repeat domain 9 |
| ENSMUSG00000037946 | 0.615 | 1.208 | -0.879 | 0.002 | K05722 | Fgd3 | FYVE, RhoGEF and PH domain containing 3 |
| ENSMUSG00000037966 | 75.602 | 57.346 | 0.492 | 0.000 | None | Ninj1 | ninjurin 1 |
| ENSMUSG00000037995 | 0.154 | 0.439 | -1.415 | 0.003 | None | Igsf9 | immunoglobulin superfamily, member 9 |
| ENSMUSG00000037997 | 1.809 | 3.315 | -0.781 | 0.000 | K15259 | Parp11 | poly (ADP-ribose) polymerase family, member 11 |
| ENSMUSG00000038007 | 15.460 | 9.949 | 0.727 | 0.000 | K01441 | Acer2 | alkaline ceramidase 2 |
| ENSMUSG00000038028 | 17.920 | 14.522 | 0.397 | 0.000 | K14634 | Tigar | Trp53 induced glycolysis repulatory phosphatase |
| ENSMUSG00000038151 | 0.445 | 0.931 | -0.970 | 0.008 | None | Prdm1 | PR domain containing 1, with ZNF domain |
| ENSMUSG00000038152 | 3.267 | 2.013 | 0.792 | 0.039 |  | 5033430I15Rik | RIKEN cDNA 5033430I15 gene |
| ENSMUSG00000038175 | 32.232 | 17.765 | 0.952 | 0.000 | K10637 | Mylip | myosin regulatory light chain interacting protein |
| ENSMUSG00000038178 | 10.254 | 6.794 | 0.687 | 0.000 | K08229 | Slc43a2 | solute carrier family 43, member 2 |
| ENSMUSG00000038179 | 0.186 | 0.429 | -1.105 | 0.046 | K06733 | Slamf7 | SLAM family member 7 |
| ENSMUSG00000038181 | 3.296 | 2.574 | 0.450 | 0.001 | K03419 | Chpf2 | chondroitin polymerizing factor 2 |
| ENSMUSG00000038298 | 1.575 | 0.916 | 0.877 | 0.001 | None | Pdzk1 | PDZ domain containing 1 |
| ENSMUSG00000038319 | 32.315 | 20.788 | 0.731 | 0.000 | K04905 | Kcnh2 | potassium voltage-gated channel, subfamily H (eag-related), member 2 |
| ENSMUSG00000038417 | 23.316 | 17.050 | 0.545 | 0.000 | None | Fig4 | FIG4 phosphoinositide 5-phosphatase |
| ENSMUSG00000038457 | 6.953 | 12.622 | -0.768 | 0.000 | None | Tmem255b | transmembrane protein 255B |
| ENSMUSG00000038486 | 1.901 | 1.181 | 0.780 | 0.000 | K06258 | Sv2a | synaptic vesicle glycoprotein 2 a |
| ENSMUSG00000038508 | 0.813 | 1.587 | -0.872 | 0.031 | K05504 | Gdf15 | growth differentiation factor 15 |
| ENSMUSG00000038526 | 83.219 | 61.122 | 0.538 | 0.000 | K01672 | Car14 | carbonic anhydrase 14 |
| ENSMUSG00000038528 | 3.161 | 1.730 | 0.960 | 0.000 | None | Mfsd4b5 | major facilitator superfamily domain containing 4B5 |
| ENSMUSG00000038530 | 5.414 | 9.181 | -0.669 | 0.000 | K16449 | Rgs4 | regulator of G-protein signaling 4 |
| ENSMUSG00000038552 | 1.260 | 2.107 | -0.650 | 0.021 | None | Fndc4 | fibronectin type III domain containing 4 |
| ENSMUSG00000038618 | 2.097 | 1.100 | 1.024 | 0.002 | K09855 | Rassf7 | Ras association (RalGDS/AF-6) domain family (N-terminal) member 7 |
| ENSMUSG00000038622 | 14.808 | 12.029 | 0.392 | 0.015 | K15143 | Med30 | mediator complex subunit 30 |
| ENSMUSG00000038623 | 2.679 | 3.858 | -0.434 | 0.014 | None | Tm6sf1 | transmembrane 6 superfamily member 1 |
| ENSMUSG00000038642 | 16.190 | 29.265 | -0.763 | 0.000 | K01368 | Ctss | cathepsin S |
| ENSMUSG00000038668 | 2.268 | 3.270 | -0.435 | 0.013 | K04289 | Lpar1 | lysophosphatidic acid receptor 1 |
| ENSMUSG00000038670 | 3.652 | 9.316 | -1.258 | 0.000 | K12558 | Mybpc2 | myosin binding protein C, fast-type |
| ENSMUSG00000038732 | 1.238 | 2.393 | -0.857 | 0.000 | K13517 | Mboat1 | membrane bound O-acyltransferase domain containing 1 |
| ENSMUSG00000038776 | 52.879 | 43.070 | 0.389 | 0.003 | K01253 | Ephx1 | epoxide hydrolase 1, microsomal |
| ENSMUSG00000038807 | 13.579 | 18.873 | -0.382 | 0.000 | K17708 | Rap1gap2 | RAP1 GTPase activating protein 2 |
| ENSMUSG00000038860 | 2.369 | 1.438 | 0.813 | 0.000 | None | Garnl3 | GTPase activating RANGAP domain-like 3 |
| ENSMUSG00000039031 | 5.010 | 7.729 | -0.533 | 0.001 | None | Arhgap18 | Rho GTPase activating protein 18 |
| ENSMUSG00000039115 | 3.165 | 5.873 | -0.799 | 0.000 | K06585 | Itga9 | integrin alpha 9 |
| ENSMUSG00000039145 | 1.235 | 1.729 | -0.392 | 0.049 | K08794 | Camk1d | calcium/calmodulin-dependent protein kinase ID |
| ENSMUSG00000039208 | 9.675 | 13.911 | -0.431 | 0.009 | None | Metrnl | meteorin, glial cell differentiation regulator-like |
| ENSMUSG00000039232 | 0.868 | 1.439 | -0.636 | 0.026 | K08487 | Stx11 | syntaxin 11 |
| ENSMUSG00000039264 | 1.016 | 2.244 | -1.048 | 0.006 | None | Gimap3 | GTPase, IMAP family member 3 |
| ENSMUSG00000039347 | 12.464 | 9.964 | 0.416 | 0.013 | K02153 | Atp6v0e2 | ATPase, H+ transporting, lysosomal V0 subunit E2 |
| ENSMUSG00000039450 | 17.924 | 14.199 | 0.428 | 0.003 | K03331 | Dcxr | dicarbonyl L-xylulose reductase |
| ENSMUSG00000039457 | 3.706 | 2.504 | 0.656 | 0.001 | K10386 | Ppl | periplakin |
| ENSMUSG00000039461 | 62.371 | 50.568 | 0.396 | 0.000 | None | Tcta | T cell leukemia translocation altered gene |
| ENSMUSG00000039476 | 0.255 | 0.753 | -1.466 | 0.035 | K09329 | Prrx2 | paired related homeobox 2 |
| ENSMUSG00000039497 | 2.089 | 3.008 | -0.433 | 0.007 | K01794 | Dse | dermatan sulfate epimerase |
| ENSMUSG00000039616 | 5.456 | 3.939 | 0.564 | 0.001 | K15631 | Mocos | molybdenum cofactor sulfurase |
| ENSMUSG00000039661 | 1.927 | 0.558 | 1.886 | 0.000 | K14165 | Dusp26 | dual specificity phosphatase 26 (putative) |
| ENSMUSG00000039783 | 0.223 | 0.670 | -1.492 | 0.027 | K00486 | Kmo | kynurenine 3-monooxygenase (kynurenine 3-hydroxylase) |
| ENSMUSG00000039787 | 1.353 | 2.181 | -0.593 | 0.036 | None | Cercam | cerebral endothelial cell adhesion molecule |
| ENSMUSG00000039903 | 2.418 | 1.277 | 1.017 | 0.000 | None | Eva1c | eva-1 homolog C (C. elegans) |
| ENSMUSG00000039910 | 38.590 | 30.155 | 0.449 | 0.000 | None | Cited2 | Cbp/p300-interacting transactivator, with Glu/Asp-rich carboxy-terminal domain, 2 |
| ENSMUSG00000039911 | 13.935 | 9.991 | 0.575 | 0.003 | K10343 | Spsb1 | splA/ryanodine receptor domain and SOCS box containing 1 |
| ENSMUSG00000039934 | 0.717 | 1.320 | -0.791 | 0.007 | None | Gsap | gamma-secretase activating protein |
| ENSMUSG00000039956 | 7.970 | 5.260 | 0.694 | 0.000 | None | Mrap | melanocortin 2 receptor accessory protein |
| ENSMUSG00000039958 | 15.734 | 12.780 | 0.392 | 0.002 | None | Etfbkmt | electron transfer flavoprotein beta subunit lysine methyltransferase |
| ENSMUSG00000039981 | 0.182 | 0.429 | -1.141 | 0.039 | K18668 | Zc3h12d | zinc finger CCCH type containing 12D |
| ENSMUSG00000039994 | 2.707 | 5.371 | -0.895 | 0.000 | K03155 | Timeless | timeless circadian clock 1 |
| ENSMUSG00000040033 | 5.857 | 9.304 | -0.576 | 0.000 | K11221 | Stat2 | signal transducer and activator of transcription 2 |
| ENSMUSG00000040170 | 24.610 | 13.103 | 1.000 | 0.000 | K00485 | Fmo2 | flavin containing monooxygenase 2 |
| ENSMUSG00000040212 | 19.654 | 28.806 | -0.459 | 0.006 | None | Emp3 | epithelial membrane protein 3 |
| ENSMUSG00000040364 | 0.226 | 0.048 | 2.334 | 0.037 | K00718 | Sec1 | secretory blood group 1 |
| ENSMUSG00000040428 | 1.879 | 2.757 | -0.459 | 0.044 | None | Plekha4 | pleckstrin homology domain containing, family A (phosphoinositide binding specific) member 4 |
| ENSMUSG00000040483 | 5.940 | 9.460 | -0.578 | 0.000 | None | Xaf1 | XIAP associated factor 1 |
| ENSMUSG00000040490 | 0.096 | 0.305 | -1.583 | 0.018 | K16355 | Lrfn2 | leucine rich repeat and fibronectin type III domain containing 2 |
| ENSMUSG00000040592 | 0.963 | 3.047 | -1.567 | 0.005 | K06507 | Cd79b | CD79B antigen |
| ENSMUSG00000040653 | 28.987 | 40.963 | -0.407 | 0.000 | K17556 | Ppp1r14c | protein phosphatase 1, regulatory (inhibitor) subunit 14c |
| ENSMUSG00000040659 | 38.434 | 29.064 | 0.497 | 0.000 | None | Efhd2 | EF hand domain containing 2 |
| ENSMUSG00000040666 | 52.542 | 85.319 | -0.607 | 0.000 | None | Sh3bgr | SH3-binding domain glutamic acid-rich protein |
| ENSMUSG00000040690 | 2.492 | 4.059 | -0.611 | 0.000 | None | Col16a1 | collagen, type XVI, alpha 1 |
| ENSMUSG00000040711 | 2.349 | 3.615 | -0.529 | 0.000 | None | Sh3pxd2b | SH3 and PX domains 2B |
| ENSMUSG00000040724 | 1.017 | 0.753 | 0.527 | 0.004 | K04875 | Kcna2 | potassium voltage-gated channel, shaker-related subfamily, member 2 |
| ENSMUSG00000040740 | 236.104 | 183.973 | 0.454 | 0.000 | K15117 | Slc25a34 | solute carrier family 25, member 34 |
| ENSMUSG00000040747 | 3.748 | 6.429 | -0.685 | 0.009 | K06489 | Cd53 | CD53 antigen |
| ENSMUSG00000040797 | 0.674 | 1.130 | -0.650 | 0.011 | K12495 | Iqsec3 | IQ motif and Sec7 domain 3 |
| ENSMUSG00000040820 | 3.842 | 2.908 | 0.495 | 0.001 | K01942 | Hlcs | holocarboxylase synthetase (biotin- [propriony-Coenzyme A-carboxylase (ATP-hydrolysing)] ligase) |
| ENSMUSG00000040950 | 5.229 | 9.955 | -0.835 | 0.000 | None | Mgl2 | macrophage galactose N-acetyl-galactosamine specific lectin 2 |
| ENSMUSG00000040957 | 2.523 | 3.568 | -0.407 | 0.024 | None | Cables1 | CDK5 and Abl enzyme substrate 1 |
| ENSMUSG00000041040 | 4.242 | 2.967 | 0.611 | 0.003 | None | Fam117b | family with sequence similarity 117, member B |
| ENSMUSG00000041046 | 5.288 | 8.116 | -0.526 | 0.020 | K08449 | Ramp3 | receptor (calcitonin) activity modifying protein 3 |
| ENSMUSG00000041096 | 9.866 | 7.243 | 0.540 | 0.000 | K11285 | Tspyl2 | TSPY-like 2 |
| ENSMUSG00000041112 | 2.681 | 3.997 | -0.483 | 0.000 | K12366 | Elmo1 | engulfment and cell motility 1 |
| ENSMUSG00000041143 | 9.817 | 7.189 | 0.543 | 0.000 | None | Tmco4 | transmembrane and coiled-coil domains 4 |
| ENSMUSG00000041193 | 9.659 | 14.293 | -0.473 | 0.004 | K01047 | Pla2g5 | phospholipase A2, group V |
| ENSMUSG00000041202 | 1.685 | 3.254 | -0.856 | 0.006 | K01047 | Pla2g2d | phospholipase A2, group IID |
| ENSMUSG00000041219 | 0.447 | 0.770 | -0.691 | 0.020 | None | Arhgap11a | Rho GTPase activating protein 11A |
| ENSMUSG00000041361 | 142.699 | 101.846 | 0.580 | 0.000 | None | Myzap | myocardial zonula adherens protein |
| ENSMUSG00000041417 | 19.522 | 10.745 | 0.955 | 0.000 | K02649 | Pik3r1 | phosphoinositide-3-kinase regulatory subunit 1 |
| ENSMUSG00000041556 | 0.733 | 0.242 | 1.694 | 0.023 | K10099 | Fbxo2 | F-box protein 2 |
| ENSMUSG00000041592 | 0.907 | 0.300 | 1.692 | 0.000 | K16353 | Sdk2 | sidekick cell adhesion molecule 2 |
| ENSMUSG00000041598 | 17.326 | 11.445 | 0.691 | 0.000 | None | Cdc42ep4 | CDC42 effector protein (Rho GTPase binding) 4 |
| ENSMUSG00000041633 | 2.170 | 3.349 | -0.533 | 0.002 | None | Kctd12b | potassium channel tetramerisation domain containing 12b |
| ENSMUSG00000041773 | 11.011 | 8.313 | 0.498 | 0.000 | K10462 | Enc1 | ectodermal-neural cortex 1 |
| ENSMUSG00000041782 | 3.817 | 2.807 | 0.538 | 0.021 | None | Lad1 | ladinin |
| ENSMUSG00000041794 | 4.375 | 2.627 | 0.829 | 0.000 | None | Myrip | myosin VIIA and Rab interacting protein |
| ENSMUSG00000041797 | 4.637 | 6.568 | -0.409 | 0.002 | K05651 | Abca9 | ATP-binding cassette, sub-family A (ABC1), member 9 |
| ENSMUSG00000041817 | 0.181 | 0.058 | 1.755 | 0.002 | None | Fam169a | family with sequence similarity 169, member A |
| ENSMUSG00000041840 | 0.376 | 1.917 | -2.254 | 0.016 | K16584 | Haus1 | HAUS augmin-like complex, subunit 1 |
| ENSMUSG00000041889 | 11.248 | 6.667 | 0.848 | 0.000 | None | Shisa4 | shisa family member 4 |
| ENSMUSG00000041926 | 7.866 | 14.011 | -0.741 | 0.000 | K01260 | Rnpep | arginyl aminopeptidase (aminopeptidase B) |
| ENSMUSG00000041959 | 48.721 | 74.345 | -0.518 | 0.000 | K17274 | S100a10 | S100 calcium binding protein A10 (calpactin) |
| ENSMUSG00000041986 | 0.159 | 0.034 | 2.351 | 0.035 | None | Elmod1 | ELMO/CED-12 domain containing 1 |
| ENSMUSG00000042010 | 93.798 | 76.298 | 0.392 | 0.000 | K01946 | Acacb | acetyl-Coenzyme A carboxylase beta |
| ENSMUSG00000042045 | 58.684 | 128.351 | -1.031 | 0.022 | None | Sln | sarcolipin |
| ENSMUSG00000042129 | 6.955 | 5.073 | 0.549 | 0.000 | K09851 | Rassf4 | Ras association (RalGDS/AF-6) domain family member 4 |
| ENSMUSG00000042225 | 1.423 | 1.055 | 0.523 | 0.012 | None | Ammecr1 | Alport syndrome, mental retardation, midface hypoplasia and elliptocytosis chromosomal region gene 1 |
| ENSMUSG00000042284 | 3.879 | 5.605 | -0.439 | 0.001 | K06480 | Itga1 | integrin alpha 1 |
| ENSMUSG00000042302 | 8.967 | 12.515 | -0.388 | 0.007 | None | Ehbp1 | EH domain binding protein 1 |
| ENSMUSG00000042359 | 1.823 | 2.782 | -0.517 | 0.023 | K20463 | Osbpl6 | oxysterol binding protein-like 6 |
| ENSMUSG00000042436 | 13.894 | 26.266 | -0.828 | 0.000 | None | Mfap4 | microfibrillar-associated protein 4 |
| ENSMUSG00000042453 | 1.869 | 0.985 | 1.016 | 0.000 | K06249 | Reln | reelin |
| ENSMUSG00000042500 | 2.182 | 3.033 | -0.383 | 0.013 | K11593 | Ago4 | argonaute RISC catalytic subunit 4 |
| ENSMUSG00000042529 | 16.225 | 9.358 | 0.887 | 0.000 | K05005 | Kcnj12 | potassium inwardly-rectifying channel, subfamily J, member 12 |
| ENSMUSG00000042589 | 0.754 | 1.837 | -1.192 | 0.000 | K09313 | Cux2 | cut-like homeobox 2 |
| ENSMUSG00000042622 | 14.711 | 10.467 | 0.584 | 0.000 | K09037 | Maff | v-maf musculoaponeurotic fibrosarcoma oncogene family, protein F (avian) |
| ENSMUSG00000042757 | 2.065 | 1.597 | 0.463 | 0.013 | None | Tmem108 | transmembrane protein 108 |
| ENSMUSG00000042770 | 7.975 | 5.955 | 0.515 | 0.001 | None | Hebp1 | heme binding protein 1 |
| ENSMUSG00000042804 | 5.663 | 8.588 | -0.508 | 0.000 | K08437 | Gpr153 | G protein-coupled receptor 153 |
| ENSMUSG00000042807 | 2.506 | 1.836 | 0.543 | 0.000 | K12168 | Hecw2 | HECT, C2 and WW domain containing E3 ubiquitin protein ligase 2 |
| ENSMUSG00000042826 | 1.555 | 2.437 | -0.555 | 0.046 | K04358 | Fgf11 | fibroblast growth factor 11 |
| ENSMUSG00000042834 | 4.740 | 17.037 | -1.751 | 0.000 | None | Nrep | neuronal regeneration related protein |
| ENSMUSG00000042851 | 1.734 | 1.119 | 0.723 | 0.001 | None | Zc3h6 | zinc finger CCCH type containing 6 |
| ENSMUSG00000043003 | 0.489 | 0.258 | 1.010 | 0.008 | K17199 | Rasef | RAS and EF hand domain containing |
| ENSMUSG00000043067 | 1.106 | 1.600 | -0.441 | 0.039 | None | Dpy19l1 | dpy-19-like 1 (C. elegans) |
| ENSMUSG00000043091 | 22.257 | 16.027 | 0.568 | 0.000 | K07374 | Tuba1c | tubulin, alpha 1C |
| ENSMUSG00000043099 | 7.867 | 12.247 | -0.546 | 0.005 | None | Hic1 | hypermethylated in cancer 1 |
| ENSMUSG00000043122 | 36.020 | 26.356 | 0.543 | 0.000 | None | A530016L24Rik | RIKEN cDNA A530016L24 gene |
| ENSMUSG00000043230 | 0.672 | 1.521 | -1.082 | 0.003 | None | Fam124b | family with sequence similarity 124, member B |
| ENSMUSG00000043415 | 8.160 | 12.261 | -0.494 | 0.007 | K13716 | Otud1 | OTU domain containing 1 |
| ENSMUSG00000043432 | 3.244 | 2.256 | 0.617 | 0.001 | None | Leng9 | leukocyte receptor cluster (LRC) member 9 |
| ENSMUSG00000043557 | 1.035 | 2.013 | -0.866 | 0.000 | None | Mdga1 | MAM domain containing glycosylphosphatidylinositol anchor 1 |
| ENSMUSG00000043613 | 8.583 | 3.560 | 1.361 | 0.000 | K01394 | Mmp3 | matrix metallopeptidase 3 |
| ENSMUSG00000043629 | 0.198 | 0.483 | -1.190 | 0.010 | None | 1700019D03Rik | RIKEN cDNA 1700019D03 gene |
| ENSMUSG00000043668 | 1.275 | 0.846 | 0.687 | 0.017 | None | Tox3 | TOX high mobility group box family member 3 |
| ENSMUSG00000043683 | 69.267 | 54.725 | 0.434 | 0.000 | None | Fem1a | feminization 1 homolog a (C. elegans) |
| ENSMUSG00000043895 | 1.495 | 2.469 | -0.630 | 0.003 | K04292 | S1pr2 | sphingosine-1-phosphate receptor 2 |
| ENSMUSG00000043993 | 3.136 | 2.205 | 0.602 | 0.040 |  | 2900052L18Rik | RIKEN cDNA 2900052L18 gene |
| ENSMUSG00000044006 | 1.061 | 3.209 | -1.503 | 0.000 | None | Cilp2 | cartilage intermediate layer protein 2 |
| ENSMUSG00000044086 | 24.912 | 34.777 | -0.389 | 0.000 | None | Lmod3 | leiomodin 3 (fetal) |
| ENSMUSG00000044177 | 8.564 | 4.599 | 0.990 | 0.000 | None | Wfikkn2 | WAP, follistatin/kazal, immunoglobulin, kunitz and netrin domain containing 2 |
| ENSMUSG00000044206 | 4.335 | 2.180 | 1.089 | 0.005 | K19822 | Vsig4 | V-set and immunoglobulin domain containing 4 |
| ENSMUSG00000044216 | 0.483 | 0.181 | 1.510 | 0.020 | K04998 | Kcnj4 | potassium inwardly-rectifying channel, subfamily J, member 4 |
| ENSMUSG00000044285 | 57.012 | 46.605 | 0.385 | 0.006 |  | Gm1821 | ubiquitin pseudogene |
| ENSMUSG00000044337 | 31.208 | 45.547 | -0.451 | 0.000 | K04304 | Ackr3 | atypical chemokine receptor 3 |
| ENSMUSG00000044338 | 5.190 | 29.379 | -2.412 | 0.000 | K04174 | Aplnr | apelin receptor |
| ENSMUSG00000044350 | 1.518 | 2.875 | -0.827 | 0.001 | K05810 | Lacc1 | laccase domain containing 1 |
| ENSMUSG00000044442 | 5.303 | 4.261 | 0.407 | 0.004 | K19589 | N6amt1 | N-6 adenine-specific DNA methyltransferase 1 (putative) |
| ENSMUSG00000044716 | 15.907 | 11.752 | 0.531 | 0.000 | None | Dok7 | docking protein 7 |
| ENSMUSG00000044734 | 1.763 | 3.054 | -0.700 | 0.010 | K13963 | Serpinb1a | serine (or cysteine) peptidase inhibitor, clade B, member 1a |
| ENSMUSG00000044768 | 0.086 | 0.138 | -0.580 | 0.028 | None | D1Ertd622e | DNA segment, Chr 1, ERATO Doi 622, expressed |
| ENSMUSG00000044811 | 1.847 | 4.146 | -1.070 | 0.001 | K06719 | Cd300c2 | CD300C molecule 2 |
| ENSMUSG00000044951 | 16.977 | 25.502 | -0.494 | 0.000 | K00907 | Mylk4 | myosin light chain kinase family, member 4 |
| ENSMUSG00000044952 | 1.641 | 2.733 | -0.644 | 0.036 | None | Kctd21 | potassium channel tetramerisation domain containing 21 |
| ENSMUSG00000045094 | 2.351 | 1.715 | 0.548 | 0.018 | None | Arhgef37 | Rho guanine nucleotide exchange factor (GEF) 37 |
| ENSMUSG00000045193 | 93.985 | 54.922 | 0.868 | 0.000 | K13195 | Cirbp | cold inducible RNA binding protein |
| ENSMUSG00000045215 | 0.097 | 0.209 | -1.018 | 0.013 | K11471 | Asxl3 | additional sex combs like 3, transcriptional regulator |
| ENSMUSG00000045287 | 4.275 | 3.069 | 0.571 | 0.000 | K16660 | Rtn4rl1 | reticulon 4 receptor-like 1 |
| ENSMUSG00000045319 | 1.726 | 1.095 | 0.750 | 0.000 | None | Proser2 | proline and serine rich 2 |
| ENSMUSG00000045382 | 5.541 | 8.400 | -0.508 | 0.028 | K04189 | Cxcr4 | chemokine (C-X-C motif) receptor 4 |
| ENSMUSG00000045392 | 17.688 | 10.482 | 0.844 | 0.005 | K04257 | Olfr1033 | olfactory receptor 1033 |
| ENSMUSG00000045404 | 0.471 | 0.207 | 1.270 | 0.046 | K04922 | Kcnk13 | potassium channel, subfamily K, member 13 |
| ENSMUSG00000045573 | 9.447 | 6.263 | 0.685 | 0.000 | K18832 | Penk | preproenkephalin |
| ENSMUSG00000045629 | 1.932 | 3.185 | -0.628 | 0.001 | None | Sh3tc2 | SH3 domain and tetratricopeptide repeats 2 |
| ENSMUSG00000045761 | 0.654 | 1.491 | -1.101 | 0.000 | None | Togaram2 | TOG array regulator of axonemal microtubules 2 |
| ENSMUSG00000045868 | 0.125 | 0.256 | -0.934 | 0.046 | None | Gvin1 | GTPase, very large interferon inducible 1 |
| ENSMUSG00000045962 | 25.681 | 38.051 | -0.474 | 0.000 | K08867 | Wnk1 | WNK lysine deficient protein kinase 1 |
| ENSMUSG00000046157 | 3.171 | 4.790 | -0.502 | 0.006 | None | Tmem229b | transmembrane protein 229B |
| ENSMUSG00000046182 | 2.783 | 4.024 | -0.440 | 0.009 | None | Gsg1l | GSG1-like |
| ENSMUSG00000046186 | 0.584 | 1.335 | -1.097 | 0.000 | K06530 | Cd109 | CD109 antigen |
| ENSMUSG00000046318 | 2.066 | 1.210 | 0.864 | 0.000 | K19638 | Ccbe1 | collagen and calcium binding EGF domains 1 |
| ENSMUSG00000046413 | 0.627 | 0.255 | 1.383 | 0.023 |  | Irx3os | iroquois homeobox 3, opposite strand |
| ENSMUSG00000046415 | 3.384 | 2.591 | 0.478 | 0.022 |  | B430212C06Rik | RIKEN cDNA B430212C06 gene |
| ENSMUSG00000046447 | 10.076 | 6.972 | 0.624 | 0.000 | None | Camk2n1 | calcium/calmodulin-dependent protein kinase II inhibitor 1 |
| ENSMUSG00000046491 | 5.226 | 9.077 | -0.704 | 0.005 | None | C1qtnf2 | C1q and tumor necrosis factor related protein 2 |
| ENSMUSG00000046546 | 20.312 | 31.470 | -0.539 | 0.000 | None | Fam43a | family with sequence similarity 43, member A |
| ENSMUSG00000046694 | 1.326 | 0.349 | 2.026 | 0.000 | None | Fam46b | family with sequence similarity 46, member B |
| ENSMUSG00000046718 | 43.422 | 77.052 | -0.734 | 0.000 | K06731 | Bst2 | bone marrow stromal cell antigen 2 |
| ENSMUSG00000046722 | 6.969 | 10.586 | -0.510 | 0.000 | None | Cdc42se1 | CDC42 small effector 1 |
| ENSMUSG00000046731 | 2.052 | 3.002 | -0.455 | 0.042 | None | Kctd11 | potassium channel tetramerisation domain containing 11 |
| ENSMUSG00000046794 | 6.062 | 4.898 | 0.400 | 0.001 | K07189 | Ppp1r3b | protein phosphatase 1, regulatory (inhibitor) subunit 3B |
| ENSMUSG00000046818 | 1.023 | 2.462 | -1.173 | 0.000 | None | Ddit4l | DNA-damage-inducible transcript 4-like |
| ENSMUSG00000046879 | 2.556 | 4.502 | -0.722 | 0.001 | K14139 | Irgm1 | immunity-related GTPase family M member 1 |
| ENSMUSG00000046916 | 8.162 | 11.767 | -0.435 | 0.021 | None | Myct1 | myc target 1 |
| ENSMUSG00000046993 | 1.863 | 3.250 | -0.711 | 0.036 |  | Gm5637 | predicted pseudogene 5637 |
| ENSMUSG00000047085 | 16.862 | 8.480 | 1.086 | 0.000 | K16360 | Lrrc4b | leucine rich repeat containing 4B |
| ENSMUSG00000047129 | 0.703 | 0.216 | 1.780 | 0.040 | None | 1700113H08Rik | RIKEN cDNA 1700113H08 gene |
| ENSMUSG00000047180 | 3.812 | 8.020 | -0.979 | 0.000 | K15689 | Neurl3 | neuralized E3 ubiquitin protein ligase 3 |
| ENSMUSG00000047250 | 9.926 | 7.438 | 0.509 | 0.000 | K00509 | Ptgs1 | prostaglandin-endoperoxide synthase 1 |
| ENSMUSG00000047363 | 2.353 | 0.978 | 1.361 | 0.002 | None | Cstad | CSA-conditional, T cell activation-dependent protein |
| ENSMUSG00000047423 | 39.110 | 31.114 | 0.424 | 0.000 | None | AI837181 | expressed sequence AI837181 |
| ENSMUSG00000047466 | 0.103 | 0.448 | -2.022 | 0.036 | None | 8030462N17Rik | RIKEN cDNA 8030462N17 gene |
| ENSMUSG00000047485 | 1.904 | 3.303 | -0.702 | 0.028 | K10469 | Klhl34 | kelch-like 34 |
| ENSMUSG00000047497 | 0.384 | 0.786 | -0.937 | 0.000 | K08626 | Adamts12 | a disintegrin-like and metallopeptidase (reprolysin type) with thrombospondin type 1 motif, 12 |
| ENSMUSG00000047617 | 18.530 | 13.489 | 0.551 | 0.019 | None | Paxx | non-homologous end joining factor |
| ENSMUSG00000047676 | 24.516 | 19.235 | 0.442 | 0.046 | K02998 | Rpsa-ps10 | ribosomal protein SA, pseudogene 10 |
| ENSMUSG00000047867 | 14.501 | 20.317 | -0.393 | 0.016 | None | Gimap6 | GTPase, IMAP family member 6 |
| ENSMUSG00000047963 | 9.572 | 7.139 | 0.514 | 0.011 | None | Stbd1 | starch binding domain 1 |
| ENSMUSG00000048065 | 5.166 | 7.216 | -0.389 | 0.013 | K00326 | Cyb5r2 | cytochrome b5 reductase 2 |
| ENSMUSG00000048087 | 4.692 | 3.705 | 0.433 | 0.048 | K01251 | Gm4737 | predicted gene 4737 |
| ENSMUSG00000048126 | 3.664 | 8.686 | -1.152 | 0.000 | K06238 | Col6a3 | collagen, type VI, alpha 3 |
| ENSMUSG00000048163 | 2.846 | 5.433 | -0.841 | 0.009 | K06544 | Selplg | selectin, platelet (p-selectin) ligand |
| ENSMUSG00000048330 | 0.810 | 0.488 | 0.821 | 0.010 | None | Ric3 | RIC3 acetylcholine receptor chaperone |
| ENSMUSG00000048387 | 5.116 | 3.276 | 0.733 | 0.003 | K09215 | Osr1 | odd-skipped related transcription factor 1 |
| ENSMUSG00000048416 | 43.569 | 70.948 | -0.610 | 0.000 | K15622 | Mlf1 | myeloid leukemia factor 1 |
| ENSMUSG00000048756 | 32.223 | 22.866 | 0.589 | 0.000 | K09408 | Foxo3 | forkhead box O3 |
| ENSMUSG00000048779 | 3.852 | 6.365 | -0.631 | 0.000 | K04272 | P2ry6 | pyrimidinergic receptor P2Y, G-protein coupled, 6 |
| ENSMUSG00000048826 | 0.439 | 0.833 | -0.831 | 0.033 | None | Dact2 | dishevelled-binding antagonist of beta-catenin 2 |
| ENSMUSG00000048865 | 1.591 | 2.806 | -0.726 | 0.002 | None | Arhgap30 | Rho GTPase activating protein 30 |
| ENSMUSG00000048967 | 11.602 | 8.982 | 0.463 | 0.045 |  | Yjefn3 | YjeF N-terminal domain containing 3 |
| ENSMUSG00000049001 | 0.796 | 0.532 | 0.674 | 0.008 | None | Ndnf | neuron-derived neurotrophic factor |
| ENSMUSG00000049115 | 28.727 | 21.778 | 0.492 | 0.000 | K04166 | Agtr1a | angiotensin II receptor, type 1a |
| ENSMUSG00000049502 | 4.011 | 6.639 | -0.634 | 0.000 | K06058 | Dtx3l | deltex 3-like, E3 ubiquitin ligase |
| ENSMUSG00000049515 | 0.150 | 0.043 | 1.908 | 0.023 | None | Espnl | espin-like |
| ENSMUSG00000049625 | 0.703 | 1.945 | -1.374 | 0.000 | None | Tifab | TRAF-interacting protein with forkhead-associated domain, family member B |
| ENSMUSG00000049775 | 298.491 | 540.356 | -0.766 | 0.000 | K05764 | Tmsb4x | thymosin, beta 4, X chromosome |
| ENSMUSG00000049866 | 3.206 | 5.293 | -0.630 | 0.001 | K07945 | Arl4c | ADP-ribosylation factor-like 4C |
| ENSMUSG00000049892 | 10.782 | 6.839 | 0.753 | 0.001 | K07843 | Rasd1 | RAS, dexamethasone-induced 1 |
| ENSMUSG00000050014 | 1.767 | 4.688 | -1.314 | 0.000 | K14480 | Apol10b | apolipoprotein L 10B |
| ENSMUSG00000050064 | 5.459 | 3.816 | 0.610 | 0.000 | None | Zfp697 | zinc finger protein 697 |
| ENSMUSG00000050147 | 0.352 | 0.980 | -1.377 | 0.036 | K04236 | F2rl3 | coagulation factor II (thrombin) receptor-like 3 |
| ENSMUSG00000050578 | 0.060 | 0.666 | -3.340 | 0.000 | K07994 | Mmp13 | matrix metallopeptidase 13 |
| ENSMUSG00000050721 | 5.025 | 7.607 | -0.506 | 0.000 | None | Plekho2 | pleckstrin homology domain containing, family O member 2 |
| ENSMUSG00000050737 | 1.196 | 0.871 | 0.550 | 0.046 | K15729 | Ptges | prostaglandin E synthase |
| ENSMUSG00000050821 | 11.335 | 16.712 | -0.467 | 0.001 | None | Fam131a | family with sequence similarity 131, member A |
| ENSMUSG00000050830 | 0.662 | 0.204 | 1.794 | 0.001 | None | Vwc2 | von Willebrand factor C domain containing 2 |
| ENSMUSG00000050896 | 5.993 | 3.686 | 0.794 | 0.000 | K16661 | Rtn4rl2 | reticulon 4 receptor-like 2 |
| ENSMUSG00000051238 | 7.206 | 5.869 | 0.389 | 0.028 | None | Swsap1 | SWIM type zinc finger 7 associated protein 1 |
| ENSMUSG00000051457 | 0.730 | 1.376 | -0.819 | 0.041 | K06477 | Spn | sialophorin |
| ENSMUSG00000051506 | 0.578 | 1.156 | -0.909 | 0.002 | None | Wdfy4 | WD repeat and FYVE domain containing 4 |
| ENSMUSG00000051674 | 4.194 | 3.440 | 0.379 | 0.025 | K17824 | Dcun1d4 | DCN1, defective in cullin neddylation 1, domain containing 4 (S. cerevisiae) |
| ENSMUSG00000051735 | 1.125 | 2.433 | -1.018 | 0.001 | None | Rinl | Ras and Rab interactor-like |
| ENSMUSG00000051811 | 7.920 | 3.632 | 1.216 | 0.000 | K02267 | Cox6b2 | cytochrome c oxidase subunit VIb polypeptide 2 |
| ENSMUSG00000051855 | 2.487 | 5.256 | -0.986 | 0.000 | None | Mest | mesoderm specific transcript |
| ENSMUSG00000051978 | 4.646 | 3.449 | 0.522 | 0.020 | None | Erich1 | glutamate rich 1 |
| ENSMUSG00000051984 | 6.532 | 5.338 | 0.384 | 0.003 | K14005 | Sec31b | Sec31 homolog B (S. cerevisiae) |
| ENSMUSG00000052013 | 0.170 | 0.546 | -1.584 | 0.016 | K06707 | Btla | B and T lymphocyte associated |
| ENSMUSG00000052102 | 7.317 | 10.458 | -0.422 | 0.002 | K02564 | Gnpda1 | glucosamine-6-phosphate deaminase 1 |
| ENSMUSG00000052133 | 0.858 | 0.496 | 0.884 | 0.003 | K06841 | Sema5b | sema domain, seven thrombospondin repeats (type 1 and type 1-like), transmembrane domain (TM)  and short cytoplasmic domain, (semaphorin) 5B |
| ENSMUSG00000052142 | 0.200 | 0.609 | -1.506 | 0.002 | K17634 | Rasal3 | RAS protein activator like 3 |
| ENSMUSG00000052336 | 0.839 | 3.253 | -1.859 | 0.000 | K04192 | Cx3cr1 | chemokine (C-X3-C motif) receptor 1 |
| ENSMUSG00000052384 | 4.653 | 7.127 | -0.522 | 0.001 | None | Nrros | negative regulator of reactive oxygen species |
| ENSMUSG00000052396 | 1.269 | 2.228 | -0.723 | 0.030 | K14457 | Mogat2 | monoacylglycerol O-acyltransferase 2 |
| ENSMUSG00000052406 | 5.845 | 8.527 | -0.452 | 0.003 | K18327 | Rexo4 | REX4, 3'-5' exonuclease |
| ENSMUSG00000052512 | 10.955 | 8.022 | 0.542 | 0.000 | K19483 | Nav2 | neuron navigator 2 |
| ENSMUSG00000052593 | 5.635 | 9.480 | -0.657 | 0.000 | K06059 | Adam17 | a disintegrin and metallopeptidase domain 17 |
| ENSMUSG00000052688 | 1.368 | 2.160 | -0.568 | 0.014 | K07898 | Rab7b | RAB7B, member RAS oncogene family |
| ENSMUSG00000052776 | 3.107 | 5.622 | -0.762 | 0.000 | K14216 | Oas1a | 2'-5' oligoadenylate synthetase 1A |
| ENSMUSG00000052889 | 1.629 | 2.924 | -0.749 | 0.000 | K19662 | Prkcb | protein kinase C, beta |
| ENSMUSG00000053063 | 1.049 | 2.228 | -0.995 | 0.000 | K17516 | Clec12a | C-type lectin domain family 12, member a |
| ENSMUSG00000053093 | 228.141 | 34.367 | 2.827 | 0.000 | K17751 | Myh7 | myosin, heavy polypeptide 7, cardiac muscle, beta |
| ENSMUSG00000053214 | 4.553 | 3.731 | 0.379 | 0.049 |  | Gm9899 | predicted gene 9899 |
| ENSMUSG00000053317 | 11.677 | 17.359 | -0.479 | 0.003 | K09481 | Sec61b | Sec61 beta subunit |
| ENSMUSG00000053318 | 0.136 | 0.612 | -2.045 | 0.003 | K16853 | Slamf8 | SLAM family member 8 |
| ENSMUSG00000053411 | 12.968 | 9.453 | 0.549 | 0.000 | K11454 | Cbx7 | chromobox 7 |
| ENSMUSG00000053604 | 3.475 | 5.197 | -0.487 | 0.002 | K01807 | Rpia | ribose 5-phosphate isomerase A |
| ENSMUSG00000053719 | 0.688 | 0.096 | 2.966 | 0.000 | K01325 | Klk1b26 | kallikrein 1-related petidase b26 |
| ENSMUSG00000053886 | 5.689 | 7.989 | -0.397 | 0.007 | K17577 | Sh2d4a | SH2 domain containing 4A |
| ENSMUSG00000053898 | 2026.584 | 1235.248 | 0.807 | 0.000 | K12663 | Ech1 | enoyl coenzyme A hydratase 1, peroxisomal |
| ENSMUSG00000053977 | 0.178 | 0.459 | -1.268 | 0.047 | K06458 | Cd8a | CD8 antigen, alpha chain |
| ENSMUSG00000054013 | 10.732 | 6.538 | 0.807 | 0.000 | None | Tmem179 | transmembrane protein 179 |
| ENSMUSG00000054072 | 7.622 | 14.192 | -0.807 | 0.000 | None | Iigp1 | interferon inducible GTPase 1 |
| ENSMUSG00000054150 | 4.328 | 3.479 | 0.407 | 0.001 | None | Syne3 | spectrin repeat containing, nuclear envelope family member 3 |
| ENSMUSG00000054203 | 3.948 | 2.195 | 0.939 | 0.000 | None | Ifi205 | interferon activated gene 205 |
| ENSMUSG00000054428 | 46.416 | 33.077 | 0.582 | 0.000 | None | Atpif1 | ATPase inhibitory factor 1 |
| ENSMUSG00000054435 | 13.163 | 24.526 | -0.804 | 0.000 | None | Gimap4 | GTPase, IMAP family member 4 |
| ENSMUSG00000054499 | 7.902 | 6.439 | 0.388 | 0.000 | None | Dedd2 | death effector domain-containing DNA binding protein 2 |
| ENSMUSG00000054520 | 1.810 | 2.821 | -0.547 | 0.027 | K07984 | Sh3bp2 | SH3-domain binding protein 2 |
| ENSMUSG00000054641 | 5.137 | 3.111 | 0.813 | 0.000 | None | Mmrn1 | multimerin 1 |
| ENSMUSG00000054676 | 0.190 | 0.497 | -1.289 | 0.039 | None | 1600014C10Rik | RIKEN cDNA 1600014C10 gene |
| ENSMUSG00000054966 | 1.094 | 2.003 | -0.783 | 0.007 | None | Lmntd1 | lamin tail domain containing 1 |
| ENSMUSG00000055116 | 5.505 | 9.179 | -0.642 | 0.001 | K02296 | Arntl | aryl hydrocarbon receptor nuclear translocator-like |
| ENSMUSG00000055271 | 0.765 | 0.085 | 3.258 | 0.000 |  | 9330161L09Rik | RIKEN cDNA 9330161L09 gene |
| ENSMUSG00000055322 | 229.820 | 160.265 | 0.614 | 0.000 | K18080 | Tns1 | tensin 1 |
| ENSMUSG00000055413 | 7.498 | 12.689 | -0.665 | 0.003 |  | H2-Q5 | histocompatibility 2, Q region locus 5 |
| ENSMUSG00000055541 | 0.333 | 0.712 | -1.000 | 0.005 | K06725 | Lair1 | leukocyte-associated Ig-like receptor 1 |
| ENSMUSG00000055632 | 2.731 | 4.413 | -0.596 | 0.000 | K17341 | Hmcn2 | hemicentin 2 |
| ENSMUSG00000055660 | 0.942 | 1.421 | -0.502 | 0.019 | None | Mettl4 | methyltransferase like 4 |
| ENSMUSG00000055805 | 7.732 | 11.010 | -0.417 | 0.001 | None | Fmnl1 | formin-like 1 |
| ENSMUSG00000055866 | 4.653 | 1.272 | 1.966 | 0.000 | K02633 | Per2 | period circadian clock 2 |
| ENSMUSG00000055980 | 2.205 | 3.328 | -0.500 | 0.001 | K16172 | Irs1 | insulin receptor substrate 1 |
| ENSMUSG00000056025 | 0.975 | 0.476 | 1.125 | 0.000 | K05030 | Clca3a1 | chloride channel accessory 3A1 |
| ENSMUSG00000056054 | 4.850 | 2.248 | 1.204 | 0.009 | None | S100a8 | S100 calcium binding protein A8 (calgranulin A) |
| ENSMUSG00000056069 | 1.189 | 2.229 | -0.813 | 0.031 | None | Fam105a | family with sequence similarity 105, member A |
| ENSMUSG00000056071 | 13.367 | 4.283 | 1.736 | 0.009 | None | S100a9 | S100 calcium binding protein A9 (calgranulin B) |
| ENSMUSG00000056130 | 0.078 | 0.417 | -2.302 | 0.017 | K05409 | Ticam2 | toll-like receptor adaptor molecule 2 |
| ENSMUSG00000056209 | 10.352 | 14.477 | -0.391 | 0.020 | K11278 | Npm3 | nucleoplasmin 3 |
| ENSMUSG00000056290 | 0.142 | 0.460 | -1.590 | 0.001 | None | Ms4a4b | membrane-spanning 4-domains, subfamily A, member 4B |
| ENSMUSG00000056427 | 9.858 | 7.783 | 0.433 | 0.002 | K06850 | Slit3 | slit guidance ligand 3 |
| ENSMUSG00000056481 | 4.913 | 7.660 | -0.548 | 0.000 | K06706 | Cd248 | CD248 antigen, endosialin |
| ENSMUSG00000056515 | 7.297 | 10.297 | -0.404 | 0.000 | K07891 | Rab31 | RAB31, member RAS oncogene family |
| ENSMUSG00000056529 | 1.443 | 3.054 | -0.987 | 0.000 | K04279 | Ptafr | platelet-activating factor receptor |
| ENSMUSG00000056596 | 4.222 | 1.527 | 1.556 | 0.000 | None | Trnp1 | TMF1-regulated nuclear protein 1 |
| ENSMUSG00000056602 | 13.053 | 10.106 | 0.463 | 0.000 | None | Fry | FRY microtubule binding protein |
| ENSMUSG00000056666 | 36.869 | 29.188 | 0.431 | 0.000 | K09516 | Retsat | retinol saturase (all trans retinol 13,14 reductase) |
| ENSMUSG00000056698 | 18.101 | 14.737 | 0.390 | 0.000 | None | Elmod3 | ELMO/CED-12 domain containing 3 |
| ENSMUSG00000056917 | 11.747 | 17.748 | -0.501 | 0.000 | K08013 | Sipa1 | signal-induced proliferation associated gene 1 |
| ENSMUSG00000056938 | 15.684 | 12.450 | 0.426 | 0.000 | None | Acbd4 | acyl-Coenzyme A binding domain containing 4 |
| ENSMUSG00000057135 | 0.196 | 1.006 | -2.253 | 0.007 | None | Scimp | SLP adaptor and CSK interacting membrane protein |
| ENSMUSG00000057346 | 0.215 | 0.859 | -1.899 | 0.000 | K14480 | Apol9a | apolipoprotein L 9a |
| ENSMUSG00000057880 | 3.138 | 5.719 | -0.774 | 0.000 | K13524 | Abat | 4-aminobutyrate aminotransferase |
| ENSMUSG00000057897 | 4.378 | 7.314 | -0.648 | 0.000 | K04515 | Camk2b | calcium/calmodulin-dependent protein kinase II, beta |
| ENSMUSG00000057913 | 0.920 | 0.332 | 1.561 | 0.029 |  | Gm10032 | predicted gene 10032 |
| ENSMUSG00000058135 | 339.565 | 247.548 | 0.549 | 0.000 | K00799 | Gstm1 | glutathione S-transferase, mu 1 |
| ENSMUSG00000058173 | 24.322 | 38.817 | -0.581 | 0.000 | None | Smco4 | single-pass membrane protein with coiled-coil domains 4 |
| ENSMUSG00000058297 | 24.257 | 6.544 | 1.984 | 0.000 | K08136 | Spock2 | sparc/osteonectin, cwcv and kazal-like domains proteoglycan 2 |
| ENSMUSG00000058396 | 5.763 | 4.459 | 0.462 | 0.006 | K04242 | Gpr182 | G protein-coupled receptor 182 |
| ENSMUSG00000058672 | 17.877 | 24.813 | -0.380 | 0.002 | K07375 | Tubb2a | tubulin, beta 2A class IIA |
| ENSMUSG00000058715 | 15.811 | 25.829 | -0.615 | 0.013 | K07983 | Fcer1g | Fc receptor, IgE, high affinity I, gamma polypeptide |
| ENSMUSG00000058740 | 0.439 | 0.207 | 1.179 | 0.007 | K04946 | Kcnt1 | potassium channel, subfamily T, member 1 |
| ENSMUSG00000058806 | 1.551 | 0.965 | 0.776 | 0.002 | K16617 | Col13a1 | collagen, type XIII, alpha 1 |
| ENSMUSG00000058997 | 69.424 | 49.473 | 0.583 | 0.000 | None | Vwa8 | von Willebrand factor A domain containing 8 |
| ENSMUSG00000059013 | 25.034 | 20.172 | 0.406 | 0.001 | None | Sh2d3c | SH2 domain containing 3C |
| ENSMUSG00000059213 | 0.331 | 0.117 | 1.601 | 0.021 | None | Ddn | dendrin |
| ENSMUSG00000059401 | 4.822 | 3.895 | 0.401 | 0.010 | K19512 | Mamld1 | mastermind-like domain containing 1 |
| ENSMUSG00000059408 | 1.259 | 2.607 | -0.957 | 0.006 | None | Mrgprh | MAS-related GPR, member H |
| ENSMUSG00000059447 | 416.253 | 341.037 | 0.381 | 0.000 | K07509 | Hadhb | hydroxyacyl-Coenzyme A dehydrogenase/3-ketoacyl-Coenzyme A thiolase/enoyl-  Coenzyme A hydratase (trifunctional protein), beta subunit |
| ENSMUSG00000059456 | 6.666 | 4.714 | 0.593 | 0.000 | K05871 | Ptk2b | PTK2 protein tyrosine kinase 2 beta |
| ENSMUSG00000059498 | 15.873 | 24.850 | -0.555 | 0.023 | K16824 | Fcgr3 | Fc receptor, IgG, low affinity III |
| ENSMUSG00000059824 | 21.886 | 6.584 | 1.824 | 0.000 | K09056 | Dbp | D site albumin promoter binding protein |
| ENSMUSG00000059895 | 123.539 | 89.251 | 0.562 | 0.000 | K18041 | Ptp4a3 | protein tyrosine phosphatase 4a3 |
| ENSMUSG00000060187 | 156.037 | 113.350 | 0.557 | 0.000 | None | Lrrc10 | leucine rich repeat containing 10 |
| ENSMUSG00000060275 | 1.843 | 0.862 | 1.191 | 0.000 | K05456 | Nrg2 | neuregulin 2 |
| ENSMUSG00000060487 | 0.315 | 0.707 | -1.070 | 0.000 | None | Samd5 | sterile alpha motif domain containing 5 |
| ENSMUSG00000060586 | 54.984 | 152.811 | -1.389 | 0.000 | K06752 | H2-Eb1 | histocompatibility 2, class II antigen E beta |
| ENSMUSG00000060882 | 2.595 | 3.763 | -0.444 | 0.043 | K04892 | Kcnd2 | potassium voltage-gated channel, Shal-related family, member 2 |
| ENSMUSG00000060961 | 4.816 | 3.478 | 0.562 | 0.000 | K13575 | Slc4a4 | solute carrier family 4 (anion exchanger), member 4 |
| ENSMUSG00000061046 | 7.072 | 5.430 | 0.474 | 0.015 | K01069 | Haghl | hydroxyacylglutathione hydrolase-like |
| ENSMUSG00000061086 | 58.234 | 121.187 | -0.960 | 0.021 | K12750 | Myl4 | myosin, light polypeptide 4 |
| ENSMUSG00000061100 | 14.251 | 28.256 | -0.894 | 0.005 | None | Retnla | resistin like alpha |
| ENSMUSG00000061119 | 6.620 | 10.470 | -0.568 | 0.000 | K01285 | Prcp | prolylcarboxypeptidase (angiotensinase C) |
| ENSMUSG00000061132 | 0.689 | 1.397 | -0.925 | 0.000 | K07371 | Blnk | B cell linker |
| ENSMUSG00000061143 | 2.824 | 2.197 | 0.455 | 0.001 | K06061 | Maml3 | mastermind like transcriptional coactivator 3 |
| ENSMUSG00000061288 | 2.598 | 4.006 | -0.531 | 0.000 | K04429 | Taok3 | TAO kinase 3 |
| ENSMUSG00000061533 | 3.057 | 2.180 | 0.581 | 0.000 | K16460 | Cep128 | centrosomal protein 128 |
| ENSMUSG00000061740 | 23.897 | 15.413 | 0.726 | 0.000 | K07414 | Cyp2d22 | cytochrome P450, family 2, subfamily d, polypeptide 22 |
| ENSMUSG00000061762 | 1.526 | 0.470 | 1.780 | 0.000 | K05239 | Tac1 | tachykinin 1 |
| ENSMUSG00000061816 | 14.307 | 35.779 | -1.227 | 0.000 | K05738 | Myl1 | myosin, light polypeptide 1 |
| ENSMUSG00000062012 | 3.729 | 2.931 | 0.439 | 0.050 | K09228 | Zfp13 | zinc finger protein 13 |
| ENSMUSG00000062077 | 215.146 | 147.479 | 0.639 | 0.000 | K10653 | Trim54 | tripartite motif-containing 54 |
| ENSMUSG00000062081 | 100.334 | 77.037 | 0.474 | 0.000 | K10435 | Gm6055 | microtubule-associated protein 1 light chain 3 pseudogene 2 |
| ENSMUSG00000062115 | 8.649 | 6.874 | 0.424 | 0.000 | K19749 | Rai1 | retinoic acid induced 1 |
| ENSMUSG00000062309 | 3.646 | 1.705 | 1.188 | 0.000 | K14525 | Rpp25 | ribonuclease P/MRP 25 subunit |
| ENSMUSG00000062488 | 2.576 | 4.441 | -0.692 | 0.028 | None | Ifit3b | interferon-induced protein with tetratricopeptide repeats 3B |
| ENSMUSG00000062591 | 1.194 | 2.835 | -1.156 | 0.000 | K07375 | Tubb4a | tubulin, beta 4A class IVA |
| ENSMUSG00000062991 | 1.309 | 0.701 | 0.990 | 0.002 | K05455 | Nrg1 | neuregulin 1 |
| ENSMUSG00000063060 | 7.469 | 12.393 | -0.640 | 0.002 | K09270 | Sox7 | SRY (sex determining region Y)-box 7 |
| ENSMUSG00000063275 | 13.246 | 20.559 | -0.542 | 0.000 | K10703 | Hacd1 | 3-hydroxyacyl-CoA dehydratase 1 |
| ENSMUSG00000063388 | 1.091 | 5.860 | -2.331 | 0.000 | None | BC023105 | cDNA sequence BC023105 |
| ENSMUSG00000063445 | 3.468 | 2.510 | 0.561 | 0.009 | None | Nmral1 | NmrA-like family domain containing 1 |
| ENSMUSG00000063506 | 0.557 | 1.170 | -0.978 | 0.013 | None | Arhgap22 | Rho GTPase activating protein 22 |
| ENSMUSG00000063558 | 10.238 | 8.243 | 0.406 | 0.001 | K00157 | Aox1 | aldehyde oxidase 1 |
| ENSMUSG00000063564 | 2.190 | 1.395 | 0.741 | 0.001 | None | Col23a1 | collagen, type XXIII, alpha 1 |
| ENSMUSG00000063594 | 0.872 | 2.145 | -1.203 | 0.004 | K04544 | Gng8 | guanine nucleotide binding protein (G protein), gamma 8 |
| ENSMUSG00000063889 | 2.482 | 3.995 | -0.595 | 0.004 | K09052 | Crem | cAMP responsive element modulator |
| ENSMUSG00000064023 | 4.371 | 7.628 | -0.710 | 0.002 | K08650 | Klk8 | kallikrein related-peptidase 8 |
| ENSMUSG00000064215 | 37.089 | 67.971 | -0.781 | 0.000 | None | Ifi27 | interferon, alpha-inducible protein 27 |
| ENSMUSG00000064302 | 24.600 | 42.677 | -0.700 | 0.000 | K16578 | Clasp1 | CLIP associating protein 1 |
| ENSMUSG00000064367 | 5353.880 | 4330.656 | 0.396 | 0.010 | K03883 |  |  |
| ENSMUSG00000066170 | 0.807 | 0.165 | 2.390 | 0.000 |  | E230001N04Rik | RIKEN cDNA E230001N04 gene |
| ENSMUSG00000066245 | 5.303 | 2.942 | 0.946 | 0.005 |  | Gm10156 | predicted gene 10156 |
| ENSMUSG00000066687 | 26.565 | 12.504 | 1.185 | 0.001 | K10055 | Zbtb16 | zinc finger and BTB domain containing 16 |
| ENSMUSG00000066800 | 2.848 | 2.049 | 0.566 | 0.006 | K01165 | Rnasel | ribonuclease L (2', 5'-oligoisoadenylate synthetase-dependent) |
| ENSMUSG00000066861 | 0.571 | 1.320 | -1.112 | 0.028 | K14216 | Oas1g | 2'-5' oligoadenylate synthetase 1G |
| ENSMUSG00000066877 | 7.939 | 11.677 | -0.464 | 0.002 | K19862 | Nck2 | non-catalytic region of tyrosine kinase adaptor protein 2 |
| ENSMUSG00000066975 | 3.803 | 8.159 | -1.010 | 0.000 | None | Cryba4 | crystallin, beta A4 |
| ENSMUSG00000067028 | 17.237 | 13.527 | 0.442 | 0.014 | None | Cntnap5b | contactin associated protein-like 5B |
| ENSMUSG00000067158 | 1.407 | 2.183 | -0.543 | 0.011 | K06237 | Col4a4 | collagen, type IV, alpha 4 |
| ENSMUSG00000067377 | 4.406 | 7.830 | -0.736 | 0.000 | K17295 | Tspan6 | tetraspanin 6 |
| ENSMUSG00000068036 | 18.310 | 26.688 | -0.450 | 0.000 | K05702 | Afdn | afadin, adherens junction formation factor |
| ENSMUSG00000068196 | 3.014 | 4.591 | -0.514 | 0.001 | None | Col8a1 | collagen, type VIII, alpha 1 |
| ENSMUSG00000068245 | 2.810 | 5.123 | -0.773 | 0.000 | None | Phf11d | PHD finger protein 11D |
| ENSMUSG00000068606 | 0.329 | 2.148 | -2.620 | 0.003 | None | Gm4841 | predicted gene 4841 |
| ENSMUSG00000068735 | 26.937 | 44.934 | -0.645 | 0.023 | None | Trp53i11 | transformation related protein 53 inducible protein 11 |
| ENSMUSG00000068742 | 9.897 | 6.876 | 0.620 | 0.000 | K02295 | Cry2 | cryptochrome 2 (photolyase-like) |
| ENSMUSG00000068854 | 3.544 | 5.228 | -0.467 | 0.010 | K11252 | Hist2h2be | histone cluster 2, H2be |
| ENSMUSG00000068874 | 126.621 | 103.727 | 0.381 | 0.000 | K17285 | Selenbp1 | selenium binding protein 1 |
| ENSMUSG00000069662 | 6.501 | 10.129 | -0.546 | 0.000 | K12561 | Marcks | myristoylated alanine rich protein kinase C substrate |
| ENSMUSG00000069835 | 4.821 | 3.423 | 0.588 | 0.042 | K00657 | Sat2 | spermidine/spermine N1-acetyl transferase 2 |
| ENSMUSG00000069874 | 11.498 | 24.061 | -0.974 | 0.040 | None | Irgm2 | immunity-related GTPase family M member 2 |
| ENSMUSG00000069920 | 2.158 | 3.777 | -0.713 | 0.000 | K18705 | B3gnt9 | UDP-GlcNAc:betaGal beta-1,3-N-acetylglucosaminyltransferase 9 |
| ENSMUSG00000070327 | 2.852 | 4.325 | -0.507 | 0.000 | None | Rnf213 | ring finger protein 213 |
| ENSMUSG00000070436 | 61.391 | 127.399 | -0.960 | 0.000 | K09501 | Serpinh1 | serine (or cysteine) peptidase inhibitor, clade H, member 1 |
| ENSMUSG00000070604 | 1.691 | 1.194 | 0.593 | 0.012 | None | Vsig10l | V-set and immunoglobulin domain containing 10 like |
| ENSMUSG00000070637 | 1.638 | 0.836 | 1.066 | 0.038 | None | Srarp | steroid receptor associated and regulated protein |
| ENSMUSG00000070691 | 0.206 | 0.523 | -1.245 | 0.011 | K09279 | Runx3 | runt related transcription factor 3 |
| ENSMUSG00000070705 | 11.726 | 9.342 | 0.420 | 0.001 | None | Eid2b | EP300 interacting inhibitor of differentiation 2B |
| ENSMUSG00000070729 | 7.966 | 5.415 | 0.650 | 0.006 |  | Gm12966 | serine/arginine-rich splicing factor 5 pseudogene |
| ENSMUSG00000070802 | 2.838 | 2.001 | 0.597 | 0.002 | None | Pnmal2 | PNMA-like 2 |
| ENSMUSG00000070873 | 0.812 | 1.748 | -1.008 | 0.002 | K06512 | Lilra5 | leukocyte immunoglobulin-like receptor, subfamily A (with TM domain), member 5 |
| ENSMUSG00000071042 | 7.738 | 11.314 | -0.456 | 0.001 | K12362 | Rasgrp3 | RAS, guanyl releasing protein 3 |
| ENSMUSG00000071253 | 14.182 | 10.818 | 0.483 | 0.000 | K15084 | Slc25a16 | solute carrier family 25 (mitochondrial carrier, Graves disease autoantigen), member 16 |
| ENSMUSG00000071342 | 7.546 | 10.854 | -0.432 | 0.007 | None | Lsmem1 | leucine-rich single-pass membrane protein 1 |
| ENSMUSG00000071454 | 1.865 | 1.410 | 0.497 | 0.020 | None | Dtnb | dystrobrevin, beta |
| ENSMUSG00000071552 | 0.346 | 0.058 | 2.679 | 0.032 | K16350 | Tigit | T cell immunoreceptor with Ig and ITIM domains |
| ENSMUSG00000071637 | 15.419 | 10.559 | 0.639 | 0.000 |  | Cebpd | CCAAT/enhancer binding protein (C/EBP), delta |
| ENSMUSG00000071715 | 1.250 | 2.377 | -0.833 | 0.040 | K08012 | Ncf4 | neutrophil cytosolic factor 4 |
| ENSMUSG00000071984 | 4.021 | 6.222 | -0.537 | 0.000 | None | Fndc1 | fibronectin type III domain containing 1 |
| ENSMUSG00000072082 | 0.157 | 0.417 | -1.309 | 0.022 | K10289 | Ccnf | cyclin F |
| ENSMUSG00000072568 | 1.701 | 2.606 | -0.523 | 0.041 | None | Fam84b | family with sequence similarity 84, member B |
| ENSMUSG00000072949 | 74.929 | 35.845 | 1.157 | 0.000 | K01068 | Acot1 | acyl-CoA thioesterase 1 |
| ENSMUSG00000073421 | 54.392 | 164.382 | -1.508 | 0.000 | K06752 | H2-Ab1 | histocompatibility 2, class II antigen A, beta 1 |
| ENSMUSG00000073424 | 0.477 | 0.117 | 2.115 | 0.005 | K00490 | Cyp4f15 | cytochrome P450, family 4, subfamily f, polypeptide 15 |
| ENSMUSG00000073490 | 4.548 | 3.284 | 0.563 | 0.001 | None | Ifi207 | interferon activated gene 207 |
| ENSMUSG00000073491 | 0.575 | 1.135 | -0.886 | 0.048 | None | Ifi213 | interferon activated gene 213 |
| ENSMUSG00000073535 | 48.175 | 33.732 | 0.609 | 0.000 |  | Gm5532 | predicted gene 5532 |
| ENSMUSG00000074151 | 1.322 | 2.213 | -0.651 | 0.006 |  | Nlrc5 | NLR family, CARD domain containing 5 |
| ENSMUSG00000074207 | 8.807 | 5.662 | 0.731 | 0.000 | K13951 | Adh1 | alcohol dehydrogenase 1 (class I) |
| ENSMUSG00000074264 | 3.675 | 1.925 | 1.023 | 0.000 | K01176 | Amy1 | amylase 1, salivary |
| ENSMUSG00000074345 | 0.218 | 0.082 | 1.503 | 0.037 | None | Tnfaip8l3 | tumor necrosis factor, alpha-induced protein 8-like 3 |
| ENSMUSG00000074476 | 1.671 | 3.054 | -0.778 | 0.018 | K11549 | Spc24 | SPC24, NDC80 kinetochore complex component, homolog (S. cerevisiae) |
| ENSMUSG00000074529 | 18.583 | 12.842 | 0.624 | 0.006 | None | Zfp972 | zinc finger protein 972 |
| ENSMUSG00000074676 | 3.090 | 6.287 | -0.930 | 0.001 | K09412 | Foxs1 | forkhead box S1 |
| ENSMUSG00000074813 | 1.017 | 1.853 | -0.775 | 0.030 |  | Gm14005 | predicted gene 14005 |
| ENSMUSG00000074896 | 9.630 | 15.333 | -0.578 | 0.005 | None | Ifit3 | interferon-induced protein with tetratricopeptide repeats 3 |
| ENSMUSG00000075010 | 12.959 | 27.691 | -1.002 | 0.000 |  | AW112010 | expressed sequence AW112010 |
| ENSMUSG00000075033 | 0.742 | 0.469 | 0.755 | 0.009 | None | Nxpe3 | neurexophilin and PC-esterase domain family, member 3 |
| ENSMUSG00000075270 | 0.458 | 0.143 | 1.775 | 0.002 | K13298 | Pde11a | phosphodiesterase 11A |
| ENSMUSG00000075284 | 4.726 | 6.556 | -0.380 | 0.007 | K19475 | Wipf1 | WAS/WASL interacting protein family, member 1 |
| ENSMUSG00000075324 | 1.021 | 0.733 | 0.569 | 0.008 | None | Fign | fidgetin |
| ENSMUSG00000075408 | 3.491 | 1.772 | 1.068 | 0.000 |  | 6030408B16Rik | RIKEN cDNA 6030408B16 gene |
| ENSMUSG00000075704 | 0.385 | 1.944 | -2.234 | 0.017 | K00384 | Txnrd2 | thioredoxin reductase 2 |
| ENSMUSG00000076435 | 28.446 | 20.499 | 0.567 | 0.000 | K00666 | Acsf2 | acyl-CoA synthetase family member 2 |
| ENSMUSG00000076612 | 6.014 | 2.014 | 1.666 | 0.000 |  | Ighg2c | immunoglobulin heavy constant gamma 2C |
| ENSMUSG00000076617 | 9.928 | 22.283 | -1.076 | 0.004 |  | Ighm | immunoglobulin heavy constant mu |
| ENSMUSG00000076928 | 0.664 | 1.903 | -1.418 | 0.033 |  | Trac | T cell receptor alpha constant |
| ENSMUSG00000078137 | 1.239 | 0.536 | 1.306 | 0.000 | None | Ankrd63 | ankyrin repeat domain 63 |
| ENSMUSG00000078234 | 2.815 | 1.549 | 0.954 | 0.000 | None | Klhdc7a | kelch domain containing 7A |
| ENSMUSG00000078566 | 62.098 | 36.260 | 0.871 | 0.000 | K15464 | Bnip3 | BCL2/adenovirus E1B interacting protein 3 |
| ENSMUSG00000078607 | 1.796 | 1.193 | 0.685 | 0.036 |  | 1810010H24Rik | RIKEN cDNA 1810010H24 gene |
| ENSMUSG00000078932 | 0.410 | 1.138 | -1.385 | 0.001 |  | CN725425 | cDNA sequence CN725425 |
| ENSMUSG00000079017 | 43.860 | 78.649 | -0.749 | 0.000 | None | Ifi27l2a | interferon, alpha-inducible protein 27 like 2A |
| ENSMUSG00000079056 | 2.556 | 1.654 | 0.721 | 0.001 | None | Kcnip3 | Kv channel interacting protein 3, calsenilin |
| ENSMUSG00000079084 | 3.737 | 3.004 | 0.407 | 0.047 | None | Ccdc82 | coiled-coil domain containing 82 |
| ENSMUSG00000079105 | 5.551 | 10.330 | -0.803 | 0.000 | K03996 | C7 | complement component 7 |
| ENSMUSG00000079111 | 14.558 | 20.364 | -0.392 | 0.001 | K10949 | Kdelr2 | KDEL (Lys-Asp-Glu-Leu) endoplasmic reticulum protein retention receptor 2 |
| ENSMUSG00000079227 | 0.241 | 0.926 | -1.853 | 0.004 | K04180 | Ccr5 | chemokine (C-C motif) receptor 5 |
| ENSMUSG00000079242 | 2.230 | 1.288 | 0.883 | 0.012 |  | C730034F03Rik | RIKEN cDNA C730034F03 gene |
| ENSMUSG00000079293 | 1.143 | 3.181 | -1.379 | 0.000 | K10074 | Clec7a | C-type lectin domain family 7, member a |
| ENSMUSG00000079523 | 37.769 | 71.857 | -0.837 | 0.000 | K13785 | Tmsb10 | thymosin, beta 10 |
| ENSMUSG00000079547 | 3.818 | 9.661 | -1.247 | 0.000 | K06752 | H2-DMb1 | histocompatibility 2, class II, locus Mb1 |
| ENSMUSG00000083816 | 0.099 | 0.426 | -2.016 | 0.041 |  | Gm13033 | prostaglandin-endoperoxide synthase 2 pseudogene |
| ENSMUSG00000084128 | 0.118 | 0.355 | -1.498 | 0.033 | K14947 | Esrp2 | epithelial splicing regulatory protein 2 |
| ENSMUSG00000084145 | 0.914 | 0.304 | 1.677 | 0.005 |  | Gm12263 | ariadne homolog 2 pseudogene |
| ENSMUSG00000085287 | 2.306 | 1.559 | 0.658 | 0.047 |  | 4833418N02Rik | RIKEN cDNA 4833418N02 gene |
| ENSMUSG00000085336 | 3.335 | 1.945 | 0.870 | 0.004 |  | Gm11732 | predicted gene 11732 |
| ENSMUSG00000085337 | 0.307 | 0.059 | 2.475 | 0.002 |  | Gm15964 | predicted gene 15964 |
| ENSMUSG00000085412 | 0.335 | 1.521 | -2.098 | 0.042 |  | Halr1 | Hoxa adjacent long noncoding RNA 1 |
| ENSMUSG00000085632 | 1.052 | 0.549 | 1.032 | 0.011 |  | Gm12381 | predicted gene 12381 |
| ENSMUSG00000085642 | 1.139 | 0.654 | 0.897 | 0.042 |  | 3110053B16Rik | RIKEN cDNA 3110053B16 gene |
| ENSMUSG00000085719 | 1.014 | 0.286 | 1.914 | 0.001 |  | 4933424L21Rik | RIKEN cDNA 4933424L21 gene |
| ENSMUSG00000085793 | 6.344 | 4.666 | 0.537 | 0.000 | None | Lin52 | lin-52 homolog (C. elegans) |
| ENSMUSG00000086451 | 1.484 | 0.896 | 0.818 | 0.001 |  | 4933431K23Rik | RIKEN cDNA 4933431K23 gene |
| ENSMUSG00000086541 | 0.133 | 0.712 | -2.338 | 0.024 |  | Has2os | hyaluronan synthase 2, opposite strand |
| ENSMUSG00000086765 | 1.320 | 0.515 | 1.456 | 0.012 |  | Gm11827 | predicted gene 11827 |
| ENSMUSG00000087002 | 0.964 | 0.255 | 2.004 | 0.027 |  | Gm16277 | predicted gene 16277 |
| ENSMUSG00000087057 | 6.114 | 3.991 | 0.707 | 0.004 |  | Gm11730 | predicted gene 11730 |
| ENSMUSG00000087366 | 3.925 | 2.423 | 0.787 | 0.036 |  | Junos | jun proto-oncogene, opposite strand |
| ENSMUSG00000087500 | 1.918 | 5.931 | -1.531 | 0.014 |  | Gm12426 | predicted gene 12426 |
| ENSMUSG00000087579 | 3.626 | 2.683 | 0.529 | 0.010 |  | Hectd2os | Hectd2, opposite strand |
| ENSMUSG00000087684 | 0.697 | 0.123 | 2.606 | 0.000 |  | 1200007C13Rik | RIKEN cDNA 1200007C13 gene |
| ENSMUSG00000090125 | 0.231 | 0.069 | 1.843 | 0.049 |  | Pou3f1 | POU domain, class 3, transcription factor 1 |
| ENSMUSG00000090213 | 1.079 | 0.429 | 1.419 | 0.000 | K10704 | Tmem189 | transmembrane protein 189 |
| ENSMUSG00000090272 | 4.089 | 6.719 | -0.624 | 0.006 | None | Mndal | myeloid nuclear differentiation antigen like |
| ENSMUSG00000090799 | 5.729 | 8.787 | -0.525 | 0.002 | K13957 | Klhl33 | kelch-like 33 |
| ENSMUSG00000090942 | 0.675 | 2.071 | -1.534 | 0.031 | None | F830016B08Rik | RIKEN cDNA F830016B08 gene |
| ENSMUSG00000091119 | 6.700 | 3.425 | 1.060 | 0.000 | None | Ccdc152 | coiled-coil domain containing 152 |
| ENSMUSG00000091177 | 15.908 | 8.592 | 0.981 | 0.000 |  |  |  |
| ENSMUSG00000091514 | 0.474 | 0.151 | 1.740 | 0.011 |  | Gm17484 | predicted gene, 17484 |
| ENSMUSG00000091649 | 1.334 | 3.079 | -1.113 | 0.002 | None | Phf11b | PHD finger protein 11B |
| ENSMUSG00000091712 | 0.338 | 0.809 | -1.172 | 0.000 | None | Sec14l5 | SEC14-like lipid binding 5 |
| ENSMUSG00000091955 | 0.905 | 2.385 | -1.293 | 0.046 |  | Gm9844 | thymosin, beta 10 pseudogene |
| ENSMUSG00000092329 | 10.022 | 7.848 | 0.446 | 0.000 | K00710 |  |  |
| ENSMUSG00000092368 | 9.605 | 14.965 | -0.546 | 0.012 |  | A930015D03Rik | RIKEN cDNA A930015D03 gene |
| ENSMUSG00000092539 | 3.661 | 1.962 | 0.989 | 0.047 |  |  |  |
| ENSMUSG00000093577 | 1.906 | 0.998 | 1.026 | 0.000 |  |  |  |
| ENSMUSG00000093805 | 0.505 | 0.987 | -0.869 | 0.019 |  | Gal3st2b | galactose-3-O-sulfotransferase 2B |
| ENSMUSG00000094796 | 0.115 | 0.630 | -2.342 | 0.002 |  | BC147527 | cDNA sequence BC147527 |
| ENSMUSG00000095134 | 0.979 | 0.408 | 1.352 | 0.038 |  | Mid1-ps1 | midline 1, pseudogene 1 |
| ENSMUSG00000095253 | 0.248 | 0.124 | 1.091 | 0.020 |  | Zfp799 | zinc finger protein 799 |
| ENSMUSG00000095654 | 0.903 | 1.866 | -0.952 | 0.028 |  | Plscr5 | phospholipid scramblase family, member 5 |
| ENSMUSG00000095930 | 1.521 | 1.048 | 0.629 | 0.011 |  | Nim1k | NIM1 serine/threonine protein kinase |
| ENSMUSG00000096146 | 78.642 | 61.915 | 0.438 | 0.000 |  | Kcnj11 | potassium inwardly rectifying channel, subfamily J, member 11 |
| ENSMUSG00000096957 | 8.764 | 5.261 | 0.830 | 0.000 |  | E230013L22Rik | RIKEN cDNA E230013L22 gene |
| ENSMUSG00000097164 | 1.408 | 0.962 | 0.645 | 0.001 |  | Cep83os | centrosomal protein 83, opposite strand |
| ENSMUSG00000097518 | 2.326 | 1.609 | 0.625 | 0.026 |  |  |  |
| ENSMUSG00000097644 | 0.141 | 0.532 | -1.806 | 0.007 |  |  |  |
| ENSMUSG00000097652 | 3.941 | 7.800 | -0.894 | 0.040 |  | Mhrt | myosin heavy chain associated RNA transcript |
| ENSMUSG00000097675 | 0.845 | 0.410 | 1.141 | 0.029 |  | 1700101I11Rik | RIKEN cDNA 1700101I11 gene |
| ENSMUSG00000097877 | 0.513 | 0.222 | 1.297 | 0.018 |  | Gm26703 | predicted gene, 26703 |
| ENSMUSG00000098112 | 0.929 | 1.988 | -1.001 | 0.017 |  | Bin2 | bridging integrator 2 |
| ENSMUSG00000098132 | 0.619 | 1.426 | -1.110 | 0.004 |  | Rassf10 | Ras association (RalGDS/AF-6) domain family (N-terminal) member 10 |
| ENSMUSG00000100075 | 0.297 | 0.054 | 2.535 | 0.004 |  | 1700018L02Rik | RIKEN cDNA 1700018L02 gene |
| ENSMUSG00000100410 | 1.586 | 4.562 | -1.427 | 0.000 |  | 2310020H05Rik | RIKEN cDNA 2310020H05 gene |
| ENSMUSG00000100457 | 2.368 | 1.242 | 1.023 | 0.001 |  | D830032E09Rik | RIKEN cDNA D830032E09 gene |
| ENSMUSG00000100510 | 9.868 | 7.786 | 0.434 | 0.003 |  | AV026068 | expressed sequence AV026068 |
| ENSMUSG00000100599 | 2.093 | 1.228 | 0.861 | 0.044 |  | 1700120C14Rik | RIKEN cDNA 1700120C14 gene |
| ENSMUSG00000100658 | 0.110 | 0.324 | -1.463 | 0.031 |  | F730311O21Rik | RIKEN cDNA F730311O21 gene |
| ENSMUSG00000101086 | 0.996 | 1.723 | -0.699 | 0.016 |  | Gm28651 | predicted gene 28651 |
| ENSMUSG00000101111 | 403.459 | 241.593 | 0.828 | 0.001 |  |  |  |
| ENSMUSG00000101249 | 313.491 | 216.543 | 0.628 | 0.012 |  |  |  |
| ENSMUSG00000101941 | 0.263 | 2.250 | -2.990 | 0.000 |  | Gm28979 | predicted gene 28979 |
| ENSMUSG00000102070 | 215.707 | 130.597 | 0.816 | 0.000 |  |  |  |
| ENSMUSG00000102260 | 1.305 | 0.529 | 1.396 | 0.000 |  | D330025C20Rik | RIKEN cDNA D330025C20 gene |
| ENSMUSG00000102805 | 3.559 | 5.036 | -0.408 | 0.015 |  |  |  |
| ENSMUSG00000103156 | 2.444 | 1.036 | 1.327 | 0.021 |  | Gm38293 | predicted gene, 38293 |
| ENSMUSG00000103649 | 0.923 | 2.491 | -1.335 | 0.000 |  |  |  |
| ENSMUSG00000104263 | 0.418 | 0.177 | 1.328 | 0.011 |  | 9430062P05Rik | RIKEN cDNA 9430062P05 gene |
| ENSMUSG00000105168 | 2.719 | 5.451 | -0.910 | 0.012 |  | Gm30735 | predicted gene, 30735 |
| ENSMUSG00000105376 | 9.731 | 17.217 | -0.730 | 0.007 |  | Gm36535 | predicted gene, 36535 |
| ENSMUSG00000105449 | 0.016 | 0.074 | -2.079 | 0.042 |  |  |  |
| ENSMUSG00000105504 | 3.079 | 5.850 | -0.833 | 0.001 |  | Gbp5 | guanylate binding protein 5 |
| ENSMUSG00000105703 | 0.943 | 1.565 | -0.637 | 0.046 |  | Gm43305 | predicted gene 43305 |
| ENSMUSG00000105843 | 0.867 | 0.263 | 1.812 | 0.000 |  | Gm19439 | predicted gene, 19439 |
| ENSMUSG00000106140 | 1.797 | 0.502 | 1.922 | 0.001 |  | Gm42208 | predicted gene, 42208 |
| ENSMUSG00000106734 | 1.480 | 3.127 | -0.984 | 0.001 |  | Gm20559 | predicted gene, 20559 |
| ENSMUSG00000106795 | 9.766 | 6.048 | 0.786 | 0.000 |  |  |  |
| ENSMUSG00000107653 | 2.830 | 1.838 | 0.715 | 0.006 |  | Gm31520 | sterol-C5-desaturase pseudogene |
| ENSMUSG00000109008 | 25.806 | 18.821 | 0.547 | 0.005 |  |  |  |
| ENSMUSG00000109015 | 0.495 | 0.203 | 1.372 | 0.017 |  | Gm31024 | predicted gene, 31024 |
| ENSMUSG00000109695 | 2.659 | 1.664 | 0.769 | 0.029 |  | Gm31166 | predicted gene, 31166 |
| ENSMUSG00000109953 | 0.341 | 0.858 | -1.241 | 0.021 |  | 5430430B14Rik | RIKEN cDNA 5430430B14 gene |
| ENSMUSG00000110626 | 1.205 | 0.705 | 0.867 | 0.036 |  |  |  |
| ENSMUSG00000110649 | 5.882 | 3.215 | 0.968 | 0.006 |  | Gm40466 | predicted gene, 40466 |
| ENSMUSG00000111275 | 0.292 | 0.084 | 1.887 | 0.004 |  | Gm29824 | predicted gene, 29824 |
| ENSMUSG00000111815 | 2.950 | 1.961 | 0.685 | 0.046 |  | Gm6018 | MAD1 mitotic arrest deficient 1-like 1 pseudogene |
| ENSMUSG00000112129 | 3.522 | 1.680 | 1.159 | 0.000 |  | Pbld1 | phenazine biosynthesis-like protein domain containing 1 |
| ENSMUSG00000112327 | 3.534 | 7.402 | -0.974 | 0.014 |  | Gm36827 | predicted gene, 36827 |
| ENSMUSG00000112376 | 1.976 | 0.736 | 1.509 | 0.000 |  | Gm40649 | predicted gene, 40649 |
| ENSMUSG00000112831 | 9.091 | 6.106 | 0.664 | 0.041 |  |  |  |
| ENSMUSG00000113010 | 0.042 | 0.551 | -3.578 | 0.001 |  | Gm34084 | predicted gene, 34084 |
| ENSMUSG00000113047 | 1.000 | 0.522 | 1.029 | 0.033 |  |  |  |
| ENSMUSG00000113216 | 0.149 | 0.556 | -1.792 | 0.000 |  | Gm40841 | predicted gene, 40841 |
| ENSMUSG00000113737 | 0.684 | 0.158 | 2.219 | 0.000 |  | BB123696 | expressed sequence BB123696 |
| ENSMUSG00000113764 | 0.382 | 1.027 | -1.332 | 0.042 |  |  |  |
| ENSMUSG00000114069 | 8.373 | 4.842 | 0.882 | 0.000 |  | E130119H09Rik | RIKEN cDNA E130119H09 gene |
| ENSMUSG00000114898 | 7.339 | 5.973 | 0.389 | 0.023 |  |  |  |
| ENSMUSG00000116180 | 3.050 | 1.957 | 0.734 | 0.000 |  |  |  |
| ENSMUSG00000116604 | 0.640 | 0.338 | 1.023 | 0.042 |  |  |  |
| ENSMUSG00000116725 | 0.861 | 0.275 | 1.728 | 0.000 |  |  |  |
| ENSMUSG00000116851 | 0.119 | 0.300 | -1.245 | 0.013 |  |  |  |
| ENSMUSG00000116946 | 0.778 | 0.456 | 0.860 | 0.038 |  |  |  |
| ENSMUSG00000116953 | 1.019 | 0.377 | 1.515 | 0.047 |  |  |  |
| ENSMUSG00000117465 | 2.059 | 3.937 | -0.840 | 0.030 |  |  |  |
| ENSMUSG00000117604 | 5.916 | 1.956 | 1.686 | 0.000 |  |  |  |

**Table SII. RNA-seq identified 1393 genes that differentially expressed in SPM compared to DCM.**

| Gene_ID | SPM_FPKM | DCM_FPKM | logFC | FDR | KEGG_ID | symbol | ncbi_descritption |
| --- | --- | --- | --- | --- | --- | --- | --- |
| ENSMUSG00000000049 | 3.071 | 0.060 | 5.653 | 0.000 | K17305 | Apoh | apolipoprotein H |
| ENSMUSG00000000248 | 0.273 | 0.037 | 2.869 | 0.013 | None | Clec2g | C-type lectin domain family 2, member g |
| ENSMUSG00000000290 | 7.645 | 5.341 | 0.522 | 0.010 | K06464 | Itgb2 | integrin beta 2 |
| ENSMUSG00000000296 | 7.581 | 10.432 | -0.457 | 0.008 | None | Tpd52l1 | tumor protein D52-like 1 |
| ENSMUSG00000000305 | 0.873 | 1.447 | -0.727 | 0.005 | K06797 | Cdh4 | cadherin 4 |
| ENSMUSG00000000318 | 4.157 | 3.044 | 0.453 | 0.047 | K06721 | Clec10a | C-type lectin domain family 10, member A |
| ENSMUSG00000000378 | 14.912 | 19.632 | -0.393 | 0.001 | None | Ccm2 | cerebral cavernous malformation 2 |
| ENSMUSG00000000568 | 5.567 | 4.206 | 0.407 | 0.001 | K13044 | Hnrnpd | heterogeneous nuclear ribonucleoprotein D |
| ENSMUSG00000000581 | 10.744 | 8.183 | 0.396 | 0.010 | K12592 | C1d | C1D nuclear receptor co-repressor |
| ENSMUSG00000000600 | 4.223 | 3.230 | 0.389 | 0.016 | K17705 | Krit1 | KRIT1, ankyrin repeat containing |
| ENSMUSG00000000682 | 12.757 | 6.022 | 1.088 | 0.005 | K06488 | Cd52 | CD52 antigen |
| ENSMUSG00000000838 | 2.832 | 1.987 | 0.514 | 0.017 | K15516 | Fmr1 | fragile X mental retardation syndrome 1 |
| ENSMUSG00000001027 | 2.958 | 4.254 | -0.522 | 0.007 | K04837 | Scn4a | sodium channel, voltage-gated, type IV, alpha |
| ENSMUSG00000001158 | 20.941 | 14.256 | 0.558 | 0.001 | K12846 | Snrnp27 | small nuclear ribonucleoprotein 27 (U4/U6.U5) |
| ENSMUSG00000001444 | 0.000 | 0.076 | -5.035 | 0.023 | K10166 | Tbx21 | T-box 21 |
| ENSMUSG00000001508 | 72.299 | 101.201 | -0.484 | 0.000 | K12565 | Sgca | sarcoglycan, alpha (dystrophin-associated glycoprotein) |
| ENSMUSG00000001524 | 4.631 | 6.562 | -0.500 | 0.005 | K03144 | Gtf2h4 | general transcription factor II H, polypeptide 4 |
| ENSMUSG00000001552 | 113.827 | 162.398 | -0.512 | 0.000 | K10056 | Jup | junction plakoglobin |
| ENSMUSG00000001670 | 1.819 | 0.339 | 2.429 | 0.000 | K00815 | Tat | tyrosine aminotransferase |
| ENSMUSG00000001739 | 7.952 | 10.599 | -0.412 | 0.035 | K06087 | Cldn15 | claudin 15 |
| ENSMUSG00000001763 | 0.288 | 0.530 | -0.876 | 0.029 | K17346 | Tspan33 | tetraspanin 33 |
| ENSMUSG00000001802 | 3.380 | 5.250 | -0.633 | 0.000 | K20050 | Lrp3 | low density lipoprotein receptor-related protein 3 |
| ENSMUSG00000001833 | 14.983 | 10.060 | 0.579 | 0.002 | K16944 | 7-Sep | septin 7 |
| ENSMUSG00000001865 | 1.023 | 0.455 | 1.166 | 0.017 | K08780 | Cpa3 | carboxypeptidase A3, mast cell |
| ENSMUSG00000001891 | 49.141 | 37.744 | 0.383 | 0.000 | K00963 | Ugp2 | UDP-glucose pyrophosphorylase 2 |
| ENSMUSG00000002012 | 4.967 | 7.312 | -0.555 | 0.003 | K08795 | Pnck | pregnancy upregulated non-ubiquitously expressed CaM kinase |
| ENSMUSG00000002020 | 1.300 | 1.971 | -0.599 | 0.028 | K08023 | Ltbp2 | latent transforming growth factor beta binding protein 2 |
| ENSMUSG00000002058 | 5.227 | 7.150 | -0.449 | 0.016 | None | Unc119 | unc-119 lipid binding chaperone |
| ENSMUSG00000002107 | 6.602 | 4.584 | 0.529 | 0.000 | K13207 | Celf2 | CUGBP, Elav-like family member 2 |
| ENSMUSG00000002111 | 6.756 | 4.321 | 0.649 | 0.014 | K09438 | Spi1 | spleen focus forming virus (SFFV) proviral integration oncogene |
| ENSMUSG00000002228 | 6.057 | 8.080 | -0.414 | 0.013 | K17504 | Ppm1j | protein phosphatase 1J |
| ENSMUSG00000002289 | 11.617 | 22.377 | -0.941 | 0.006 | K08767 | Angptl4 | angiopoietin-like 4 |
| ENSMUSG00000002297 | 0.726 | 0.301 | 1.281 | 0.008 | K06629 | Dbf4 | DBF4 zinc finger |
| ENSMUSG00000002308 | 5.887 | 8.171 | -0.471 | 0.004 | K06734 | Cd320 | CD320 antigen |
| ENSMUSG00000002319 | 3.887 | 5.134 | -0.399 | 0.023 | K20221 | Ipo4 | importin 4 |
| ENSMUSG00000002325 | 8.920 | 6.868 | 0.380 | 0.003 | K04693 | Irf9 | interferon regulatory factor 9 |
| ENSMUSG00000002346 | 26.067 | 34.952 | -0.421 | 0.000 | K15085 | Slc25a42 | solute carrier family 25, member 42 |
| ENSMUSG00000002486 | 2.818 | 3.827 | -0.439 | 0.020 | K16811 | Tchp | trichoplein, keratin filament binding |
| ENSMUSG00000002578 | 0.133 | 0.326 | -1.294 | 0.008 | K09220 | Ikzf4 | IKAROS family zinc finger 4 |
| ENSMUSG00000002661 | 24.839 | 33.687 | -0.437 | 0.000 | K10769 | Alkbh7 | alkB homolog 7 |
| ENSMUSG00000002808 | 9.756 | 12.833 | -0.393 | 0.003 | None | Epdr1 | ependymin related protein 1 (zebrafish) |
| ENSMUSG00000002831 | 65.067 | 85.007 | -0.385 | 0.001 | K20254 | Plin4 | perilipin 4 |
| ENSMUSG00000002908 | 2.442 | 4.271 | -0.805 | 0.000 | K04942 | Kcnn1 | potassium intermediate/small conductance calcium-activated channel, subfamily N, member 1 |
| ENSMUSG00000003053 | 0.795 | 0.000 | 8.735 | 0.045 | K07413 | Cyp2c29 | cytochrome P450, family 2, subfamily c, polypeptide 29 |
| ENSMUSG00000003206 | 1.022 | 0.285 | 1.822 | 0.008 | None | Ebi3 | Epstein-Barr virus induced gene 3 |
| ENSMUSG00000003282 | 1.333 | 0.633 | 1.076 | 0.000 | K19484 | Plag1 | pleiomorphic adenoma gene 1 |
| ENSMUSG00000003283 | 2.935 | 1.562 | 0.911 | 0.004 | K08893 | Hck | hemopoietic cell kinase |
| ENSMUSG00000003299 | 35.039 | 46.033 | -0.392 | 0.001 | K02926 | Mrpl4 | mitochondrial ribosomal protein L4 |
| ENSMUSG00000003477 | 56.226 | 80.366 | -0.513 | 0.000 | K00562 | Inmt | indolethylamine N-methyltransferase |
| ENSMUSG00000003500 | 11.805 | 15.990 | -0.435 | 0.000 | K00088 | Impdh1 | inosine monophosphate dehydrogenase 1 |
| ENSMUSG00000003617 | 29.488 | 20.819 | 0.506 | 0.000 | K13624 | Cp | ceruloplasmin |
| ENSMUSG00000003762 | 1.489 | 2.230 | -0.579 | 0.024 | K08869 | Coq8b | coenzyme Q8B |
| ENSMUSG00000003809 | 21.966 | 29.780 | -0.437 | 0.000 | K00252 | Gcdh | glutaryl-Coenzyme A dehydrogenase |
| ENSMUSG00000003865 | 26.254 | 36.014 | -0.455 | 0.000 | K00693 | Gys1 | glycogen synthase 1, muscle |
| ENSMUSG00000003929 | 0.565 | 0.319 | 0.824 | 0.015 | K09228 | Zfp81 | zinc finger protein 81 |
| ENSMUSG00000004043 | 7.425 | 9.827 | -0.402 | 0.005 | K11223 | Stat5a | signal transducer and activator of transcription 5A |
| ENSMUSG00000004113 | 0.004 | 0.030 | -2.818 | 0.039 | K04849 | Cacna1b | calcium channel, voltage-dependent, N type, alpha 1B subunit |
| ENSMUSG00000004266 | 4.987 | 3.404 | 0.554 | 0.024 | K05697 | Ptpn6 | protein tyrosine phosphatase, non-receptor type 6 |
| ENSMUSG00000004270 | 26.026 | 34.590 | -0.408 | 0.000 | K13515 | Lpcat3 | lysophosphatidylcholine acyltransferase 3 |
| ENSMUSG00000004347 | 9.443 | 7.172 | 0.399 | 0.000 | K13755 | Pde1c | phosphodiesterase 1C |
| ENSMUSG00000004567 | 9.459 | 12.799 | -0.434 | 0.000 | K04992 | Mcoln1 | mucolipin 1 |
| ENSMUSG00000004609 | 1.957 | 1.150 | 0.770 | 0.001 | K06473 | Cd33 | CD33 antigen |
| ENSMUSG00000004655 | 231.661 | 330.351 | -0.512 | 0.000 | K09864 | Aqp1 | aquaporin 1 |
| ENSMUSG00000004698 | 3.252 | 2.313 | 0.494 | 0.002 | K11409 | Hdac9 | histone deacetylase 9 |
| ENSMUSG00000004709 | 0.314 | 0.110 | 1.510 | 0.011 | K06582 | Cd244 | CD244 natural killer cell receptor 2B4 |
| ENSMUSG00000004730 | 11.281 | 5.120 | 1.144 | 0.002 | K04591 | Adgre1 | adhesion G protein-coupled receptor E1 |
| ENSMUSG00000004929 | 6.046 | 8.889 | -0.553 | 0.001 | K01392 | Thop1 | thimet oligopeptidase 1 |
| ENSMUSG00000005373 | 4.551 | 6.041 | -0.406 | 0.009 | K09113 | Mlxipl | MLX interacting protein-like |
| ENSMUSG00000005656 | 19.531 | 14.651 | 0.418 | 0.000 | K17920 | Snx6 | sorting nexin 6 |
| ENSMUSG00000005681 | 8.714 | 1.065 | 3.036 | 0.019 | K08758 | Apoa2 | apolipoprotein A-II |
| ENSMUSG00000005800 | 0.591 | 0.083 | 2.845 | 0.000 | K01402 | Mmp8 | matrix metallopeptidase 8 |
| ENSMUSG00000005947 | 0.035 | 0.164 | -2.217 | 0.030 | K06524 | Itgae | integrin alpha E, epithelial-associated |
| ENSMUSG00000005968 | 3.203 | 1.947 | 0.723 | 0.016 | None | Tuft1 | tuftelin 1 |
| ENSMUSG00000006235 | 1.817 | 2.840 | -0.641 | 0.014 | K05079 | Epor | erythropoietin receptor |
| ENSMUSG00000006262 | 3.030 | 1.914 | 0.666 | 0.000 | K06685 | Mob1b | MOB kinase activator 1B |
| ENSMUSG00000006299 | 27.448 | 36.150 | -0.395 | 0.000 | None | Aamp | angio-associated migratory protein |
| ENSMUSG00000006403 | 0.362 | 0.681 | -0.913 | 0.013 | K07764 | Adamts4 | a disintegrin-like and metallopeptidase (reprolysin type) with thrombospondin type 1 motif, 4 |
| ENSMUSG00000006442 | 6.945 | 10.470 | -0.589 | 0.000 | K00797 | Srm | spermidine synthase |
| ENSMUSG00000006457 | 0.090 | 0.275 | -1.612 | 0.030 | K05699 | Actn3 | actinin alpha 3 |
| ENSMUSG00000006522 | 1.335 | 0.264 | 2.340 | 0.001 | None | Itih3 | inter-alpha trypsin inhibitor, heavy chain 3 |
| ENSMUSG00000006529 | 0.464 | 0.006 | 6.266 | 0.000 | K19014 | Itih1 | inter-alpha trypsin inhibitor, heavy chain 1 |
| ENSMUSG00000006542 | 0.396 | 0.755 | -0.929 | 0.039 | K07200 | Prkag3 | protein kinase, AMP-activated, gamma 3 non-catalytic subunit |
| ENSMUSG00000006586 | 1.018 | 0.663 | 0.623 | 0.014 | K10053 | Runx1t1 | runt-related transcription factor 1; translocated to, 1 (cyclin D-related) |
| ENSMUSG00000006651 | 1.281 | 2.047 | -0.674 | 0.017 | K05639 | Aplp1 | amyloid beta (A4) precursor-like protein 1 |
| ENSMUSG00000007030 | 2.790 | 4.017 | -0.526 | 0.019 | None | Vwa7 | von Willebrand factor A domain containing 7 |
| ENSMUSG00000007613 | 4.344 | 3.074 | 0.502 | 0.000 | K04674 | Tgfbr1 | transforming growth factor, beta receptor I |
| ENSMUSG00000007617 | 4.526 | 2.699 | 0.749 | 0.000 | K15010 | Homer1 | homer scaffolding protein 1 |
| ENSMUSG00000007850 | 20.618 | 15.731 | 0.393 | 0.001 | K12898 | Hnrnph1 | heterogeneous nuclear ribonucleoprotein H1 |
| ENSMUSG00000007987 | 2.441 | 3.460 | -0.500 | 0.013 | K07935 | Ift22 | intraflagellar transport 22 |
| ENSMUSG00000008090 | 9.168 | 12.095 | -0.398 | 0.014 | None | Fgfrl1 | fibroblast growth factor receptor-like 1 |
| ENSMUSG00000008333 | 7.223 | 5.107 | 0.503 | 0.012 | K11094 | Snrpb2 | U2 small nuclear ribonucleoprotein B |
| ENSMUSG00000008496 | 0.275 | 0.112 | 1.294 | 0.042 | K09364 | Pou2f2 | POU domain, class 2, transcription factor 2 |
| ENSMUSG00000008976 | 8.392 | 6.289 | 0.420 | 0.009 | K09441 | Gabpa | GA repeat binding protein, alpha |
| ENSMUSG00000009013 | 9.069 | 12.610 | -0.472 | 0.001 | K10418 | Dynll1 | dynein light chain LC8-type 1 |
| ENSMUSG00000009185 | 23.724 | 4.540 | 2.398 | 0.004 | None | Ccl8 | chemokine (C-C motif) ligand 8 |
| ENSMUSG00000009292 | 0.283 | 0.126 | 1.165 | 0.038 | K04977 | Trpm2 | transient receptor potential cation channel, subfamily M, member 2 |
| ENSMUSG00000009585 | 8.031 | 6.066 | 0.409 | 0.011 | K18750 | Apobec3 | apolipoprotein B mRNA editing enzyme, catalytic polypeptide 3 |
| ENSMUSG00000010080 | 3.892 | 5.179 | -0.409 | 0.003 | K12471 | Epn3 | epsin 3 |
| ENSMUSG00000010607 | 22.250 | 29.083 | -0.384 | 0.003 | K11001 | Pigyl | phosphatidylinositol glycan anchor biosynthesis, class Y-like |
| ENSMUSG00000010608 | 7.047 | 5.273 | 0.424 | 0.034 | K12822 | Rbm25 | RNA binding motif protein 25 |
| ENSMUSG00000010651 | 0.799 | 0.173 | 2.209 | 0.017 | K07513 | Acaa1b | acetyl-Coenzyme A acyltransferase 1B |
| ENSMUSG00000011305 | 111.149 | 154.567 | -0.475 | 0.000 | K20255 | Plin5 | perilipin 5 |
| ENSMUSG00000011658 | 2.219 | 3.077 | -0.469 | 0.029 | None | Fuz | fuzzy planar cell polarity protein |
| ENSMUSG00000011752 | 20.776 | 27.934 | -0.425 | 0.001 | K01834 | Pgam1 | phosphoglycerate mutase 1 |
| ENSMUSG00000011831 | 2.715 | 1.950 | 0.481 | 0.016 | K20242 | Evi5 | ecotropic viral integration site 5 |
| ENSMUSG00000012640 | 4.622 | 3.442 | 0.427 | 0.018 | K09228 | Zfp715 | zinc finger protein 715 |
| ENSMUSG00000013150 | 1.107 | 1.541 | -0.472 | 0.049 | None | Gfod2 | glucose-fructose oxidoreductase domain containing 2 |
| ENSMUSG00000013275 | 31.232 | 42.090 | -0.429 | 0.000 | K15122 | Slc41a1 | solute carrier family 41, member 1 |
| ENSMUSG00000013662 | 29.522 | 21.425 | 0.464 | 0.000 | None | Atad1 | ATPase family, AAA domain containing 1 |
| ENSMUSG00000013736 | 10.550 | 8.005 | 0.401 | 0.019 | K00974 | Trnt1 | tRNA nucleotidyl transferase, CCA-adding, 1 |
| ENSMUSG00000013833 | 18.205 | 24.300 | -0.416 | 0.001 | K15159 | Med16 | mediator complex subunit 16 |
| ENSMUSG00000013974 | 0.472 | 0.113 | 2.068 | 0.038 | None | Mcemp1 | mast cell expressed membrane protein 1 |
| ENSMUSG00000014226 | 13.686 | 10.370 | 0.403 | 0.025 | K04507 | Cacybp | calcyclin binding protein |
| ENSMUSG00000014503 | 6.732 | 5.089 | 0.406 | 0.026 | K04991 | Pkd2l2 | polycystic kidney disease 2-like 2 |
| ENSMUSG00000014846 | 17.316 | 23.026 | -0.409 | 0.003 | None | Tppp3 | tubulin polymerization-promoting protein family member 3 |
| ENSMUSG00000014956 | 44.056 | 31.498 | 0.487 | 0.000 | K06269 | Ppp1cb | protein phosphatase 1, catalytic subunit, beta isoform |
| ENSMUSG00000015085 | 6.163 | 8.754 | -0.504 | 0.002 | K01509 | Entpd2 | ectonucleoside triphosphate diphosphohydrolase 2 |
| ENSMUSG00000015247 | 5.441 | 4.092 | 0.415 | 0.038 | None | Nipsnap3b | nipsnap homolog 3B |
| ENSMUSG00000015312 | 20.309 | 12.267 | 0.731 | 0.008 | K04402 | Gadd45b | growth arrest and DNA-damage-inducible 45 beta |
| ENSMUSG00000015337 | 43.003 | 56.916 | -0.402 | 0.000 | K01173 | Endog | endonuclease G |
| ENSMUSG00000015340 | 6.099 | 3.375 | 0.858 | 0.000 | K08008 | Cybb | cytochrome b-245, beta polypeptide |
| ENSMUSG00000015355 | 5.006 | 2.566 | 0.967 | 0.001 | K06479 | Cd48 | CD48 antigen |
| ENSMUSG00000015396 | 2.881 | 4.559 | -0.660 | 0.002 | K06510 | Cd83 | CD83 antigen |
| ENSMUSG00000015437 | 0.879 | 0.144 | 2.584 | 0.005 | K01353 | Gzmb | granzyme B |
| ENSMUSG00000015451 | 0.177 | 0.043 | 2.058 | 0.022 |  | C4a | complement component 4A (Rodgers blood group) |
| ENSMUSG00000015468 | 8.780 | 11.598 | -0.400 | 0.000 | K02599 | Notch4 | notch 4 |
| ENSMUSG00000015672 | 6.212 | 4.400 | 0.501 | 0.011 | K02911 | Mrpl32 | mitochondrial ribosomal protein L32 |
| ENSMUSG00000015733 | 35.512 | 26.035 | 0.451 | 0.000 | K10364 | Capza2 | capping protein (actin filament) muscle Z-line, alpha 2 |
| ENSMUSG00000015749 | 25.260 | 17.370 | 0.543 | 0.000 | K18648 | Anp32e | acidic (leucine-rich) nuclear phosphoprotein 32 family, member E |
| ENSMUSG00000015757 | 4.832 | 3.478 | 0.478 | 0.014 | K12735 | Ppil4 | peptidylprolyl isomerase (cyclophilin)-like 4 |
| ENSMUSG00000015837 | 264.172 | 373.624 | -0.499 | 0.000 | K14381 | Sqstm1 | sequestosome 1 |
| ENSMUSG00000015854 | 0.842 | 0.133 | 2.646 | 0.001 | None | Cd5l | CD5 antigen-like |
| ENSMUSG00000015943 | 20.347 | 28.236 | -0.469 | 0.001 | K05527 | Bola1 | bolA-like 1 (E. coli) |
| ENSMUSG00000015957 | 4.260 | 6.130 | -0.522 | 0.000 | K01384 | Wnt11 | wingless-type MMTV integration site family, member 11 |
| ENSMUSG00000015968 | 0.552 | 0.368 | 0.589 | 0.013 | K04851 | Cacna1d | calcium channel, voltage-dependent, L type, alpha 1D subunit |
| ENSMUSG00000016028 | 1.448 | 1.966 | -0.439 | 0.014 | K04600 | Celsr1 | cadherin, EGF LAG seven-pass G-type receptor 1 |
| ENSMUSG00000016252 | 148.311 | 193.565 | -0.382 | 0.000 | K02135 | Atp5e | ATP synthase, H+ transporting, mitochondrial F1 complex, epsilon subunit |
| ENSMUSG00000016559 | 91.402 | 64.984 | 0.496 | 0.000 | K11253 | H3f3b | H3 histone, family 3B |
| ENSMUSG00000016637 | 5.930 | 8.015 | -0.431 | 0.025 | K07934 | Ift27 | intraflagellar transport 27 |
| ENSMUSG00000016763 | 0.576 | 0.952 | -0.725 | 0.022 | None | Scube1 | signal peptide, CUB domain, EGF-like 1 |
| ENSMUSG00000016942 | 0.422 | 0.104 | 2.029 | 0.046 | K09637 | Tmprss6 | transmembrane serine protease 6 |
| ENSMUSG00000016984 | 0.588 | 0.314 | 0.910 | 0.022 | None | Etaa1 | Ewing tumor-associated antigen 1 |
| ENSMUSG00000017057 | 12.029 | 8.554 | 0.494 | 0.000 | K05076 | Il13ra1 | interleukin 13 receptor, alpha 1 |
| ENSMUSG00000017314 | 1.824 | 2.838 | -0.635 | 0.001 | None | Mpp2 | membrane protein, palmitoylated 2 (MAGUK p55 subfamily member 2) |
| ENSMUSG00000017386 | 2.284 | 3.188 | -0.479 | 0.048 | K09848 | Traf4 | TNF receptor associated factor 4 |
| ENSMUSG00000017485 | 3.967 | 2.684 | 0.569 | 0.007 | K03164 | Top2b | topoisomerase (DNA) II beta |
| ENSMUSG00000017548 | 3.080 | 1.902 | 0.699 | 0.002 | K11463 | Suz12 | SUZ12 polycomb repressive complex 2 subunit |
| ENSMUSG00000017713 | 2.311 | 3.558 | -0.620 | 0.001 | K01620 | Tha1 | threonine aldolase 1 |
| ENSMUSG00000017950 | 0.177 | 0.000 | 7.194 | 0.000 | K07292 | Hnf4a | hepatic nuclear factor 4, alpha |
| ENSMUSG00000018199 | 1.679 | 1.178 | 0.515 | 0.026 | K11089 | Trove2 | TROVE domain family, member 2 |
| ENSMUSG00000018509 | 8.013 | 10.947 | -0.446 | 0.038 | None | Cenpv | centromere protein V |
| ENSMUSG00000018572 | 8.656 | 11.449 | -0.400 | 0.004 | None | Phf23 | PHD finger protein 23 |
| ENSMUSG00000018599 | 9.799 | 13.083 | -0.415 | 0.004 | None | Mief2 | mitochondrial elongation factor 2 |
| ENSMUSG00000018654 | 1.174 | 0.716 | 0.717 | 0.035 | K09220 | Ikzf1 | IKAROS family zinc finger 1 |
| ENSMUSG00000018821 | 9.036 | 12.069 | -0.414 | 0.021 | None | Avpi1 | arginine vasopressin-induced 1 |
| ENSMUSG00000018861 | 4.286 | 5.750 | -0.421 | 0.025 | K18914 | Fdxr | ferredoxin reductase |
| ENSMUSG00000018924 | 0.233 | 0.019 | 3.580 | 0.005 | K00460 | Alox15 | arachidonate 15-lipoxygenase |
| ENSMUSG00000018932 | 31.833 | 42.945 | -0.430 | 0.000 | K04432 | Map2k3 | mitogen-activated protein kinase kinase 3 |
| ENSMUSG00000019039 | 13.847 | 18.091 | -0.383 | 0.003 | None | Dalrd3 | DALR anticodon binding domain containing 3 |
| ENSMUSG00000019066 | 11.051 | 15.308 | -0.468 | 0.001 | K07884 | Rab3d | RAB3D, member RAS oncogene family |
| ENSMUSG00000019082 | 9.845 | 13.244 | -0.425 | 0.000 | K15107 | Slc25a22 | solute carrier family 25 (mitochondrial carrier, glutamate), member 22 |
| ENSMUSG00000019122 | 5.923 | 4.080 | 0.543 | 0.026 | K05510 | Ccl9 | chemokine (C-C motif) ligand 9 |
| ENSMUSG00000019139 | 16.281 | 22.766 | -0.481 | 0.000 | K01858 | Isyna1 | myo-inositol 1-phosphate synthase A1 |
| ENSMUSG00000019256 | 3.162 | 2.096 | 0.596 | 0.000 | K09093 | Ahr | aryl-hydrocarbon receptor |
| ENSMUSG00000019505 | 373.867 | 500.943 | -0.420 | 0.000 | K08770 | Ubb | ubiquitin B |
| ENSMUSG00000019577 | 463.931 | 627.105 | -0.433 | 0.002 | K00898 | Pdk4 | pyruvate dehydrogenase kinase, isoenzyme 4 |
| ENSMUSG00000019787 | 28.378 | 16.946 | 0.752 | 0.032 | None | Trdn | triadin |
| ENSMUSG00000019792 | 2.024 | 1.313 | 0.626 | 0.024 | K15430 | Trmt11 | tRNA methyltransferase 11 |
| ENSMUSG00000019802 | 10.080 | 7.052 | 0.519 | 0.000 | K09540 | Sec63 | SEC63-like (S. cerevisiae) |
| ENSMUSG00000019810 | 36.146 | 27.314 | 0.406 | 0.000 | K01206 | Fuca2 | fucosidase, alpha-L- 2, plasma |
| ENSMUSG00000019817 | 3.559 | 2.156 | 0.727 | 0.002 | K19485 | Plagl1 | pleiomorphic adenoma gene-like 1 |
| ENSMUSG00000019897 | 3.460 | 2.626 | 0.401 | 0.015 | None | Ccdc59 | coiled-coil domain containing 59 |
| ENSMUSG00000019907 | 10.933 | 7.979 | 0.459 | 0.004 | K06270 | Ppp1r12a | protein phosphatase 1, regulatory (inhibitor) subunit 12A |
| ENSMUSG00000019920 | 19.446 | 14.948 | 0.384 | 0.007 | None | Lims1 | LIM and senescent cell antigen-like domains 1 |
| ENSMUSG00000019966 | 9.301 | 7.059 | 0.404 | 0.030 | K05461 | Kitl | kit ligand |
| ENSMUSG00000019987 | 1.502 | 0.093 | 4.021 | 0.010 | K01476 | Arg1 | arginase, liver |
| ENSMUSG00000020023 | 0.740 | 1.069 | -0.527 | 0.033 | None | Tmcc3 | transmembrane and coiled coil domains 3 |
| ENSMUSG00000020027 | 8.484 | 6.020 | 0.498 | 0.004 | K04695 | Socs2 | suppressor of cytokine signaling 2 |
| ENSMUSG00000020042 | 0.613 | 0.955 | -0.636 | 0.017 | K10483 | Btbd11 | BTB (POZ) domain containing 11 |
| ENSMUSG00000020048 | 30.919 | 18.105 | 0.776 | 0.000 | K09487 | Hsp90b1 | heat shock protein 90, beta (Grp94), member 1 |
| ENSMUSG00000020070 | 2.267 | 1.518 | 0.582 | 0.003 | None | Rufy2 | RUN and FYVE domain-containing 2 |
| ENSMUSG00000020076 | 10.230 | 7.610 | 0.429 | 0.001 | K13183 | Ddx50 | DEAD (Asp-Glu-Ala-Asp) box polypeptide 50 |
| ENSMUSG00000020078 | 24.623 | 18.141 | 0.444 | 0.000 | K18466 | Vps26a | VPS26 retromer complex component A |
| ENSMUSG00000020102 | 0.780 | 0.532 | 0.553 | 0.005 | K08184 | Slc16a7 | solute carrier family 16 (monocarboxylic acid transporters), member 7 |
| ENSMUSG00000020120 | 3.172 | 1.693 | 0.909 | 0.000 | K19993 | Plek | pleckstrin |
| ENSMUSG00000020133 | 15.911 | 21.424 | -0.426 | 0.000 | None | 2310011J03Rik | RIKEN cDNA 2310011J03 gene |
| ENSMUSG00000020135 | 0.984 | 1.328 | -0.430 | 0.033 | K02085 | Apc2 | adenomatosis polyposis coli 2 |
| ENSMUSG00000020143 | 1.628 | 1.091 | 0.579 | 0.002 | K12367 | Dock2 | dedicator of cyto-kinesis 2 |
| ENSMUSG00000020152 | 17.337 | 13.337 | 0.382 | 0.002 | K17260 | Actr2 | ARP2 actin-related protein 2 |
| ENSMUSG00000020163 | 359.236 | 477.183 | -0.407 | 0.000 | K00420 | Uqcr11 | ubiquinol-cytochrome c reductase, complex III subunit XI |
| ENSMUSG00000020181 | 1.292 | 0.952 | 0.441 | 0.037 | None | Nav3 | neuron navigator 3 |
| ENSMUSG00000020182 | 0.751 | 1.406 | -0.897 | 0.020 | K01593 | Ddc | dopa decarboxylase |
| ENSMUSG00000020189 | 8.200 | 5.538 | 0.571 | 0.000 | K20464 | Osbpl8 | oxysterol binding protein-like 8 |
| ENSMUSG00000020218 | 4.369 | 6.526 | -0.575 | 0.002 | K01691 | Wif1 | Wnt inhibitory factor 1 |
| ENSMUSG00000020264 | 5.565 | 7.536 | -0.434 | 0.002 | K14209 | Slc36a2 | solute carrier family 36 (proton/amino acid symporter), member 2 |
| ENSMUSG00000020300 | 7.949 | 6.043 | 0.400 | 0.034 | K02602 | Cpeb4 | cytoplasmic polyadenylation element binding protein 4 |
| ENSMUSG00000020331 | 5.827 | 8.502 | -0.543 | 0.000 | K04955 | Hcn2 | hyperpolarization-activated, cyclic nucleotide-gated K+ 2 |
| ENSMUSG00000020333 | 1.254 | 1.801 | -0.522 | 0.038 | K01897 | Acsl6 | acyl-CoA synthetase long-chain family member 6 |
| ENSMUSG00000020354 | 22.337 | 17.045 | 0.394 | 0.015 | K12563 | Sgcd | sarcoglycan, delta (dystrophin-associated glycoprotein) |
| ENSMUSG00000020437 | 1.423 | 0.907 | 0.653 | 0.029 | K10356 | Myo1g | myosin IG |
| ENSMUSG00000020463 | 3.041 | 2.110 | 0.532 | 0.035 | K17491 | Ppp4r3b | protein phosphatase 4 regulatory subunit 3B |
| ENSMUSG00000020471 | 16.405 | 24.120 | -0.554 | 0.000 | K02328 | Pold2 | polymerase (DNA directed), delta 2, regulatory subunit |
| ENSMUSG00000020477 | 81.209 | 107.191 | -0.398 | 0.000 | K17403 | Mrps24 | mitochondrial ribosomal protein S24 |
| ENSMUSG00000020486 | 20.086 | 26.244 | -0.383 | 0.015 | K16943 | 4-Sep | septin 4 |
| ENSMUSG00000020592 | 3.598 | 4.736 | -0.394 | 0.010 | K06257 | Sdc1 | syndecan 1 |
| ENSMUSG00000020608 | 2.935 | 1.969 | 0.581 | 0.020 | None | Smc6 | structural maintenance of chromosomes 6 |
| ENSMUSG00000020609 | 0.497 | 0.000 | 10.363 | 0.008 | K14462 | Apob | apolipoprotein B |
| ENSMUSG00000020620 | 2.628 | 1.884 | 0.482 | 0.007 | K05650 | Abca8b | ATP-binding cassette, sub-family A (ABC1), member 8b |
| ENSMUSG00000020630 | 3.809 | 5.425 | -0.507 | 0.002 | K03469 | Rnaseh1 | ribonuclease H1 |
| ENSMUSG00000020644 | 10.295 | 7.071 | 0.547 | 0.011 | K17693 | Id2 | inhibitor of DNA binding 2 |
| ENSMUSG00000020648 | 4.363 | 2.980 | 0.553 | 0.007 | K05545 | Dus4l | dihydrouridine synthase 4-like (S. cerevisiae) |
| ENSMUSG00000020649 | 0.670 | 1.161 | -0.793 | 0.021 | K10808 | Rrm2 | ribonucleotide reductase M2 |
| ENSMUSG00000020650 | 4.008 | 2.458 | 0.708 | 0.000 | None | Bcap29 | B cell receptor associated protein 29 |
| ENSMUSG00000020677 | 5.663 | 4.282 | 0.406 | 0.005 | K14779 | Ddx52 | DEAD (Asp-Glu-Ala-Asp) box polypeptide 52 |
| ENSMUSG00000020687 | 3.267 | 2.432 | 0.428 | 0.007 | K03350 | Cdc27 | cell division cycle 27 |
| ENSMUSG00000020743 | 18.677 | 24.346 | -0.380 | 0.001 | None | Mif4gd | MIF4G domain containing |
| ENSMUSG00000020782 | 6.184 | 9.084 | -0.553 | 0.000 | K06094 | Llgl2 | LLGL2 scribble cell polarity complex component |
| ENSMUSG00000020802 | 7.860 | 11.096 | -0.496 | 0.000 | K10581 | Ube2o | ubiquitin-conjugating enzyme E2O |
| ENSMUSG00000020817 | 5.801 | 4.321 | 0.428 | 0.018 | K12480 | Rabep1 | rabaptin, RAB GTPase binding effector protein 1 |
| ENSMUSG00000020829 | 1.209 | 2.081 | -0.781 | 0.005 | K14613 | Slc46a1 | solute carrier family 46, member 1 |
| ENSMUSG00000020832 | 9.052 | 12.826 | -0.500 | 0.000 | None | Eral1 | Era (G-protein)-like 1 (E. coli) |
| ENSMUSG00000020844 | 9.377 | 12.587 | -0.422 | 0.003 | K17609 | Nxn | nucleoredoxin |
| ENSMUSG00000020882 | 1.976 | 3.351 | -0.759 | 0.000 | K04862 | Cacnb1 | calcium channel, voltage-dependent, beta 1 subunit |
| ENSMUSG00000020926 | 3.765 | 5.149 | -0.450 | 0.001 | K16067 | Adam11 | a disintegrin and metallopeptidase domain 11 |
| ENSMUSG00000020952 | 2.236 | 1.697 | 0.401 | 0.037 | K19998 | Scfd1 | Sec1 family domain containing 1 |
| ENSMUSG00000021000 | 10.021 | 7.494 | 0.423 | 0.006 | None |  |  |
| ENSMUSG00000021027 | 7.609 | 5.861 | 0.379 | 0.003 | None | Ralgapa1 | Ral GTPase activating protein, alpha subunit 1 |
| ENSMUSG00000021028 | 5.428 | 3.502 | 0.634 | 0.008 | None | Mbip | MAP3K12 binding inhibitory protein 1 |
| ENSMUSG00000021033 | 11.116 | 14.767 | -0.407 | 0.000 | K01800 | Gstz1 | glutathione transferase zeta 1 (maleylacetoacetate isomerase) |
| ENSMUSG00000021072 | 20.972 | 13.076 | 0.684 | 0.000 | None | Tmx1 | thioredoxin-related transmembrane protein 1 |
| ENSMUSG00000021091 | 34.390 | 15.830 | 1.123 | 0.012 | K04525 | Serpina3n | serine (or cysteine) peptidase inhibitor, clade A, member 3N |
| ENSMUSG00000021112 | 3.350 | 2.257 | 0.574 | 0.009 | K06091 | Mpp5 | membrane protein, palmitoylated 5 (MAGUK p55 subfamily member 5) |
| ENSMUSG00000021135 | 0.703 | 0.025 | 4.770 | 0.000 | K14341 | Slc10a1 | solute carrier family 10 (sodium/bile acid cotransporter family), member 1 |
| ENSMUSG00000021139 | 0.194 | 0.028 | 2.759 | 0.026 | K20057 | Gm20498 | predicted gene 20498 |
| ENSMUSG00000021188 | 3.044 | 1.956 | 0.641 | 0.000 | None | Trip11 | thyroid hormone receptor interactor 11 |
| ENSMUSG00000021219 | 3.315 | 2.521 | 0.398 | 0.023 | K16449 | Rgs6 | regulator of G-protein signaling 6 |
| ENSMUSG00000021235 | 20.682 | 26.971 | -0.382 | 0.005 | K06126 | Coq6 | coenzyme Q6 monooxygenase |
| ENSMUSG00000021257 | 2.131 | 2.900 | -0.443 | 0.037 | K18729 | Angel1 | angel homolog 1 |
| ENSMUSG00000021270 | 22.345 | 15.764 | 0.507 | 0.003 | K04079 | Hsp90aa1 | heat shock protein 90, alpha (cytosolic), class A member 1 |
| ENSMUSG00000021273 | 23.150 | 30.673 | -0.403 | 0.000 | K00801 | Fdft1 | farnesyl diphosphate farnesyl transferase 1 |
| ENSMUSG00000021280 | 1.467 | 2.275 | -0.633 | 0.024 | None | Exoc3l4 | exocyst complex component 3-like 4 |
| ENSMUSG00000021282 | 29.095 | 18.379 | 0.666 | 0.000 | K03262 | Eif5 | eukaryotic translation initiation factor 5 |
| ENSMUSG00000021294 | 6.329 | 9.143 | -0.528 | 0.000 | K10404 | Kif26a | kinesin family member 26A |
| ENSMUSG00000021364 | 0.161 | 0.013 | 3.577 | 0.035 | K10205 | Elovl2 | elongation of very long chain fatty acids (FEN1/Elo2, SUR4/Elo3, yeast)-like 2 |
| ENSMUSG00000021367 | 3.381 | 1.895 | 0.839 | 0.007 | K16366 | Edn1 | endothelin 1 |
| ENSMUSG00000021392 | 1.572 | 0.994 | 0.664 | 0.037 | None | Nol8 | nucleolar protein 8 |
| ENSMUSG00000021411 | 9.454 | 12.351 | -0.383 | 0.002 | None | Pxdc1 | PX domain containing 1 |
| ENSMUSG00000021423 | 2.557 | 1.535 | 0.740 | 0.019 | None | Ly86 | lymphocyte antigen 86 |
| ENSMUSG00000021428 | 4.215 | 3.239 | 0.383 | 0.048 | K07178 | Riok1 | RIO kinase 1 |
| ENSMUSG00000021451 | 14.987 | 19.581 | -0.384 | 0.000 | K06521 | Sema4d | sema domain, immunoglobulin domain (Ig), transmembrane domain (TM) and short cytoplasmic domain, (semaphorin) 4D |
| ENSMUSG00000021470 | 1.953 | 1.488 | 0.395 | 0.029 | K20098 | Ercc6l2 | excision repair cross-complementing rodent repair deficiency, complementation group 6 like 2 |
| ENSMUSG00000021492 | 0.216 | 0.000 | 6.334 | 0.002 | K01328 | F12 | coagulation factor XII (Hageman factor) |
| ENSMUSG00000021508 | 8.690 | 12.869 | -0.565 | 0.003 | K10033 | Cxcl14 | chemokine (C-X-C motif) ligand 14 |
| ENSMUSG00000021519 | 6.938 | 5.101 | 0.446 | 0.022 | K15032 | Mterf3 | mitochondrial transcription termination factor 3 |
| ENSMUSG00000021583 | 6.453 | 4.768 | 0.439 | 0.001 | K09604 | Erap1 | endoplasmic reticulum aminopeptidase 1 |
| ENSMUSG00000021615 | 1.751 | 1.179 | 0.571 | 0.016 | K10886 | Xrcc4 | X-ray repair complementing defective repair in Chinese hamster cells 4 |
| ENSMUSG00000021676 | 1.440 | 0.977 | 0.563 | 0.017 | K05767 | Iqgap2 | IQ motif containing GTPase activating protein 2 |
| ENSMUSG00000021716 | 6.204 | 4.594 | 0.437 | 0.010 | None | Srek1ip1 | splicing regulatory glutamine/lysine-rich protein 1interacting protein 1 |
| ENSMUSG00000021728 | 2.594 | 1.761 | 0.561 | 0.023 | None | Emb | embigin |
| ENSMUSG00000021775 | 18.263 | 11.440 | 0.678 | 0.000 | K08531 | Nr1d2 | nuclear receptor subfamily 1, group D, member 2 |
| ENSMUSG00000021796 | 13.086 | 9.322 | 0.492 | 0.000 | K04673 | Bmpr1a | bone morphogenetic protein receptor, type 1A |
| ENSMUSG00000021866 | 9.278 | 12.185 | -0.391 | 0.012 | K17095 | Anxa11 | annexin A11 |
| ENSMUSG00000021870 | 22.301 | 15.903 | 0.491 | 0.000 | None | Slmap | sarcolemma associated protein |
| ENSMUSG00000021913 | 60.149 | 81.315 | -0.435 | 0.000 | K00164 | Ogdhl | oxoglutarate dehydrogenase-like |
| ENSMUSG00000021936 | 4.132 | 3.108 | 0.413 | 0.008 | K04440 | Mapk8 | mitogen-activated protein kinase 8 |
| ENSMUSG00000021952 | 3.193 | 2.386 | 0.422 | 0.007 | None | Xpo4 | exportin 4 |
| ENSMUSG00000021967 | 8.603 | 11.720 | -0.444 | 0.001 | None | Mrpl57 | mitochondrial ribosomal protein L57 |
| ENSMUSG00000021969 | 2.805 | 2.078 | 0.436 | 0.034 | K20028 | Zdhhc20 | zinc finger, DHHC domain containing 20 |
| ENSMUSG00000021982 | 5.100 | 3.797 | 0.428 | 0.008 | None | Cdadc1 | cytidine and dCMP deaminase domain containing 1 |
| ENSMUSG00000021998 | 13.943 | 7.787 | 0.845 | 0.000 | K17276 | Lcp1 | lymphocyte cytosolic protein 1 |
| ENSMUSG00000021999 | 0.434 | 0.032 | 3.751 | 0.007 | K01300 | Cpb2 | carboxypeptidase B2 (plasma) |
| ENSMUSG00000022014 | 2.127 | 1.249 | 0.773 | 0.026 | None | Epsti1 | epithelial stromal interaction 1 (breast) |
| ENSMUSG00000022023 | 4.492 | 3.088 | 0.543 | 0.001 | K13220 | Wbp4 | WW domain binding protein 4 |
| ENSMUSG00000022096 | 1.877 | 2.674 | -0.508 | 0.046 | K00478 | Hr | hairless |
| ENSMUSG00000022141 | 3.434 | 2.586 | 0.414 | 0.045 | K06672 | Nipbl | NIPBL cohesin loading factor |
| ENSMUSG00000022148 | 1.564 | 0.466 | 1.748 | 0.000 | K17698 | Fyb | FYN binding protein |
| ENSMUSG00000022181 | 0.536 | 0.115 | 2.218 | 0.009 | K03995 | C6 | complement component 6 |
| ENSMUSG00000022205 | 8.229 | 5.755 | 0.518 | 0.000 | None | Sub1 | SUB1 homolog (S. cerevisiae) |
| ENSMUSG00000022215 | 43.039 | 58.830 | -0.448 | 0.000 | None | Fitm1 | fat storage-inducing transmembrane protein 1 |
| ENSMUSG00000022217 | 15.434 | 20.212 | -0.386 | 0.000 | None | Emc9 | ER membrane protein complex subunit 9 |
| ENSMUSG00000022231 | 2.463 | 3.234 | -0.391 | 0.012 | K06841 | Sema5a | sema domain, seven thrombospondin repeats (type 1 and type 1-like), transmembrane domain (TM) and short cytoplasmic domain, (semaphorin) 5A |
| ENSMUSG00000022255 | 11.885 | 8.995 | 0.405 | 0.001 | None | Mtdh | metadherin |
| ENSMUSG00000022270 | 164.703 | 109.443 | 0.592 | 0.000 | None | Retreg1 | reticulophagy regulator 1 |
| ENSMUSG00000022292 | 6.242 | 4.682 | 0.419 | 0.040 | K10808 | Rrm2b | ribonucleotide reductase M2 B (TP53 inducible) |
| ENSMUSG00000022305 | 1.909 | 1.411 | 0.437 | 0.039 | K20050 | Lrp12 | low density lipoprotein-related protein 12 |
| ENSMUSG00000022307 | 13.725 | 9.937 | 0.469 | 0.000 | None | Oxr1 | oxidation resistance 1 |
| ENSMUSG00000022309 | 6.722 | 3.834 | 0.813 | 0.001 | K05465 | Angpt1 | angiopoietin 1 |
| ENSMUSG00000022329 | 1.955 | 1.213 | 0.692 | 0.039 | K04412 | Stk3 | serine/threonine kinase 3 |
| ENSMUSG00000022336 | 28.673 | 21.597 | 0.412 | 0.000 | K03250 | Eif3e | eukaryotic translation initiation factor 3, subunit E |
| ENSMUSG00000022378 | 4.508 | 3.286 | 0.459 | 0.006 | None | Fam49b | family with sequence similarity 49, member B |
| ENSMUSG00000022464 | 3.772 | 2.895 | 0.385 | 0.019 | K14991 | Slc38a4 | solute carrier family 38, member 4 |
| ENSMUSG00000022474 | 18.600 | 25.149 | -0.432 | 0.002 | K17497 | Pmm1 | phosphomannomutase 1 |
| ENSMUSG00000022488 | 3.780 | 2.557 | 0.567 | 0.001 | K05750 | Nckap1l | NCK associated protein 1 like |
| ENSMUSG00000022516 | 5.008 | 6.980 | -0.476 | 0.010 | K16867 | Nudt16l1 | nudix (nucleoside diphosphate linked moiety X)-type motif 16-like 1 |
| ENSMUSG00000022523 | 1.679 | 0.600 | 1.486 | 0.000 | K04358 | Fgf12 | fibroblast growth factor 12 |
| ENSMUSG00000022533 | 5.577 | 4.263 | 0.391 | 0.011 | K14951 | Atp13a3 | ATPase type 13A3 |
| ENSMUSG00000022543 | 0.345 | 0.926 | -1.423 | 0.003 | None | 4930451G09Rik | RIKEN cDNA 4930451G09 gene |
| ENSMUSG00000022546 | 9.551 | 14.772 | -0.627 | 0.000 | K00814 | Gpt | glutamic pyruvic transaminase, soluble |
| ENSMUSG00000022562 | 18.354 | 24.934 | -0.442 | 0.000 | K01469 | Oplah | 5-oxoprolinase (ATP-hydrolysing) |
| ENSMUSG00000022568 | 6.201 | 8.680 | -0.483 | 0.000 | K16175 | Scrib | scribbled planar cell polarity |
| ENSMUSG00000022579 | 90.591 | 118.117 | -0.381 | 0.004 | K20001 | Gpihbp1 | GPI-anchored HDL-binding protein 1 |
| ENSMUSG00000022584 | 1.164 | 0.504 | 1.210 | 0.030 | None | Ly6c2 | lymphocyte antigen 6 complex, locus C2 |
| ENSMUSG00000022595 | 1.262 | 3.261 | -1.363 | 0.007 | None | Lypd2 | Ly6/Plaur domain containing 2 |
| ENSMUSG00000022651 | 7.536 | 3.248 | 1.216 | 0.010 | None | Retnlg | resistin like gamma |
| ENSMUSG00000022658 | 0.629 | 0.187 | 1.747 | 0.025 | K20526 | Tagln3 | transgelin 3 |
| ENSMUSG00000022698 | 9.050 | 6.528 | 0.476 | 0.006 | None | Naa50 | N(alpha)-acetyltransferase 50, NatE catalytic subunit |
| ENSMUSG00000022754 | 1.577 | 1.027 | 0.619 | 0.038 | None | Tmem45a | transmembrane protein 45a |
| ENSMUSG00000022788 | 1.435 | 0.887 | 0.697 | 0.015 | K05723 | Fgd4 | FYVE, RhoGEF and PH domain containing 4 |
| ENSMUSG00000022789 | 14.975 | 11.052 | 0.442 | 0.004 | K17065 | Dnm1l | dynamin 1-like |
| ENSMUSG00000022797 | 5.958 | 2.399 | 1.315 | 0.000 | K06503 | Tfrc | transferrin receptor |
| ENSMUSG00000022800 | 14.106 | 9.895 | 0.514 | 0.000 | None | Fyttd1 | forty-two-three domain containing 1 |
| ENSMUSG00000022821 | 0.221 | 0.000 | 6.162 | 0.003 | K00451 | Hgd | homogentisate 1, 2-dioxygenase |
| ENSMUSG00000022831 | 11.035 | 7.830 | 0.499 | 0.008 | K06106 | Hcls1 | hematopoietic cell specific Lyn substrate 1 |
| ENSMUSG00000022853 | 9.211 | 14.489 | -0.651 | 0.000 | K07514 | Ehhadh | enoyl-Coenzyme A, hydratase/3-hydroxyacyl Coenzyme A dehydrogenase |
| ENSMUSG00000022877 | 0.871 | 0.005 | 7.203 | 0.010 | None | Hrg | histidine-rich glycoprotein |
| ENSMUSG00000022883 | 0.592 | 0.384 | 0.629 | 0.037 | K06753 | Robo1 | roundabout guidance receptor 1 |
| ENSMUSG00000022941 | 4.377 | 5.988 | -0.449 | 0.037 | None | Ripply3 | ripply transcriptional repressor 3 |
| ENSMUSG00000022947 | 3.619 | 5.258 | -0.537 | 0.019 | K00084 | Cbr3 | carbonyl reductase 3 |
| ENSMUSG00000022949 | 0.309 | 0.121 | 1.351 | 0.024 | K05026 | Clic6 | chloride intracellular channel 6 |
| ENSMUSG00000022974 | 4.091 | 2.797 | 0.552 | 0.025 | K13211 | Paxbp1 | PAX3 and PAX7 binding protein 1 |
| ENSMUSG00000023025 | 4.489 | 3.082 | 0.546 | 0.005 | K18763 | Larp4 | La ribonucleoprotein domain family, member 4 |
| ENSMUSG00000023034 | 36.616 | 66.327 | -0.849 | 0.049 | K04465 | Nr4a1 | nuclear receptor subfamily 4, group A, member 1 |
| ENSMUSG00000023050 | 1.197 | 1.615 | -0.430 | 0.035 | K04423 | Map3k12 | mitogen-activated protein kinase kinase kinase 12 |
| ENSMUSG00000023051 | 9.393 | 12.631 | -0.424 | 0.001 | None | Tarbp2 | TARBP2, RISC loading complex RNA binding subunit |
| ENSMUSG00000023070 | 0.993 | 0.385 | 1.374 | 0.030 | K01053 | Rgn | regucalcin |
| ENSMUSG00000023078 | 27.591 | 2.285 | 3.597 | 0.000 | K10032 | Cxcl13 | chemokine (C-X-C motif) ligand 13 |
| ENSMUSG00000023153 | 0.850 | 2.296 | -1.427 | 0.001 | None | Tmem52 | transmembrane protein 52 |
| ENSMUSG00000023169 | 3.215 | 2.406 | 0.420 | 0.004 | K14990 | Slc38a1 | solute carrier family 38, member 1 |
| ENSMUSG00000023262 | 5.800 | 8.110 | -0.482 | 0.004 | K14677 | Acy1 | aminoacylase 1 |
| ENSMUSG00000023277 | 199.875 | 280.786 | -0.488 | 0.000 | K08870 | Twf2 | twinfilin actin binding protein 2 |
| ENSMUSG00000023345 | 1.444 | 2.243 | -0.633 | 0.026 | K16482 | Poc1a | POC1 centriolar protein A |
| ENSMUSG00000023349 | 2.731 | 1.219 | 1.169 | 0.003 | K17514 | Clec4n | C-type lectin domain family 4, member n |
| ENSMUSG00000023353 | 20.794 | 27.735 | -0.413 | 0.000 | K12491 | Agap3 | ArfGAP with GTPase domain, ankyrin repeat and PH domain 3 |
| ENSMUSG00000023367 | 14.144 | 9.474 | 0.584 | 0.007 | None | Tmem176a | transmembrane protein 176A |
| ENSMUSG00000023439 | 8.921 | 13.456 | -0.592 | 0.005 | K07825 | Gnb3 | guanine nucleotide binding protein (G protein), beta 3 |
| ENSMUSG00000023845 | 14.661 | 10.517 | 0.482 | 0.001 | K01257 | Lnpep | leucyl/cystinyl aminopeptidase |
| ENSMUSG00000023861 | 14.817 | 10.858 | 0.452 | 0.045 | None | Mpc1 | mitochondrial pyruvate carrier 1 |
| ENSMUSG00000023939 | 68.247 | 93.244 | -0.448 | 0.000 | K02874 | Mrpl14 | mitochondrial ribosomal protein L14 |
| ENSMUSG00000024079 | 2.905 | 2.083 | 0.483 | 0.012 | K16195 | Eif2ak2 | eukaryotic translation initiation factor 2-alpha kinase 2 |
| ENSMUSG00000024087 | 8.940 | 5.823 | 0.621 | 0.000 | K07410 | Cyp1b1 | cytochrome P450, family 1, subfamily b, polypeptide 1 |
| ENSMUSG00000024158 | 39.678 | 51.856 | -0.383 | 0.004 | K01069 | Hagh | hydroxyacyl glutathione hydrolase |
| ENSMUSG00000024164 | 140.862 | 83.946 | 0.750 | 0.000 | K03990 | C3 | complement component 3 |
| ENSMUSG00000024181 | 68.577 | 94.225 | -0.456 | 0.000 | K02902 | Mrpl28 | mitochondrial ribosomal protein L28 |
| ENSMUSG00000024187 | 26.950 | 35.495 | -0.396 | 0.002 | K17258 | Fam234a | family with sequence similarity 234, member A |
| ENSMUSG00000024197 | 64.713 | 92.845 | -0.521 | 0.000 | K20287 | Plin3 | perilipin 3 |
| ENSMUSG00000024234 | 4.283 | 2.966 | 0.533 | 0.002 | K18060 | Mtpap | mitochondrial poly(A) polymerase |
| ENSMUSG00000024235 | 3.099 | 1.998 | 0.637 | 0.025 | K04415 | Map3k8 | mitogen-activated protein kinase kinase kinase 8 |
| ENSMUSG00000024276 | 3.008 | 2.176 | 0.471 | 0.041 | K09230 | Zfp397 | zinc finger protein 397 |
| ENSMUSG00000024290 | 4.003 | 2.604 | 0.626 | 0.013 | K04514 | Rock1 | Rho-associated coiled-coil containing protein kinase 1 |
| ENSMUSG00000024298 | 4.077 | 3.001 | 0.445 | 0.012 | K09228 | Zfp871 | zinc finger protein 871 |
| ENSMUSG00000024378 | 2.695 | 1.972 | 0.452 | 0.014 | None | Stard4 | StAR-related lipid transfer (START) domain containing 4 |
| ENSMUSG00000024383 | 1.695 | 1.104 | 0.621 | 0.003 | K04420 | Map3k2 | mitogen-activated protein kinase kinase kinase 2 |
| ENSMUSG00000024397 | 4.179 | 2.070 | 1.017 | 0.022 | K18617 | Aif1 | allograft inflammatory factor 1 |
| ENSMUSG00000024410 | 7.258 | 9.664 | -0.410 | 0.001 | None | 3110002H16Rik | RIKEN cDNA 3110002H16 gene |
| ENSMUSG00000024440 | 5.183 | 7.315 | -0.495 | 0.016 | K16499 | Pcdh12 | protocadherin 12 |
| ENSMUSG00000024480 | 8.559 | 6.243 | 0.458 | 0.005 | K12399 | Ap3s1 | adaptor-related protein complex 3, sigma 1 subunit |
| ENSMUSG00000024539 | 4.822 | 3.369 | 0.520 | 0.006 | K18026 | Ptpn2 | protein tyrosine phosphatase, non-receptor type 2 |
| ENSMUSG00000024558 | 1.995 | 3.110 | -0.637 | 0.001 | K06855 | Mapk4 | mitogen-activated protein kinase 4 |
| ENSMUSG00000024561 | 15.531 | 21.464 | -0.465 | 0.000 | K11589 | Mbd1 | methyl-CpG binding domain protein 1 |
| ENSMUSG00000024579 | 0.597 | 1.194 | -1.000 | 0.041 | None | Pcyox1l | prenylcysteine oxidase 1 like |
| ENSMUSG00000024614 | 5.136 | 3.424 | 0.589 | 0.001 | K09585 | Tmx3 | thioredoxin-related transmembrane protein 3 |
| ENSMUSG00000024677 | 2.752 | 1.635 | 0.755 | 0.010 | None | Ms4a6b | membrane-spanning 4-domains, subfamily A, member 6B |
| ENSMUSG00000024679 | 5.003 | 2.432 | 1.044 | 0.001 | None | Ms4a6d | membrane-spanning 4-domains, subfamily A, member 6D |
| ENSMUSG00000024695 | 19.220 | 14.666 | 0.394 | 0.001 | None | Zfp91 | zinc finger protein 91 |
| ENSMUSG00000024696 | 2.001 | 1.062 | 0.915 | 0.042 | None | Lpxn | leupaxin |
| ENSMUSG00000024730 | 0.961 | 0.319 | 1.592 | 0.049 | None | Ms4a8a | membrane-spanning 4-domains, subfamily A, member 8A |
| ENSMUSG00000024747 | 0.278 | 0.080 | 1.811 | 0.048 | K07249 | Aldh1a7 | aldehyde dehydrogenase family 1, subfamily A7 |
| ENSMUSG00000024754 | 7.181 | 5.470 | 0.396 | 0.029 | None | Tmem2 | transmembrane protein 2 |
| ENSMUSG00000024759 | 6.586 | 4.703 | 0.489 | 0.000 | K17339 | Atl3 | atlastin GTPase 3 |
| ENSMUSG00000024766 | 2.540 | 1.493 | 0.769 | 0.001 | None | Lipo3 | lipase, member O3 |
| ENSMUSG00000024793 | 0.940 | 1.803 | -0.934 | 0.017 | K05160 | Tnfrsf25 | tumor necrosis factor receptor superfamily, member 25 |
| ENSMUSG00000024803 | 964.787 | 538.196 | 0.843 | 0.000 | None | Ankrd1 | ankyrin repeat domain 1 (cardiac muscle) |
| ENSMUSG00000024806 | 4.617 | 1.834 | 1.333 | 0.014 | K17303 | Mlana | melan-A |
| ENSMUSG00000024807 | 5.826 | 7.595 | -0.380 | 0.001 | K10601 | Syvn1 | synovial apoptosis inhibitor 1, synoviolin |
| ENSMUSG00000024810 | 4.434 | 3.222 | 0.464 | 0.036 | K12967 | Il33 | interleukin 33 |
| ENSMUSG00000024835 | 0.976 | 2.668 | -1.449 | 0.003 | K13886 | Coro1b | coronin, actin binding protein 1B |
| ENSMUSG00000024845 | 0.648 | 1.219 | -0.909 | 0.042 |  | Tmem134 | transmembrane protein 134 |
| ENSMUSG00000024866 | 14.256 | 19.603 | -0.457 | 0.002 | K18458 | Acy3 | aspartoacylase (aminoacylase) 3 |
| ENSMUSG00000024875 | 39.097 | 53.098 | -0.441 | 0.001 | K20362 | Yif1a | Yip1 interacting factor homolog A (S. cerevisiae) |
| ENSMUSG00000024885 | 4.090 | 3.008 | 0.444 | 0.037 | K00129 | Aldh3b1 | aldehyde dehydrogenase 3 family, member B1 |
| ENSMUSG00000024892 | 6.569 | 11.204 | -0.771 | 0.000 | K01958 | Pcx | pyruvate carboxylase |
| ENSMUSG00000024925 | 10.675 | 14.159 | -0.404 | 0.013 | K10745 | Rnaseh2c | ribonuclease H2, subunit C |
| ENSMUSG00000024944 | 17.929 | 23.564 | -0.392 | 0.000 | K07943 | Arl2 | ADP-ribosylation factor-like 2 |
| ENSMUSG00000024974 | 4.626 | 3.315 | 0.487 | 0.049 | K06669 | Smc3 | structural maintenance of chromosomes 3 |
| ENSMUSG00000024975 | 7.587 | 5.603 | 0.440 | 0.009 | K16865 | Pdcd4 | programmed cell death 4 |
| ENSMUSG00000024978 | 50.960 | 67.836 | -0.411 | 0.005 | K00629 | Gpam | glycerol-3-phosphate acyltransferase, mitochondrial |
| ENSMUSG00000024993 | 4.279 | 3.065 | 0.483 | 0.003 | None | Fam45a | family with sequence similarity 45, member A |
| ENSMUSG00000025017 | 3.711 | 1.794 | 1.053 | 0.000 | K12230 | Pik3ap1 | phosphoinositide-3-kinase adaptor protein 1 |
| ENSMUSG00000025019 | 2.592 | 1.772 | 0.551 | 0.022 | None | Lcor | ligand dependent nuclear receptor corepressor |
| ENSMUSG00000025024 | 11.535 | 8.701 | 0.410 | 0.001 | K12839 | Smndc1 | survival motor neuron domain containing 1 |
| ENSMUSG00000025040 | 11.223 | 8.063 | 0.480 | 0.007 | K17986 | Fundc1 | FUN14 domain containing 1 |
| ENSMUSG00000025044 | 1.227 | 0.624 | 0.977 | 0.006 | K06558 | Msr1 | macrophage scavenger receptor 1 |
| ENSMUSG00000025059 | 5.013 | 3.382 | 0.571 | 0.000 | K00864 | Gk | glycerol kinase |
| ENSMUSG00000025060 | 11.555 | 8.436 | 0.458 | 0.000 | K08836 | Slk | STE20-like kinase |
| ENSMUSG00000025141 | 39.654 | 53.482 | -0.429 | 0.000 | None | Myadml2 | myeloid-associated differentiation marker-like 2 |
| ENSMUSG00000025142 | 7.724 | 10.072 | -0.380 | 0.016 | K15627 | Aspscr1 | alveolar soft part sarcoma chromosome region, candidate 1 (human) |
| ENSMUSG00000025178 | 11.076 | 14.584 | -0.394 | 0.000 | K13711 | Pi4k2a | phosphatidylinositol 4-kinase type 2 alpha |
| ENSMUSG00000025196 | 0.247 | 0.013 | 4.256 | 0.015 | K01292 | Cpn1 | carboxypeptidase N, polypeptide 1 |
| ENSMUSG00000025204 | 126.542 | 167.731 | -0.404 | 0.000 | K03964 | Ndufb8 | NADH dehydrogenase (ubiquinone) 1 beta subcomplex 8 |
| ENSMUSG00000025262 | 2.642 | 2.021 | 0.389 | 0.015 | None | Fam120c | family with sequence similarity 120, member C |
| ENSMUSG00000025279 | 0.134 | 0.037 | 1.874 | 0.027 | K11995 | Dnase1l3 | deoxyribonuclease 1-like 3 |
| ENSMUSG00000025326 | 2.733 | 1.690 | 0.699 | 0.004 | K10587 | Ube3a | ubiquitin protein ligase E3A |
| ENSMUSG00000025491 | 14.424 | 8.162 | 0.825 | 0.009 | K19831 | Ifitm1 | interferon induced transmembrane protein 1 |
| ENSMUSG00000025495 | 6.779 | 10.344 | -0.607 | 0.000 | K08730 | Ptdss2 | phosphatidylserine synthase 2 |
| ENSMUSG00000025509 | 325.148 | 454.846 | -0.482 | 0.000 | K16816 | Pnpla2 | patatin-like phospholipase domain containing 2 |
| ENSMUSG00000025579 | 54.331 | 71.002 | -0.385 | 0.000 | K12316 | Gaa | glucosidase, alpha, acid |
| ENSMUSG00000025612 | 10.111 | 7.621 | 0.411 | 0.000 | K09042 | Bach1 | BTB and CNC homology 1, basic leucine zipper transcription factor 1 |
| ENSMUSG00000025626 | 1.887 | 1.360 | 0.476 | 0.021 | None | Phf6 | PHD finger protein 6 |
| ENSMUSG00000025651 | 11.765 | 16.138 | -0.453 | 0.000 | K00414 | Uqcrc1 | ubiquinol-cytochrome c reductase core protein 1 |
| ENSMUSG00000025656 | 2.930 | 2.160 | 0.443 | 0.003 | None | Arhgef9 | CDC42 guanine nucleotide exchange factor (GEF) 9 |
| ENSMUSG00000025792 | 9.859 | 13.007 | -0.396 | 0.005 | K13577 | Slc25a10 | solute carrier family 25 (mitochondrial carrier, dicarboxylate transporter), member 10 |
| ENSMUSG00000025854 | 3.984 | 5.657 | -0.504 | 0.001 | None | Fam20c | family with sequence similarity 20, member C |
| ENSMUSG00000025860 | 7.016 | 5.257 | 0.419 | 0.010 | K04725 | Xiap | X-linked inhibitor of apoptosis |
| ENSMUSG00000025862 | 5.719 | 4.046 | 0.504 | 0.005 | K06671 | Stag2 | stromal antigen 2 |
| ENSMUSG00000025888 | 1.917 | 1.026 | 0.902 | 0.035 | K01370 | Casp1 | caspase 1 |
| ENSMUSG00000025898 | 2.498 | 1.614 | 0.633 | 0.020 | None | Cwf19l2 | CWF19-like 2, cell cycle control (S. pombe) |
| ENSMUSG00000025915 | 4.519 | 2.656 | 0.768 | 0.000 | K13304 | Sgk3 | serum/glucocorticoid regulated kinase 3 |
| ENSMUSG00000025939 | 7.015 | 5.171 | 0.442 | 0.007 | K10688 | Ube2w | ubiquitin-conjugating enzyme E2W (putative) |
| ENSMUSG00000025991 | 1.150 | 0.049 | 4.563 | 0.012 | K01948 | Cps1 | carbamoyl-phosphate synthetase 1 |
| ENSMUSG00000025993 | 7.881 | 12.716 | -0.688 | 0.000 | K14685 | Slc40a1 | solute carrier family 40 (iron-regulated transporter), member 1 |
| ENSMUSG00000026005 | 2.490 | 1.731 | 0.528 | 0.030 | K01783 | Rpe | ribulose-5-phosphate-3-epimerase |
| ENSMUSG00000026072 | 5.081 | 3.756 | 0.439 | 0.000 | K04386 | Il1r1 | interleukin 1 receptor, type I |
| ENSMUSG00000026094 | 5.342 | 3.867 | 0.469 | 0.015 | K08804 | Stk17b | serine/threonine kinase 17b (apoptosis-inducing) |
| ENSMUSG00000026104 | 3.914 | 2.788 | 0.491 | 0.034 | K11220 | Stat1 | signal transducer and activator of transcription 1 |
| ENSMUSG00000026107 | 7.872 | 5.885 | 0.422 | 0.009 | None | Nabp1 | nucleic acid binding protein 1 |
| ENSMUSG00000026121 | 7.537 | 10.308 | -0.450 | 0.006 | K06521 | Sema4c | sema domain, immunoglobulin domain (Ig), transmembrane domain (TM) and short cytoplasmic domain, (semaphorin) 4C |
| ENSMUSG00000026153 | 4.744 | 3.386 | 0.489 | 0.001 | None | Fam135a | family with sequence similarity 135, member A |
| ENSMUSG00000026158 | 3.447 | 2.600 | 0.410 | 0.022 | None | Ogfrl1 | opioid growth factor receptor-like 1 |
| ENSMUSG00000026202 | 143.473 | 105.824 | 0.441 | 0.001 | K07374 | Tuba4a | tubulin, alpha 4A |
| ENSMUSG00000026213 | 5.098 | 6.759 | -0.404 | 0.002 | None | Stk11ip | serine/threonine kinase 11 interacting protein |
| ENSMUSG00000026357 | 0.592 | 0.137 | 2.098 | 0.009 | K16449 | Rgs18 | regulator of G-protein signaling 18 |
| ENSMUSG00000026361 | 2.456 | 1.822 | 0.433 | 0.001 | K15175 | Cdc73 | cell division cycle 73, Paf1/RNA polymerase II complex component |
| ENSMUSG00000026384 | 1.900 | 1.297 | 0.555 | 0.041 | K18037 | Ptpn4 | protein tyrosine phosphatase, non-receptor type 4 |
| ENSMUSG00000026390 | 0.930 | 0.018 | 5.657 | 0.004 | K13884 | Marco | macrophage receptor with collagenous structure |
| ENSMUSG00000026393 | 17.954 | 13.515 | 0.413 | 0.001 | K08857 | Nek7 | NIMA (never in mitosis gene a)-related expressed kinase 7 |
| ENSMUSG00000026395 | 2.194 | 0.995 | 1.152 | 0.024 | K06478 | Ptprc | protein tyrosine phosphatase, receptor type, C |
| ENSMUSG00000026399 | 3.923 | 2.622 | 0.583 | 0.005 | K04006 | Cd55 | CD55 molecule, decay accelerating factor for complement |
| ENSMUSG00000026405 | 0.263 | 0.000 | 7.587 | 0.041 | K04002 | C4bp | complement component 4 binding protein |
| ENSMUSG00000026418 | 1.329 | 0.650 | 1.040 | 0.027 | K10371 | Tnni1 | troponin I, skeletal, slow 1 |
| ENSMUSG00000026421 | 50.723 | 68.729 | -0.434 | 0.004 | K09377 | Csrp1 | cysteine and glycine-rich protein 1 |
| ENSMUSG00000026434 | 8.654 | 5.645 | 0.619 | 0.000 | None | Nucks1 | nuclear casein kinase and cyclin-dependent kinase substrate 1 |
| ENSMUSG00000026437 | 5.767 | 8.302 | -0.525 | 0.001 | K15596 | Cdk18 | cyclin-dependent kinase 18 |
| ENSMUSG00000026484 | 6.509 | 4.645 | 0.489 | 0.008 | K10695 | Rnf2 | ring finger protein 2 |
| ENSMUSG00000026544 | 9.794 | 14.289 | -0.542 | 0.000 | K14165 | Dusp23 | dual specificity phosphatase 23 |
| ENSMUSG00000026548 | 5.518 | 3.094 | 0.840 | 0.009 | None | Slamf9 | SLAM family member 9 |
| ENSMUSG00000026579 | 0.230 | 0.089 | 1.369 | 0.028 | K03902 | F5 | coagulation factor V |
| ENSMUSG00000026589 | 1.497 | 0.942 | 0.673 | 0.005 | K20353 | Sec16b | SEC16 homolog B (S. cerevisiae) |
| ENSMUSG00000026605 | 0.598 | 0.358 | 0.741 | 0.033 | K11499 | Cenpf | centromere protein F |
| ENSMUSG00000026643 | 3.336 | 2.488 | 0.426 | 0.006 | K00671 | Nmt2 | N-myristoyltransferase 2 |
| ENSMUSG00000026648 | 0.742 | 0.480 | 0.630 | 0.042 | K10887 | Dclre1c | DNA cross-link repair 1C |
| ENSMUSG00000026656 | 6.876 | 4.008 | 0.783 | 0.003 | K12560 | Fcgr2b | Fc receptor, IgG, low affinity IIb |
| ENSMUSG00000026675 | 5.151 | 3.846 | 0.425 | 0.006 | K13373 | Hsd17b7 | hydroxysteroid (17-beta) dehydrogenase 7 |
| ENSMUSG00000026712 | 11.250 | 7.286 | 0.630 | 0.000 | K06560 | Mrc1 | mannose receptor, C type 1 |
| ENSMUSG00000026715 | 0.489 | 0.013 | 5.278 | 0.046 | K03911 | Serpinc1 | serine (or cysteine) peptidase inhibitor, clade C (antithrombin), member 1 |
| ENSMUSG00000026773 | 10.728 | 7.613 | 0.500 | 0.003 | K01103 | Pfkfb3 | 6-phosphofructo-2-kinase/fructose-2,6-biphosphatase 3 |
| ENSMUSG00000026775 | 9.090 | 6.961 | 0.388 | 0.040 | K08955 | Yme1l1 | YME1-like 1 (S. cerevisiae) |
| ENSMUSG00000026786 | 2.336 | 1.308 | 0.840 | 0.000 | K17704 | Apbb1ip | amyloid beta (A4) precursor protein-binding, family B, member 1 interacting protein |
| ENSMUSG00000026791 | 8.334 | 11.195 | -0.423 | 0.000 | K08145 | Slc2a8 | solute carrier family 2, (facilitated glucose transporter), member 8 |
| ENSMUSG00000026796 | 16.550 | 21.611 | -0.382 | 0.001 | None | Fam129b | family with sequence similarity 129, member B |
| ENSMUSG00000026822 | 8.181 | 5.521 | 0.571 | 0.029 | None | Lcn2 | lipocalin 2 |
| ENSMUSG00000026824 | 6.714 | 4.430 | 0.602 | 0.003 | K04997 | Kcnj3 | potassium inwardly-rectifying channel, subfamily J, member 3 |
| ENSMUSG00000026832 | 0.677 | 0.371 | 0.868 | 0.034 | None | Cytip | cytohesin 1 interacting protein |
| ENSMUSG00000026874 | 0.161 | 0.000 | 7.377 | 0.007 | K03994 | Hc | hemolytic complement |
| ENSMUSG00000026896 | 2.436 | 1.807 | 0.434 | 0.013 | K12647 | Ifih1 | interferon induced with helicase C domain 1 |
| ENSMUSG00000026944 | 9.620 | 12.637 | -0.392 | 0.001 | K05642 | Abca2 | ATP-binding cassette, sub-family A (ABC1), member 2 |
| ENSMUSG00000026950 | 0.235 | 0.105 | 1.170 | 0.003 | K18267 | Neb | nebulin |
| ENSMUSG00000026977 | 7.669 | 5.540 | 0.471 | 0.003 | K10662 | 7-Mar | membrane-associated ring finger (C3HC4) 7 |
| ENSMUSG00000026981 | 0.262 | 0.070 | 1.903 | 0.022 | K05481 | Il1rn | interleukin 1 receptor antagonist |
| ENSMUSG00000026987 | 4.517 | 3.344 | 0.437 | 0.002 | None | Baz2b | bromodomain adjacent to zinc finger domain, 2B |
| ENSMUSG00000027009 | 0.537 | 0.251 | 1.104 | 0.016 | K06483 | Itga4 | integrin alpha 4 |
| ENSMUSG00000027076 | 60.507 | 80.906 | -0.416 | 0.002 | K17778 | Timm10 | translocase of inner mitochondrial membrane 10 |
| ENSMUSG00000027109 | 7.727 | 5.588 | 0.471 | 0.001 | K09193 | Sp3 | trans-acting transcription factor 3 |
| ENSMUSG00000027162 | 8.643 | 6.008 | 0.528 | 0.000 | K19931 | Lin7c | lin-7 homolog C (C. elegans) |
| ENSMUSG00000027164 | 4.389 | 3.380 | 0.379 | 0.007 | K03175 | Traf6 | TNF receptor-associated factor 6 |
| ENSMUSG00000027176 | 2.195 | 1.477 | 0.573 | 0.020 | K14408 | Cstf3 | cleavage stimulation factor, 3' pre-RNA, subunit 3 |
| ENSMUSG00000027195 | 13.701 | 9.231 | 0.572 | 0.000 | K10251 | Hsd17b12 | hydroxysteroid (17-beta) dehydrogenase 12 |
| ENSMUSG00000027206 | 14.970 | 11.354 | 0.402 | 0.015 | K12176 | Cops2 | COP9 signalosome subunit 2 |
| ENSMUSG00000027222 | 18.495 | 25.688 | -0.471 | 0.000 | K13335 | Pex16 | peroxisomal biogenesis factor 16 |
| ENSMUSG00000027236 | 6.478 | 4.725 | 0.458 | 0.004 | K03245 | Eif3j1 | eukaryotic translation initiation factor 3, subunit J1 |
| ENSMUSG00000027248 | 54.186 | 41.442 | 0.390 | 0.000 | K08056 | Pdia3 | protein disulfide isomerase associated 3 |
| ENSMUSG00000027249 | 0.887 | 0.003 | 8.097 | 0.026 | K01313 | F2 | coagulation factor II |
| ENSMUSG00000027296 | 0.162 | 0.542 | -1.749 | 0.038 | K00911 | Itpka | inositol 1,4,5-trisphosphate 3-kinase A |
| ENSMUSG00000027314 | 8.686 | 11.470 | -0.399 | 0.009 | K06051 | Dll4 | delta like canonical Notch ligand 4 |
| ENSMUSG00000027322 | 3.206 | 2.057 | 0.644 | 0.000 | K06548 | Siglec1 | sialic acid binding Ig-like lectin 1, sialoadhesin |
| ENSMUSG00000027332 | 84.249 | 126.229 | -0.582 | 0.000 | K00253 | Ivd | isovaleryl coenzyme A dehydrogenase |
| ENSMUSG00000027335 | 0.898 | 1.495 | -0.734 | 0.027 | K04137 | Adra1d | adrenergic receptor, alpha 1d |
| ENSMUSG00000027351 | 5.453 | 3.627 | 0.591 | 0.000 | K04703 | Spred1 | sprouty protein with EVH-1 domain 1, related sequence |
| ENSMUSG00000027359 | 0.431 | 0.037 | 3.550 | 0.009 | K08746 | Slc27a2 | solute carrier family 27 (fatty acid transporter), member 2 |
| ENSMUSG00000027365 | 5.618 | 3.755 | 0.585 | 0.002 | K04982 | Trpm7 | transient receptor potential cation channel, subfamily M, member 7 |
| ENSMUSG00000027377 | 2.759 | 4.148 | -0.586 | 0.005 | None | Mall | mal, T cell differentiation protein-like |
| ENSMUSG00000027387 | 2.890 | 1.693 | 0.775 | 0.005 | None | Zc3h8 | zinc finger CCCH type containing 8 |
| ENSMUSG00000027423 | 30.851 | 21.976 | 0.492 | 0.000 | K17920 | Snx5 | sorting nexin 5 |
| ENSMUSG00000027427 | 3.617 | 2.419 | 0.583 | 0.001 | K03025 | Polr3f | polymerase (RNA) III (DNA directed) polypeptide F |
| ENSMUSG00000027438 | 1.129 | 0.691 | 0.712 | 0.015 | None | Napb | N-ethylmaleimide sensitive fusion protein attachment protein beta |
| ENSMUSG00000027499 | 52.463 | 40.018 | 0.393 | 0.001 | K15985 | Pkia | protein kinase inhibitor, alpha |
| ENSMUSG00000027508 | 1.043 | 0.687 | 0.603 | 0.006 | None | Pag1 | phosphoprotein associated with glycosphingolipid microdomains 1 |
| ENSMUSG00000027514 | 1.544 | 0.810 | 0.932 | 0.010 | K12965 | Zbp1 | Z-DNA binding protein 1 |
| ENSMUSG00000027523 | 4.119 | 7.361 | -0.840 | 0.000 | K04632 | Gnas | GNAS (guanine nucleotide binding protein, alpha stimulating) complex locus |
| ENSMUSG00000027547 | 0.353 | 0.610 | -0.786 | 0.022 | K19871 | Sall4 | spalt like transcription factor 4 |
| ENSMUSG00000027602 | 355.061 | 467.301 | -0.394 | 0.000 | K10435 | Map1lc3a | microtubule-associated protein 1 light chain 3 alpha |
| ENSMUSG00000027620 | 32.665 | 24.342 | 0.428 | 0.018 | K13091 | Rbm39 | RNA binding motif protein 39 |
| ENSMUSG00000027646 | 1.860 | 2.747 | -0.560 | 0.017 | K05704 | Src | Rous sarcoma oncogene |
| ENSMUSG00000027680 | 13.668 | 10.047 | 0.447 | 0.007 | K15516 | Fxr1 | fragile X mental retardation gene 1, autosomal homolog |
| ENSMUSG00000027708 | 4.013 | 2.312 | 0.799 | 0.000 | K17822 | Dcun1d1 | DCN1, defective in cullin neddylation 1, domain containing 1 (S. cerevisiae) |
| ENSMUSG00000027714 | 4.972 | 3.760 | 0.405 | 0.024 | K03678 | Exosc9 | exosome component 9 |
| ENSMUSG00000027763 | 15.046 | 10.951 | 0.462 | 0.001 | K14943 | Mbnl1 | muscleblind like splicing factor 1 |
| ENSMUSG00000027770 | 4.610 | 3.431 | 0.429 | 0.003 | K14442 | Dhx36 | DEAH (Asp-Glu-Ala-His) box polypeptide 36 |
| ENSMUSG00000027792 | 1.956 | 1.385 | 0.500 | 0.024 | K01050 | Bche | butyrylcholinesterase |
| ENSMUSG00000027804 | 6.015 | 4.383 | 0.459 | 0.001 | K05864 | Ppid | peptidylprolyl isomerase D (cyclophilin D) |
| ENSMUSG00000027808 | 9.906 | 7.452 | 0.415 | 0.002 | None | Serp1 | stress-associated endoplasmic reticulum protein 1 |
| ENSMUSG00000027822 | 2.663 | 1.913 | 0.479 | 0.047 | K03372 | Slc33a1 | solute carrier family 33 (acetyl-CoA transporter), member 1 |
| ENSMUSG00000027828 | 52.110 | 40.116 | 0.380 | 0.000 | K13251 | Ssr3 | signal sequence receptor, gamma |
| ENSMUSG00000027835 | 8.091 | 5.126 | 0.661 | 0.001 | K18269 | Pdcd10 | programmed cell death 10 |
| ENSMUSG00000027894 | 0.498 | 0.810 | -0.696 | 0.009 | K05048 | Slc6a17 | solute carrier family 6 (neurotransmitter transporter), member 17 |
| ENSMUSG00000027968 | 8.422 | 6.319 | 0.417 | 0.012 | K15191 | Larp7 | La ribonucleoprotein domain family, member 7 |
| ENSMUSG00000027995 | 3.052 | 1.870 | 0.710 | 0.004 | K10159 | Tlr2 | toll-like receptor 2 |
| ENSMUSG00000027996 | 5.731 | 3.919 | 0.551 | 0.016 | K02176 | Sfrp2 | secreted frizzled-related protein 2 |
| ENSMUSG00000027999 | 14.974 | 21.398 | -0.513 | 0.000 | K01047 | Pla2g12a | phospholipase A2, group XIIA |
| ENSMUSG00000028001 | 2.103 | 0.000 | 10.013 | 0.009 | K03903 | Fga | fibrinogen alpha chain |
| ENSMUSG00000028011 | 0.542 | 0.005 | 6.662 | 0.003 | K00453 | Tdo2 | tryptophan 2,3-dioxygenase |
| ENSMUSG00000028028 | 0.635 | 0.416 | 0.613 | 0.037 | K08868 | Alpk1 | alpha-kinase 1 |
| ENSMUSG00000028036 | 0.943 | 0.610 | 0.631 | 0.044 | K04262 | Ptgfr | prostaglandin F receptor |
| ENSMUSG00000028042 | 7.865 | 11.084 | -0.492 | 0.000 | K10494 | Zbtb7b | zinc finger and BTB domain containing 7B |
| ENSMUSG00000028124 | 1.163 | 2.520 | -1.113 | 0.000 | K11205 | Gclm | glutamate-cysteine ligase, modifier subunit |
| ENSMUSG00000028134 | 5.158 | 3.574 | 0.532 | 0.000 | K14948 | Ptbp2 | polypyrimidine tract binding protein 2 |
| ENSMUSG00000028159 | 3.445 | 2.375 | 0.538 | 0.026 | K12229 | Dapp1 | dual adaptor for phosphotyrosine and 3-phosphoinositides 1 |
| ENSMUSG00000028180 | 9.000 | 6.640 | 0.443 | 0.027 | None | Zranb2 | zinc finger, RAN-binding domain containing 2 |
| ENSMUSG00000028211 | 6.121 | 3.476 | 0.819 | 0.000 | K15310 | Trp53inp1 | transformation related protein 53 inducible nuclear protein 1 |
| ENSMUSG00000028248 | 7.221 | 5.139 | 0.496 | 0.026 | K13170 | Pnisr | PNN interacting serine/arginine-rich |
| ENSMUSG00000028252 | 1.938 | 1.203 | 0.691 | 0.012 | K15161 | Ccnc | cyclin C |
| ENSMUSG00000028261 | 7.361 | 5.445 | 0.438 | 0.001 | K18161 | Ndufaf4 | NADH dehydrogenase (ubiquinone) 1 alpha subcomplex, assembly factor 4 |
| ENSMUSG00000028268 | 6.554 | 3.811 | 0.785 | 0.003 | None | Gbp3 | guanylate binding protein 3 |
| ENSMUSG00000028270 | 12.841 | 8.636 | 0.577 | 0.010 | None | Gbp2 | guanylate binding protein 2 |
| ENSMUSG00000028274 | 2.597 | 1.773 | 0.553 | 0.006 | K13917 | Rngtt | RNA guanylyltransferase and 5'-phosphatase |
| ENSMUSG00000028343 | 10.324 | 7.871 | 0.394 | 0.014 | K17264 | Erp44 | endoplasmic reticulum protein 44 |
| ENSMUSG00000028356 | 3.946 | 0.007 | 8.931 | 0.009 | None | Ambp | alpha 1 microglobulin/bikunin |
| ENSMUSG00000028382 | 5.443 | 3.878 | 0.494 | 0.007 | K17844 | Ptbp3 | polypyrimidine tract binding protein 3 |
| ENSMUSG00000028389 | 0.955 | 0.366 | 1.383 | 0.001 | K09228 | Zfp37 | zinc finger protein 37 |
| ENSMUSG00000028393 | 20.784 | 28.228 | -0.441 | 0.003 | K01698 | Alad | aminolevulinate, delta-, dehydratase |
| ENSMUSG00000028399 | 1.171 | 0.742 | 0.663 | 0.011 | K06777 | Ptprd | protein tyrosine phosphatase, receptor type, D |
| ENSMUSG00000028413 | 31.148 | 23.850 | 0.388 | 0.001 | K07966 | B4galt1 | UDP-Gal:betaGlcNAc beta 1,4- galactosyltransferase, polypeptide 1 |
| ENSMUSG00000028445 | 4.215 | 6.458 | -0.614 | 0.018 |  | Enho | energy homeostasis associated |
| ENSMUSG00000028459 | 1.563 | 0.654 | 1.260 | 0.020 | K06504 | Cd72 | CD72 antigen |
| ENSMUSG00000028494 | 50.449 | 83.076 | -0.718 | 0.000 | K17284 | Plin2 | perilipin 2 |
| ENSMUSG00000028518 | 15.362 | 11.669 | 0.399 | 0.002 | K07198 | Prkaa2 | protein kinase, AMP-activated, alpha 2 catalytic subunit |
| ENSMUSG00000028522 | 5.619 | 4.087 | 0.462 | 0.003 | None | Mier1 | MEIR1 treanscription regulator |
| ENSMUSG00000028541 | 1.268 | 1.831 | -0.528 | 0.026 | K07967 | B4galt2 | UDP-Gal:betaGlcNAc beta 1,4- galactosyltransferase, polypeptide 2 |
| ENSMUSG00000028560 | 5.078 | 3.535 | 0.525 | 0.001 | K11832 | Usp1 | ubiquitin specific peptidase 1 |
| ENSMUSG00000028572 | 2.000 | 1.369 | 0.550 | 0.032 | K16612 | Hook1 | hook microtubule tethering protein 1 |
| ENSMUSG00000028581 | 16.575 | 12.247 | 0.442 | 0.025 | K12387 | Laptm5 | lysosomal-associated protein transmembrane 5 |
| ENSMUSG00000028597 | 6.647 | 8.925 | -0.423 | 0.021 | K00432 | Gpx7 | glutathione peroxidase 7 |
| ENSMUSG00000028601 | 19.450 | 25.433 | -0.384 | 0.001 | None | Echdc2 | enoyl Coenzyme A hydratase domain containing 2 |
| ENSMUSG00000028631 | 3.141 | 4.505 | -0.518 | 0.001 | K04929 | Kcnq4 | potassium voltage-gated channel, subfamily Q, member 4 |
| ENSMUSG00000028676 | 8.485 | 6.120 | 0.474 | 0.004 | K12900 | Srsf10 | serine/arginine-rich splicing factor 10 |
| ENSMUSG00000028743 | 29.268 | 38.235 | -0.384 | 0.003 | K15303 | Akr7a5 | aldo-keto reductase family 7, member A5 (aflatoxin aldehyde reductase) |
| ENSMUSG00000028744 | 2.771 | 3.847 | -0.470 | 0.015 | None | Pqlc2 | PQ loop repeat containing 2 |
| ENSMUSG00000028780 | 1.913 | 1.232 | 0.638 | 0.018 | K06840 | Sema3c | sema domain, immunoglobulin domain (Ig), short basic domain, secreted, (semaphorin) 3C |
| ENSMUSG00000028782 | 0.448 | 0.855 | -0.930 | 0.002 | K04597 | Adgrb2 | adhesion G protein-coupled receptor B2 |
| ENSMUSG00000028788 | 53.557 | 41.178 | 0.382 | 0.000 | K18041 | Ptp4a2 | protein tyrosine phosphatase 4a2 |
| ENSMUSG00000028830 | 11.276 | 14.848 | -0.395 | 0.000 | None | AU040320 | expressed sequence AU040320 |
| ENSMUSG00000028838 | 4.888 | 6.813 | -0.478 | 0.035 | K02368 | Extl1 | exostoses (multiple)-like 1 |
| ENSMUSG00000028859 | 3.301 | 1.863 | 0.829 | 0.030 | K05061 | Csf3r | colony stimulating factor 3 receptor (granulocyte) |
| ENSMUSG00000028874 | 1.332 | 0.531 | 1.329 | 0.007 | K08891 | Fgr | FGR proto-oncogene, Src family tyrosine kinase |
| ENSMUSG00000028885 | 1.983 | 0.925 | 1.104 | 0.010 | K01128 | Smpdl3b | sphingomyelin phosphodiesterase, acid-like 3B |
| ENSMUSG00000028907 | 4.598 | 3.475 | 0.407 | 0.011 | K14769 | Utp11 | UTP11 small subunit processome component |
| ENSMUSG00000028909 | 3.139 | 4.198 | -0.417 | 0.009 | K16662 | Ptpru | protein tyrosine phosphatase, receptor type, U |
| ENSMUSG00000028926 | 2.808 | 1.723 | 0.708 | 0.000 | K08821 | Cdk14 | cyclin-dependent kinase 14 |
| ENSMUSG00000028990 | 11.096 | 14.646 | -0.399 | 0.006 | None | Lzic | leucine zipper and CTNNBIP1 domain containing |
| ENSMUSG00000029001 | 10.285 | 13.586 | -0.399 | 0.004 | K10103 | Fbxo44 | F-box protein 44 |
| ENSMUSG00000029028 | 9.277 | 12.191 | -0.392 | 0.006 | None | Lrrc47 | leucine rich repeat containing 47 |
| ENSMUSG00000029060 | 8.937 | 12.514 | -0.484 | 0.000 | K10645 | Mib2 | mindbomb E3 ubiquitin protein ligase 2 |
| ENSMUSG00000029108 | 10.770 | 7.790 | 0.470 | 0.000 | K16498 | Pcdh7 | protocadherin 7 |
| ENSMUSG00000029122 | 3.080 | 4.125 | -0.419 | 0.011 | K19605 | Evc | EvC ciliary complex subunit 1 |
| ENSMUSG00000029123 | 0.872 | 1.520 | -0.799 | 0.006 | K08793 | Stk32b | serine/threonine kinase 32B |
| ENSMUSG00000029145 | 19.557 | 25.818 | -0.398 | 0.000 | K03680 | Eif2b4 | eukaryotic translation initiation factor 2B, subunit 4 delta |
| ENSMUSG00000029161 | 1.324 | 2.505 | -0.919 | 0.014 | None | Cgref1 | cell growth regulator with EF hand domain 1 |
| ENSMUSG00000029167 | 14.523 | 11.039 | 0.399 | 0.001 | K07202 | Ppargc1a | peroxisome proliferative activated receptor, gamma, coactivator 1 alpha |
| ENSMUSG00000029201 | 2.619 | 1.982 | 0.405 | 0.022 | K00012 | Ugdh | UDP-glucose dehydrogenase |
| ENSMUSG00000029233 | 5.817 | 4.468 | 0.383 | 0.033 | K12345 | Srd5a3 | steroid 5 alpha-reductase 3 |
| ENSMUSG00000029253 | 1.442 | 0.869 | 0.733 | 0.010 | K11497 | Cenpc1 | centromere protein C1 |
| ENSMUSG00000029321 | 11.444 | 8.391 | 0.448 | 0.038 | K14346 | Slc10a6 | solute carrier family 10 (sodium/bile acid cotransporter family), member 6 |
| ENSMUSG00000029322 | 3.977 | 0.937 | 2.082 | 0.000 | None | Plac8 | placenta-specific 8 |
| ENSMUSG00000029328 | 30.188 | 21.956 | 0.463 | 0.000 | K13044 | Hnrnpdl | heterogeneous nuclear ribonucleoprotein D-like |
| ENSMUSG00000029368 | 57.552 | 0.148 | 8.605 | 0.000 | K16141 | Alb | albumin |
| ENSMUSG00000029376 | 1.931 | 1.418 | 0.447 | 0.043 | K13403 | Mthfd2l | methylenetetrahydrofolate dehydrogenase (NADP+ dependent) 2-like |
| ENSMUSG00000029408 | 2.044 | 3.004 | -0.552 | 0.003 | K05656 | Abcb9 | ATP-binding cassette, sub-family B (MDR/TAP), member 9 |
| ENSMUSG00000029436 | 0.725 | 1.152 | -0.666 | 0.003 | K07997 | Mmp17 | matrix metallopeptidase 17 |
| ENSMUSG00000029452 | 2.913 | 3.888 | -0.412 | 0.044 | None | Tmem116 | transmembrane protein 116 |
| ENSMUSG00000029455 | 102.315 | 135.684 | -0.405 | 0.000 | K00128 | Aldh2 | aldehyde dehydrogenase 2, mitochondrial |
| ENSMUSG00000029469 | 10.175 | 7.791 | 0.388 | 0.041 | K19677 | Ift81 | intraflagellar transport 81 |
| ENSMUSG00000029482 | 1.526 | 2.142 | -0.487 | 0.033 | K01907 | Aacs | acetoacetyl-CoA synthetase |
| ENSMUSG00000029486 | 6.048 | 4.319 | 0.488 | 0.001 | K02863 | Mrpl1 | mitochondrial ribosomal protein L1 |
| ENSMUSG00000029545 | 125.851 | 172.670 | -0.455 | 0.000 | K00248 | Acads | acyl-Coenzyme A dehydrogenase, short chain |
| ENSMUSG00000029575 | 13.322 | 17.479 | -0.390 | 0.004 | K00798 | Mmab | methylmalonic aciduria (cobalamin deficiency) cblB type homolog (human) |
| ENSMUSG00000029581 | 12.092 | 16.874 | -0.479 | 0.002 | K17455 | Fscn1 | fascin actin-bundling protein 1 |
| ENSMUSG00000029598 | 14.681 | 19.227 | -0.387 | 0.001 | None | Plbd2 | phospholipase B domain containing 2 |
| ENSMUSG00000029600 | 6.492 | 10.544 | -0.697 | 0.000 | None | Rita1 | RBPJ interacting and tubulin associated 1 |
| ENSMUSG00000029655 | 2.455 | 1.788 | 0.460 | 0.010 | None | N4bp2l2 | NEDD4 binding protein 2-like 2 |
| ENSMUSG00000029669 | 9.398 | 7.158 | 0.396 | 0.004 | K17355 | Tspan12 | tetraspanin 12 |
| ENSMUSG00000029684 | 8.489 | 6.495 | 0.389 | 0.002 | K05747 | Wasl | Wiskott-Aldrich syndrome-like (human) |
| ENSMUSG00000029716 | 0.244 | 0.031 | 2.940 | 0.019 | None | Tfr2 | transferrin receptor 2 |
| ENSMUSG00000029759 | 9.504 | 6.937 | 0.456 | 0.001 | K01045 | Pon3 | paraoxonase 3 |
| ENSMUSG00000029780 | 37.972 | 28.221 | 0.431 | 0.000 | K01081 | Nt5c3 | 5'-nucleotidase, cytosolic III |
| ENSMUSG00000029787 | 2.243 | 1.588 | 0.500 | 0.026 | None | Avl9 | AVL9 cell migration associated |
| ENSMUSG00000029798 | 0.392 | 0.138 | 1.505 | 0.018 | None | Herc6 | hect domain and RLD 6 |
| ENSMUSG00000029802 | 10.734 | 7.971 | 0.432 | 0.004 | K05681 | Abcg2 | ATP binding cassette subfamily G member 2 (Junior blood group) |
| ENSMUSG00000029810 | 40.095 | 30.623 | 0.394 | 0.014 | None | Tmem176b | transmembrane protein 176B |
| ENSMUSG00000029823 | 19.185 | 13.138 | 0.550 | 0.000 | K13212 | Luc7l2 | LUC7-like 2 (S. cerevisiae) |
| ENSMUSG00000029836 | 7.353 | 4.802 | 0.619 | 0.002 | K11586 | Cbx3 | chromobox 3 |
| ENSMUSG00000029915 | 0.672 | 0.191 | 1.811 | 0.001 | K10073 | Clec5a | C-type lectin domain family 5, member a |
| ENSMUSG00000030061 | 14.033 | 10.641 | 0.401 | 0.005 | K10686 | Uba3 | ubiquitin-like modifier activating enzyme 3 |
| ENSMUSG00000030084 | 2.315 | 3.061 | -0.401 | 0.006 | K06820 | Plxna1 | plexin A1 |
| ENSMUSG00000030086 | 3.660 | 4.868 | -0.407 | 0.028 | K17564 | Chchd6 | coiled-coil-helix-coiled-coil-helix domain containing 6 |
| ENSMUSG00000030103 | 16.354 | 11.494 | 0.512 | 0.001 | K03729 | Bhlhe40 | basic helix-loop-helix family, member e40 |
| ENSMUSG00000030144 | 1.871 | 0.567 | 1.724 | 0.007 | K10058 | Clec4d | C-type lectin domain family 4, member d |
| ENSMUSG00000030148 | 1.772 | 0.772 | 1.205 | 0.008 | K10057 | Clec4a2 | C-type lectin domain family 4, member a2 |
| ENSMUSG00000030187 | 0.382 | 0.070 | 2.429 | 0.016 | None | Klra2 | killer cell lectin-like receptor, subfamily A, member 2 |
| ENSMUSG00000030245 | 6.063 | 4.024 | 0.595 | 0.000 | None | Golt1b | golgi transport 1B |
| ENSMUSG00000030254 | 1.126 | 0.659 | 0.775 | 0.022 | K10627 | Rad18 | RAD18 E3 ubiquitin protein ligase |
| ENSMUSG00000030359 | 0.803 | 0.005 | 7.456 | 0.025 | None | Pzp | PZP, alpha-2-macroglobulin like |
| ENSMUSG00000030401 | 27.303 | 38.225 | -0.483 | 0.000 | None | Rtn2 | reticulon 2 (Z-band associated protein) |
| ENSMUSG00000030409 | 158.587 | 207.465 | -0.386 | 0.000 | K08788 | Dmpk | dystrophia myotonica-protein kinase |
| ENSMUSG00000030410 | 33.324 | 46.277 | -0.471 | 0.000 | None | Dmwd | dystrophia myotonica-containing WD repeat motif |
| ENSMUSG00000030465 | 3.077 | 2.125 | 0.537 | 0.003 | K12494 | Psd3 | pleckstrin and Sec7 domain containing 3 |
| ENSMUSG00000030474 | 2.332 | 0.874 | 1.418 | 0.000 | None | Siglece | sialic acid binding Ig-like lectin E |
| ENSMUSG00000030521 | 3.316 | 2.015 | 0.720 | 0.010 | K14559 | Mphosph10 | M-phase phosphoprotein 10 (U3 small nucleolar ribonucleoprotein) |
| ENSMUSG00000030553 | 0.098 | 0.598 | -2.611 | 0.004 | None | Pgpep1l | pyroglutamyl-peptidase I-like |
| ENSMUSG00000030557 | 1.558 | 1.047 | 0.574 | 0.016 | K09260 | Mef2a | myocyte enhancer factor 2A |
| ENSMUSG00000030560 | 16.534 | 11.014 | 0.589 | 0.000 | K01275 | Ctsc | cathepsin C |
| ENSMUSG00000030579 | 29.679 | 20.768 | 0.520 | 0.017 | K07992 | Tyrobp | TYRO protein tyrosine kinase binding protein |
| ENSMUSG00000030588 | 30.226 | 41.518 | -0.455 | 0.000 | K20362 | Yif1b | Yip1 interacting factor homolog B (S. cerevisiae) |
| ENSMUSG00000030595 | 11.435 | 16.243 | -0.504 | 0.000 | K02581 | Nfkbib | nuclear factor of kappa light polypeptide gene enhancer in B cells inhibitor, beta |
| ENSMUSG00000030630 | 8.891 | 16.155 | -0.859 | 0.000 | K01555 | Fah | fumarylacetoacetate hydrolase |
| ENSMUSG00000030660 | 1.939 | 1.256 | 0.628 | 0.002 | K00923 | Pik3c2a | phosphatidylinositol-4-phosphate 3-kinase catalytic subunit type 2 alpha |
| ENSMUSG00000030683 | 0.514 | 0.942 | -0.872 | 0.004 | None | Sez6l2 | seizure related 6 homolog like 2 |
| ENSMUSG00000030699 | 0.253 | 0.608 | -1.265 | 0.037 | K10180 | Tbx6 | T-box 6 |
| ENSMUSG00000030704 | 18.693 | 14.343 | 0.385 | 0.000 | K07893 | Rab6a | RAB6A, member RAS oncogene family |
| ENSMUSG00000030739 | 27.068 | 37.117 | -0.456 | 0.000 | K10352 | Myh14 | myosin, heavy polypeptide 14 |
| ENSMUSG00000030747 | 154.670 | 202.228 | -0.386 | 0.000 | K11160 | Dgat2 | diacylglycerol O-acyltransferase 2 |
| ENSMUSG00000030752 | 3.074 | 4.485 | -0.543 | 0.028 | K10277 | Kdm8 | lysine (K)-specific demethylase 8 |
| ENSMUSG00000030760 | 0.736 | 0.512 | 0.526 | 0.028 | K04711 | Acer3 | alkaline ceramidase 3 |
| ENSMUSG00000030787 | 18.990 | 11.918 | 0.675 | 0.000 | K19012 | Lyve1 | lymphatic vessel endothelial hyaluronan receptor 1 |
| ENSMUSG00000030788 | 9.839 | 7.550 | 0.384 | 0.001 | None | Rnf141 | ring finger protein 141 |
| ENSMUSG00000030826 | 42.212 | 60.298 | -0.513 | 0.000 | K00826 | Bcat2 | branched chain aminotransferase 2, mitochondrial |
| ENSMUSG00000030830 | 1.771 | 0.943 | 0.914 | 0.010 | K05718 | Itgal | integrin alpha L |
| ENSMUSG00000030839 | 1.476 | 2.217 | -0.584 | 0.014 | K15421 | Sergef | secretion regulating guanine nucleotide exchange factor |
| ENSMUSG00000030846 | 6.508 | 4.591 | 0.507 | 0.000 | K13201 | Tial1 | Tia1 cytotoxic granule-associated RNA binding protein-like 1 |
| ENSMUSG00000030895 | 6.491 | 0.157 | 5.369 | 0.037 | K18977 | Hpx | hemopexin |
| ENSMUSG00000030921 | 3.990 | 2.855 | 0.485 | 0.012 | None | Trim30a | tripartite motif-containing 30A |
| ENSMUSG00000030922 | 0.807 | 0.455 | 0.829 | 0.004 | None | Lyrm1 | LYR motif containing 1 |
| ENSMUSG00000031070 | 1.712 | 3.036 | -0.825 | 0.001 | K08394 | Mrgprf | MAS-related GPR, member F |
| ENSMUSG00000031079 | 0.090 | 0.006 | 3.919 | 0.012 | K09228 | Zfp300 | zinc finger protein 300 |
| ENSMUSG00000031111 | 2.691 | 3.618 | -0.426 | 0.024 | None | Igsf1 | immunoglobulin superfamily, member 1 |
| ENSMUSG00000031134 | 13.662 | 10.200 | 0.425 | 0.002 | K12885 | Rbmx | RNA binding motif protein, X chromosome |
| ENSMUSG00000031155 | 1.665 | 2.455 | -0.559 | 0.036 | K08806 | Pim2 | proviral integration site 2 |
| ENSMUSG00000031156 | 3.936 | 5.580 | -0.501 | 0.001 | K15272 | Slc35a2 | solute carrier family 35 (UDP-galactose transporter), member A2 |
| ENSMUSG00000031157 | 11.447 | 15.875 | -0.469 | 0.001 | K12865 | Pqbp1 | polyglutamine binding protein 1 |
| ENSMUSG00000031173 | 0.208 | 0.007 | 4.997 | 0.006 | K00611 | Otc | ornithine transcarbamylase |
| ENSMUSG00000031197 | 13.984 | 8.547 | 0.716 | 0.005 | None | Vbp1 | von Hippel-Lindau binding protein 1 |
| ENSMUSG00000031204 | 6.498 | 9.458 | -0.539 | 0.001 | K10334 | Asb12 | ankyrin repeat and SOCS box-containing 12 |
| ENSMUSG00000031232 | 4.721 | 3.525 | 0.424 | 0.038 | K19478 | Magt1 | magnesium transporter 1 |
| ENSMUSG00000031246 | 12.679 | 8.565 | 0.571 | 0.002 | None | Sh3bgrl | SH3-binding domain glutamic acid-rich protein like |
| ENSMUSG00000031278 | 2.519 | 1.916 | 0.397 | 0.046 | K01897 | Acsl4 | acyl-CoA synthetase long-chain family member 4 |
| ENSMUSG00000031309 | 6.212 | 4.687 | 0.409 | 0.003 | K04373 | Rps6ka3 | ribosomal protein S6 kinase polypeptide 3 |
| ENSMUSG00000031333 | 3.984 | 2.816 | 0.503 | 0.004 | K05662 | Abcb7 | ATP-binding cassette, sub-family B (MDR/TAP), member 7 |
| ENSMUSG00000031342 | 4.821 | 3.301 | 0.549 | 0.000 | None | Gpm6b | glycoprotein m6b |
| ENSMUSG00000031347 | 12.493 | 9.099 | 0.460 | 0.001 | K10840 | Cetn2 | centrin 2 |
| ENSMUSG00000031389 | 0.489 | 0.259 | 0.914 | 0.037 | K20122 | Arhgap4 | Rho GTPase activating protein 4 |
| ENSMUSG00000031444 | 0.577 | 0.071 | 3.011 | 0.001 | K01314 | F10 | coagulation factor X |
| ENSMUSG00000031445 | 0.394 | 0.123 | 1.679 | 0.019 | None | Proz | protein Z, vitamin K-dependent plasma glycoprotein |
| ENSMUSG00000031458 | 3.450 | 4.514 | -0.386 | 0.040 | None | Coprs | coordinator of PRMT5, differentiation stimulator |
| ENSMUSG00000031488 | 0.857 | 0.544 | 0.657 | 0.014 | K12484 | Rab11fip1 | RAB11 family interacting protein 1 (class I) |
| ENSMUSG00000031548 | 11.019 | 8.099 | 0.448 | 0.000 | K02166 | Sfrp1 | secreted frizzled-related protein 1 |
| ENSMUSG00000031594 | 0.299 | 0.051 | 2.533 | 0.018 | None | Fgl1 | fibrinogen-like protein 1 |
| ENSMUSG00000031596 | 1.071 | 0.765 | 0.487 | 0.037 | K13864 | Slc7a2 | solute carrier family 7 (cationic amino acid transporter, y+ system), member 2 |
| ENSMUSG00000031613 | 10.735 | 7.615 | 0.498 | 0.004 | K00069 | Hpgd | hydroxyprostaglandin dehydrogenase 15 (NAD) |
| ENSMUSG00000031626 | 25.434 | 16.798 | 0.601 | 0.000 | None | Sorbs2 | sorbin and SH3 domain containing 2 |
| ENSMUSG00000031673 | 3.657 | 2.474 | 0.566 | 0.046 | K06803 | Cdh11 | cadherin 11 |
| ENSMUSG00000031749 | 9.166 | 12.570 | -0.453 | 0.000 | K03368 | St3gal2 | ST3 beta-galactoside alpha-2,3-sialyltransferase 2 |
| ENSMUSG00000031765 | 165.007 | 233.482 | -0.498 | 0.000 | K14739 | Mt1 | metallothionein 1 |
| ENSMUSG00000031781 | 9.472 | 12.452 | -0.392 | 0.001 | None | Ciapin1 | cytokine induced apoptosis inhibitor 1 |
| ENSMUSG00000031808 | 112.181 | 154.676 | -0.462 | 0.000 | K08745 | Slc27a1 | solute carrier family 27 (fatty acid transporter), member 1 |
| ENSMUSG00000031832 | 2.037 | 2.880 | -0.496 | 0.029 | K15214 | Taf1c | TATA-box binding protein associated factor, RNA polymerase I, C |
| ENSMUSG00000031840 | 0.079 | 1.149 | -3.859 | 0.000 | K07882 | Rab3a | RAB3A, member RAS oncogene family |
| ENSMUSG00000031872 | 0.174 | 0.429 | -1.300 | 0.028 | K19324 | Bean1 | brain expressed, associated with Nedd4, 1 |
| ENSMUSG00000031878 | 6.385 | 4.418 | 0.534 | 0.000 | K04532 | Nae1 | NEDD8 activating enzyme E1 subunit 1 |
| ENSMUSG00000031939 | 1.287 | 0.787 | 0.713 | 0.009 | K15215 | Taf1d | TATA-box binding protein associated factor, RNA polymerase I, D |
| ENSMUSG00000031951 | 0.932 | 1.418 | -0.605 | 0.032 | K19362 | Tmem231 | transmembrane protein 231 |
| ENSMUSG00000031958 | 11.956 | 16.006 | -0.419 | 0.000 | K00102 | Ldhd | lactate dehydrogenase D |
| ENSMUSG00000031965 | 14.472 | 10.935 | 0.407 | 0.005 | K10185 | Tbx20 | T-box 20 |
| ENSMUSG00000031982 | 4.717 | 6.470 | -0.453 | 0.030 | None | Arv1 | ARV1 homolog, fatty acid homeostasis modulator |
| ENSMUSG00000031994 | 0.819 | 0.178 | 2.208 | 0.000 | K08623 | Adamts8 | a disintegrin-like and metallopeptidase (reprolysin type) with thrombospondin type 1 motif, 8 |
| ENSMUSG00000032014 | 4.769 | 6.507 | -0.446 | 0.006 | None | Oaf | out at first homolog |
| ENSMUSG00000032064 | 2.944 | 2.171 | 0.442 | 0.010 | None | Dixdc1 | DIX domain containing 1 |
| ENSMUSG00000032083 | 10.276 | 0.092 | 6.812 | 0.002 | K08757 | Apoa1 | apolipoprotein A-I |
| ENSMUSG00000032109 | 5.336 | 6.946 | -0.379 | 0.025 | K12653 | Nlrx1 | NLR family member X1 |
| ENSMUSG00000032123 | 6.985 | 9.104 | -0.379 | 0.032 | K01001 | Dpagt1 | dolichyl-phosphate (UDP-N-acetylglucosamine) acetylglucosaminephosphotransferase 1 (GlcNAc-1-P transferase) |
| ENSMUSG00000032212 | 5.988 | 3.781 | 0.666 | 0.001 | None | Sltm | SAFB-like, transcription modulator |
| ENSMUSG00000032224 | 1.028 | 1.508 | -0.551 | 0.033 | None | Fam81a | family with sequence similarity 81, member A |
| ENSMUSG00000032238 | 2.788 | 1.881 | 0.572 | 0.001 | K08532 | Rora | RAR-related orphan receptor alpha |
| ENSMUSG00000032253 | 1.639 | 1.118 | 0.557 | 0.033 | K11797 | Phip | pleckstrin homology domain interacting protein |
| ENSMUSG00000032263 | 22.150 | 29.674 | -0.420 | 0.000 | K00167 | Bckdhb | branched chain ketoacid dehydrogenase E1, beta polypeptide |
| ENSMUSG00000032265 | 4.975 | 3.732 | 0.418 | 0.018 | None | Fam46a | family with sequence similarity 46, member A |
| ENSMUSG00000032293 | 4.972 | 3.717 | 0.423 | 0.003 | None | Ireb2 | iron responsive element binding protein 2 |
| ENSMUSG00000032315 | 22.884 | 9.551 | 1.260 | 0.041 | K07408 | Cyp1a1 | cytochrome P450, family 1, subfamily a, polypeptide 1 |
| ENSMUSG00000032372 | 23.644 | 18.199 | 0.381 | 0.044 | None | Plscr2 | phospholipid scramblase 2 |
| ENSMUSG00000032374 | 6.264 | 4.289 | 0.549 | 0.000 | K13645 | Plod2 | procollagen lysine, 2-oxoglutarate 5-dioxygenase 2 |
| ENSMUSG00000032407 | 3.390 | 2.299 | 0.563 | 0.000 | K12842 | U2surp | U2 snRNP-associated SURP domain containing |
| ENSMUSG00000032422 | 7.473 | 5.276 | 0.505 | 0.000 | K17926 | Snx14 | sorting nexin 14 |
| ENSMUSG00000032478 | 9.030 | 12.488 | -0.466 | 0.008 | K00940 | Nme6 | NME/NM23 nucleoside diphosphate kinase 6 |
| ENSMUSG00000032484 | 0.966 | 0.042 | 4.439 | 0.000 | None | Ngp | neutrophilic granule protein |
| ENSMUSG00000032525 | 3.250 | 2.377 | 0.454 | 0.008 | K12740 | Nktr | natural killer tumor recognition sequence |
| ENSMUSG00000032527 | 60.115 | 80.791 | -0.425 | 0.000 | K01966 | Pccb | propionyl Coenzyme A carboxylase, beta polypeptide |
| ENSMUSG00000032578 | 2.847 | 1.690 | 0.755 | 0.014 | K04701 | Cish | cytokine inducible SH2-containing protein |
| ENSMUSG00000032661 | 0.419 | 0.185 | 1.174 | 0.017 | K14216 | Oas3 | 2'-5' oligoadenylate synthetase 3 |
| ENSMUSG00000032666 | 4.695 | 3.582 | 0.393 | 0.005 | None | 1700025G04Rik | RIKEN cDNA 1700025G04 gene |
| ENSMUSG00000032671 | 0.026 | 0.382 | -3.915 | 0.022 | None | A930018P22Rik | RIKEN cDNA A930018P22 gene |
| ENSMUSG00000032741 | 11.788 | 15.748 | -0.415 | 0.001 | K16896 | Tpcn1 | two pore channel 1 |
| ENSMUSG00000032744 | 7.301 | 10.396 | -0.507 | 0.000 | K09091 | Heyl | hairy/enhancer-of-split related with YRPW motif-like |
| ENSMUSG00000032802 | 8.676 | 13.151 | -0.597 | 0.000 | K12260 | Srxn1 | sulfiredoxin 1 homolog (S. cerevisiae) |
| ENSMUSG00000032839 | 2.522 | 1.921 | 0.395 | 0.025 | K04964 | Trpc1 | transient receptor potential cation channel, subfamily C, member 1 |
| ENSMUSG00000032860 | 13.213 | 17.276 | -0.385 | 0.004 | K04269 | P2ry2 | purinergic receptor P2Y, G-protein coupled 2 |
| ENSMUSG00000032915 | 0.471 | 0.097 | 2.258 | 0.000 | K08445 | Adgre4 | adhesion G protein-coupled receptor E4 |
| ENSMUSG00000032997 | 7.324 | 10.355 | -0.498 | 0.001 | K00747 | Chpf | chondroitin polymerizing factor |
| ENSMUSG00000033007 | 0.187 | 0.468 | -1.323 | 0.037 | K04831 | Asic4 | acid-sensing (proton-gated) ion channel family member 4 |
| ENSMUSG00000033082 | 3.532 | 2.537 | 0.480 | 0.007 | K10069 | Clec1a | C-type lectin domain family 1, member a |
| ENSMUSG00000033124 | 11.619 | 15.540 | -0.418 | 0.001 | K17907 | Atg9a | autophagy related 9A |
| ENSMUSG00000033152 | 3.520 | 5.234 | -0.569 | 0.002 | K06818 | Podxl2 | podocalyxin-like 2 |
| ENSMUSG00000033184 | 7.399 | 5.594 | 0.406 | 0.000 | K20349 | Tmed7 | transmembrane p24 trafficking protein 7 |
| ENSMUSG00000033186 | 18.896 | 12.031 | 0.655 | 0.000 | K18633 | Mzt1 | mitotic spindle organizing protein 1 |
| ENSMUSG00000033220 | 4.958 | 3.230 | 0.624 | 0.019 | K07860 | Rac2 | RAS-related C3 botulinum substrate 2 |
| ENSMUSG00000033249 | 3.693 | 5.126 | -0.470 | 0.034 | K09417 | Hsf4 | heat shock transcription factor 4 |
| ENSMUSG00000033306 | 2.904 | 2.084 | 0.483 | 0.003 | K16676 | Lpp | LIM domain containing preferred translocation partner in lipoma |
| ENSMUSG00000033313 | 1.396 | 2.236 | -0.677 | 0.015 | K10274 | Fbxl8 | F-box and leucine-rich repeat protein 8 |
| ENSMUSG00000033355 | 7.858 | 5.531 | 0.510 | 0.014 | None | Rtp4 | receptor transporter protein 4 |
| ENSMUSG00000033364 | 1.380 | 1.026 | 0.430 | 0.040 | K11850 | Usp37 | ubiquitin specific peptidase 37 |
| ENSMUSG00000033365 | 20.941 | 28.287 | -0.432 | 0.000 | None | Ipo13 | importin 13 |
| ENSMUSG00000033405 | 1.669 | 1.119 | 0.578 | 0.027 | K03574 | Nudt15 | nudix (nucleoside diphosphate linked moiety X)-type motif 15 |
| ENSMUSG00000033411 | 2.240 | 1.653 | 0.441 | 0.022 | K17616 | Ctdspl2 | CTD (carboxy-terminal domain, RNA polymerase II, polypeptide A) small phosphatase like 2 |
| ENSMUSG00000033439 | 2.029 | 1.401 | 0.538 | 0.019 | K15446 | Trmt13 | tRNA methyltransferase 13 |
| ENSMUSG00000033487 | 5.006 | 3.715 | 0.433 | 0.005 | None | Fndc3a | fibronectin type III domain containing 3A |
| ENSMUSG00000033634 | 0.417 | 0.000 | 8.030 | 0.010 | None | Nat8f2 | N-acetyltransferase 8 (GCN5-related) family member 2 |
| ENSMUSG00000033688 | 0.590 | 0.059 | 3.308 | 0.001 | K14736 | 1300017J02Rik | RIKEN cDNA 1300017J02 gene |
| ENSMUSG00000033697 | 1.778 | 2.780 | -0.641 | 0.000 | None | Arhgap39 | Rho GTPase activating protein 39 |
| ENSMUSG00000033751 | 7.375 | 10.395 | -0.493 | 0.000 | None | Gadd45gip1 | growth arrest and DNA-damage-inducible, gamma interacting protein 1 |
| ENSMUSG00000033763 | 9.915 | 13.119 | -0.402 | 0.000 | K20128 | Mtss1l | metastasis suppressor 1-like |
| ENSMUSG00000033777 | 1.571 | 0.665 | 1.246 | 0.006 | K18808 | Tlr13 | toll-like receptor 13 |
| ENSMUSG00000033790 | 2.724 | 1.936 | 0.496 | 0.005 | K16572 | Tubgcp5 | tubulin, gamma complex associated protein 5 |
| ENSMUSG00000033808 | 3.636 | 2.745 | 0.408 | 0.012 | None | Tmem87a | transmembrane protein 87A |
| ENSMUSG00000033813 | 7.412 | 4.970 | 0.579 | 0.000 | K03145 | Tcea1 | transcription elongation factor A (SII) 1 |
| ENSMUSG00000033819 | 6.678 | 9.143 | -0.451 | 0.012 | K17458 | Ppp1r16a | protein phosphatase 1, regulatory (inhibitor) subunit 16A |
| ENSMUSG00000033831 | 1.946 | 0.002 | 9.498 | 0.005 | K03904 | Fgb | fibrinogen beta chain |
| ENSMUSG00000033860 | 2.227 | 0.000 | 9.525 | 0.003 | K03905 | Fgg | fibrinogen gamma chain |
| ENSMUSG00000033898 | 0.595 | 0.105 | 2.492 | 0.023 | None | Cfhr2 | complement factor H-related 2 |
| ENSMUSG00000033943 | 3.206 | 2.154 | 0.577 | 0.000 | None | Mga | MAX gene associated |
| ENSMUSG00000033991 | 2.196 | 1.676 | 0.391 | 0.044 | K12600 | Ttc37 | tetratricopeptide repeat domain 37 |
| ENSMUSG00000034042 | 10.037 | 7.415 | 0.439 | 0.001 | None | Gpbp1l1 | GC-rich promoter binding protein 1-like 1 |
| ENSMUSG00000034083 | 1.893 | 1.275 | 0.572 | 0.005 | None | Ccdc174 | coiled-coil domain containing 174 |
| ENSMUSG00000034105 | 6.212 | 8.846 | -0.508 | 0.001 | None | Tldc1 | TBC/LysM associated domain containing 1 |
| ENSMUSG00000034116 | 2.280 | 1.506 | 0.602 | 0.037 | K05730 | Vav1 | vav 1 oncogene |
| ENSMUSG00000034121 | 2.315 | 3.183 | -0.457 | 0.048 | K19332 | Mks1 | Meckel syndrome, type 1 |
| ENSMUSG00000034158 | 7.787 | 5.901 | 0.403 | 0.002 | None | Lrrc58 | leucine rich repeat containing 58 |
| ENSMUSG00000034245 | 10.983 | 14.622 | -0.411 | 0.003 | K11418 | Hdac11 | histone deacetylase 11 |
| ENSMUSG00000034252 | 7.404 | 5.551 | 0.420 | 0.009 | K08595 | Senp6 | SUMO/sentrin specific peptidase 6 |
| ENSMUSG00000034275 | 0.181 | 0.283 | -0.643 | 0.024 | None | Igsf9b | immunoglobulin superfamily, member 9B |
| ENSMUSG00000034303 | 0.686 | 0.307 | 1.157 | 0.015 | K16752 | Ccdc15 | coiled-coil domain containing 15 |
| ENSMUSG00000034349 | 1.726 | 1.066 | 0.699 | 0.031 | K06675 | Smc4 | structural maintenance of chromosomes 4 |
| ENSMUSG00000034438 | 1.867 | 1.145 | 0.707 | 0.041 | None | Gbp8 | guanylate-binding protein 8 |
| ENSMUSG00000034472 | 5.435 | 7.410 | -0.444 | 0.007 | K07844 | Rasd2 | RASD family, member 2 |
| ENSMUSG00000034528 | 0.355 | 0.013 | 4.907 | 0.014 | None | Hsd17b13 | hydroxysteroid (17-beta) dehydrogenase 13 |
| ENSMUSG00000034593 | 1.694 | 1.111 | 0.611 | 0.000 | K10357 | Myo5a | myosin VA |
| ENSMUSG00000034601 | 1.312 | 0.935 | 0.491 | 0.028 | None | 2700049A03Rik | RIKEN cDNA 2700049A03 gene |
| ENSMUSG00000034623 | 0.000 | 0.148 | -4.777 | 0.007 | None | Prss55 | protease, serine 55 |
| ENSMUSG00000034640 | 3.005 | 1.955 | 0.622 | 0.003 | K15259 | Tiparp | TCDD-inducible poly(ADP-ribose) polymerase |
| ENSMUSG00000034685 | 2.365 | 3.176 | -0.424 | 0.034 | None | Fam171a2 | family with sequence similarity 171, member A2 |
| ENSMUSG00000034687 | 0.250 | 0.099 | 1.336 | 0.010 | None | Fras1 | Fraser extracellular matrix complex subunit 1 |
| ENSMUSG00000034706 | 0.074 | 0.321 | -2.098 | 0.018 | K11143 | Dnaic2 | dynein, axonemal, intermediate chain 2 |
| ENSMUSG00000034724 | 3.907 | 2.847 | 0.459 | 0.007 | K12603 | Cnot6l | CCR4-NOT transcription complex, subunit 6-like |
| ENSMUSG00000034748 | 3.988 | 5.291 | -0.405 | 0.046 | K11416 | Sirt6 | sirtuin 6 |
| ENSMUSG00000034758 | 2.317 | 3.291 | -0.504 | 0.035 | None | Tle6 | transducin-like enhancer of split 6 |
| ENSMUSG00000034799 | 0.236 | 0.423 | -0.840 | 0.015 | K15293 | Unc13a | unc-13 homolog A (C. elegans) |
| ENSMUSG00000034807 | 21.070 | 27.983 | -0.407 | 0.000 | K11703 | Colgalt1 | collagen beta(1-O)galactosyltransferase 1 |
| ENSMUSG00000034810 | 6.026 | 4.137 | 0.546 | 0.000 | K04839 | Scn7a | sodium channel, voltage-gated, type VII, alpha |
| ENSMUSG00000034863 | 4.364 | 6.091 | -0.480 | 0.000 | K19502 | Ano8 | anoctamin 8 |
| ENSMUSG00000035000 | 1.279 | 0.826 | 0.631 | 0.025 | K01278 | Dpp4 | dipeptidylpeptidase 4 |
| ENSMUSG00000035151 | 1.934 | 1.296 | 0.579 | 0.016 | None | Elmod2 | ELMO/CED-12 domain containing 2 |
| ENSMUSG00000035173 | 3.836 | 2.864 | 0.425 | 0.035 | None | Ccdc186 | coiled-coil domain containing 186 |
| ENSMUSG00000035273 | 1.647 | 1.020 | 0.692 | 0.025 | K07964 | Hpse | heparanase |
| ENSMUSG00000035352 | 3.228 | 0.919 | 1.812 | 0.001 | K14624 | Ccl12 | chemokine (C-C motif) ligand 12 |
| ENSMUSG00000035356 | 2.142 | 1.532 | 0.486 | 0.023 | K14242 | Nfkbiz | nuclear factor of kappa light polypeptide gene enhancer in B cells inhibitor, zeta |
| ENSMUSG00000035373 | 3.624 | 1.092 | 1.732 | 0.007 | K05509 | Ccl7 | chemokine (C-C motif) ligand 7 |
| ENSMUSG00000035486 | 0.622 | 1.228 | -0.983 | 0.032 | K19596 | Plk5 | polo like kinase 5 |
| ENSMUSG00000035506 | 0.568 | 0.244 | 1.232 | 0.013 | K14428 | Slc12a8 | solute carrier family 12 (potassium/chloride transporters), member 8 |
| ENSMUSG00000035540 | 5.269 | 0.047 | 6.803 | 0.009 | K12258 | Gc | group specific component |
| ENSMUSG00000035757 | 4.497 | 6.571 | -0.545 | 0.001 | None | Selenoo | selenoprotein O |
| ENSMUSG00000035762 | 2.831 | 1.859 | 0.609 | 0.006 | None | Tmem161b | transmembrane protein 161B |
| ENSMUSG00000035798 | 4.843 | 3.673 | 0.401 | 0.014 | K20032 | Zdhhc17 | zinc finger, DHHC domain containing 17 |
| ENSMUSG00000035834 | 1.607 | 0.782 | 1.046 | 0.001 | K03024 | Polr3g | polymerase (RNA) III (DNA directed) polypeptide G |
| ENSMUSG00000035851 | 4.817 | 3.647 | 0.404 | 0.013 | K20100 | Ythdc1 | YTH domain containing 1 |
| ENSMUSG00000035944 | 8.594 | 12.935 | -0.589 | 0.000 | None | Ttc38 | tetratricopeptide repeat domain 38 |
| ENSMUSG00000035967 | 2.032 | 1.363 | 0.580 | 0.014 | K13180 | Ints6l | integrator complex subunit 6 like |
| ENSMUSG00000035992 | 6.380 | 4.162 | 0.621 | 0.001 | K20400 | Fnip1 | folliculin interacting protein 1 |
| ENSMUSG00000036019 | 1.967 | 1.467 | 0.426 | 0.037 | None | Tmtc2 | transmembrane and tetratricopeptide repeat containing 2 |
| ENSMUSG00000036053 | 2.380 | 1.808 | 0.400 | 0.024 | None | Fmnl2 | formin-like 2 |
| ENSMUSG00000036067 | 0.462 | 0.186 | 1.317 | 0.027 | K08144 | Slc2a6 | solute carrier family 2 (facilitated glucose transporter), member 6 |
| ENSMUSG00000036093 | 6.390 | 4.442 | 0.527 | 0.000 | K07949 | Arl5a | ADP-ribosylation factor-like 5A |
| ENSMUSG00000036097 | 4.543 | 3.304 | 0.462 | 0.006 | None | Slf2 | SMC5-SMC6 complex localization factor 2 |
| ENSMUSG00000036144 | 3.358 | 4.532 | -0.430 | 0.028 | K09322 | Meox2 | mesenchyme homeobox 2 |
| ENSMUSG00000036181 | 201.314 | 286.445 | -0.505 | 0.000 | K11275 | Hist1h1c | histone cluster 1, H1c |
| ENSMUSG00000036202 | 1.504 | 0.965 | 0.645 | 0.027 | K11138 | Rif1 | replication timing regulatory factor 1 |
| ENSMUSG00000036256 | 100.037 | 135.786 | -0.438 | 0.000 | None | Igfbp7 | insulin-like growth factor binding protein 7 |
| ENSMUSG00000036292 | 0.401 | 0.138 | 1.536 | 0.021 | None | Gramd1c | GRAM domain containing 1C |
| ENSMUSG00000036334 | 0.668 | 0.484 | 0.466 | 0.038 | None | Igsf10 | immunoglobulin superfamily, member 10 |
| ENSMUSG00000036371 | 13.388 | 10.088 | 0.411 | 0.000 | K13199 | Serbp1 | serpine1 mRNA binding protein 1 |
| ENSMUSG00000036446 | 36.649 | 27.488 | 0.418 | 0.000 | K08122 | Lum | lumican |
| ENSMUSG00000036469 | 1.093 | 0.488 | 1.167 | 0.006 | K10656 | 1-Mar | membrane-associated ring finger (C3HC4) 1 |
| ENSMUSG00000036499 | 3.623 | 2.438 | 0.577 | 0.008 | K12478 | Eea1 | early endosome antigen 1 |
| ENSMUSG00000036501 | 4.251 | 2.793 | 0.609 | 0.001 | None | Fam13b | family with sequence similarity 13, member B |
| ENSMUSG00000036743 | 0.595 | 0.159 | 1.880 | 0.005 | K02731 | Psma8 | proteasome (prosome, macropain) subunit, alpha type, 8 |
| ENSMUSG00000036779 | 6.080 | 4.518 | 0.431 | 0.005 | K03514 | Papd5 | PAP associated domain containing 5 |
| ENSMUSG00000036833 | 10.745 | 16.724 | -0.637 | 0.000 | K14676 | Pnpla7 | patatin-like phospholipase domain containing 7 |
| ENSMUSG00000036863 | 5.410 | 4.039 | 0.424 | 0.014 | None | Syde2 | synapse defective 1, Rho GTPase, homolog 2 (C. elegans) |
| ENSMUSG00000036864 | 0.673 | 1.250 | -0.887 | 0.005 | None | Proser3 | proline and serine rich 3 |
| ENSMUSG00000036887 | 99.908 | 60.537 | 0.729 | 0.032 | K03986 | C1qa | complement component 1, q subcomponent, alpha polypeptide |
| ENSMUSG00000036894 | 0.464 | 0.257 | 0.855 | 0.010 | K07838 | Rap2b | RAP2B, member of RAS oncogene family |
| ENSMUSG00000036896 | 69.600 | 45.457 | 0.620 | 0.046 | K03988 | C1qc | complement component 1, q subcomponent, C chain |
| ENSMUSG00000036898 | 1.314 | 0.901 | 0.548 | 0.035 | K09228 | Zfp157 | zinc finger protein 157 |
| ENSMUSG00000036904 | 2.642 | 3.557 | -0.427 | 0.023 | K02375 | Fzd8 | frizzled class receptor 8 |
| ENSMUSG00000036943 | 5.273 | 3.991 | 0.404 | 0.002 | K07902 | Rab8b | RAB8B, member RAS oncogene family |
| ENSMUSG00000036944 | 5.886 | 4.465 | 0.402 | 0.037 | None | Tmem71 | transmembrane protein 71 |
| ENSMUSG00000037007 | 1.435 | 1.092 | 0.398 | 0.031 | K09228 | Zfp113 | zinc finger protein 113 |
| ENSMUSG00000037035 | 1.799 | 2.928 | -0.701 | 0.000 | K04667 | Inhbb | inhibin beta-B |
| ENSMUSG00000037095 | 67.552 | 91.256 | -0.432 | 0.000 | None | Lrg1 | leucine-rich alpha-2-glycoprotein 1 |
| ENSMUSG00000037166 | 6.820 | 9.466 | -0.470 | 0.028 | K12328 | Ppp1r14a | protein phosphatase 1, regulatory (inhibitor) subunit 14A |
| ENSMUSG00000037234 | 0.936 | 0.612 | 0.615 | 0.014 | K16536 | Hook3 | hook microtubule tethering protein 3 |
| ENSMUSG00000037236 | 14.553 | 8.702 | 0.747 | 0.000 | K13213 | Matr3 | matrin 3 |
| ENSMUSG00000037286 | 5.008 | 3.746 | 0.422 | 0.004 | K06671 | Stag1 | stromal antigen 1 |
| ENSMUSG00000037349 | 15.427 | 22.114 | -0.516 | 0.002 | None | Nudt22 | nudix (nucleoside diphosphate linked moiety X)-type motif 22 |
| ENSMUSG00000037369 | 4.027 | 3.075 | 0.392 | 0.005 | K11447 | Kdm6a | lysine (K)-specific demethylase 6A |
| ENSMUSG00000037400 | 4.078 | 2.862 | 0.514 | 0.001 | K01530 | Atp11b | ATPase, class VI, type 11B |
| ENSMUSG00000037408 | 1.128 | 1.634 | -0.532 | 0.010 | K16302 | Cnnm4 | cyclin M4 |
| ENSMUSG00000037411 | 22.276 | 33.232 | -0.576 | 0.000 | K03982 | Serpine1 | serine (or cysteine) peptidase inhibitor, clade E, member 1 |
| ENSMUSG00000037447 | 2.279 | 3.267 | -0.517 | 0.006 | None | Arid5a | AT rich interactive domain 5A (MRF1-like) |
| ENSMUSG00000037475 | 2.205 | 1.384 | 0.678 | 0.015 | K12879 | Thoc2 | THO complex 2 |
| ENSMUSG00000037523 | 20.805 | 28.207 | -0.436 | 0.000 | K12648 | Mavs | mitochondrial antiviral signaling protein |
| ENSMUSG00000037533 | 3.964 | 2.817 | 0.496 | 0.002 | K08020 | Rapgef6 | Rap guanine nucleotide exchange factor (GEF) 6 |
| ENSMUSG00000037608 | 6.773 | 4.537 | 0.583 | 0.003 | K13087 | Bclaf1 | BCL2-associated transcription factor 1 |
| ENSMUSG00000037674 | 1.298 | 0.957 | 0.443 | 0.015 | None | Rfx7 | regulatory factor X, 7 |
| ENSMUSG00000037703 | 2.091 | 3.216 | -0.619 | 0.000 | None | Lzts3 | leucine zipper, putative tumor suppressor family member 3 |
| ENSMUSG00000037731 | 3.161 | 2.173 | 0.544 | 0.026 | None | Themis2 | thymocyte selection associated family member 2 |
| ENSMUSG00000037761 | 2.789 | 3.656 | -0.387 | 0.042 | K11672 | Actr5 | ARP5 actin-related protein 5 |
| ENSMUSG00000037795 | 1.337 | 0.993 | 0.431 | 0.040 | K15720 | N4bp2 | NEDD4 binding protein 2 |
| ENSMUSG00000037798 | 1.260 | 0.026 | 5.589 | 0.009 | K00789 | Mat1a | methionine adenosyltransferase I, alpha |
| ENSMUSG00000037808 | 4.201 | 2.923 | 0.527 | 0.018 | None | Fam76b | family with sequence similarity 76, member B |
| ENSMUSG00000037860 | 0.810 | 0.370 | 1.137 | 0.044 | K12966 | Aim2 | absent in melanoma 2 |
| ENSMUSG00000037876 | 3.162 | 2.433 | 0.381 | 0.007 | K11449 | Jmjd1c | jumonji domain containing 1C |
| ENSMUSG00000037902 | 14.111 | 10.762 | 0.395 | 0.012 | K06551 | Sirpa | signal-regulatory protein alpha |
| ENSMUSG00000037989 | 14.233 | 18.927 | -0.411 | 0.001 | K08867 | Wnk2 | WNK lysine deficient protein kinase 2 |
| ENSMUSG00000038005 | 5.675 | 3.856 | 0.560 | 0.001 | None | Hpf1 | histone PARylation factor 1 |
| ENSMUSG00000038020 | 0.115 | 0.318 | -1.464 | 0.010 | None | Rapgefl1 | Rap guanine nucleotide exchange factor (GEF)-like 1 |
| ENSMUSG00000038046 | 9.337 | 12.872 | -0.460 | 0.001 | K20095 | Mrm3 | mitochondrial rRNA methyltransferase 3 |
| ENSMUSG00000038068 | 12.692 | 9.636 | 0.399 | 0.004 | K11975 | Rnf144b | ring finger protein 144B |
| ENSMUSG00000038074 | 3.936 | 2.859 | 0.463 | 0.010 | K09577 | Fkbp14 | FK506 binding protein 14 |
| ENSMUSG00000038127 | 6.749 | 4.918 | 0.460 | 0.001 | None | Ccdc50 | coiled-coil domain containing 50 |
| ENSMUSG00000038132 | 63.919 | 47.823 | 0.421 | 0.000 | None | Rbm24 | RNA binding motif protein 24 |
| ENSMUSG00000038147 | 2.019 | 1.057 | 0.937 | 0.000 | K06511 | Cd84 | CD84 antigen |
| ENSMUSG00000038173 | 2.418 | 1.498 | 0.696 | 0.017 | K08743 | Enpp6 | ectonucleotide pyrophosphatase/phosphodiesterase 6 |
| ENSMUSG00000038174 | 0.877 | 0.512 | 0.778 | 0.004 | None | Fam126b | family with sequence similarity 126, member B |
| ENSMUSG00000038178 | 6.279 | 10.254 | -0.706 | 0.000 | K08229 | Slc43a2 | solute carrier family 43, member 2 |
| ENSMUSG00000038201 | 3.612 | 5.101 | -0.496 | 0.041 | K04880 | Kcna7 | potassium voltage-gated channel, shaker-related subfamily, member 7 |
| ENSMUSG00000038224 | 0.541 | 0.004 | 6.854 | 0.042 | K03983 | Serpinf2 | serine (or cysteine) peptidase inhibitor, clade F, member 2 |
| ENSMUSG00000038301 | 1.826 | 1.204 | 0.605 | 0.001 | K17924 | Snx10 | sorting nexin 10 |
| ENSMUSG00000038387 | 47.458 | 61.974 | -0.383 | 0.004 | K07829 | Rras | related RAS viral (r-ras) oncogene |
| ENSMUSG00000038457 | 5.040 | 6.953 | -0.460 | 0.014 | None | Tmem255b | transmembrane protein 255B |
| ENSMUSG00000038486 | 1.355 | 1.901 | -0.485 | 0.036 | K06258 | Sv2a | synaptic vesicle glycoprotein 2 a |
| ENSMUSG00000038502 | 32.632 | 45.067 | -0.465 | 0.000 | None | Ptov1 | prostate tumor over expressed gene 1 |
| ENSMUSG00000038524 | 0.642 | 1.053 | -0.710 | 0.015 | K20125 | Fchsd1 | FCH and double SH3 domains 1 |
| ENSMUSG00000038538 | 1.971 | 1.499 | 0.398 | 0.027 | K17492 | Ubn2 | ubinuclein 2 |
| ENSMUSG00000038594 | 4.232 | 2.839 | 0.579 | 0.008 | K16766 | Cep85l | centrosomal protein 85-like |
| ENSMUSG00000038642 | 28.355 | 16.190 | 0.814 | 0.000 | K01368 | Ctss | cathepsin S |
| ENSMUSG00000038646 | 21.001 | 15.880 | 0.407 | 0.009 | K18708 | Fam103a1 | family with sequence similarity 103, member A1 |
| ENSMUSG00000038668 | 3.077 | 2.268 | 0.442 | 0.011 | K04289 | Lpar1 | lysophosphatidic acid receptor 1 |
| ENSMUSG00000038777 | 2.126 | 3.110 | -0.547 | 0.001 | K06842 | Sema6c | sema domain, transmembrane domain (TM), and cytoplasmic domain, (semaphorin) 6C |
| ENSMUSG00000038838 | 2.927 | 4.045 | -0.465 | 0.004 | K01873 | Vars2 | valyl-tRNA synthetase 2, mitochondrial |
| ENSMUSG00000038843 | 1.904 | 1.100 | 0.793 | 0.002 | K00727 | Gcnt1 | glucosaminyl (N-acetyl) transferase 1, core 2 |
| ENSMUSG00000039005 | 6.236 | 4.723 | 0.404 | 0.046 | K10160 | Tlr4 | toll-like receptor 4 |
| ENSMUSG00000039031 | 6.575 | 5.010 | 0.395 | 0.020 | None | Arhgap18 | Rho GTPase activating protein 18 |
| ENSMUSG00000039068 | 4.301 | 3.252 | 0.406 | 0.002 | None | Zzz3 | zinc finger, ZZ domain containing 3 |
| ENSMUSG00000039109 | 15.112 | 10.040 | 0.593 | 0.000 | K03917 | F13a1 | coagulation factor XIII, A1 subunit |
| ENSMUSG00000039145 | 1.654 | 1.235 | 0.423 | 0.026 | K08794 | Camk1d | calcium/calmodulin-dependent protein kinase ID |
| ENSMUSG00000039219 | 2.161 | 1.372 | 0.663 | 0.041 | K19195 | Arid4b | AT rich interactive domain 4B (RBP1-like) |
| ENSMUSG00000039220 | 7.278 | 9.977 | -0.453 | 0.003 | K17552 | Ppp1r10 | protein phosphatase 1, regulatory subunit 10 |
| ENSMUSG00000039232 | 1.297 | 0.868 | 0.579 | 0.042 | K08487 | Stx11 | syntaxin 11 |
| ENSMUSG00000039285 | 16.447 | 12.488 | 0.400 | 0.002 | K12651 | Azi2 | 5-azacytidine induced gene 2 |
| ENSMUSG00000039304 | 7.638 | 4.623 | 0.728 | 0.000 | K04721 | Tnfsf10 | tumor necrosis factor (ligand) superfamily, member 10 |
| ENSMUSG00000039345 | 7.402 | 9.993 | -0.430 | 0.001 | None | Mettl22 | methyltransferase like 22 |
| ENSMUSG00000039361 | 19.188 | 13.719 | 0.488 | 0.000 | K20044 | Picalm | phosphatidylinositol binding clathrin assembly protein |
| ENSMUSG00000039376 | 44.127 | 32.860 | 0.427 | 0.000 | None | Synpo2l | synaptopodin 2-like |
| ENSMUSG00000039377 | 4.208 | 6.966 | -0.725 | 0.000 | K09339 | Hlx | H2.0-like homeobox |
| ENSMUSG00000039450 | 9.648 | 17.924 | -0.890 | 0.000 | K03331 | Dcxr | dicarbonyl L-xylulose reductase |
| ENSMUSG00000039478 | 1.517 | 0.813 | 0.902 | 0.012 | None | Micu3 | mitochondrial calcium uptake family, member 3 |
| ENSMUSG00000039568 | 5.390 | 7.064 | -0.388 | 0.003 | None | Ubald1 | UBA-like domain containing 1 |
| ENSMUSG00000039607 | 1.479 | 0.972 | 0.610 | 0.032 | None | Rbms3 | RNA binding motif, single stranded interacting protein |
| ENSMUSG00000039629 | 20.388 | 14.243 | 0.518 | 0.000 | None | Strip2 | striatin interacting protein 2 |
| ENSMUSG00000039630 | 0.792 | 0.418 | 0.923 | 0.008 | K12888 | Hnrnpu | heterogeneous nuclear ribonucleoprotein U |
| ENSMUSG00000039639 | 5.147 | 3.161 | 0.707 | 0.001 | K04894 | Kcne1 | potassium voltage-gated channel, Isk-related subfamily, member 1 |
| ENSMUSG00000039715 | 3.716 | 5.589 | -0.585 | 0.001 | None | Wdr34 | WD repeat domain 34 |
| ENSMUSG00000039813 | 1.170 | 1.877 | -0.679 | 0.001 | K20165 | Tbc1d2 | TBC1 domain family, member 2 |
| ENSMUSG00000039831 | 14.111 | 10.873 | 0.379 | 0.001 | None | Arhgap29 | Rho GTPase activating protein 29 |
| ENSMUSG00000039879 | 8.925 | 6.394 | 0.484 | 0.000 | None | Heca | hdc homolog, cell cycle regulator |
| ENSMUSG00000039886 | 13.189 | 18.762 | -0.506 | 0.000 | None | Tmem120a | transmembrane protein 120A |
| ENSMUSG00000039911 | 20.533 | 13.935 | 0.559 | 0.004 | K10343 | Spsb1 | splA/ryanodine receptor domain and SOCS box containing 1 |
| ENSMUSG00000039914 | 89.209 | 120.959 | -0.437 | 0.000 | K18588 | Coq10a | coenzyme Q10A |
| ENSMUSG00000039963 | 0.004 | 0.057 | -3.799 | 0.042 | None | Ccdc40 | coiled-coil domain containing 40 |
| ENSMUSG00000039967 | 1.733 | 0.983 | 0.820 | 0.000 | None | Zfp292 | zinc finger protein 292 |
| ENSMUSG00000039968 | 2.454 | 1.655 | 0.574 | 0.027 | None | Rsbn1l | round spermatid basic protein 1-like |
| ENSMUSG00000040017 | 0.381 | 0.000 | 7.244 | 0.000 | K17310 | Saa4 | serum amyloid A 4 |
| ENSMUSG00000040026 | 2.762 | 0.106 | 4.713 | 0.000 | K17310 | Saa3 | serum amyloid A 3 |
| ENSMUSG00000040146 | 3.492 | 4.564 | -0.384 | 0.041 | K17639 | Rgl3 | ral guanine nucleotide dissociation stimulator-like 3 |
| ENSMUSG00000040253 | 2.711 | 1.585 | 0.778 | 0.002 | None | Gbp7 | guanylate binding protein 7 |
| ENSMUSG00000040260 | 2.642 | 3.445 | -0.381 | 0.007 | K04512 | Daam2 | dishevelled associated activator of morphogenesis 2 |
| ENSMUSG00000040270 | 1.028 | 0.765 | 0.430 | 0.038 | K09042 | Bach2 | BTB and CNC homology, basic leucine zipper transcription factor 2 |
| ENSMUSG00000040289 | 5.159 | 8.031 | -0.638 | 0.001 | K09091 | Hey1 | hairy/enhancer-of-split related with YRPW motif 1 |
| ENSMUSG00000040297 | 2.251 | 1.727 | 0.385 | 0.036 | None | Suco | SUN domain containing ossification factor |
| ENSMUSG00000040321 | 2.973 | 2.097 | 0.506 | 0.012 | None | Zfp770 | zinc finger protein 770 |
| ENSMUSG00000040329 | 0.410 | 0.187 | 1.135 | 0.045 | K05431 | Il7 | interleukin 7 |
| ENSMUSG00000040339 | 7.036 | 4.936 | 0.514 | 0.000 | None | Fam102b | family with sequence similarity 102, member B |
| ENSMUSG00000040350 | 0.937 | 1.377 | -0.553 | 0.025 | K12000 | Trim7 | tripartite motif-containing 7 |
| ENSMUSG00000040459 | 9.260 | 6.549 | 0.504 | 0.004 | K13173 | Arglu1 | arginine and glutamate rich 1 |
| ENSMUSG00000040466 | 9.451 | 13.112 | -0.469 | 0.009 | K05901 | Blvrb | biliverdin reductase B (flavin reductase (NADPH)) |
| ENSMUSG00000040488 | 102.580 | 146.248 | -0.509 | 0.000 | K08023 | Ltbp4 | latent transforming growth factor beta binding protein 4 |
| ENSMUSG00000040528 | 1.334 | 0.573 | 1.220 | 0.012 | None | Milr1 | mast cell immunoglobulin like receptor 1 |
| ENSMUSG00000040532 | 10.833 | 15.737 | -0.536 | 0.000 | K13703 | Abhd11 | abhydrolase domain containing 11 |
| ENSMUSG00000040537 | 1.357 | 1.024 | 0.409 | 0.031 | K16068 | Adam22 | a disintegrin and metallopeptidase domain 22 |
| ENSMUSG00000040543 | 1.075 | 1.440 | -0.420 | 0.021 | None | Pitpnm3 | PITPNM family member 3 |
| ENSMUSG00000040552 | 3.364 | 1.897 | 0.830 | 0.005 | K04009 | C3ar1 | complement component 3a receptor 1 |
| ENSMUSG00000040564 | 3.334 | 0.149 | 4.476 | 0.005 | None | Apoc1 | apolipoprotein C-I |
| ENSMUSG00000040565 | 3.644 | 2.554 | 0.516 | 0.004 | K15192 | Btaf1 | B-TFIID TATA-box binding protein associated factor 1 |
| ENSMUSG00000040613 | 5.896 | 3.734 | 0.665 | 0.025 | K16932 | Apobec1 | apolipoprotein B mRNA editing enzyme, catalytic polypeptide 1 |
| ENSMUSG00000040653 | 41.916 | 28.987 | 0.535 | 0.000 | K17556 | Ppp1r14c | protein phosphatase 1, regulatory (inhibitor) subunit 14c |
| ENSMUSG00000040740 | 152.320 | 236.104 | -0.631 | 0.000 | K15117 | Slc25a34 | solute carrier family 25, member 34 |
| ENSMUSG00000040747 | 7.171 | 3.748 | 0.939 | 0.000 | K06489 | Cd53 | CD53 antigen |
| ENSMUSG00000040751 | 1.413 | 0.690 | 1.040 | 0.023 | None | Lat2 | linker for activation of T cells family, member 2 |
| ENSMUSG00000040760 | 3.034 | 2.293 | 0.408 | 0.018 | K08733 | Appl1 | adaptor protein, phosphotyrosine interaction, PH domain and leucine zipper containing 1 |
| ENSMUSG00000040774 | 6.778 | 4.644 | 0.548 | 0.002 | K13644 | Cept1 | choline/ethanolaminephosphotransferase 1 |
| ENSMUSG00000040875 | 0.733 | 1.729 | -1.238 | 0.000 | K20465 | Osbpl10 | oxysterol binding protein-like 10 |
| ENSMUSG00000040883 | 23.019 | 31.746 | -0.462 | 0.001 | None | Tmem205 | transmembrane protein 205 |
| ENSMUSG00000040990 | 10.975 | 7.589 | 0.535 | 0.000 | K12470 | Sh3kbp1 | SH3-domain kinase binding protein 1 |
| ENSMUSG00000041143 | 6.648 | 9.817 | -0.559 | 0.000 | None | Tmco4 | transmembrane and coiled-coil domains 4 |
| ENSMUSG00000041193 | 6.471 | 9.659 | -0.576 | 0.001 | K01047 | Pla2g5 | phospholipase A2, group V |
| ENSMUSG00000041202 | 0.691 | 1.685 | -1.286 | 0.000 | K01047 | Pla2g2d | phospholipase A2, group IID |
| ENSMUSG00000041362 | 1.792 | 1.054 | 0.768 | 0.006 | None | Shtn1 | shootin 1 |
| ENSMUSG00000041378 | 31.976 | 71.209 | -1.156 | 0.000 | K06087 | Cldn5 | claudin 5 |
| ENSMUSG00000041449 | 0.610 | 0.192 | 1.675 | 0.047 |  | Serpina3h | serine (or cysteine) peptidase inhibitor, clade A, member 3H |
| ENSMUSG00000041481 | 4.895 | 2.602 | 0.916 | 0.009 | K04525 | Serpina3g | serine (or cysteine) peptidase inhibitor, clade A, member 3G |
| ENSMUSG00000041685 | 7.294 | 5.365 | 0.449 | 0.017 | K20042 | Fcho2 | FCH domain only 2 |
| ENSMUSG00000041747 | 3.059 | 2.332 | 0.394 | 0.030 | K14549 | Utp15 | UTP15 small subunit processome component |
| ENSMUSG00000041889 | 6.512 | 11.248 | -0.785 | 0.000 | None | Shisa4 | shisa family member 4 |
| ENSMUSG00000041935 | 7.397 | 5.389 | 0.460 | 0.001 | None | AW549877 | expressed sequence AW549877 |
| ENSMUSG00000042043 | 34.944 | 24.514 | 0.514 | 0.003 | K17292 | Tbca | tubulin cofactor A |
| ENSMUSG00000042109 | 24.170 | 33.573 | -0.473 | 0.000 | None | Csdc2 | cold shock domain containing C2, RNA binding |
| ENSMUSG00000042167 | 6.255 | 4.754 | 0.399 | 0.011 | K14079 | Papd4 | PAP associated domain containing 4 |
| ENSMUSG00000042302 | 12.978 | 8.967 | 0.536 | 0.000 | None | Ehbp1 | EH domain binding protein 1 |
| ENSMUSG00000042349 | 0.884 | 0.429 | 1.048 | 0.010 | K07211 | Ikbke | inhibitor of kappaB kinase epsilon |
| ENSMUSG00000042354 | 6.698 | 4.814 | 0.479 | 0.005 | K14538 | Gnl3 | guanine nucleotide binding protein-like 3 (nucleolar) |
| ENSMUSG00000042389 | 1.741 | 2.624 | -0.590 | 0.005 | K15322 | Tsen2 | tRNA splicing endonuclease subunit 2 |
| ENSMUSG00000042460 | 3.670 | 2.259 | 0.703 | 0.000 | K00731 | C1galt1 | core 1 synthase, glycoprotein-N-acetylgalactosamine 3-beta-galactosyltransferase, 1 |
| ENSMUSG00000042462 | 19.605 | 27.166 | -0.468 | 0.007 | K16904 | Dctpp1 | dCTP pyrophosphatase 1 |
| ENSMUSG00000042515 | 0.473 | 0.198 | 1.260 | 0.023 | None | Mum1l1 | melanoma associated antigen (mutated) 1-like 1 |
| ENSMUSG00000042642 | 14.180 | 18.619 | -0.390 | 0.000 | K00953 | Flad1 | flavin adenine dinucleotide synthetase 1 |
| ENSMUSG00000042675 | 52.442 | 75.816 | -0.529 | 0.000 | None | Ypel3 | yippee like 3 |
| ENSMUSG00000042684 | 1.807 | 0.877 | 1.049 | 0.007 | K01639 | Npl | N-acetylneuraminate pyruvate lyase |
| ENSMUSG00000042712 | 14.197 | 8.664 | 0.715 | 0.000 | None | Tceal9 | transcription elongation factor A like 9 |
| ENSMUSG00000042742 | 2.881 | 1.906 | 0.599 | 0.003 | None | Bmt2 | base methyltransferase of 25S rRNA 2 |
| ENSMUSG00000042807 | 3.427 | 2.506 | 0.454 | 0.005 | K12168 | Hecw2 | HECT, C2 and WW domain containing E3 ubiquitin protein ligase 2 |
| ENSMUSG00000042895 | 10.829 | 15.312 | -0.498 | 0.000 | None | Abra | actin-binding Rho activating protein |
| ENSMUSG00000042988 | 0.155 | 0.009 | 4.169 | 0.020 | K19882 | Notum | notum palmitoleoyl-protein carboxylesterase |
| ENSMUSG00000043004 | 4.135 | 2.441 | 0.763 | 0.000 | K07826 | Gng2 | guanine nucleotide binding protein (G protein), gamma 2 |
| ENSMUSG00000043099 | 5.901 | 7.867 | -0.411 | 0.049 | None | Hic1 | hypermethylated in cancer 1 |
| ENSMUSG00000043122 | 26.948 | 36.020 | -0.416 | 0.005 | None | A530016L24Rik | RIKEN cDNA A530016L24 gene |
| ENSMUSG00000043230 | 0.366 | 0.672 | -0.880 | 0.034 | None | Fam124b | family with sequence similarity 124, member B |
| ENSMUSG00000043251 | 1.548 | 2.218 | -0.517 | 0.029 | K19987 | Exoc3l | exocyst complex component 3-like |
| ENSMUSG00000043384 | 1.553 | 1.163 | 0.418 | 0.027 | None | Gprasp1 | G protein-coupled receptor associated sorting protein 1 |
| ENSMUSG00000043432 | 2.380 | 3.244 | -0.443 | 0.027 | None | Leng9 | leukocyte receptor cluster (LRC) member 9 |
| ENSMUSG00000043542 | 4.240 | 3.192 | 0.412 | 0.021 | None | Zc2hc1a | zinc finger, C2HC-type containing 1A |
| ENSMUSG00000043629 | 0.422 | 0.198 | 1.093 | 0.020 | None | 1700019D03Rik | RIKEN cDNA 1700019D03 gene |
| ENSMUSG00000043832 | 3.443 | 1.652 | 1.061 | 0.004 | K10057 | Clec4a3 | C-type lectin domain family 4, member a3 |
| ENSMUSG00000043943 | 3.985 | 2.896 | 0.463 | 0.005 | K01301 | Naalad2 | N-acetylated alpha-linked acidic dipeptidase 2 |
| ENSMUSG00000044030 | 10.359 | 13.552 | -0.385 | 0.005 | None | Irf2bp1 | interferon regulatory factor 2 binding protein 1 |
| ENSMUSG00000044433 | 0.239 | 0.510 | -1.089 | 0.012 | K17493 | Camsap3 | calmodulin regulated spectrin-associated protein family, member 3 |
| ENSMUSG00000044499 | 1.892 | 0.901 | 1.073 | 0.000 | K08104 | Hs3st5 | heparan sulfate (glucosamine) 3-O-sulfotransferase 5 |
| ENSMUSG00000044548 | 0.979 | 1.505 | -0.617 | 0.047 | None | Dact1 | dishevelled-binding antagonist of beta-catenin 1 |
| ENSMUSG00000044583 | 2.287 | 1.019 | 1.168 | 0.000 | K05404 | Tlr7 | toll-like receptor 7 |
| ENSMUSG00000044595 | 1.241 | 2.154 | -0.793 | 0.004 | None | Dnd1 | DND microRNA-mediated repression inhibitor 1 |
| ENSMUSG00000044768 | 0.135 | 0.086 | 0.644 | 0.012 | None | D1Ertd622e | DNA segment, Chr 1, ERATO Doi 622, expressed |
| ENSMUSG00000044807 | 0.727 | 0.469 | 0.638 | 0.026 | K09228 | Zfp354c | zinc finger protein 354C |
| ENSMUSG00000044811 | 3.125 | 1.847 | 0.759 | 0.024 | K06719 | Cd300c2 | CD300C molecule 2 |
| ENSMUSG00000044827 | 0.720 | 0.359 | 1.006 | 0.030 | K05398 | Tlr1 | toll-like receptor 1 |
| ENSMUSG00000044876 | 1.853 | 2.420 | -0.383 | 0.020 | K09230 | Zfp444 | zinc finger protein 444 |
| ENSMUSG00000044894 | 304.289 | 416.558 | -0.451 | 0.000 | K00418 | Uqcrq | ubiquinol-cytochrome c reductase, complex III subunit VII |
| ENSMUSG00000044934 | 5.093 | 3.552 | 0.522 | 0.006 | None | Zfp367 | zinc finger protein 367 |
| ENSMUSG00000044951 | 26.511 | 16.977 | 0.646 | 0.000 | K00907 | Mylk4 | myosin light chain kinase family, member 4 |
| ENSMUSG00000044986 | 5.343 | 7.258 | -0.439 | 0.001 | K01011 | Tst | thiosulfate sulfurtransferase, mitochondrial |
| ENSMUSG00000045102 | 0.451 | 0.884 | -0.970 | 0.017 | K16618 | Poln | DNA polymerase N |
| ENSMUSG00000045237 | 10.329 | 15.081 | -0.543 | 0.007 | None | 1110012L19Rik | RIKEN cDNA 1110012L19 gene |
| ENSMUSG00000045348 | 0.297 | 0.551 | -0.886 | 0.036 | None | Nyap1 | neuronal tyrosine-phosphorylated phosphoinositide 3-kinase adaptor 1 |
| ENSMUSG00000045414 | 7.526 | 5.565 | 0.438 | 0.005 | None | 1190002N15Rik | RIKEN cDNA 1190002N15 gene |
| ENSMUSG00000045573 | 6.320 | 9.447 | -0.577 | 0.001 | K18832 | Penk | preproenkephalin |
| ENSMUSG00000045613 | 19.839 | 13.498 | 0.559 | 0.001 | K04130 | Chrm2 | cholinergic receptor, muscarinic 2, cardiac |
| ENSMUSG00000045822 | 1.380 | 2.025 | -0.552 | 0.039 | K17604 | Zswim3 | zinc finger SWIM-type containing 3 |
| ENSMUSG00000045868 | 0.286 | 0.125 | 1.191 | 0.006 | None | Gvin1 | GTPase, very large interferon inducible 1 |
| ENSMUSG00000045932 | 6.148 | 4.023 | 0.614 | 0.000 | None | Ifit2 | interferon-induced protein with tetratricopeptide repeats 2 |
| ENSMUSG00000046240 | 0.165 | 0.030 | 2.508 | 0.029 | None | Hepacam | hepatocyte cell adhesion molecule |
| ENSMUSG00000046245 | 0.992 | 0.283 | 1.803 | 0.000 | K15411 | Pilra | paired immunoglobin-like type 2 receptor alpha |
| ENSMUSG00000046312 | 4.176 | 5.464 | -0.386 | 0.012 | None | AI464131 | expressed sequence AI464131 |
| ENSMUSG00000046402 | 10.771 | 14.151 | -0.390 | 0.012 | None | Rbp1 | retinol binding protein 1, cellular |
| ENSMUSG00000046415 | 2.336 | 3.384 | -0.533 | 0.009 |  | B430212C06Rik | RIKEN cDNA B430212C06 gene |
| ENSMUSG00000046532 | 0.781 | 0.546 | 0.516 | 0.016 | K08557 | Ar | androgen receptor |
| ENSMUSG00000046598 | 2.435 | 6.756 | -1.472 | 0.034 | K00019 | Bdh1 | 3-hydroxybutyrate dehydrogenase, type 1 |
| ENSMUSG00000046805 | 10.183 | 6.322 | 0.695 | 0.034 | None | Mpeg1 | macrophage expressed gene 1 |
| ENSMUSG00000046814 | 3.751 | 8.370 | -1.156 | 0.000 | None | Gchfr | GTP cyclohydrolase I feedback regulator |
| ENSMUSG00000046879 | 3.904 | 2.556 | 0.613 | 0.008 | K14139 | Irgm1 | immunity-related GTPase family M member 1 |
| ENSMUSG00000046947 | 3.474 | 4.620 | -0.409 | 0.006 | K08869 | Adck2 | aarF domain containing kinase 2 |
| ENSMUSG00000047181 | 2.628 | 3.771 | -0.518 | 0.014 | None | Samd14 | sterile alpha motif domain containing 14 |
| ENSMUSG00000047205 | 6.528 | 8.754 | -0.420 | 0.020 | K14165 | Dusp18 | dual specificity phosphatase 18 |
| ENSMUSG00000047379 | 23.703 | 30.878 | -0.380 | 0.003 | K00741 | B4gat1 | beta-1,4-glucuronyltransferase 1 |
| ENSMUSG00000047554 | 5.784 | 4.407 | 0.395 | 0.015 | None | Tmem41b | transmembrane protein 41B |
| ENSMUSG00000047694 | 3.649 | 2.761 | 0.404 | 0.042 | None | Yipf6 | Yip1 domain family, member 6 |
| ENSMUSG00000047996 | 2.299 | 1.401 | 0.718 | 0.010 | None | Prrg1 | proline rich Gla (G-carboxyglutamic acid) 1 |
| ENSMUSG00000048106 | 3.029 | 2.316 | 0.390 | 0.049 |  | 4632415L05Rik | RRS1 ribosome biogenesis regulator homolog pseudogene |
| ENSMUSG00000048118 | 3.403 | 2.525 | 0.434 | 0.035 | K19194 | Arid4a | AT rich interactive domain 4A (RBP1-like) |
| ENSMUSG00000048249 | 7.434 | 5.148 | 0.534 | 0.001 | None | Crebrf | CREB3 regulatory factor |
| ENSMUSG00000048371 | 4.021 | 5.249 | -0.382 | 0.018 | K01102 | Pdp2 | pyruvate dehyrogenase phosphatase catalytic subunit 2 |
| ENSMUSG00000048440 | 3.504 | 4.872 | -0.473 | 0.020 | K00490 | Cyp4f16 | cytochrome P450, family 4, subfamily f, polypeptide 16 |
| ENSMUSG00000048498 | 0.456 | 0.037 | 3.560 | 0.001 | K06719 | Cd300e | CD300E molecule |
| ENSMUSG00000048852 | 0.479 | 0.249 | 0.941 | 0.047 | None | Gm12185 | predicted gene 12185 |
| ENSMUSG00000048865 | 2.266 | 1.591 | 0.515 | 0.042 | None | Arhgap30 | Rho GTPase activating protein 30 |
| ENSMUSG00000049037 | 5.381 | 2.629 | 1.037 | 0.003 | K10057 | Clec4a1 | C-type lectin domain family 4, member a1 |
| ENSMUSG00000049076 | 6.413 | 4.807 | 0.420 | 0.005 | K12489 | Acap2 | ArfGAP with coiled-coil, ankyrin repeat and PH domains 2 |
| ENSMUSG00000049103 | 1.007 | 0.428 | 1.238 | 0.001 | K04177 | Ccr2 | chemokine (C-C motif) receptor 2 |
| ENSMUSG00000049130 | 5.008 | 3.111 | 0.691 | 0.001 | K04010 | C5ar1 | complement component 5a receptor 1 |
| ENSMUSG00000049164 | 0.863 | 0.540 | 0.680 | 0.044 | None | Zfp518a | zinc finger protein 518A |
| ENSMUSG00000049287 | 8.529 | 13.052 | -0.613 | 0.000 | None | Iba57 | IBA57 homolog, iron-sulfur cluster assembly |
| ENSMUSG00000049537 | 19.773 | 14.841 | 0.417 | 0.007 | None | Tecrl | trans-2,3-enoyl-CoA reductase-like |
| ENSMUSG00000049556 | 0.599 | 0.246 | 1.281 | 0.014 | None | Lingo1 | leucine rich repeat and Ig domain containing 1 |
| ENSMUSG00000049858 | 7.737 | 10.227 | -0.400 | 0.002 | K00387 | Suox | sulfite oxidase |
| ENSMUSG00000049878 | 3.180 | 2.298 | 0.470 | 0.022 | None | Rlf | rearranged L-myc fusion sequence |
| ENSMUSG00000049892 | 6.339 | 10.782 | -0.767 | 0.002 | K07843 | Rasd1 | RAS, dexamethasone-induced 1 |
| ENSMUSG00000049932 | 4.559 | 7.303 | -0.677 | 0.000 | K11251 | H2afx | H2A histone family, member X |
| ENSMUSG00000049969 | 4.743 | 3.617 | 0.393 | 0.011 | None | Plekhf2 | pleckstrin homology domain containing, family F (with FYVE domain) member 2 |
| ENSMUSG00000049985 | 0.152 | 0.438 | -1.528 | 0.011 | None | Ankrd55 | ankyrin repeat domain 55 |
| ENSMUSG00000049988 | 2.832 | 1.776 | 0.678 | 0.036 | None | Lrrc25 | leucine rich repeat containing 25 |
| ENSMUSG00000050052 | 1.785 | 2.559 | -0.517 | 0.048 | None | Tdrp | testis development related protein |
| ENSMUSG00000050195 | 30.716 | 22.403 | 0.456 | 0.001 | K00507 | Scd4 | stearoyl-coenzyme A desaturase 4 |
| ENSMUSG00000050199 | 6.178 | 4.695 | 0.399 | 0.003 | K04309 | Lgr4 | leucine-rich repeat-containing G protein-coupled receptor 4 |
| ENSMUSG00000050212 | 6.928 | 10.527 | -0.601 | 0.003 | None | Eva1b | eva-1 homolog B (C. elegans) |
| ENSMUSG00000050222 | 2.411 | 3.864 | -0.677 | 0.038 | K05492 | Il17d | interleukin 17D |
| ENSMUSG00000050296 | 1.054 | 0.761 | 0.472 | 0.017 | K05646 | Abca12 | ATP-binding cassette, sub-family A (ABC1), member 12 |
| ENSMUSG00000050310 | 2.632 | 1.950 | 0.436 | 0.011 | K08267 | Rictor | RPTOR independent companion of MTOR, complex 2 |
| ENSMUSG00000050335 | 12.693 | 8.272 | 0.622 | 0.011 | K06831 | Lgals3 | lectin, galactose binding, soluble 3 |
| ENSMUSG00000050447 | 0.043 | 0.186 | -2.103 | 0.038 | None | Lypd6 | LY6/PLAUR domain containing 6 |
| ENSMUSG00000050493 | 1.155 | 0.435 | 1.412 | 0.032 | None | Fam167b | family with sequence similarity 167, member B |
| ENSMUSG00000050623 | 1.125 | 2.463 | -1.127 | 0.012 | None | Catsperz | cation channel sperm associated auxiliary subunit zeta |
| ENSMUSG00000050730 | 2.049 | 1.274 | 0.689 | 0.001 | None | Arhgap42 | Rho GTPase activating protein 42 |
| ENSMUSG00000050737 | 0.802 | 1.196 | -0.574 | 0.031 | K15729 | Ptges | prostaglandin E synthase |
| ENSMUSG00000050747 | 0.017 | 0.164 | -3.290 | 0.032 | K12005 | Trim15 | tripartite motif-containing 15 |
| ENSMUSG00000050821 | 7.974 | 11.335 | -0.506 | 0.000 | None | Fam131a | family with sequence similarity 131, member A |
| ENSMUSG00000050890 | 0.926 | 0.610 | 0.603 | 0.042 | K17539 | Pdik1l | PDLIM1 interacting kinase 1 like |
| ENSMUSG00000051285 | 8.131 | 5.522 | 0.564 | 0.008 | None | Pcmtd1 | protein-L-isoaspartate (D-aspartate) O-methyltransferase domain containing 1 |
| ENSMUSG00000051314 | 0.161 | 0.021 | 2.935 | 0.041 | K04328 | Ffar2 | free fatty acid receptor 2 |
| ENSMUSG00000051412 | 3.476 | 2.351 | 0.568 | 0.013 | K08515 | Vamp7 | vesicle-associated membrane protein 7 |
| ENSMUSG00000051457 | 2.199 | 0.730 | 1.594 | 0.000 | K06477 | Spn | sialophorin |
| ENSMUSG00000051579 | 7.813 | 4.805 | 0.704 | 0.000 | None | Tceal8 | transcription elongation factor A (SII)-like 8 |
| ENSMUSG00000051730 | 4.528 | 2.966 | 0.613 | 0.011 | None | Mettl5 | methyltransferase like 5 |
| ENSMUSG00000051811 | 5.074 | 7.920 | -0.640 | 0.036 | K02267 | Cox6b2 | cytochrome c oxidase subunit VIb polypeptide 2 |
| ENSMUSG00000051910 | 2.709 | 2.034 | 0.416 | 0.008 | K09269 | Sox6 | SRY (sex determining region Y)-box 6 |
| ENSMUSG00000052144 | 5.809 | 4.310 | 0.433 | 0.001 | K15425 | Ppp4r2 | protein phosphatase 4, regulatory subunit 2 |
| ENSMUSG00000052160 | 0.413 | 0.125 | 1.726 | 0.032 | K16860 | Pld4 | phospholipase D family, member 4 |
| ENSMUSG00000052212 | 0.316 | 0.058 | 2.465 | 0.019 | K06552 | Cd177 | CD177 antigen |
| ENSMUSG00000052336 | 1.476 | 0.839 | 0.817 | 0.009 | K04192 | Cx3cr1 | chemokine (C-X3-C motif) receptor 1 |
| ENSMUSG00000052749 | 0.222 | 0.051 | 2.110 | 0.003 | None | Trim30b | tripartite motif-containing 30B |
| ENSMUSG00000052812 | 1.521 | 1.103 | 0.467 | 0.048 | None | Atad2b | ATPase family, AAA domain containing 2B |
| ENSMUSG00000052825 | 5.246 | 2.942 | 0.836 | 0.004 |  | Gm9892 | eukaryotic translation initiation factor 2, subunit 2 (beta) pseudogene |
| ENSMUSG00000052889 | 2.602 | 1.629 | 0.678 | 0.002 | K19662 | Prkcb | protein kinase C, beta |
| ENSMUSG00000052917 | 4.827 | 3.559 | 0.442 | 0.001 | K08596 | Senp7 | SUMO1/sentrin specific peptidase 7 |
| ENSMUSG00000052920 | 3.491 | 2.211 | 0.662 | 0.003 | K07376 | Prkg1 | protein kinase, cGMP-dependent, type I |
| ENSMUSG00000053004 | 0.077 | 0.216 | -1.491 | 0.040 | K04149 | Hrh1 | histamine receptor H1 |
| ENSMUSG00000053007 | 1.042 | 0.745 | 0.487 | 0.047 | K09047 | Creb5 | cAMP responsive element binding protein 5 |
| ENSMUSG00000053063 | 1.985 | 1.049 | 0.925 | 0.001 | K17516 | Clec12a | C-type lectin domain family 12, member a |
| ENSMUSG00000053093 | 535.192 | 228.141 | 1.227 | 0.025 | K17751 | Myh7 | myosin, heavy polypeptide 7, cardiac muscle, beta |
| ENSMUSG00000053101 | 0.236 | 0.052 | 2.163 | 0.039 | K08429 | Gpr141 | G protein-coupled receptor 141 |
| ENSMUSG00000053166 | 0.283 | 0.727 | -1.363 | 0.019 | K06812 | Cdh22 | cadherin 22 |
| ENSMUSG00000053318 | 0.684 | 0.136 | 2.303 | 0.001 | K16853 | Slamf8 | SLAM family member 8 |
| ENSMUSG00000053329 | 232.418 | 306.155 | -0.396 | 0.000 | None | D10Jhu81e | DNA segment, Chr 10, Johns Hopkins University 81 expressed |
| ENSMUSG00000053347 | 3.115 | 1.888 | 0.727 | 0.032 | None | Zfp943 | zinc finger prtoein 943 |
| ENSMUSG00000053453 | 3.217 | 2.269 | 0.507 | 0.006 | K13176 | Thoc7 | THO complex 7 |
| ENSMUSG00000053702 | 21.116 | 13.980 | 0.600 | 0.000 | None | Nebl | nebulette |
| ENSMUSG00000054072 | 15.811 | 7.622 | 1.058 | 0.000 | None | Iigp1 | interferon inducible GTPase 1 |
| ENSMUSG00000054203 | 7.653 | 3.948 | 0.958 | 0.000 | None | Ifi205 | interferon activated gene 205 |
| ENSMUSG00000054226 | 6.303 | 4.487 | 0.493 | 0.004 | K15901 | Tprkb | Tp53rk binding protein |
| ENSMUSG00000054404 | 6.007 | 4.444 | 0.439 | 0.014 | None | Slfn5 | schlafen 5 |
| ENSMUSG00000054408 | 12.989 | 9.783 | 0.411 | 0.000 |  | Spcs3 | signal peptidase complex subunit 3 homolog (S. cerevisiae) |
| ENSMUSG00000054519 | 3.519 | 2.384 | 0.565 | 0.005 | K09228 | Zfp867 | zinc finger protein 867 |
| ENSMUSG00000054594 | 0.742 | 1.512 | -1.024 | 0.027 | K14377 | Oscar | osteoclast associated receptor |
| ENSMUSG00000054612 | 3.014 | 4.160 | -0.462 | 0.005 | K00567 | Mgmt | O-6-methylguanine-DNA methyltransferase |
| ENSMUSG00000054640 | 20.305 | 12.973 | 0.648 | 0.000 | K05849 | Slc8a1 | solute carrier family 8 (sodium/calcium exchanger), member 1 |
| ENSMUSG00000054716 | 16.781 | 23.905 | -0.508 | 0.000 | None | Zfp771 | zinc finger protein 771 |
| ENSMUSG00000054752 | 3.877 | 2.749 | 0.500 | 0.017 | None | Fsd1l | fibronectin type III and SPRY domain containing 1-like |
| ENSMUSG00000054863 | 2.245 | 3.716 | -0.725 | 0.000 | None | Fam19a5 | family with sequence similarity 19, member A5 |
| ENSMUSG00000055116 | 3.828 | 5.505 | -0.524 | 0.014 | K02296 | Arntl | aryl hydrocarbon receptor nuclear translocator-like |
| ENSMUSG00000055148 | 64.644 | 91.955 | -0.509 | 0.001 | K17845 | Klf2 | Kruppel-like factor 2 (lung) |
| ENSMUSG00000055150 | 0.382 | 0.164 | 1.221 | 0.018 | K09228 | Zfp78 | zinc finger protein 78 |
| ENSMUSG00000055413 | 11.405 | 7.498 | 0.609 | 0.010 |  | H2-Q5 | histocompatibility 2, Q region locus 5 |
| ENSMUSG00000055421 | 0.522 | 0.269 | 0.957 | 0.025 | K16498 | Pcdh9 | protocadherin 9 |
| ENSMUSG00000055435 | 2.135 | 1.370 | 0.644 | 0.002 | K09035 | Maf | avian musculoaponeurotic fibrosarcoma oncogene homolog |
| ENSMUSG00000055541 | 0.731 | 0.333 | 1.135 | 0.001 | K06725 | Lair1 | leukocyte-associated Ig-like receptor 1 |
| ENSMUSG00000055629 | 0.164 | 0.030 | 2.464 | 0.023 | K09657 | B4galnt4 | beta-1,4-N-acetyl-galactosaminyl transferase 4 |
| ENSMUSG00000055652 | 2.069 | 2.742 | -0.403 | 0.034 | K10462 | Klhl25 | kelch-like 25 |
| ENSMUSG00000055660 | 1.373 | 0.942 | 0.546 | 0.009 | None | Mettl4 | methyltransferase like 4 |
| ENSMUSG00000055745 | 2.935 | 4.081 | -0.474 | 0.011 | None | Rtl6 | retrotransposon Gag like 6 |
| ENSMUSG00000055912 | 1.039 | 0.194 | 2.419 | 0.004 | None | Tmem150a | transmembrane protein 150A |
| ENSMUSG00000055978 | 0.220 | 0.664 | -1.589 | 0.003 | K00718 | Fut2 | fucosyltransferase 2 |
| ENSMUSG00000055994 | 0.788 | 1.152 | -0.545 | 0.049 | K10165 | Nod2 | nucleotide-binding oligomerization domain containing 2 |
| ENSMUSG00000056019 | 0.645 | 0.411 | 0.650 | 0.036 | K09228 | Zfp709 | zinc finger protein 709 |
| ENSMUSG00000056054 | 15.287 | 4.850 | 1.660 | 0.000 | None | S100a8 | S100 calcium binding protein A8 (calgranulin A) |
| ENSMUSG00000056071 | 34.086 | 13.367 | 1.354 | 0.046 | None | S100a9 | S100 calcium binding protein A9 (calgranulin B) |
| ENSMUSG00000056290 | 0.367 | 0.142 | 1.359 | 0.007 | None | Ms4a4b | membrane-spanning 4-domains, subfamily A, member 4B |
| ENSMUSG00000056501 | 0.811 | 1.655 | -1.027 | 0.038 | K10048 | Cebpb | CCAAT/enhancer binding protein (C/EBP), beta |
| ENSMUSG00000056698 | 13.415 | 18.101 | -0.429 | 0.000 | None | Elmod3 | ELMO/CED-12 domain containing 3 |
| ENSMUSG00000056749 | 5.476 | 7.828 | -0.513 | 0.001 | K09059 | Nfil3 | nuclear factor, interleukin 3, regulated |
| ENSMUSG00000056973 | 44.696 | 67.914 | -0.602 | 0.000 | K01044 | Ces1d | carboxylesterase 1D |
| ENSMUSG00000057133 | 8.191 | 5.636 | 0.541 | 0.001 | K14436 | Chd6 | chromodomain helicase DNA binding protein 6 |
| ENSMUSG00000057193 | 26.354 | 34.842 | -0.400 | 0.000 | K15377 | Slc44a2 | solute carrier family 44, member 2 |
| ENSMUSG00000057342 | 6.240 | 8.776 | -0.490 | 0.000 | K04718 | Sphk2 | sphingosine kinase 2 |
| ENSMUSG00000057465 | 0.269 | 0.004 | 6.005 | 0.005 | K17310 | Saa2 | serum amyloid A 2 |
| ENSMUSG00000057561 | 6.099 | 4.410 | 0.471 | 0.003 | K03236 | Eif1a | eukaryotic translation initiation factor 1A |
| ENSMUSG00000057596 | 2.053 | 0.881 | 1.221 | 0.000 | None | Trim30d | tripartite motif-containing 30D |
| ENSMUSG00000057604 | 31.452 | 41.443 | -0.396 | 0.000 | None | Lmcd1 | LIM and cysteine-rich domains 1 |
| ENSMUSG00000057722 | 1.234 | 0.688 | 0.849 | 0.043 | K05062 | Lepr | leptin receptor |
| ENSMUSG00000057880 | 2.034 | 3.138 | -0.624 | 0.000 | K13524 | Abat | 4-aminobutyrate aminotransferase |
| ENSMUSG00000057913 | 0.300 | 0.920 | -1.623 | 0.021 |  | Gm10032 | predicted gene 10032 |
| ENSMUSG00000057967 | 4.809 | 3.304 | 0.545 | 0.030 | K04358 | Fgf18 | fibroblast growth factor 18 |
| ENSMUSG00000058099 | 1.521 | 1.121 | 0.444 | 0.035 | None | Nfam1 | Nfat activating molecule with ITAM motif 1 |
| ENSMUSG00000058183 | 0.003 | 0.099 | -4.707 | 0.013 | K08635 | Mmel1 | membrane metallo-endopeptidase-like 1 |
| ENSMUSG00000058192 | 0.405 | 0.279 | 0.542 | 0.025 | None | Zfp846 | zinc finger protein 846 |
| ENSMUSG00000058291 | 5.071 | 3.610 | 0.492 | 0.004 | K09228 | Zfp68 | zinc finger protein 68 |
| ENSMUSG00000058298 | 1.904 | 1.442 | 0.403 | 0.046 | K10738 | Mcm9 | minichromosome maintenance 9 homologous recombination repair factor |
| ENSMUSG00000058624 | 5.377 | 3.270 | 0.720 | 0.000 | K01487 | Gda | guanine deaminase |
| ENSMUSG00000058672 | 13.714 | 17.877 | -0.379 | 0.004 | K07375 | Tubb2a | tubulin, beta 2A class IIA |
| ENSMUSG00000058818 | 3.712 | 2.010 | 0.890 | 0.003 | K06512 | Pirb | paired Ig-like receptor B |
| ENSMUSG00000058833 | 50.885 | 68.709 | -0.430 | 0.000 | None | Rex1bd | required for excision 1-B domain containing |
| ENSMUSG00000058835 | 47.630 | 31.055 | 0.625 | 0.045 | None | Abi1 | abl-interactor 1 |
| ENSMUSG00000058975 | 0.015 | 0.146 | -3.311 | 0.000 | K04887 | Kcnc1 | potassium voltage gated channel, Shaw-related subfamily, member 1 |
| ENSMUSG00000059089 | 4.113 | 0.850 | 2.270 | 0.000 | K06463 | Fcgr4 | Fc receptor, IgG, low affinity IV |
| ENSMUSG00000059142 | 2.822 | 1.936 | 0.547 | 0.014 | None | Zfp945 | zinc finger protein 945 |
| ENSMUSG00000059278 | 14.590 | 22.153 | -0.599 | 0.000 | None | Naa38 | N(alpha)-acetyltransferase 38, NatC auxiliary subunit |
| ENSMUSG00000059422 | 7.168 | 4.380 | 0.710 | 0.048 |  | Gm8116 | predicted gene 8116 |
| ENSMUSG00000059481 | 1.405 | 0.003 | 8.561 | 0.035 | K01315 | Plg | plasminogen |
| ENSMUSG00000059498 | 25.534 | 15.873 | 0.691 | 0.003 | K16824 | Fcgr3 | Fc receptor, IgG, low affinity III |
| ENSMUSG00000059588 | 4.871 | 2.946 | 0.729 | 0.000 | K04577 | Calcrl | calcitonin receptor-like |
| ENSMUSG00000059820 | 3.470 | 2.495 | 0.479 | 0.012 | None | AU019823 | expressed sequence AU019823 |
| ENSMUSG00000059878 | 10.391 | 7.982 | 0.383 | 0.001 | None | Zfp422 | zinc finger protein 422 |
| ENSMUSG00000059897 | 1.624 | 0.992 | 0.714 | 0.027 | K09228 | Zfp930 | zinc finger protein 930 |
| ENSMUSG00000059900 | 0.518 | 0.120 | 2.088 | 0.033 | None | Tmem40 | transmembrane protein 40 |
| ENSMUSG00000059908 | 1.811 | 0.000 | 10.646 | 0.003 | None | Mug1 | murinoglobulin 1 |
| ENSMUSG00000059970 | 3.130 | 4.626 | -0.560 | 0.001 | K03283 | Hspa2 | heat shock protein 2 |
| ENSMUSG00000060063 | 4.797 | 3.158 | 0.607 | 0.048 | None | Alox5ap | arachidonate 5-lipoxygenase activating protein |
| ENSMUSG00000060131 | 0.371 | 0.169 | 1.142 | 0.012 | K01530 | Atp8b4 | ATPase, class I, type 8B, member 4 |
| ENSMUSG00000060376 | 51.234 | 72.547 | -0.500 | 0.000 | K00166 | Bckdha | branched chain ketoacid dehydrogenase E1, alpha polypeptide |
| ENSMUSG00000060509 | 0.016 | 0.075 | -2.211 | 0.048 | K04193 | Xcr1 | chemokine (C motif) receptor 1 |
| ENSMUSG00000060512 | 2.554 | 3.804 | -0.573 | 0.019 | None | 0610040J01Rik | RIKEN cDNA 0610040J01 gene |
| ENSMUSG00000060548 | 2.386 | 1.784 | 0.422 | 0.033 | K05155 | Tnfrsf19 | tumor necrosis factor receptor superfamily, member 19 |
| ENSMUSG00000060568 | 0.760 | 1.110 | -0.545 | 0.032 | None | Fam78b | family with sequence similarity 78, member B |
| ENSMUSG00000060703 | 13.193 | 10.082 | 0.392 | 0.035 | K06722 | Cd302 | CD302 antigen |
| ENSMUSG00000060771 | 1.080 | 0.663 | 0.706 | 0.014 | None | Tsga10 | testis specific 10 |
| ENSMUSG00000060913 | 33.093 | 24.888 | 0.414 | 0.000 | K10654 | Trim55 | tripartite motif-containing 55 |
| ENSMUSG00000060981 | 23.596 | 31.008 | -0.391 | 0.017 | K11254 | Hist1h4h | histone cluster 1, H4h |
| ENSMUSG00000061136 | 2.940 | 1.960 | 0.590 | 0.015 | K12821 | Prpf40a | pre-mRNA processing factor 40A |
| ENSMUSG00000061175 | 3.461 | 2.256 | 0.618 | 0.006 | K20401 | Fnip2 | folliculin interacting protein 2 |
| ENSMUSG00000061273 | 10.522 | 7.814 | 0.431 | 0.001 | None | Mmgt1 | membrane magnesium transporter 1 |
| ENSMUSG00000061482 | 0.654 | 0.142 | 2.233 | 0.018 | K11254 | Hist1h4d | histone cluster 1, H4d |
| ENSMUSG00000061518 | 614.273 | 811.149 | -0.399 | 0.000 | K02265 | Cox5b | cytochrome c oxidase subunit Vb |
| ENSMUSG00000061535 | 2.678 | 1.898 | 0.500 | 0.008 | None | C1qtnf7 | C1q and tumor necrosis factor related protein 7 |
| ENSMUSG00000061603 | 11.541 | 8.624 | 0.423 | 0.000 | K16523 | Akap6 | A kinase (PRKA) anchor protein 6 |
| ENSMUSG00000061665 | 4.032 | 2.848 | 0.508 | 0.030 | K13738 | Cd2ap | CD2-associated protein |
| ENSMUSG00000062014 | 9.785 | 7.242 | 0.437 | 0.000 | None | Gmfb | glia maturation factor, beta |
| ENSMUSG00000062078 | 31.418 | 22.452 | 0.489 | 0.000 | K14945 | Qk | quaking |
| ENSMUSG00000062209 | 1.050 | 0.622 | 0.758 | 0.002 | K05085 | Erbb4 | erb-b2 receptor tyrosine kinase 4 |
| ENSMUSG00000062270 | 29.220 | 22.484 | 0.382 | 0.000 | K11339 | Morf4l1 | mortality factor 4 like 1 |
| ENSMUSG00000062300 | 12.355 | 17.739 | -0.520 | 0.000 | K06531 | Nectin2 | nectin cell adhesion molecule 2 |
| ENSMUSG00000062604 | 5.656 | 4.163 | 0.445 | 0.004 | K08831 | Srpk2 | serine/arginine-rich protein specific kinase 2 |
| ENSMUSG00000062785 | 0.112 | 0.300 | -1.423 | 0.003 | K04889 | Kcnc3 | potassium voltage gated channel, Shaw-related subfamily, member 3 |
| ENSMUSG00000062929 | 87.047 | 65.445 | 0.415 | 0.000 | K05765 | Cfl2 | cofilin 2, muscle |
| ENSMUSG00000063052 | 2.797 | 2.123 | 0.400 | 0.028 | None | Lrrc40 | leucine rich repeat containing 40 |
| ENSMUSG00000063146 | 4.120 | 5.728 | -0.473 | 0.000 | K10422 | Clip2 | CAP-GLY domain containing linker protein 2 |
| ENSMUSG00000063273 | 4.370 | 3.255 | 0.430 | 0.020 | K00670 | Naa15 | N(alpha)-acetyltransferase 15, NatA auxiliary subunit |
| ENSMUSG00000063286 | 1.190 | 0.717 | 0.737 | 0.046 |  | Gm8995 | predicted gene 8995 |
| ENSMUSG00000063334 | 2.591 | 1.563 | 0.732 | 0.000 | K06961 | Krr1 | KRR1, small subunit (SSU) processome component, homolog (yeast) |
| ENSMUSG00000063382 | 7.492 | 10.844 | -0.531 | 0.000 | None | Bcl9l | B cell CLL/lymphoma 9-like |
| ENSMUSG00000063388 | 2.364 | 1.091 | 1.117 | 0.027 | None | BC023105 | cDNA sequence BC023105 |
| ENSMUSG00000063594 | 1.649 | 0.872 | 0.919 | 0.040 | K04544 | Gng8 | guanine nucleotide binding protein (G protein), gamma 8 |
| ENSMUSG00000063889 | 1.588 | 2.482 | -0.641 | 0.004 | K09052 | Crem | cAMP responsive element modulator |
| ENSMUSG00000063897 | 8.015 | 10.749 | -0.421 | 0.003 | K11170 |  |  |
| ENSMUSG00000064254 | 15.549 | 23.121 | -0.570 | 0.000 | K17725 | Ethe1 | ethylmalonic encephalopathy 1 |
| ENSMUSG00000064289 | 4.170 | 3.165 | 0.400 | 0.034 | K12650 | Tank | TRAF family member-associated Nf-kappa B activator |
| ENSMUSG00000064337 | 1096.092 | 740.523 | 0.573 | 0.017 |  | mt-Rnr1 | s-rRNA |
| ENSMUSG00000064339 | 1167.065 | 703.591 | 0.735 | 0.014 |  | mt-Rnr2 | 16S ribosomal RNA |
| ENSMUSG00000064341 | 22237.205 | 14051.937 | 0.667 | 0.000 | K03878 |  |  |
| ENSMUSG00000064345 | 7661.998 | 4429.183 | 0.799 | 0.007 | K03879 |  |  |
| ENSMUSG00000064356 | 3.097 | 0.616 | 2.303 | 0.020 | K02125 |  |  |
| ENSMUSG00000064360 | 86.929 | 44.751 | 0.964 | 0.010 | K03880 |  |  |
| ENSMUSG00000064363 | 6145.061 | 4535.450 | 0.442 | 0.003 | K03881 |  |  |
| ENSMUSG00000064367 | 7552.471 | 5353.880 | 0.502 | 0.001 | K03883 |  |  |
| ENSMUSG00000064368 | 2436.974 | 857.340 | 1.517 | 0.000 | K03884 |  |  |
| ENSMUSG00000065037 | 15.664 | 22.465 | -0.517 | 0.022 |  | Rn7sk | RNA, 7SK, nuclear |
| ENSMUSG00000066154 | 3.965 | 0.011 | 8.572 | 0.018 | None | Mup3 | major urinary protein 3 |
| ENSMUSG00000066235 | 5.918 | 8.178 | -0.466 | 0.047 | K18207 | Pomgnt2 | protein O-linked mannose beta 1,4-N-acetylglucosaminyltransferase 2 |
| ENSMUSG00000066363 | 1.340 | 0.267 | 2.331 | 0.002 | K04525 | Serpina3f | serine (or cysteine) peptidase inhibitor, clade A, member 3F |
| ENSMUSG00000066366 | 1.936 | 0.006 | 8.222 | 0.008 | K03984 | Serpina1a | serine (or cysteine) peptidase inhibitor, clade A, member 1A |
| ENSMUSG00000066551 | 18.469 | 11.967 | 0.630 | 0.000 | K10802 | Hmgb1 | high mobility group box 1 |
| ENSMUSG00000066607 | 7.981 | 10.910 | -0.448 | 0.003 | None | 6030419C18Rik | RIKEN cDNA 6030419C18 gene |
| ENSMUSG00000066621 | 15.130 | 21.867 | -0.530 | 0.000 | K17988 | Tecpr1 | tectonin beta-propeller repeat containing 1 |
| ENSMUSG00000066798 | 1.228 | 0.773 | 0.669 | 0.030 | K10493 | Zbtb6 | zinc finger and BTB domain containing 6 |
| ENSMUSG00000066880 | 4.672 | 3.479 | 0.427 | 0.016 | K09228 | Zfp617 | zinc finger protein 617 |
| ENSMUSG00000066894 | 3.898 | 5.154 | -0.401 | 0.007 | None | Vsig10 | V-set and immunoglobulin domain containing 10 |
| ENSMUSG00000067235 | 0.538 | 0.195 | 1.467 | 0.015 | K06751 | H2-Q10 | histocompatibility 2, Q region locus 10 |
| ENSMUSG00000067297 | 1.352 | 0.803 | 0.756 | 0.004 | None | Ifit1bl2 | interferon induced protein with tetratricopeptide repeats 1B like 2 |
| ENSMUSG00000067336 | 7.546 | 5.714 | 0.405 | 0.010 | K04671 | Bmpr2 | bone morphogenetic protein receptor, type II (serine/threonine kinase) |
| ENSMUSG00000067367 | 13.754 | 10.490 | 0.393 | 0.006 | K15263 | Lyar | Ly1 antibody reactive clone |
| ENSMUSG00000067736 | 41.433 | 23.822 | 0.802 | 0.003 | K03882 | Gm10222 | predicted gene 10222 |
| ENSMUSG00000067869 | 3.078 | 1.681 | 0.880 | 0.033 |  | Tcea1-ps1 | transcription elongation factor A (SII) 1, pseudogene 1 |
| ENSMUSG00000068037 | 0.065 | 0.233 | -1.839 | 0.032 | K04303 | Mas1 | MAS1 oncogene |
| ENSMUSG00000068079 | 30.475 | 45.839 | -0.588 | 0.003 | K09070 | Tcf15 | transcription factor 15 |
| ENSMUSG00000068086 | 1.265 | 0.017 | 6.241 | 0.014 | K07414 | Cyp2d9 | cytochrome P450, family 2, subfamily d, polypeptide 9 |
| ENSMUSG00000068184 | 4.014 | 2.967 | 0.439 | 0.021 | K18160 | Ndufaf2 | NADH dehydrogenase (ubiquinone) 1 alpha subcomplex, assembly factor 2 |
| ENSMUSG00000068226 | 0.235 | 1.010 | -2.103 | 0.042 | K15902 | Gm6723 | L antigen family, member 3 pseudogene |
| ENSMUSG00000068250 | 0.437 | 0.285 | 0.622 | 0.025 | None | Amn1 | antagonist of mitotic exit network 1 |
| ENSMUSG00000068735 | 16.809 | 26.937 | -0.678 | 0.015 | None | Trp53i11 | transformation related protein 53 inducible protein 11 |
| ENSMUSG00000068740 | 0.797 | 1.139 | -0.513 | 0.008 | K04601 | Celsr2 | cadherin, EGF LAG seven-pass G-type receptor 2 |
| ENSMUSG00000068798 | 17.432 | 13.127 | 0.413 | 0.010 | K04353 | Rap1a | RAS-related protein 1a |
| ENSMUSG00000069045 | 6.700 | 5.031 | 0.418 | 0.039 | K17642 | Ddx3y | DEAD (Asp-Glu-Ala-Asp) box polypeptide 3, Y-linked |
| ENSMUSG00000069089 | 8.167 | 6.050 | 0.437 | 0.006 | K02202 | Cdk7 | cyclin-dependent kinase 7 |
| ENSMUSG00000069094 | 4.488 | 3.057 | 0.557 | 0.001 | K18436 | Pde7a | phosphodiesterase 7A |
| ENSMUSG00000069495 | 5.166 | 3.553 | 0.544 | 0.001 | K11322 | Epc2 | enhancer of polycomb homolog 2 |
| ENSMUSG00000069792 | 20.537 | 12.617 | 0.708 | 0.015 | None | Wfdc17 | WAP four-disulfide core domain 17 |
| ENSMUSG00000069917 | 24.711 | 72.250 | -1.530 | 0.041 | K13822 | Hba-a2 | hemoglobin alpha, adult chain 2 |
| ENSMUSG00000070283 | 16.591 | 21.722 | -0.386 | 0.001 | K09008 | Ndufaf3 | NADH dehydrogenase (ubiquinone) 1 alpha subcomplex, assembly factor 3 |
| ENSMUSG00000070394 | 19.953 | 26.351 | -0.397 | 0.015 | None | Tmem256 | transmembrane protein 256 |
| ENSMUSG00000070462 | 6.383 | 8.873 | -0.473 | 0.001 | None | Tlnrd1 | talin rod domain containing 1 |
| ENSMUSG00000070576 | 2.035 | 2.658 | -0.383 | 0.022 | None | Mn1 | meningioma 1 |
| ENSMUSG00000070803 | 10.239 | 13.471 | -0.394 | 0.023 | None | Cited4 | Cbp/p300-interacting transactivator, with Glu/Asp-rich carboxy-terminal domain, 4 |
| ENSMUSG00000070873 | 1.375 | 0.812 | 0.760 | 0.033 | K06512 | Lilra5 | leukocyte immunoglobulin-like receptor, subfamily A (with TM domain), member 5 |
| ENSMUSG00000070923 | 14.536 | 10.694 | 0.446 | 0.004 | K10447 | Klhl9 | kelch-like 9 |
| ENSMUSG00000071001 | 8.848 | 12.009 | -0.437 | 0.015 | None | Hrct1 | histidine rich carboxyl terminus 1 |
| ENSMUSG00000071072 | 20.298 | 15.428 | 0.398 | 0.000 | K15730 | Ptges3 | prostaglandin E synthase 3 |
| ENSMUSG00000071178 | 4.782 | 0.267 | 4.156 | 0.013 | K03984 | Serpina1b | serine (or cysteine) preptidase inhibitor, clade A, member 1B |
| ENSMUSG00000071342 | 11.394 | 7.546 | 0.597 | 0.000 | None | Lsmem1 | leucine-rich single-pass membrane protein 1 |
| ENSMUSG00000071454 | 1.386 | 1.865 | -0.426 | 0.049 | None | Dtnb | dystrobrevin, beta |
| ENSMUSG00000071533 | 20.154 | 15.213 | 0.409 | 0.000 | None | Pcnp | PEST proteolytic signal containing nuclear protein |
| ENSMUSG00000071547 | 16.914 | 22.170 | -0.388 | 0.005 | None | Nt5dc2 | 5'-nucleotidase domain containing 2 |
| ENSMUSG00000071691 | 0.296 | 0.080 | 1.908 | 0.044 | None | Gm960 | predicted gene 960 |
| ENSMUSG00000071711 | 23.247 | 33.123 | -0.507 | 0.000 | K01011 | Mpst | mercaptopyruvate sulfurtransferase |
| ENSMUSG00000071713 | 1.673 | 0.821 | 1.031 | 0.004 | K04738 | Csf2rb | colony stimulating factor 2 receptor, beta, low-affinity (granulocyte-macrophage) |
| ENSMUSG00000071714 | 1.025 | 0.553 | 0.894 | 0.019 | K04738 | Csf2rb2 | colony stimulating factor 2 receptor, beta 2, low-affinity (granulocyte-macrophage) |
| ENSMUSG00000072381 | 3.611 | 6.418 | -0.830 | 0.008 | None | Gm10355 | RAN guanine nucleotide release factor pseudogene |
| ENSMUSG00000072501 | 3.267 | 2.382 | 0.460 | 0.016 | K18402 | Phf20l1 | PHD finger protein 20-like 1 |
| ENSMUSG00000072949 | 53.873 | 74.929 | -0.474 | 0.000 | K01068 | Acot1 | acyl-CoA thioesterase 1 |
| ENSMUSG00000073131 | 3.649 | 2.673 | 0.452 | 0.032 | None | Vma21 | VMA21 vacuolar H+-ATPase homolog (S. cerevisiae) |
| ENSMUSG00000073418 | 20.991 | 12.408 | 0.762 | 0.017 | K03989 | C4b | complement component 4B (Chido blood group) |
| ENSMUSG00000073478 | 0.320 | 0.756 | -1.233 | 0.029 |  | D730003I15Rik | RIKEN cDNA D730003I15 gene |
| ENSMUSG00000073489 | 3.738 | 2.116 | 0.823 | 0.000 | None | Ifi204 | interferon activated gene 204 |
| ENSMUSG00000073490 | 6.617 | 4.548 | 0.544 | 0.001 | None | Ifi207 | interferon activated gene 207 |
| ENSMUSG00000073555 | 1.416 | 0.604 | 1.229 | 0.002 | None | Gm4951 | predicted gene 4951 |
| ENSMUSG00000073608 | 0.664 | 0.413 | 0.688 | 0.029 | K09675 | Gal3st2c | galactose-3-O-sulfotransferase 2C |
| ENSMUSG00000073664 | 2.118 | 1.493 | 0.509 | 0.023 | None | Nbeal1 | neurobeachin like 1 |
| ENSMUSG00000073838 | 114.311 | 149.212 | -0.384 | 0.001 | K02358 | Tufm | Tu translation elongation factor, mitochondrial |
| ENSMUSG00000073987 | 6.231 | 4.536 | 0.460 | 0.017 | K01307 | Ggh | gamma-glutamyl hydrolase |
| ENSMUSG00000074064 | 75.050 | 111.176 | -0.565 | 0.000 | K01578 | Mlycd | malonyl-CoA decarboxylase |
| ENSMUSG00000074088 | 0.946 | 0.241 | 1.973 | 0.020 | K12857 | Snrnp40 | small nuclear ribonucleoprotein 40 (U5) |
| ENSMUSG00000074151 | 2.101 | 1.322 | 0.672 | 0.005 |  | Nlrc5 | NLR family, CARD domain containing 5 |
| ENSMUSG00000074217 | 1.304 | 2.244 | -0.780 | 0.023 | None | 2210011C24Rik | RIKEN cDNA 2210011C24 gene |
| ENSMUSG00000074218 | 266.845 | 383.261 | -0.521 | 0.000 | K02270 | Cox7a1 | cytochrome c oxidase subunit VIIa 1 |
| ENSMUSG00000074476 | 0.997 | 1.671 | -0.740 | 0.036 | K11549 | Spc24 | SPC24, NDC80 kinetochore complex component, homolog (S. cerevisiae) |
| ENSMUSG00000074652 | 9.707 | 6.378 | 0.608 | 0.000 | K10352 | Myh7b | myosin, heavy chain 7B, cardiac muscle, beta |
| ENSMUSG00000074738 | 1.470 | 2.131 | -0.532 | 0.041 | None | Fndc10 | fibronectin type III domain containing 10 |
| ENSMUSG00000075229 | 12.058 | 9.160 | 0.400 | 0.024 | None | Ccdc58 | coiled-coil domain containing 58 |
| ENSMUSG00000075700 | 32.848 | 24.376 | 0.433 | 0.001 | None | Selenot | selenoprotein T |
| ENSMUSG00000076431 | 5.280 | 3.796 | 0.480 | 0.020 | K09268 | Sox4 | SRY (sex determining region Y)-box 4 |
| ENSMUSG00000076612 | 3.174 | 6.014 | -0.919 | 0.004 |  | Ighg2c | immunoglobulin heavy constant gamma 2C |
| ENSMUSG00000076613 | 0.674 | 0.138 | 2.278 | 0.005 |  | Ighg2b | immunoglobulin heavy constant gamma 2B |
| ENSMUSG00000076615 | 0.135 | 0.003 | 4.997 | 0.016 |  | Ighg3 | Immunoglobulin heavy constant gamma 3 |
| ENSMUSG00000076937 | 0.051 | 0.589 | -3.522 | 0.014 |  | Iglc2 | immunoglobulin lambda constant 2 |
| ENSMUSG00000078122 | 0.377 | 0.128 | 1.550 | 0.017 |  | F630028O10Rik | RIKEN cDNA F630028O10 gene |
| ENSMUSG00000078234 | 1.889 | 2.815 | -0.574 | 0.020 | None | Klhdc7a | kelch domain containing 7A |
| ENSMUSG00000078427 | 9.444 | 5.480 | 0.790 | 0.015 | K18732 | Sarnp | SAP domain containing ribonucleoprotein |
| ENSMUSG00000078566 | 46.202 | 62.098 | -0.426 | 0.004 | K15464 | Bnip3 | BCL2/adenovirus E1B interacting protein 3 |
| ENSMUSG00000078592 | 0.132 | 0.561 | -2.072 | 0.035 | K00134 | Gm4609 | glyceraldehyde-3-phosphate dehydrogenase pseudogene |
| ENSMUSG00000078606 | 0.212 | 0.107 | 0.983 | 0.028 | None | Gm4070 | predicted gene 4070 |
| ENSMUSG00000078636 | 296.491 | 389.249 | -0.393 | 0.004 | K00134 | Gm7336 | glyceraldehyde-3-phosphate dehydrogenase pseudogene |
| ENSMUSG00000078784 | 9.957 | 6.291 | 0.664 | 0.002 | None | 1810022K09Rik | RIKEN cDNA 1810022K09 gene |
| ENSMUSG00000078896 | 0.750 | 0.164 | 2.191 | 0.016 | None | Zfp965 | zinc finger protein 965 |
| ENSMUSG00000079014 | 0.346 | 0.093 | 1.904 | 0.040 | K04525 | Serpina3i | serine (or cysteine) peptidase inhibitor, clade A, member 3I |
| ENSMUSG00000079038 | 0.722 | 0.336 | 1.102 | 0.029 | None | D130040H23Rik | RIKEN cDNA D130040H23 gene |
| ENSMUSG00000079084 | 5.221 | 3.737 | 0.485 | 0.011 | None | Ccdc82 | coiled-coil domain containing 82 |
| ENSMUSG00000079108 | 4.115 | 3.117 | 0.404 | 0.046 | K03106 | Srp54b | signal recognition particle 54B |
| ENSMUSG00000079110 | 0.276 | 0.623 | -1.177 | 0.010 | K08573 | Capn3 | calpain 3 |
| ENSMUSG00000079139 | 10.046 | 7.705 | 0.386 | 0.041 |  | Gm4204 | nucleosome assembly protein 1-like 1 pseudogene |
| ENSMUSG00000079227 | 0.639 | 0.241 | 1.416 | 0.035 | K04180 | Ccr5 | chemokine (C-C motif) receptor 5 |
| ENSMUSG00000079339 | 3.551 | 2.141 | 0.734 | 0.004 | K14217 | Ifit1bl1 | interferon induced protein with tetratricpeptide repeats 1B like 1 |
| ENSMUSG00000079418 | 0.360 | 0.041 | 3.102 | 0.004 | K08342 | Atg4a | autophagy related 4A, cysteine peptidase |
| ENSMUSG00000079465 | 0.136 | 0.249 | -0.875 | 0.042 | K06237 | Col4a3 | collagen, type IV, alpha 3 |
| ENSMUSG00000079563 | 0.192 | 0.022 | 3.101 | 0.016 | K01446 | Pglyrp2 | peptidoglycan recognition protein 2 |
| ENSMUSG00000079941 | 45.394 | 64.533 | -0.504 | 0.001 | K02265 | Gm11273 | cytochrome c oxidase, subunit Vb pseudogene |
| ENSMUSG00000080845 | 0.037 | 0.332 | -3.125 | 0.018 |  | Gm9115 | ornithine decarboxylase, structural 1 pseudogene |
| ENSMUSG00000081219 | 4.756 | 7.338 | -0.621 | 0.048 |  | Bambi-ps1 | BMP and activin membrane-bound inhibitor, pseudogene (Xenopus laevis) |
| ENSMUSG00000081665 | 0.230 | 0.039 | 2.556 | 0.018 |  |  |  |
| ENSMUSG00000081723 | 1.448 | 0.683 | 1.087 | 0.006 |  | Gm15931 | predicted gene 15931 |
| ENSMUSG00000083282 | 27.417 | 36.673 | -0.417 | 0.000 | K01373 | Ctsf | cathepsin F |
| ENSMUSG00000083816 | 0.511 | 0.099 | 2.372 | 0.011 |  | Gm13033 | prostaglandin-endoperoxide synthase 2 pseudogene |
| ENSMUSG00000084093 | 59.468 | 86.778 | -0.542 | 0.001 |  | Gm16418 | ubiquinol-cytochrome c reductase subunit pseudogene |
| ENSMUSG00000084350 | 0.620 | 0.175 | 1.820 | 0.021 |  | Znf41-ps | ZNF41, pseudogene |
| ENSMUSG00000084792 | 0.406 | 0.050 | 3.086 | 0.033 |  | 1700056N10Rik | RIKEN cDNA 1700056N10 gene |
| ENSMUSG00000084957 | 7.433 | 5.360 | 0.475 | 0.005 |  | Bbip1 | BBSome interacting protein 1 |
| ENSMUSG00000085162 | 3.574 | 2.055 | 0.801 | 0.001 |  | Gm12295 | predicted gene 12295 |
| ENSMUSG00000085438 | 19.395 | 14.806 | 0.394 | 0.016 |  | 1700020I14Rik | RIKEN cDNA 1700020I14 gene |
| ENSMUSG00000085653 | 0.312 | 1.321 | -2.086 | 0.013 |  | Gm15179 | predicted gene 15179 |
| ENSMUSG00000085664 | 2.051 | 0.978 | 1.070 | 0.001 |  | Atxn7l1os2 | ataxin 7-like 1, opposite strand 2 |
| ENSMUSG00000086784 | 24.393 | 34.910 | -0.515 | 0.000 | None | Isoc2a | isochorismatase domain containing 2a |
| ENSMUSG00000087006 | 3.166 | 4.839 | -0.607 | 0.024 |  | Gm13889 | predicted gene 13889 |
| ENSMUSG00000087370 | 2.490 | 1.840 | 0.439 | 0.012 | None | Tmem170b | transmembrane protein 170B |
| ENSMUSG00000087651 | 2.058 | 1.295 | 0.672 | 0.044 | None | 1500009L16Rik | RIKEN cDNA 1500009L16 gene |
| ENSMUSG00000089832 | 2.258 | 3.162 | -0.482 | 0.027 | None | Shkbp1 | Sh3kbp1 binding protein 1 |
| ENSMUSG00000090084 | 4.385 | 2.989 | 0.557 | 0.033 | None | Srpx | sushi-repeat-containing protein |
| ENSMUSG00000090110 | 2.144 | 0.953 | 1.171 | 0.013 | None | Cmc4 | C-x(9)-C motif containing 4 |
| ENSMUSG00000090215 | 0.005 | 0.103 | -4.081 | 0.044 | K11999 | Trim34b | tripartite motif-containing 34B |
| ENSMUSG00000090222 | 2.262 | 1.426 | 0.667 | 0.026 |  | Ifi203-ps | interferon activated gene 203, pseudogene |
| ENSMUSG00000090272 | 6.773 | 4.089 | 0.731 | 0.001 | None | Mndal | myeloid nuclear differentiation antigen like |
| ENSMUSG00000090386 | 2.625 | 1.316 | 0.998 | 0.000 |  | Mir99ahg | Mir99a and Mirlet7c-1 host gene (non-protein coding) |
| ENSMUSG00000090799 | 4.259 | 5.729 | -0.425 | 0.017 | K13957 | Klhl33 | kelch-like 33 |
| ENSMUSG00000090958 | 6.763 | 9.862 | -0.542 | 0.000 | None | Lrrc32 | leucine rich repeat containing 32 |
| ENSMUSG00000091119 | 9.739 | 6.700 | 0.543 | 0.020 | None | Ccdc152 | coiled-coil domain containing 152 |
| ENSMUSG00000091537 | 35.838 | 25.383 | 0.501 | 0.000 | None | Tma7 | translational machinery associated 7 |
| ENSMUSG00000091649 | 2.680 | 1.334 | 1.010 | 0.006 | None | Phf11b | PHD finger protein 11B |
| ENSMUSG00000093805 | 1.005 | 0.505 | 0.991 | 0.006 |  | Gal3st2b | galactose-3-O-sulfotransferase 2B |
| ENSMUSG00000094483 | 6.079 | 4.238 | 0.524 | 0.001 |  | Purb | purine rich element binding protein B |
| ENSMUSG00000094694 | 0.809 | 0.000 | 5.750 | 0.007 |  | Ighv1-9 | immunoglobulin heavy variable V1-9 |
| ENSMUSG00000094796 | 0.368 | 0.115 | 1.665 | 0.048 |  | BC147527 | cDNA sequence BC147527 |
| ENSMUSG00000094806 | 0.633 | 0.017 | 5.192 | 0.001 |  | Cyp2d10 | cytochrome P450, family 2, subfamily d, polypeptide 10 |
| ENSMUSG00000095134 | 0.371 | 0.979 | -1.395 | 0.028 |  | Mid1-ps1 | midline 1, pseudogene 1 |
| ENSMUSG00000095432 | 0.824 | 0.527 | 0.647 | 0.047 |  | Zfp748 | zinc finger protein 748 |
| ENSMUSG00000095654 | 1.797 | 0.903 | 0.990 | 0.019 |  | Plscr5 | phospholipid scramblase family, member 5 |
| ENSMUSG00000096010 | 0.287 | 0.017 | 4.110 | 0.005 |  | Hist4h4 | histone cluster 4, H4 |
| ENSMUSG00000097195 | 5.143 | 3.051 | 0.757 | 0.015 |  | Snhg5 | small nucleolar RNA host gene 5 |
| ENSMUSG00000097287 | 1.145 | 1.810 | -0.660 | 0.016 |  | D130017N08Rik | RIKEN cDNA D130017N08 gene |
| ENSMUSG00000097503 | 0.384 | 0.061 | 2.679 | 0.022 |  | 3110045C21Rik | RIKEN cDNA 3110045C21 gene |
| ENSMUSG00000097776 | 3.453 | 2.437 | 0.504 | 0.033 |  | Gm26795 | predicted gene, 26795 |
| ENSMUSG00000097792 | 1.273 | 0.513 | 1.323 | 0.014 |  | Gm27042 | predicted gene, 27042 |
| ENSMUSG00000098188 | 0.846 | 0.473 | 0.842 | 0.041 |  | Sowahc | sosondowah ankyrin repeat domain family member C |
| ENSMUSG00000098557 | 0.992 | 0.506 | 0.970 | 0.004 |  | Kctd12 | potassium channel tetramerisation domain containing 12 |
| ENSMUSG00000099757 | 0.142 | 0.024 | 2.624 | 0.015 |  | BE692007 | expressed sequence BE692007 |
| ENSMUSG00000100131 | 29.660 | 15.683 | 0.925 | 0.000 |  |  |  |
| ENSMUSG00000100969 | 3.238 | 1.591 | 1.028 | 0.000 |  | 1700030N03Rik | RIKEN cDNA 1700030N03 gene |
| ENSMUSG00000101086 | 1.638 | 0.996 | 0.721 | 0.012 |  | Gm28651 | predicted gene 28651 |
| ENSMUSG00000101389 | 8.076 | 4.104 | 0.980 | 0.004 |  | Ms4a4a | membrane-spanning 4-domains, subfamily A, member 4A |
| ENSMUSG00000101904 | 0.277 | 0.016 | 4.184 | 0.009 |  |  |  |
| ENSMUSG00000102070 | 321.205 | 215.707 | 0.579 | 0.001 |  |  |  |
| ENSMUSG00000103156 | 0.968 | 2.444 | -1.335 | 0.019 |  | Gm38293 | predicted gene, 38293 |
| ENSMUSG00000103520 | 1.577 | 0.654 | 1.281 | 0.043 |  | 1110025M09Rik | RIKEN cDNA 1110025M09 gene |
| ENSMUSG00000103620 | 1.478 | 0.427 | 1.787 | 0.020 |  | Gm37359 | predicted gene, 37359 |
| ENSMUSG00000103649 | 2.598 | 0.923 | 1.492 | 0.000 |  |  |  |
| ENSMUSG00000103735 | 3.080 | 6.043 | -0.969 | 0.033 |  |  |  |
| ENSMUSG00000105444 | 0.569 | 0.125 | 2.178 | 0.024 |  | Gm10727 | predicted gene 10727 |
| ENSMUSG00000105449 | 0.113 | 0.016 | 2.787 | 0.003 |  |  |  |
| ENSMUSG00000105504 | 5.262 | 3.079 | 0.777 | 0.003 |  | Gbp5 | guanylate binding protein 5 |
| ENSMUSG00000106547 | 0.292 | 0.077 | 1.934 | 0.018 |  | B230303O12Rik | RIKEN cDNA B230303O12 gene |
| ENSMUSG00000107002 | 65.053 | 91.683 | -0.493 | 0.000 |  | 0610012G03Rik | RIKEN cDNA 0610012G03 gene |
| ENSMUSG00000107747 | 0.000 | 0.517 | -5.053 | 0.011 |  | Gm5881 | calmodulin 2 pseudogene |
| ENSMUSG00000108628 | 0.016 | 0.436 | -4.597 | 0.030 |  | Gm5037 | frataxin pseudogene |
| ENSMUSG00000108854 | 0.258 | 0.707 | -1.444 | 0.030 |  | D830036C21Rik | RIKEN cDNA D830036C21 gene |
| ENSMUSG00000110221 | 0.758 | 1.399 | -0.879 | 0.046 |  | Gm36210 | predicted gene, 36210 |
| ENSMUSG00000112639 | 0.331 | 0.045 | 2.834 | 0.011 |  |  |  |
| ENSMUSG00000112964 | 5.179 | 3.454 | 0.587 | 0.027 |  | E430024I08Rik | RIKEN cDNA E430024I08 gene |
| ENSMUSG00000113010 | 0.307 | 0.042 | 2.834 | 0.014 |  | Gm34084 | predicted gene, 34084 |
| ENSMUSG00000113216 | 0.329 | 0.149 | 1.130 | 0.041 |  | Gm40841 | predicted gene, 40841 |
| ENSMUSG00000113425 | 0.345 | 0.093 | 1.876 | 0.040 |  |  |  |
| ENSMUSG00000114362 | 1.675 | 0.797 | 1.074 | 0.013 |  |  |  |
| ENSMUSG00000114800 | 0.090 | 0.247 | -1.459 | 0.023 |  |  |  |
| ENSMUSG00000115120 | 0.615 | 0.033 | 4.211 | 0.037 |  |  |  |
| ENSMUSG00000115924 | 13.533 | 10.014 | 0.438 | 0.015 |  |  |  |
| ENSMUSG00000116305 | 4.223 | 2.059 | 1.040 | 0.001 |  |  |  |
| ENSMUSG00000116946 | 1.468 | 0.778 | 0.919 | 0.016 |  |  |  |
| ENSMUSG00000116953 | 0.267 | 1.019 | -1.916 | 0.008 |  |  |  |
| ENSMUSG00000117310 | 0.743 | 1.316 | -0.827 | 0.020 |  |  |  |

**Table SIII. Differentially expressed genes of DCM/CK that enriched in immune system related pathways.**

| symbol | CK-1 | CK-2 | CK-3 | DCM-1 | DCM-2 | DCM-3 | KEGG_ID |
| --- | --- | --- | --- | --- | --- | --- | --- |
| Lck | 1.89793 | 0.902127 | 0.994561 | 0.59918 | 0.164757 | 0.445221 | K05856 |
| Icam2 | 38.24335 | 30.75114 | 27.35628 | 21.42604 | 21.41785 | 19.90786 | K06523 |
| Itgb7 | 2.343404 | 2.039561 | 1.345776 | 1.379437 | 0.792856 | 1.02376 | K06590 |
| Col1a1 | 41.63219 | 40.82103 | 38.6645 | 19.36045 | 17.79271 | 17.4229 | K06236 |
| Cldn15 | 6.822305 | 5.8469 | 8.648169 | 11.21187 | 11.30464 | 9.281591 | K06087 |
| Vwf | 31.4142 | 29.67655 | 32.04363 | 62.95693 | 49.01592 | 39.68883 | K03900 |
| Hck | 3.373112 | 3.178273 | 2.285304 | 1.468261 | 1.016842 | 2.201973 | K08893 |
| Fcer2a | 1.208257 | 1.076026 | 0.193855 | 0.224595 | 0.175165 | 0.293522 | K06468 |
| Kit | 2.188718 | 2.733752 | 2.404898 | 0.912735 | 0.886699 | 1.015422 | K05091 |
| Cd247 | 1.900063 | 2.348038 | 2.001205 | 1.328246 | 0.664052 | 0.919221 | K06453 |
| Tnfrsf13b | 1.543942 | 1.266124 | 0.902522 | 0.620562 | 0.640193 | 0.570896 | K05150 |
| Cybb | 5.890592 | 6.34588 | 4.960007 | 3.957759 | 2.64458 | 3.522307 | K08008 |
| Cd48 | 5.103919 | 5.333326 | 4.002886 | 3.226741 | 2.373098 | 2.098854 | K06479 |
| Fcgr1 | 3.552308 | 2.102249 | 2.285656 | 1.137782 | 0.872206 | 1.281958 | K06498 |
| Cd34 | 23.86784 | 22.71583 | 22.51758 | 16.84225 | 14.06082 | 14.58155 | K06474 |
| Mmp9 | 1.221868 | 1.047078 | 0.831865 | 2.355146 | 1.195683 | 1.432851 | K01403 |
| Dock2 | 2.228166 | 2.015202 | 1.87794 | 1.092093 | 0.944677 | 1.236623 | K12367 |
| Itk | 0.360111 | 0.303016 | 0.219127 | 0.058935 | 0.09959 | 0.113858 | K07363 |
| Ccl11 | 0.927265 | 2.365573 | 1.575841 | 3.280931 | 3.266618 | 2.776578 | K16597 |
| Nfkbia | 41.92077 | 46.44202 | 50.89036 | 58.31571 | 76.79777 | 62.8448 | K04734 |
| Traf3 | 4.628069 | 4.917243 | 4.332973 | 3.120388 | 3.059434 | 3.116516 | K03174 |
| Ocln | 0.214592 | 0.188777 | 0.330035 | 0.652417 | 0.43614 | 0.303631 | K06088 |
| Erbin | 4.164011 | 6.139007 | 4.755832 | 7.123423 | 4.78388 | 6.558875 | K12796 |
| Ciita | 0.27837 | 0.171417 | 0.161402 | 0.0964 | 0.032689 | 0.042868 | K08060 |
| Ifngr2 | 57.86748 | 57.26132 | 53.67422 | 37.56191 | 32.95615 | 38.08756 | K05133 |
| Adcy6 | 43.41323 | 40.99716 | 46.46671 | 54.35295 | 57.57792 | 61.04329 | K08046 |
| Gnb3 | 12.40991 | 9.514523 | 7.298656 | 11.89296 | 15.17553 | 13.29852 | K07825 |
| Tmem173 | 6.305134 | 5.431603 | 4.589352 | 4.057643 | 3.53994 | 2.970574 | K12654 |
| Cd74 | 318.5658 | 258.2085 | 216.0786 | 128.6173 | 78.07981 | 81.69631 | K06505 |
| Csf1r | 33.42913 | 29.9771 | 23.61116 | 21.7655 | 18.97743 | 17.59566 | K05090 |
| Pik3ap1 | 3.63209 | 3.071393 | 2.480274 | 2.281822 | 1.339103 | 1.761578 | K12230 |
| Ifitm1 | 6.180576 | 4.336298 | 4.85189 | 8.649266 | 7.792065 | 8.045089 | K19831 |
| Casp8 | 6.171174 | 6.942465 | 5.85878 | 4.704265 | 4.063931 | 4.38311 | K04398 |
| Col3a1 | 95.34123 | 94.49441 | 89.41586 | 33.79719 | 27.28595 | 28.9925 | K19720 |
| Il1r2 | 0.023568 | 0.020733 | 0 | 0.479056 | 0.276758 | 0.161308 | K04387 |
| Stat1 | 6.239385 | 3.746767 | 4.108485 | 2.732282 | 2.522324 | 3.110601 | K11220 |
| Cxcr2 | 0.241548 | 0.048293 | 0.054879 | 0.297562 | 0.300836 | 0.375732 | K05050 |
| Inpp5d | 5.961142 | 4.761327 | 3.832859 | 3.498374 | 2.585255 | 3.593158 | K03084 |
| Cd55 | 3.394507 | 4.742151 | 4.108373 | 3.198364 | 2.120496 | 2.547954 | K04006 |
| Ncf2 | 2.891584 | 3.124428 | 2.775885 | 2.365921 | 1.481512 | 1.731083 | K08010 |
| Ifih1 | 2.372217 | 2.898984 | 2.426458 | 1.795074 | 1.932489 | 1.694749 | K12647 |
| Itga6 | 30.97585 | 29.1539 | 31.02789 | 16.74188 | 17.13025 | 18.1696 | K06485 |
| Rasgrp1 | 0.524451 | 0.455733 | 0.300503 | 0.317783 | 0.087622 | 0.125851 | K04350 |
| Zbp1 | 2.625178 | 1.433401 | 1.176424 | 0.88593 | 0.649612 | 0.894931 | K12965 |
| Gnas | 3.999308 | 4.956772 | 4.819641 | 6.187423 | 10.29808 | 5.596261 | K04632 |
| Gnb4 | 5.699369 | 5.401292 | 4.983943 | 4.210872 | 3.459849 | 3.835922 | K04538 |
| Tlr2 | 3.450858 | 3.269986 | 2.540164 | 2.313885 | 1.531009 | 1.765702 | K10159 |
| Cxcl9 | 6.274248 | 4.22615 | 3.043531 | 1.159069 | 1.178115 | 1.544987 | K05416 |
| Actb | 322.9897 | 298.3843 | 255.6372 | 232.1405 | 215.9449 | 181.835 | K05692 |
| Col1a2 | 62.84075 | 57.81702 | 59.9823 | 36.71107 | 29.78935 | 30.19558 | K06236 |
| Irf5 | 4.820261 | 4.883661 | 3.90867 | 3.471537 | 2.745477 | 2.987595 | K09446 |
| Tbxas1 | 1.422186 | 1.59934 | 1.187215 | 0.965802 | 0.774766 | 0.890596 | K01832 |
| A2m | 0.733503 | 0.80498 | 0.689705 | 1.208281 | 1.328966 | 1.050038 | K03910 |
| Tyrobp | 38.59711 | 41.0009 | 32.76054 | 23.78537 | 16.66484 | 21.85437 | K07992 |
| Lat | 1.28691 | 0.702185 | 0.439688 | 0.38017 | 0.159409 | 0.236923 | K07362 |
| Itgax | 0.661263 | 0.661698 | 0.528839 | 0.435589 | 0.194128 | 0.261652 | K06462 |
| Cd37 | 4.764462 | 4.202874 | 2.631449 | 2.526625 | 1.442498 | 2.420926 | K06475 |
| Btk | 1.474668 | 1.760574 | 1.158293 | 0.911925 | 0.14173 | 0.698402 | K07370 |
| Casp3 | 2.391567 | 2.522371 | 1.568155 | 1.364847 | 0.843017 | 1.309044 | K02187 |
| Adcy7 | 5.32756 | 4.731146 | 3.797749 | 2.741513 | 2.111753 | 2.730902 | K08047 |
| Mmp2 | 63.97483 | 53.04797 | 53.1141 | 40.11318 | 35.63997 | 35.84398 | K01398 |
| Thy1 | 10.73657 | 9.885837 | 8.555651 | 6.559098 | 5.941731 | 4.864859 | K06514 |
| Nck1 | 6.799254 | 6.908075 | 7.200398 | 4.831793 | 4.372331 | 5.823392 | K07365 |
| Gng11 | 56.26054 | 55.31878 | 49.40306 | 35.99855 | 28.91628 | 30.91587 | K04546 |
| Klra9 | 1.438143 | 1.380146 | 0.914886 | 0.393699 | 0.255877 | 0.178965 | None |
| Rac2 | 6.219717 | 5.374643 | 3.884691 | 4.060818 | 2.596158 | 3.032605 | K07860 |
| Tbxa2r | 3.433216 | 3.449467 | 2.543592 | 2.309101 | 1.603078 | 1.565532 | K04264 |
| Isg15 | 22.73795 | 14.71006 | 14.32824 | 11.42293 | 11.21192 | 7.190839 | K12159 |
| Card11 | 0.480438 | 0.365334 | 0.341896 | 0.22069 | 0.151402 | 0.125399 | K07367 |
| H2-Aa | 193.5697 | 160.4852 | 141.7044 | 79.13429 | 44.03068 | 45.95103 | K06752 |
| Asap3 | 4.757269 | 5.487113 | 5.127644 | 3.754214 | 3.423714 | 3.000024 | K12488 |
| Serpine1 | 17.83794 | 15.7018 | 20.94115 | 32.39322 | 34.73745 | 32.565 | K03982 |
| H2-DMb2 | 2.277667 | 2.221883 | 0.69886 | 0.984673 | 0.30895 | 0.655972 | K06752 |
| H2-DMa | 12.56008 | 9.72472 | 8.299014 | 3.481074 | 2.462393 | 3.403996 | K06752 |
| Ctss | 32.08588 | 29.33327 | 26.37594 | 19.60281 | 14.92626 | 14.04204 | K01368 |
| Stat2 | 11.43174 | 8.162962 | 8.31822 | 6.67075 | 4.912351 | 5.986585 | K11221 |
| Cd79b | 4.557272 | 3.227784 | 1.355058 | 1.706622 | 0.503135 | 0.679809 | K06507 |
| Elmo1 | 4.284472 | 4.248256 | 3.457063 | 2.810764 | 2.599447 | 2.633935 | K12366 |
| Pik3r1 | 9.647937 | 10.14173 | 12.44614 | 16.42368 | 19.0572 | 23.08507 | K02649 |
| Itga1 | 5.199319 | 6.161941 | 5.45371 | 4.244771 | 3.342197 | 4.049036 | K06480 |
| Vsig4 | 2.662391 | 2.896813 | 0.980564 | 3.81524 | 5.553479 | 3.636438 | K19822 |
| Mylk4 | 25.045 | 25.99279 | 25.46899 | 16.54519 | 15.76708 | 18.61755 | K00907 |
| Cxcr4 | 9.301146 | 8.361507 | 7.538532 | 6.834773 | 5.112976 | 4.676195 | K04189 |
| Gm5637 | 2.570267 | 3.444178 | 3.734646 | 1.934967 | 1.813274 | 1.840985 |  |
| Ptgs1 | 7.675526 | 7.152362 | 7.486467 | 10.63461 | 10.09082 | 9.051642 | K00509 |
| Foxo3 | 23.08022 | 20.42395 | 25.09394 | 27.91978 | 34.87245 | 33.87644 | K09408 |
| F2rl3 | 1.413369 | 0.840095 | 0.687363 | 0.532011 | 0.280354 | 0.245105 | K04236 |
| Cx3cr1 | 4.704252 | 3.044815 | 2.009079 | 0.930201 | 0.772096 | 0.81512 | K04192 |
| Prkcb | 2.994628 | 3.023341 | 2.75256 | 1.694611 | 1.396391 | 1.796845 | K19662 |
| Cd8a | 0.320748 | 0.554919 | 0.502343 | 0.257566 | 0.0837 | 0.192088 | K06458 |
| Sh3bp2 | 3.031287 | 2.76359 | 2.66667 | 2.099472 | 1.876876 | 1.452289 | K07984 |
| H2-Q5 | 16.63489 | 11.22342 | 10.20908 | 8.624671 | 6.567983 | 7.301498 |  |
| Ticam2 | 0.285841 | 0.46519 | 0.50006 | 0.18291 | 0.013986 | 0.036682 | K05409 |
| Sipa1 | 21.22633 | 17.47865 | 14.53815 | 12.11988 | 12.33596 | 10.78497 | K08013 |
| Fcer1g | 28.0082 | 28.77399 | 20.70567 | 18.28379 | 12.93734 | 16.21205 | K07983 |
| Ptk2b | 5.264386 | 4.36853 | 4.508505 | 7.22733 | 6.920142 | 5.851481 | K05871 |
| H2-Eb1 | 181.5658 | 146.3551 | 130.5112 | 78.35938 | 41.95869 | 44.63371 | K06752 |
| Blnk | 1.622748 | 1.388598 | 1.179799 | 0.660801 | 0.591881 | 0.814059 | K07371 |
| Gng8 | 2.131555 | 1.842799 | 2.461518 | 0.940682 | 0.827158 | 0.848927 | K04544 |
| Nck2 | 12.33013 | 11.74894 | 10.95121 | 8.09263 | 9.055324 | 6.667869 | K19862 |
| Afdn | 26.73479 | 27.31977 | 26.00838 | 18.35855 | 18.43309 | 18.13966 | K05702 |
| Marcks | 10.07927 | 11.45408 | 8.85368 | 6.393782 | 5.94222 | 7.167129 | K12561 |
| Rasgrp3 | 10.16055 | 12.50355 | 11.2764 | 8.491927 | 7.502174 | 7.219673 | K12362 |
| Ncf4 | 2.467133 | 2.974165 | 1.689897 | 1.100637 | 1.028297 | 1.622127 | K08012 |
| H2-Ab1 | 206.0177 | 152.3481 | 134.7793 | 74.57486 | 46.4281 | 42.17371 | K06752 |
| Ighm | 29.36634 | 23.14798 | 14.33386 | 13.4337 | 8.094937 | 8.256693 |  |
| C7 | 9.62309 | 11.14798 | 10.21967 | 5.607449 | 5.870256 | 5.17496 | K03996 |
| H2-DMb1 | 12.26733 | 9.073298 | 7.643768 | 5.415581 | 2.797152 | 3.240244 | K06752 |

**Table SⅣ. Differentially expressed genes of SPM/DCM that enriched in immune system related pathways.**

| symbol | DCM-1 | DCM-2 | DCM-3 | SPM-1 | SPM-2 | SPM-3 | KEGG_ID |
| --- | --- | --- | --- | --- | --- | --- | --- |
| Itgb2 | 6.247643 | 4.418652 | 5.356315 | 8.184873 | 7.614561 | 7.135711 | K06464 |
| Cldn15 | 11.21187 | 11.30464 | 9.281591 | 7.844547 | 7.943345 | 8.068717 | K06087 |
| Hck | 1.468261 | 1.016842 | 2.201973 | 3.172993 | 2.897774 | 2.733875 | K08893 |
| Ptpn6 | 3.872556 | 2.694137 | 3.644679 | 5.505933 | 4.980972 | 4.4742 | K05697 |
| Cd33 | 1.125232 | 0.92558 | 1.39863 | 2.249842 | 2.094736 | 1.525448 | K06473 |
| Cd244 | 0.133921 | 0.113151 | 0.083705 | 0.286281 | 0.325796 | 0.329568 | K06582 |
| Epor | 3.03097 | 2.564612 | 2.924563 | 1.901676 | 1.890668 | 1.659955 | K05079 |
| Actn3 | 0.38465 | 0.193178 | 0.248368 | 0.072675 | 0.065438 | 0.13239 | K05699 |
| Ppp1cb | 30.764 | 27.08377 | 36.64559 | 43.97805 | 43.86978 | 44.31904 | K06269 |
| Cybb | 3.957759 | 2.64458 | 3.522307 | 7.154213 | 6.048024 | 5.095026 | K08008 |
| Cd48 | 3.226741 | 2.373098 | 2.098854 | 6.075894 | 5.058451 | 3.884643 | K06479 |
| Gzmb | 0.350656 | 0.02279 | 0.059774 | 0.832882 | 0.962425 | 0.840808 | K01353 |
| Map2k3 | 43.96251 | 43.70009 | 41.17364 | 28.05038 | 32.62474 | 34.82386 | K04432 |
| Ccl9 | 4.891354 | 3.560087 | 3.788278 | 7.529822 | 5.809713 | 4.42887 | K05510 |
| Ppp1r12a | 9.154395 | 6.653864 | 8.127245 | 10.83641 | 11.47032 | 10.49297 | K06270 |
| Kitl | 8.572151 | 5.493075 | 7.112466 | 9.026253 | 9.103803 | 9.771764 | K05461 |
| Hsp90b1 | 18.25377 | 14.67723 | 21.3835 | 30.58718 | 31.1761 | 30.99465 | K09487 |
| Dock2 | 1.092093 | 0.944677 | 1.236623 | 1.74952 | 1.676118 | 1.457555 | K12367 |
| Hsp90aa1 | 16.25268 | 14.16394 | 16.87417 | 20.93305 | 22.94725 | 23.15481 | K04079 |
| F12 | 0 | 0 | 0 | 0.488171 | 0.14423 | 0.016211 | K01328 |
| Cxcl14 | 12.4714 | 12.68226 | 13.45248 | 7.020961 | 8.561693 | 10.48689 | K10033 |
| Mapk8 | 3.175565 | 2.717747 | 3.429857 | 3.807685 | 4.360084 | 4.228398 | K04440 |
| Cpb2 | 0.017046 | 0.022158 | 0.058115 | 0.769274 | 0.425322 | 0.107562 | K01300 |
| C6 | 0.078788 | 0.068275 | 0.198971 | 1.091637 | 0.349486 | 0.165718 | K03995 |
| Tfrc | 2.105812 | 1.780224 | 3.311733 | 6.169644 | 5.941577 | 5.763451 | K06503 |
| Cxcl13 | 2.25499 | 2.162806 | 2.438237 | 51.14278 | 26.65759 | 4.973275 | K10032 |
| Gnb3 | 11.89296 | 15.17553 | 13.29852 | 9.459299 | 8.473571 | 8.829854 | K07825 |
| C3 | 92.51201 | 84.44833 | 74.87802 | 172.0557 | 140.2139 | 110.3171 | K03990 |
| Map3k8 | 2.724494 | 1.566906 | 1.704022 | 3.619713 | 3.10555 | 2.572569 | K04415 |
| Il33 | 3.711243 | 2.907323 | 3.047895 | 4.724836 | 4.494208 | 4.08412 | K12967 |
| Pik3ap1 | 2.281822 | 1.339103 | 1.761578 | 4.939578 | 3.4473 | 2.746026 | K12230 |
| Ifitm1 | 8.649266 | 7.792065 | 8.045089 | 21.01846 | 13.31901 | 8.934103 | K19831 |
| Casp1 | 1.345468 | 0.546538 | 1.184998 | 2.356883 | 1.993291 | 1.400845 | K01370 |
| Il1r1 | 4.015178 | 3.650045 | 3.604154 | 5.385063 | 5.030792 | 4.826938 | K04386 |
| Stat1 | 2.732282 | 2.522324 | 3.110601 | 3.389996 | 3.84165 | 4.50914 | K11220 |
| Ptprc | 1.43869 | 0.473225 | 1.071703 | 2.35974 | 2.15055 | 2.070273 | K06478 |
| Cd55 | 3.198364 | 2.120496 | 2.547954 | 3.874748 | 4.040299 | 3.853817 | K04006 |
| F5 | 0.132154 | 0.026428 | 0.107824 | 0.273652 | 0.219828 | 0.196715 | K03902 |
| Fcgr2b | 4.511129 | 3.509068 | 4.004384 | 9.719336 | 6.505358 | 4.402057 | K12560 |
| Serpinc1 | 0 | 0.026339 | 0.011514 | 0.986655 | 0.473995 | 0.006393 | K03911 |
| Apbb1ip | 1.438335 | 1.160463 | 1.324563 | 2.849416 | 2.281696 | 1.877786 | K17704 |
| Hc | 0 | 0 | 0 | 0.280059 | 0.178827 | 0.023342 | K03994 |
| Ifih1 | 1.795074 | 1.932489 | 1.694749 | 2.361425 | 2.302639 | 2.642968 | K12647 |
| Itga4 | 0.38151 | 0.080418 | 0.290016 | 0.450022 | 0.598164 | 0.562798 | K06483 |
| Traf6 | 3.330279 | 2.960202 | 3.84956 | 4.163477 | 4.450056 | 4.553081 | K03175 |
| Pdia3 | 41.23768 | 39.75641 | 43.33112 | 55.83937 | 54.85989 | 51.85881 | K08056 |
| F2 | 0 | 0 | 0.009034 | 1.737191 | 0.922367 | 0 | K01313 |
| Polr3f | 2.544582 | 2.222411 | 2.488845 | 3.592401 | 3.745238 | 3.514375 | K03025 |
| Zbp1 | 0.88593 | 0.649612 | 0.894931 | 1.285944 | 1.624821 | 1.72008 | K12965 |
| Gnas | 6.187423 | 10.29808 | 5.596261 | 4.24985 | 4.122223 | 3.984794 | K04632 |
| Src | 3.093956 | 3.066555 | 2.080338 | 1.622861 | 1.881709 | 2.07432 | K05704 |
| Tlr2 | 2.313885 | 1.531009 | 1.765702 | 3.531706 | 3.095152 | 2.530089 | K10159 |
| Fga | 0 | 0 | 0 | 4.415143 | 1.894074 | 0 | K03903 |
| Dapp1 | 2.131435 | 1.921352 | 3.071866 | 2.977438 | 3.576041 | 3.782338 | K12229 |
| Cd72 | 0.662811 | 0.468791 | 0.830777 | 2.454083 | 1.471397 | 0.762667 | K06504 |
| Csf3r | 1.808178 | 1.11878 | 2.663108 | 3.59905 | 3.473966 | 2.829603 | K05061 |
| Fgr | 0.411757 | 0.321136 | 0.859295 | 1.467016 | 1.167487 | 1.360515 | K08891 |
| Wasl | 7.185184 | 5.474499 | 6.82427 | 8.567525 | 8.451559 | 8.447191 | K05747 |
| Tyrobp | 23.78537 | 16.66484 | 21.85437 | 37.5133 | 27.25362 | 24.26936 | K07992 |
| Nfkbib | 16.56483 | 17.43517 | 14.7294 | 11.89635 | 11.17107 | 11.23873 | K02581 |
| Itgal | 1.176408 | 0.582838 | 1.068429 | 1.482999 | 1.888694 | 1.940983 | K05718 |
| F10 | 0.120705 | 0 | 0.091449 | 1.027343 | 0.514508 | 0.190414 | K01314 |
| Nlrx1 | 6.621933 | 7.626412 | 6.589565 | 4.726701 | 5.376266 | 5.906151 | K12653 |
| Rac2 | 4.060818 | 2.596158 | 3.032605 | 6.259608 | 4.786985 | 3.827905 | K07860 |
| Fgb | 0.006608 | 0 | 0 | 3.908249 | 1.929122 | 0 | K03904 |
| Fgg | 0 | 0 | 0 | 4.585538 | 1.94847 | 0.146002 | K03905 |
| Vav1 | 1.665442 | 1.336459 | 1.516058 | 2.633824 | 2.459377 | 1.747929 | K05730 |
| Ccl12 | 1.172117 | 0.731322 | 0.852498 | 4.510125 | 2.749112 | 2.425925 | K14624 |
| Ccl7 | 1.603034 | 0.245144 | 1.428812 | 5.636665 | 3.41158 | 1.824707 | K05509 |
| Polr3g | 0.601809 | 0.975156 | 0.768232 | 1.674191 | 1.481025 | 1.664634 | K03024 |
| C1qa | 72.1662 | 52.55149 | 56.8938 | 130.6132 | 99.69676 | 69.41332 | K03986 |
| C1qc | 52.53071 | 41.07837 | 42.76336 | 89.80079 | 69.66089 | 49.33772 | K03988 |
| Serpine1 | 32.39322 | 34.73745 | 32.565 | 17.9939 | 22.24054 | 26.59241 | K03982 |
| Mavs | 29.03146 | 28.69071 | 26.89918 | 20.61831 | 20.43658 | 21.35916 | K12648 |
| Aim2 | 0.345863 | 0.380409 | 0.382971 | 1.242781 | 0.81872 | 0.369332 | K12966 |
| Serpinf2 | 0 | 0 | 0.013053 | 1.050335 | 0.573176 | 0 | K03983 |
| Ctss | 19.60281 | 14.92626 | 14.04204 | 35.96882 | 28.99926 | 20.09599 | K01368 |
| Tlr4 | 4.816805 | 4.101872 | 5.249388 | 7.09925 | 5.80127 | 5.806085 | K10160 |
| F13a1 | 10.36516 | 9.606008 | 10.1503 | 18.55917 | 14.91024 | 11.8669 | K03917 |
| Azi2 | 11.98061 | 11.97858 | 13.50559 | 16.042 | 16.88892 | 16.4108 | K12651 |
| Tnfsf10 | 5.264342 | 3.722962 | 4.882315 | 7.132274 | 7.624474 | 8.158199 | K04721 |
| Il7 | 0.243582 | 0.127996 | 0.188469 | 0.455462 | 0.381473 | 0.392429 | K05431 |
| C3ar1 | 1.924101 | 1.573617 | 2.193252 | 4.438186 | 3.275986 | 2.37657 | K04009 |
| Cldn5 | 65.43193 | 86.16175 | 62.03301 | 25.82504 | 32.56354 | 37.54054 | K06087 |
| Ikbke | 0.614809 | 0.257074 | 0.415304 | 1.128415 | 0.922563 | 0.602268 | K07211 |
| Gng2 | 1.77674 | 2.82274 | 2.722101 | 3.993501 | 4.318268 | 4.094207 | K07826 |
| Tlr7 | 0.949712 | 0.76421 | 1.343595 | 2.631731 | 2.321298 | 1.90789 | K05404 |
| Tlr1 | 0.349566 | 0.302925 | 0.425633 | 0.929138 | 0.747611 | 0.48317 | K05398 |
| Mylk4 | 16.54519 | 15.76708 | 18.61755 | 30.53915 | 26.53308 | 22.46003 | K00907 |
| Ccr2 | 0.536243 | 0.2971 | 0.44956 | 1.372874 | 0.954147 | 0.693386 | K04177 |
| C5ar1 | 3.261124 | 3.053631 | 3.018901 | 6.038951 | 5.020664 | 3.965458 | K04010 |
| Cx3cr1 | 0.930201 | 0.772096 | 0.81512 | 1.523972 | 1.489059 | 1.414365 | K04192 |
| Prkcb | 1.694611 | 1.396391 | 1.796845 | 3.072703 | 2.548293 | 2.186352 | K19662 |
| Prkg1 | 2.502269 | 1.990023 | 2.141877 | 3.315482 | 3.643375 | 3.515078 | K07376 |
| H2-Q5 | 8.624671 | 6.567983 | 7.301498 | 12.33782 | 12.00973 | 9.867439 |  |
| Nod2 | 0.93367 | 1.393952 | 1.127745 | 0.703317 | 0.785418 | 0.875309 | K10165 |
| Sphk2 | 8.408914 | 9.675953 | 8.241667 | 5.789632 | 6.258996 | 6.671761 | K04718 |
| Pirb | 2.199043 | 1.540482 | 2.289556 | 5.129367 | 3.540204 | 2.46778 | K06512 |
| Fcgr4 | 1.36309 | 0.573238 | 0.615066 | 4.523158 | 4.40141 | 3.415167 | K06463 |
| Plg | 0.009132 | 0 | 0 | 2.960763 | 1.24182 | 0.011525 | K01315 |
| Hspa2 | 4.515128 | 4.923253 | 4.440179 | 3.1605 | 3.058938 | 3.169823 | K03283 |
| Xcr1 | 0.096117 | 0.044096 | 0.083529 | 0.033573 | 0.007054 | 0.007135 | K04193 |
| Cfl2 | 67.15598 | 56.39229 | 72.78535 | 92.54903 | 86.96528 | 81.62816 | K05765 |
| Gng8 | 0.940682 | 0.827158 | 0.848927 | 1.544309 | 1.760344 | 1.641059 | K04544 |
| Tank | 3.424753 | 2.741375 | 3.329933 | 4.477311 | 3.9932 | 4.039429 | K12650 |
| Serpina1a | 0 | 0 | 0.018097 | 3.801386 | 2.006589 | 0 | K03984 |
| H2-Q10 | 0.255959 | 0.090739 | 0.237992 | 0.84285 | 0.493504 | 0.278974 | K06751 |
| Rap1a | 13.53516 | 10.61883 | 15.22723 | 17.4409 | 17.77337 | 17.0815 | K04353 |
| Serpina1b | 0.085733 | 0.390045 | 0.324767 | 9.689649 | 4.456593 | 0.19836 | K03984 |
| Ighv1-9 | 0 | 0 | 0 | 0.085186 | 1.073848 | 1.267327 |  |

**Table SⅤ. Differentially expressed genes of DCM/CK that involved in signal transduction pathway.**

| symbol | CK-1 | CK-2 | CK-3 | DCM-1 | DCM-2 | DCM-3 |
| --- | --- | --- | --- | --- | --- | --- |
| Lck | 1.89793 | 0.902127 | 0.994561 | 0.59918 | 0.164757 | 0.445221 |
| Col6a1 | 52.18796 | 46.93585 | 48.24888 | 31.54503 | 32.12802 | 28.51427 |
| Itgb7 | 2.343404 | 2.039561 | 1.345776 | 1.379437 | 0.792856 | 1.02376 |
| Col1a1 | 41.63219 | 40.82103 | 38.6645 | 19.36045 | 17.79271 | 17.4229 |
| Vwf | 31.4142 | 29.67655 | 32.04363 | 62.95693 | 49.01592 | 39.68883 |
| Irf9 | 14.10689 | 11.95359 | 11.42358 | 7.405403 | 6.276349 | 6.921893 |
| Relb | 5.895759 | 5.995484 | 5.158399 | 3.907715 | 3.859608 | 3.444276 |
| Hmox1 | 14.64346 | 11.49428 | 13.14715 | 19.27193 | 16.37377 | 17.10518 |
| Kit | 2.188718 | 2.733752 | 2.404898 | 0.912735 | 0.886699 | 1.015422 |
| Pdk1 | 41.48009 | 41.19384 | 42.60797 | 48.85371 | 54.70436 | 57.47778 |
| Bcl2l1 | 29.22387 | 22.22548 | 28.87594 | 42.59905 | 50.9379 | 48.44425 |
| Slc3a2 | 22.35106 | 21.04276 | 21.59508 | 29.32999 | 28.71292 | 26.74044 |
| Dll1 | 4.426739 | 4.14067 | 4.313276 | 2.966971 | 2.595469 | 2.65266 |
| Gadd45b | 7.39713 | 7.286291 | 6.327189 | 12.5101 | 10.83243 | 13.45921 |
| Cybb | 5.890592 | 6.34588 | 4.960007 | 3.957759 | 2.64458 | 3.522307 |
| Wnt11 | 3.866677 | 3.930071 | 3.846052 | 6.062633 | 6.325324 | 6.002627 |
| Cacna1d | 0.647537 | 0.816379 | 0.733868 | 0.359747 | 0.355798 | 0.388089 |
| Mmp9 | 1.221868 | 1.047078 | 0.831865 | 2.355146 | 1.195683 | 1.432851 |
| Hnf4a | 0.053669 | 0.040468 | 0.045987 | 0 | 0 | 0 |
| Cyth4 | 10.88253 | 9.507144 | 6.10601 | 5.423999 | 4.009281 | 4.167596 |
| Grm1 | 2.688016 | 3.167014 | 3.121337 | 3.792909 | 4.212438 | 4.562226 |
| Sgk1 | 36.54357 | 48.14978 | 46.61768 | 51.11856 | 69.10022 | 58.53625 |
| Ddit4 | 54.63369 | 77.2299 | 95.28754 | 109.861 | 167.1205 | 131.3705 |
| Grb10 | 21.38366 | 21.31665 | 21.60135 | 14.17298 | 15.64233 | 16.23663 |
| Col6a2 | 39.80931 | 37.99699 | 35.1069 | 20.41024 | 20.56744 | 19.49598 |
| Hcn2 | 14.98933 | 13.48998 | 12.98585 | 8.597241 | 8.337876 | 8.570158 |
| Acox1 | 119.9918 | 115.297 | 120.9341 | 146.9452 | 158.9628 | 156.0275 |
| Llgl2 | 7.971964 | 6.133264 | 6.812392 | 8.545705 | 10.16593 | 8.539231 |
| Atp2a3 | 12.53218 | 11.93795 | 11.27968 | 18.06015 | 16.5836 | 14.11342 |
| Nos2 | 8.36084 | 7.025176 | 7.464902 | 4.942922 | 5.113543 | 4.962047 |
| Cacna1g | 3.039943 | 3.565649 | 3.326106 | 2.087327 | 1.850464 | 2.04103 |
| Plcd3 | 7.058176 | 6.452378 | 5.67347 | 8.678302 | 8.628237 | 9.180817 |
| Nfkbia | 41.92077 | 46.44202 | 50.89036 | 58.31571 | 76.79777 | 62.8448 |
| Traf3 | 4.628069 | 4.917243 | 4.332973 | 3.120388 | 3.059434 | 3.116516 |
| Fbp2 | 29.73841 | 33.17911 | 32.46958 | 51.1909 | 54.6492 | 47.10198 |
| Ppara | 7.339314 | 6.689133 | 9.501781 | 10.90293 | 10.51895 | 12.30658 |
| Fgf12 | 1.317686 | 0.97427 | 1.123308 | 0.334721 | 0.711965 | 0.753857 |
| Itgb5 | 83.08872 | 72.27739 | 76.74615 | 90.59756 | 102.7769 | 90.64816 |
| Ifngr2 | 57.86748 | 57.26132 | 53.67422 | 37.56191 | 32.95615 | 38.08756 |
| Adcy6 | 43.41323 | 40.99716 | 46.46671 | 54.35295 | 57.57792 | 61.04329 |
| Cdkn1a | 28.52333 | 32.85718 | 41.2442 | 55.00222 | 70.62983 | 68.22854 |
| Gnb3 | 12.40991 | 9.514523 | 7.298656 | 11.89296 | 15.17553 | 13.29852 |
| Thbs2 | 9.570645 | 9.098028 | 7.843464 | 16.08001 | 17.34319 | 16.60902 |
| Cacna1h | 4.620304 | 4.489874 | 4.411635 | 5.977642 | 7.376425 | 5.993565 |
| Lama3 | 0.621435 | 0.619195 | 0.668778 | 0.324619 | 0.369214 | 0.40417 |
| Impa2 | 24.44325 | 27.09981 | 26.50853 | 36.10583 | 36.80519 | 34.81324 |
| Csf1r | 33.42913 | 29.9771 | 23.61116 | 21.7655 | 18.97743 | 17.59566 |
| Cpt1a | 32.89116 | 32.69054 | 35.63288 | 46.92889 | 47.46129 | 47.22306 |
| Pik3ap1 | 3.63209 | 3.071393 | 2.480274 | 2.281822 | 1.339103 | 1.761578 |
| Pi4k2a | 11.99917 | 11.27342 | 10.62997 | 15.72315 | 14.97911 | 13.05028 |
| Limd1 | 24.28921 | 20.63899 | 20.91604 | 27.53482 | 27.04271 | 25.85583 |
| Agap2 | 2.178895 | 1.676524 | 1.833953 | 2.949914 | 3.451029 | 3.215228 |
| Pfkfb4 | 3.16408 | 3.163 | 2.904261 | 2.468623 | 1.932825 | 1.935902 |
| Casp8 | 6.171174 | 6.942465 | 5.85878 | 4.704265 | 4.063931 | 4.38311 |
| Il1r2 | 0.023568 | 0.020733 | 0 | 0.479056 | 0.276758 | 0.161308 |
| Map4k4 | 42.94077 | 44.12 | 40.98986 | 29.38341 | 30.7796 | 30.11135 |
| Stat1 | 6.239385 | 3.746767 | 4.108485 | 2.732282 | 2.522324 | 3.110601 |
| Cxcr2 | 0.241548 | 0.048293 | 0.054879 | 0.297562 | 0.300836 | 0.375732 |
| Inpp5d | 5.961142 | 4.761327 | 3.832859 | 3.498374 | 2.585255 | 3.593158 |
| Cacna1s | 2.61619 | 2.386178 | 3.016471 | 3.794081 | 3.999869 | 3.423733 |
| Pfkfb3 | 5.851369 | 6.02374 | 4.526382 | 8.637083 | 6.394006 | 7.807057 |
| Itgb6 | 2.45641 | 3.238294 | 3.053868 | 1.032019 | 0.823955 | 1.08947 |
| Itga6 | 30.97585 | 29.1539 | 31.02789 | 16.74188 | 17.13025 | 18.1696 |
| Cat | 132.0968 | 130.4258 | 133.5511 | 159.4837 | 164.7355 | 168.1569 |
| Rassf2 | 2.973465 | 3.008835 | 2.850681 | 2.051963 | 1.753002 | 1.675084 |
| Rasgrp1 | 0.524451 | 0.455733 | 0.300503 | 0.317783 | 0.087622 | 0.125851 |
| Gnas | 3.999308 | 4.956772 | 4.819641 | 6.187423 | 10.29808 | 5.596261 |
| Gnb4 | 5.699369 | 5.401292 | 4.983943 | 4.210872 | 3.459849 | 3.835922 |
| Smad9 | 0.959441 | 0.871423 | 1.015184 | 1.439896 | 1.347354 | 1.289884 |
| Efna1 | 37.52132 | 29.00549 | 26.59035 | 24.89938 | 19.85258 | 21.03821 |
| Lef1 | 0.356754 | 0.32993 | 0.137169 | 0.13773 | 0.053709 | 0.062608 |
| Tlr2 | 3.450858 | 3.269986 | 2.540164 | 2.313885 | 1.531009 | 1.765702 |
| Sfrp2 | 6.027486 | 5.978027 | 5.574874 | 4.235982 | 4.721922 | 2.799773 |
| Pla2g12a | 17.4594 | 17.55177 | 16.29167 | 21.23053 | 22.49959 | 20.46444 |
| Cntfr | 4.257952 | 2.949385 | 3.167295 | 5.350916 | 4.937709 | 4.503338 |
| Tnfrsf1b | 9.293716 | 8.776719 | 6.777579 | 6.172273 | 4.517275 | 5.425633 |
| Slc2a1 | 2.769849 | 2.599078 | 2.39976 | 3.483085 | 3.433289 | 3.730088 |
| Map3k6 | 5.447857 | 3.489142 | 5.075824 | 8.92874 | 10.63957 | 11.26918 |
| Ctnnbip1 | 17.24697 | 16.63546 | 13.95315 | 10.73368 | 10.96601 | 11.56677 |
| Ppp2r2c | 0.916256 | 1.165866 | 1.733782 | 0.35105 | 0.608422 | 0.433939 |
| Cds1 | 1.585236 | 1.222089 | 1.593244 | 2.418898 | 2.702202 | 2.151002 |
| Actb | 322.9897 | 298.3843 | 255.6372 | 232.1405 | 215.9449 | 181.835 |
| Col1a2 | 62.84075 | 57.81702 | 59.9823 | 36.71107 | 29.78935 | 30.19558 |
| Wnt5b | 6.502484 | 5.534109 | 6.570886 | 4.651015 | 4.085658 | 4.078805 |
| Lat | 1.28691 | 0.702185 | 0.439688 | 0.38017 | 0.159409 | 0.236923 |
| Il21r | 1.324887 | 1.271458 | 0.923108 | 0.503743 | 0.235726 | 0.412177 |
| Btk | 1.474668 | 1.760574 | 1.158293 | 0.911925 | 0.14173 | 0.698402 |
| Col4a5 | 6.759284 | 7.508071 | 7.892661 | 5.088573 | 4.706713 | 5.397576 |
| Eif4ebp1 | 84.20292 | 75.5465 | 76.50815 | 112.7232 | 120.8609 | 104.3757 |
| Col4a1 | 126.7562 | 116.4174 | 110.7545 | 59.02434 | 60.5258 | 59.11843 |
| Col4a2 | 106.4795 | 98.77405 | 95.19726 | 58.41861 | 60.84117 | 57.6631 |
| Ptpn7 | 1.019397 | 0.715837 | 0.598925 | 0.491424 | 0.420021 | 0.275409 |
| Vegfc | 7.00549 | 7.352026 | 6.511772 | 5.315045 | 5.01123 | 4.568101 |
| Dusp4 | 2.624205 | 1.61059 | 2.794181 | 14.90409 | 15.80197 | 15.80968 |
| Casp3 | 2.391567 | 2.522371 | 1.568155 | 1.364847 | 0.843017 | 1.309044 |
| Adcy7 | 5.32756 | 4.731146 | 3.797749 | 2.741513 | 2.111753 | 2.730902 |
| Mmp15 | 37.52039 | 35.01767 | 30.26834 | 19.57781 | 20.50914 | 20.45463 |
| Ets1 | 29.48019 | 30.09191 | 28.09519 | 18.20877 | 17.54871 | 17.91627 |
| Il10ra | 4.99069 | 4.751963 | 4.071546 | 3.073359 | 2.170551 | 2.412959 |
| Nck1 | 6.799254 | 6.908075 | 7.200398 | 4.831793 | 4.372331 | 5.823392 |
| Gng11 | 56.26054 | 55.31878 | 49.40306 | 35.99855 | 28.91628 | 30.91587 |
| Atp1a1 | 231.7855 | 232.602 | 270.6774 | 300.4893 | 321.0932 | 285.202 |
| Rac2 | 6.219717 | 5.374643 | 3.884691 | 4.060818 | 2.596158 | 3.032605 |
| Atp6v1h | 4.81324 | 5.501422 | 4.783992 | 6.850833 | 5.387804 | 6.327932 |
| Inpp5j | 4.831898 | 4.773377 | 4.518702 | 3.050877 | 3.403269 | 3.533782 |
| Daam1 | 14.9895 | 16.06371 | 15.80355 | 10.30808 | 10.47098 | 11.82148 |
| Tbxa2r | 3.433216 | 3.449467 | 2.543592 | 2.309101 | 1.603078 | 1.565532 |
| Eef2k | 10.70391 | 9.764975 | 10.51754 | 15.76819 | 17.40781 | 15.92356 |
| Gadd45a | 5.687833 | 7.361043 | 4.647207 | 2.985017 | 3.505474 | 3.111523 |
| Card11 | 0.480438 | 0.365334 | 0.341896 | 0.22069 | 0.151402 | 0.125399 |
| Dchs1 | 3.905976 | 3.430614 | 3.270508 | 2.329476 | 2.595858 | 2.045993 |
| Il22ra1 | 0.221307 | 0.217587 | 0.234249 | 0.56842 | 0.840779 | 0.545732 |
| Map4k1 | 1.088966 | 1.479521 | 0.955561 | 0.5101 | 0.757781 | 0.31055 |
| Serpine1 | 17.83794 | 15.7018 | 20.94115 | 32.39322 | 34.73745 | 32.565 |
| Lpar1 | 3.313187 | 3.080684 | 3.415914 | 2.366346 | 2.216879 | 2.220799 |
| Itga9 | 6.150339 | 5.72896 | 5.738779 | 2.96243 | 3.585016 | 2.946788 |
| Stat2 | 11.43174 | 8.162962 | 8.31822 | 6.67075 | 4.912351 | 5.986585 |
| Pla2g5 | 13.99683 | 15.15348 | 13.7294 | 10.32821 | 9.636426 | 9.012333 |
| Pla2g2d | 3.904555 | 2.973237 | 2.885043 | 2.149404 | 1.480025 | 1.425977 |
| Pik3r1 | 9.647937 | 10.14173 | 12.44614 | 16.42368 | 19.0572 | 23.08507 |
| Acacb | 79.09119 | 75.43173 | 74.37108 | 90.01347 | 100.1053 | 91.27635 |
| Rassf4 | 5.627389 | 5.232773 | 4.357618 | 6.898361 | 6.849298 | 7.11756 |
| Itga1 | 5.199319 | 6.161941 | 5.45371 | 4.244771 | 3.342197 | 4.049036 |
| Reln | 0.851878 | 0.988299 | 1.114514 | 1.949776 | 2.042095 | 1.614147 |
| Fgf11 | 2.674148 | 2.052341 | 2.585275 | 1.665216 | 1.313805 | 1.685272 |
| Mmp3 | 2.803969 | 3.773485 | 4.102496 | 8.765143 | 8.10444 | 8.880673 |
| S1pr2 | 3.185331 | 2.245389 | 1.976463 | 1.673484 | 1.29494 | 1.516962 |
| Mylk4 | 25.045 | 25.99279 | 25.46899 | 16.54519 | 15.76708 | 18.61755 |
| Col6a3 | 8.96478 | 8.541324 | 8.551668 | 3.589962 | 3.662564 | 3.739997 |
| Foxo3 | 23.08022 | 20.42395 | 25.09394 | 27.91978 | 34.87245 | 33.87644 |
| Agtr1a | 20.7139 | 21.96809 | 22.65325 | 28.50882 | 26.84808 | 30.8239 |
| Dtx3l | 6.80962 | 6.300748 | 6.80742 | 4.130576 | 3.575338 | 4.327635 |
| F2rl3 | 1.413369 | 0.840095 | 0.687363 | 0.532011 | 0.280354 | 0.245105 |
| Rasal3 | 0.834129 | 0.619129 | 0.3735 | 0.189695 | 0.136042 | 0.275044 |
| Adam17 | 9.668567 | 9.315163 | 9.455974 | 5.583011 | 5.265267 | 6.056595 |
| Prkcb | 2.994628 | 3.023341 | 2.75256 | 1.694611 | 1.396391 | 1.796845 |
| Myh7 | 45.87055 | 21.68494 | 35.54691 | 191.277 | 296.6938 | 196.4536 |
| Irs1 | 3.127086 | 3.603514 | 3.254043 | 1.927379 | 2.55185 | 2.137136 |
| Ticam2 | 0.285841 | 0.46519 | 0.50006 | 0.18291 | 0.013986 | 0.036682 |
| Ptafr | 3.604564 | 3.259914 | 2.298086 | 1.654473 | 1.178772 | 1.494714 |
| Sipa1 | 21.22633 | 17.47865 | 14.53815 | 12.11988 | 12.33596 | 10.78497 |
| Camk2b | 8.326384 | 7.196339 | 6.41905 | 5.017103 | 3.705056 | 4.411446 |
| Fcer1g | 28.0082 | 28.77399 | 20.70567 | 18.28379 | 12.93734 | 16.21205 |
| Ptk2b | 5.264386 | 4.36853 | 4.508505 | 7.22733 | 6.920142 | 5.851481 |
| Nrg2 | 0.791229 | 0.725454 | 1.069485 | 1.686302 | 1.984757 | 1.859158 |
| Blnk | 1.622748 | 1.388598 | 1.179799 | 0.660801 | 0.591881 | 0.814059 |
| Taok3 | 4.177613 | 3.912144 | 3.929279 | 2.851936 | 2.32977 | 2.613316 |
| Nrg1 | 0.58055 | 0.733466 | 0.79028 | 1.641275 | 1.039518 | 1.246985 |
| Aox1 | 7.126677 | 8.731309 | 8.871666 | 9.528047 | 10.14023 | 11.04603 |
| Gng8 | 2.131555 | 1.842799 | 2.461518 | 0.940682 | 0.827158 | 0.848927 |
| Nck2 | 12.33013 | 11.74894 | 10.95121 | 8.09263 | 9.055324 | 6.667869 |
| Col4a4 | 1.922299 | 2.276072 | 2.351657 | 1.556754 | 1.262703 | 1.40116 |
| Afdn | 26.73479 | 27.31977 | 26.00838 | 18.35855 | 18.43309 | 18.13966 |
| Rasgrp3 | 10.16055 | 12.50355 | 11.2764 | 8.491927 | 7.502174 | 7.219673 |
| Bnip3 | 34.58607 | 37.69662 | 36.49784 | 54.6698 | 65.99382 | 65.63156 |
| Gm13033 | 0.442958 | 0.361837 | 0.474445 | 0.119097 | 0.123847 | 0.054138 |
| 4933431K23Rik | 0.760875 | 0.96797 | 0.959562 | 1.480486 | 1.660962 | 1.311924 |
| Gm17484 | 0.244848 | 0.152042 | 0.057593 | 0.715627 | 0.324166 | 0.381986 |
| A930015D03Rik | 16.2551 | 17.0536 | 11.58554 | 9.835147 | 7.982834 | 10.99668 |
| 1700101I11Rik | 0.555002 | 0.299241 | 0.375848 | 1.024335 | 0.928541 | 0.582042 |

**Table SⅥ. Differentially expressed genes of SPM/DCM that involved in signal transduction pathway.**

| symbol | DCM-1 | DCM-2 | DCM-3 | SPM-1 | SPM-2 | SPM-3 |
| --- | --- | --- | --- | --- | --- | --- |
| 1300017J02Rik | 0.071352 | 0.01325 | 0.09267 | 0.980541 | 0.584965 | 0.205822 |
| Adra1d | 1.710291 | 1.344224 | 1.431352 | 0.881741 | 0.909718 | 0.903518 |
| Angpt1 | 3.510606 | 3.034516 | 4.956722 | 6.520546 | 6.650676 | 6.996182 |
| Apbb1ip | 1.438335 | 1.160463 | 1.324563 | 2.849416 | 2.281696 | 1.877786 |
| Apc2 | 1.092388 | 1.537686 | 1.353711 | 0.968106 | 0.945076 | 1.039149 |
| Bambi-ps1 | 7.31496 | 6.578504 | 8.119613 | 6.313441 | 3.767086 | 4.1864 |
| Bmpr1a | 9.287109 | 8.68169 | 9.996896 | 13.03752 | 12.90471 | 13.31551 |
| Bmpr2 | 5.843875 | 4.772376 | 6.525809 | 6.838231 | 7.614488 | 8.185442 |
| Bnip3 | 54.6698 | 65.99382 | 65.63156 | 53.89234 | 45.57679 | 39.13811 |
| Cacna1b | 0.02586 | 0.016807 | 0.04702 | 0.006142 | 0 | 0.006527 |
| Cacna1d | 0.359747 | 0.355798 | 0.388089 | 0.575842 | 0.507348 | 0.57244 |
| Cacnb1 | 3.269461 | 3.623448 | 3.159449 | 1.945819 | 1.951568 | 2.030299 |
| Cacybp | 10.38044 | 8.563844 | 12.16486 | 12.86945 | 14.07315 | 14.11492 |
| Ccl12 | 1.172117 | 0.731322 | 0.852498 | 4.510125 | 2.749112 | 2.425925 |
| Cebpb | 1.238765 | 2.393573 | 1.331674 | 0.815106 | 0.710068 | 0.908424 |
| Chrm2 | 13.06316 | 11.08763 | 16.3425 | 20.60793 | 19.60639 | 19.30157 |
| Cish | 1.586632 | 1.825503 | 1.656899 | 3.934103 | 2.888897 | 1.718229 |
| Col4a3 | 0.235465 | 0.221902 | 0.291003 | 0.101369 | 0.135863 | 0.170866 |
| Creb5 | 0.620125 | 0.673797 | 0.939638 | 1.04238 | 0.98392 | 1.099658 |
| Csf2rb | 1.084155 | 0.621139 | 0.759093 | 2.306607 | 1.551273 | 1.160713 |
| Csf2rb2 | 0.663952 | 0.42807 | 0.56741 | 1.457174 | 0.95423 | 0.663628 |
| Csf3r | 1.808178 | 1.11878 | 2.663108 | 3.59905 | 3.473966 | 2.829603 |
| Cybb | 3.957759 | 2.64458 | 3.522307 | 7.154213 | 6.048024 | 5.095026 |
| Daam2 | 3.156973 | 3.654162 | 3.523783 | 2.443852 | 2.697001 | 2.784989 |
| Dll4 | 11.28164 | 11.70369 | 11.42341 | 8.690045 | 8.676119 | 8.691766 |
| Dnm1l | 11.90927 | 9.877415 | 11.36976 | 14.64866 | 15.10313 | 15.17383 |
| Edn1 | 2.295245 | 1.331099 | 2.059955 | 4.431251 | 3.098422 | 2.614386 |
| Epor | 3.03097 | 2.564612 | 2.924563 | 1.901676 | 1.890668 | 1.659955 |
| Erbb4 | 0.711158 | 0.459484 | 0.696462 | 1.095464 | 1.075097 | 0.979317 |
| Evc | 4.325217 | 3.987736 | 4.061907 | 2.7861 | 2.839615 | 3.614488 |
| Ffar2 | 0 | 0.014223 | 0.049738 | 0.220907 | 0.136506 | 0.124278 |
| Fgf12 | 0.334721 | 0.711965 | 0.753857 | 1.214232 | 1.571642 | 2.250349 |
| Fgf18 | 3.292029 | 3.194316 | 3.425006 | 5.09354 | 5.032552 | 4.300861 |
| Fnip1 | 4.705355 | 3.374693 | 4.405008 | 6.362511 | 6.49292 | 6.283625 |
| Fzd8 | 3.46427 | 3.653949 | 3.553747 | 2.237279 | 2.50271 | 3.18588 |
| Gadd45b | 12.5101 | 10.83243 | 13.45921 | 28.61967 | 20.15724 | 12.15022 |
| Gm13033 | 0.119097 | 0.123847 | 0.054138 | 0.396033 | 0.564609 | 0.571145 |
| Gm4609 | 0.55279 | 0.587904 | 0.542542 | 0.238726 | 0.031347 | 0.126841 |
| Gm5881 | 0.548819 | 0.535041 | 0.467771 | 0 | 0 | 0 |
| Gm7336 | 363.4326 | 466.5676 | 337.7467 | 305.4361 | 297.1464 | 286.8918 |
| Gnas | 6.187423 | 10.29808 | 5.596261 | 4.24985 | 4.122223 | 3.984794 |
| Gnb3 | 11.89296 | 15.17553 | 13.29852 | 9.459299 | 8.473571 | 8.829854 |
| Gng2 | 1.77674 | 2.82274 | 2.722101 | 3.993501 | 4.318268 | 4.094207 |
| Gng8 | 0.940682 | 0.827158 | 0.848927 | 1.544309 | 1.760344 | 1.641059 |
| Gys1 | 34.98641 | 38.60822 | 34.44638 | 22.57574 | 26.30315 | 29.8818 |
| Hcn2 | 8.597241 | 8.337876 | 8.570158 | 5.156542 | 5.810125 | 6.513858 |
| Hnf4a | 0 | 0 | 0 | 0.349593 | 0.180021 | 0 |
| Homer1 | 2.679443 | 2.572077 | 2.84467 | 4.815057 | 4.453379 | 4.310278 |
| Hrh1 | 0.332854 | 0.155759 | 0.158871 | 0.063248 | 0.074746 | 0.092414 |
| Hsp90aa1 | 16.25268 | 14.16394 | 16.87417 | 20.93305 | 22.94725 | 23.15481 |
| Hsp90b1 | 18.25377 | 14.67723 | 21.3835 | 30.58718 | 31.1761 | 30.99465 |
| Hspa2 | 4.515128 | 4.923253 | 4.440179 | 3.1605 | 3.058938 | 3.169823 |
| Id2 | 9.295952 | 5.900442 | 6.015925 | 12.027 | 10.24137 | 8.617145 |
| Ighv1-9 | 0 | 0 | 0 | 0.085186 | 1.073848 | 1.267327 |
| Il13ra1 | 8.030617 | 8.234567 | 9.396011 | 11.76042 | 11.96493 | 12.36024 |
| Il17d | 3.91292 | 4.623863 | 3.054339 | 2.417384 | 1.898411 | 2.917993 |
| Il1r1 | 4.015178 | 3.650045 | 3.604154 | 5.385063 | 5.030792 | 4.826938 |
| Il7 | 0.243582 | 0.127996 | 0.188469 | 0.455462 | 0.381473 | 0.392429 |
| Inhbb | 3.189293 | 2.615057 | 2.978873 | 1.489752 | 1.712605 | 2.19541 |
| Irf9 | 7.405403 | 6.276349 | 6.921893 | 9.049738 | 8.751629 | 8.959708 |
| Itga4 | 0.38151 | 0.080418 | 0.290016 | 0.450022 | 0.598164 | 0.562798 |
| Itgal | 1.176408 | 0.582838 | 1.068429 | 1.482999 | 1.888694 | 1.940983 |
| Itgb2 | 6.247643 | 4.418652 | 5.356315 | 8.184873 | 7.614561 | 7.135711 |
| Itpka | 0.595497 | 0.558046 | 0.472145 | 0.049341 | 0.120941 | 0.314591 |
| Kitl | 8.572151 | 5.493075 | 7.112466 | 9.026253 | 9.103803 | 9.771764 |
| Klf2 | 84.69147 | 106.0826 | 85.09234 | 54.08632 | 65.20097 | 74.64326 |
| Krit1 | 3.418886 | 2.71452 | 3.557275 | 4.071005 | 4.242778 | 4.354512 |
| Lepr | 0.795018 | 0.513753 | 0.753762 | 1.737269 | 1.144863 | 0.819856 |
| Llgl2 | 8.545705 | 10.16593 | 8.539231 | 5.902277 | 6.104614 | 6.54475 |
| Lpar1 | 2.366346 | 2.216879 | 2.220799 | 2.776531 | 3.235866 | 3.21952 |
| Map2k3 | 43.96251 | 43.70009 | 41.17364 | 28.05038 | 32.62474 | 34.82386 |
| Map3k12 | 1.607254 | 1.533767 | 1.70513 | 1.257146 | 1.211574 | 1.121425 |
| Map3k2 | 1.121704 | 0.937323 | 1.253314 | 1.494463 | 1.652233 | 1.939017 |
| Map3k8 | 2.724494 | 1.566906 | 1.704022 | 3.619713 | 3.10555 | 2.572569 |
| Mapk8 | 3.175565 | 2.717747 | 3.429857 | 3.807685 | 4.360084 | 4.228398 |
| Mlycd | 112.2453 | 115.966 | 105.317 | 67.99729 | 76.11024 | 81.04299 |
| Mob1b | 2.010347 | 1.580311 | 2.150604 | 2.767635 | 3.187594 | 3.135913 |
| Mpp5 | 2.425652 | 1.896592 | 2.447971 | 3.645997 | 3.250109 | 3.154136 |
| Myh7 | 191.277 | 296.6938 | 196.4536 | 758.6269 | 528.3445 | 318.6041 |
| Mylk4 | 16.54519 | 15.76708 | 18.61755 | 30.53915 | 26.53308 | 22.46003 |
| Nod2 | 0.93367 | 1.393952 | 1.127745 | 0.703317 | 0.785418 | 0.875309 |
| Notch4 | 10.76452 | 12.13533 | 11.89452 | 9.086727 | 8.797297 | 8.455633 |
| Notum | 0.011382 | 0.014795 | 0 | 0.121656 | 0.227198 | 0.114914 |
| Nr4a1 | 87.17795 | 53.48049 | 58.32225 | 25.93013 | 36.77426 | 47.14406 |
| Pde1c | 6.941932 | 7.067259 | 7.507133 | 10.19535 | 9.355656 | 8.778908 |
| Pfkfb3 | 8.637083 | 6.394006 | 7.807057 | 12.36251 | 10.37625 | 9.44409 |
| Pi4k2a | 15.72315 | 14.97911 | 13.05028 | 11.21168 | 10.88649 | 11.13049 |
| Pik3ap1 | 2.281822 | 1.339103 | 1.761578 | 4.939578 | 3.4473 | 2.746026 |
| Pik3c2a | 1.292657 | 1.053731 | 1.422773 | 1.865991 | 1.987534 | 1.963144 |
| Pla2g12a | 21.23053 | 22.49959 | 20.46444 | 15.12012 | 14.44527 | 15.35587 |
| Pla2g2d | 2.149404 | 1.480025 | 1.425977 | 0.469135 | 0.76822 | 0.835764 |
| Pla2g5 | 10.32821 | 9.636426 | 9.012333 | 5.029129 | 6.833645 | 7.551287 |
| Ppargc1a | 10.97212 | 10.35366 | 11.79181 | 15.67184 | 14.70403 | 13.19317 |
| Ppp1cb | 30.764 | 27.08377 | 36.64559 | 43.97805 | 43.86978 | 44.31904 |
| Ppp1r12a | 9.154395 | 6.653864 | 8.127245 | 10.83641 | 11.47032 | 10.49297 |
| Prkaa2 | 11.03886 | 10.87467 | 13.09422 | 15.19224 | 15.37986 | 15.51405 |
| Prkag3 | 0.595644 | 0.820478 | 0.84866 | 0.285068 | 0.388189 | 0.516098 |
| Prkcb | 1.694611 | 1.396391 | 1.796845 | 3.072703 | 2.548293 | 2.186352 |
| Prkg1 | 2.502269 | 1.990023 | 2.141877 | 3.315482 | 3.643375 | 3.515078 |
| Ptgfr | 0.497916 | 0.627459 | 0.704069 | 0.744803 | 1.019499 | 1.06488 |
| Ptpn2 | 3.577844 | 3.030929 | 3.497376 | 5.515945 | 4.93469 | 4.0149 |
| Ptpn6 | 3.872556 | 2.694137 | 3.644679 | 5.505933 | 4.980972 | 4.4742 |
| Rac2 | 4.060818 | 2.596158 | 3.032605 | 6.259608 | 4.786985 | 3.827905 |
| Rap1a | 13.53516 | 10.61883 | 15.22723 | 17.4409 | 17.77337 | 17.0815 |
| Rapgef6 | 2.91155 | 2.297941 | 3.242387 | 4.147871 | 3.82797 | 3.914967 |
| Rictor | 2.006163 | 1.760746 | 2.083217 | 2.87702 | 2.546671 | 2.47379 |
| Rps6ka3 | 4.928366 | 4.16086 | 4.972732 | 6.170787 | 6.222176 | 6.241577 |
| Rras | 64.09399 | 68.31488 | 53.51445 | 48.6521 | 47.06775 | 46.65465 |
| Scd4 | 20.69912 | 25.87645 | 20.63247 | 33.06596 | 30.49798 | 28.58288 |
| Scrib | 8.66066 | 9.498276 | 7.879917 | 5.714143 | 6.165326 | 6.724744 |
| Serpine1 | 32.39322 | 34.73745 | 32.565 | 17.9939 | 22.24054 | 26.59241 |
| Sfrp1 | 8.995102 | 7.290892 | 8.010305 | 11.85213 | 10.79092 | 10.41439 |
| Sfrp2 | 4.235982 | 4.721922 | 2.799773 | 5.344166 | 5.629662 | 6.218319 |
| Sgk3 | 2.751556 | 2.416179 | 2.801384 | 3.916572 | 4.72938 | 4.910252 |
| Slc8a1 | 11.91356 | 12.49229 | 14.51363 | 22.73465 | 19.96982 | 18.21172 |
| Socs2 | 5.780874 | 5.439016 | 6.839354 | 10.22762 | 8.026523 | 7.198145 |
| Sphk2 | 8.408914 | 9.675953 | 8.241667 | 5.789632 | 6.258996 | 6.671761 |
| Src | 3.093956 | 3.066555 | 2.080338 | 1.622861 | 1.881709 | 2.07432 |
| Stat1 | 2.732282 | 2.522324 | 3.110601 | 3.389996 | 3.84165 | 4.50914 |
| Stat5a | 10.23494 | 10.3108 | 8.934915 | 6.753666 | 7.332719 | 8.188271 |
| Stk3 | 1.482803 | 0.784884 | 1.372402 | 2.24208 | 1.94525 | 1.678392 |
| Tfrc | 2.105812 | 1.780224 | 3.311733 | 6.169644 | 5.941577 | 5.763451 |
| Tgfbr1 | 3.044343 | 3.115743 | 3.062018 | 4.498575 | 4.42011 | 4.112476 |
| Tlr2 | 2.313885 | 1.531009 | 1.765702 | 3.531706 | 3.095152 | 2.530089 |
| Tlr4 | 4.816805 | 4.101872 | 5.249388 | 7.09925 | 5.80127 | 5.806085 |
| Tnfsf10 | 5.264342 | 3.722962 | 4.882315 | 7.132274 | 7.624474 | 8.158199 |
| Traf6 | 3.330279 | 2.960202 | 3.84956 | 4.163477 | 4.450056 | 4.553081 |
| Vav1 | 1.665442 | 1.336459 | 1.516058 | 2.633824 | 2.459377 | 1.747929 |
| Wif1 | 7.353373 | 7.047505 | 5.176097 | 4.470731 | 4.176142 | 4.459953 |
| Wnt11 | 6.062633 | 6.325324 | 6.002627 | 4.366801 | 4.2141 | 4.198438 |
| Xiap | 5.367719 | 4.630772 | 5.773472 | 6.540998 | 7.119431 | 7.387828 |

**Table SⅦ. Venn analysis identified 22 genes in immune system related pathways and 23 genes involved in signal transduction that differentially expressed in DCM but reversal regulated by SPM.**

| Gene_ID | CK-1 | CK-2 | CK-3 | DCM-1 | DCM-2 | DCM-3 | SPM-1 | SPM-2 | SPM-3 | symbol | ncbi_descritption |
| --- | --- | --- | --- | --- | --- | --- | --- | --- | --- | --- | --- |
| ENSMUSG00000001739 | 6.822 | 5.847 | 8.648 | 11.212 | 11.305 | 9.282 | 7.845 | 7.943 | 8.069 | Cldn15 | claudin 15 |
| ENSMUSG00000003283 | 3.373 | 3.178 | 2.285 | 1.468 | 1.017 | 2.202 | 3.173 | 2.898 | 2.734 | Hck | hemopoietic cell kinase |
| ENSMUSG00000015340 | 5.891 | 6.346 | 4.960 | 3.958 | 2.645 | 3.522 | 7.154 | 6.048 | 5.095 | Cybb | cytochrome b-245, beta polypeptide |
| ENSMUSG00000015355 | 5.104 | 5.333 | 4.003 | 3.227 | 2.373 | 2.099 | 6.076 | 5.058 | 3.885 | Cd48 | CD48 antigen |
| ENSMUSG00000020143 | 2.228 | 2.015 | 1.878 | 1.092 | 0.945 | 1.237 | 1.750 | 1.676 | 1.458 | Dock2 | dedicator of cyto-kinesis 2 |
| ENSMUSG00000023439 | 12.410 | 9.515 | 7.299 | 11.893 | 15.176 | 13.299 | 9.459 | 8.474 | 8.830 | Gnb3 | guanine nucleotide binding protein (G protein), beta 3 |
| ENSMUSG00000025017 | 3.632 | 3.071 | 2.480 | 2.282 | 1.339 | 1.762 | 4.940 | 3.447 | 2.746 | Pik3ap1 | phosphoinositide-3-kinase adaptor protein 1 |
| ENSMUSG00000026104 | 6.239 | 3.747 | 4.108 | 2.732 | 2.522 | 3.111 | 3.390 | 3.842 | 4.509 | Stat1 | signal transducer and activator of transcription 1 |
| ENSMUSG00000026399 | 3.395 | 4.742 | 4.108 | 3.198 | 2.120 | 2.548 | 3.875 | 4.040 | 3.854 | Cd55 | CD55 molecule, decay accelerating factor for complement |
| ENSMUSG00000026896 | 2.372 | 2.899 | 2.426 | 1.795 | 1.932 | 1.695 | 2.361 | 2.303 | 2.643 | Ifih1 | interferon induced with helicase C domain 1 |
| ENSMUSG00000027514 | 2.625 | 1.433 | 1.176 | 0.886 | 0.650 | 0.895 | 1.286 | 1.625 | 1.720 | Zbp1 | Z-DNA binding protein 1 |
| ENSMUSG00000027523 | 3.999 | 4.957 | 4.820 | 6.187 | 10.298 | 5.596 | 4.250 | 4.122 | 3.985 | Gnas | GNAS (guanine nucleotide binding protein, alpha stimulating) complex locus |
| ENSMUSG00000027995 | 3.451 | 3.270 | 2.540 | 2.314 | 1.531 | 1.766 | 3.532 | 3.095 | 2.530 | Tlr2 | toll-like receptor 2 |
| ENSMUSG00000030579 | 38.597 | 41.001 | 32.761 | 23.785 | 16.665 | 21.854 | 37.513 | 27.254 | 24.269 | Tyrobp | TYRO protein tyrosine kinase binding protein |
| ENSMUSG00000033220 | 6.220 | 5.375 | 3.885 | 4.061 | 2.596 | 3.033 | 6.260 | 4.787 | 3.828 | Rac2 | RAS-related C3 botulinum substrate 2 |
| ENSMUSG00000037411 | 17.838 | 15.702 | 20.941 | 32.393 | 34.737 | 32.565 | 17.994 | 22.241 | 26.592 | Serpine1 | serine (or cysteine) peptidase inhibitor, clade E, member 1 |
| ENSMUSG00000038642 | 32.086 | 29.333 | 26.376 | 19.603 | 14.926 | 14.042 | 35.969 | 28.999 | 20.096 | Ctss | cathepsin S |
| ENSMUSG00000044951 | 25.045 | 25.993 | 25.469 | 16.545 | 15.767 | 18.618 | 30.539 | 26.533 | 22.460 | Mylk4 | myosin light chain kinase family, member 4 |
| ENSMUSG00000052336 | 4.704 | 3.045 | 2.009 | 0.930 | 0.772 | 0.815 | 1.524 | 1.489 | 1.414 | Cx3cr1 | chemokine (C-X3-C motif) receptor 1 |
| ENSMUSG00000052889 | 2.995 | 3.023 | 2.753 | 1.695 | 1.396 | 1.797 | 3.073 | 2.548 | 2.186 | Prkcb | protein kinase C, beta |
| ENSMUSG00000055413 | 16.635 | 11.223 | 10.209 | 8.625 | 6.568 | 7.301 | 12.338 | 12.010 | 9.867 | H2-Q5 | histocompatibility 2, Q region locus 5 |
| ENSMUSG00000063594 | 2.132 | 1.843 | 2.462 | 0.941 | 0.827 | 0.849 | 1.544 | 1.760 | 1.641 | Gng8 | guanine nucleotide binding protein (G protein), gamma 8 |
| ENSMUSG00000002325 | 14.107 | 11.954 | 11.424 | 7.405 | 6.276 | 6.922 | 9.050 | 8.752 | 8.960 | Irf9 | interferon regulatory factor 9 |
| ENSMUSG00000015340 | 5.891 | 6.346 | 4.960 | 3.958 | 2.645 | 3.522 | 7.154 | 6.048 | 5.095 | Cybb | cytochrome b-245, beta polypeptide |
| ENSMUSG00000015957 | 3.867 | 3.930 | 3.846 | 6.063 | 6.325 | 6.003 | 4.367 | 4.214 | 4.198 | Wnt11 | wingless-type MMTV integration site family, member 11 |
| ENSMUSG00000015968 | 0.648 | 0.816 | 0.734 | 0.360 | 0.356 | 0.388 | 0.576 | 0.507 | 0.572 | Cacna1d | calcium channel, voltage-dependent, L type, alpha 1D subunit |
| ENSMUSG00000017950 | 0.054 | 0.040 | 0.046 | 0.000 | 0.000 | 0.000 | 0.350 | 0.180 | 0.000 | Hnf4a | hepatic nuclear factor 4, alpha |
| ENSMUSG00000020782 | 7.972 | 6.133 | 6.812 | 8.546 | 10.166 | 8.539 | 5.902 | 6.105 | 6.545 | Llgl2 | LLGL2 scribble cell polarity complex component |
| ENSMUSG00000022523 | 1.318 | 0.974 | 1.123 | 0.335 | 0.712 | 0.754 | 1.214 | 1.572 | 2.250 | Fgf12 | fibroblast growth factor 12 |
| ENSMUSG00000023439 | 12.410 | 9.515 | 7.299 | 11.893 | 15.176 | 13.299 | 9.459 | 8.474 | 8.830 | Gnb3 | guanine nucleotide binding protein (G protein), beta 3 |
| ENSMUSG00000025017 | 3.632 | 3.071 | 2.480 | 2.282 | 1.339 | 1.762 | 4.940 | 3.447 | 2.746 | Pik3ap1 | phosphoinositide-3-kinase adaptor protein 1 |
| ENSMUSG00000025178 | 11.999 | 11.273 | 10.630 | 15.723 | 14.979 | 13.050 | 11.212 | 10.886 | 11.130 | Pi4k2a | phosphatidylinositol 4-kinase type 2 alpha |
| ENSMUSG00000026104 | 6.239 | 3.747 | 4.108 | 2.732 | 2.522 | 3.111 | 3.390 | 3.842 | 4.509 | Stat1 | signal transducer and activator of transcription 1 |
| ENSMUSG00000027523 | 3.999 | 4.957 | 4.820 | 6.187 | 10.298 | 5.596 | 4.250 | 4.122 | 3.985 | Gnas | GNAS (guanine nucleotide binding protein, alpha stimulating) complex locus |
| ENSMUSG00000027995 | 3.451 | 3.270 | 2.540 | 2.314 | 1.531 | 1.766 | 3.532 | 3.095 | 2.530 | Tlr2 | toll-like receptor 2 |
| ENSMUSG00000027996 | 6.027 | 5.978 | 5.575 | 4.236 | 4.722 | 2.800 | 5.344 | 5.630 | 6.218 | Sfrp2 | secreted frizzled-related protein 2 |
| ENSMUSG00000027999 | 17.459 | 17.552 | 16.292 | 21.231 | 22.500 | 20.464 | 15.120 | 14.445 | 15.356 | Pla2g12a | phospholipase A2, group XIIA |
| ENSMUSG00000033220 | 6.220 | 5.375 | 3.885 | 4.061 | 2.596 | 3.033 | 6.260 | 4.787 | 3.828 | Rac2 | RAS-related C3 botulinum substrate 2 |
| ENSMUSG00000037411 | 17.838 | 15.702 | 20.941 | 32.393 | 34.737 | 32.565 | 17.994 | 22.241 | 26.592 | Serpine1 | serine (or cysteine) peptidase inhibitor, clade E, member 1 |
| ENSMUSG00000038668 | 3.313 | 3.081 | 3.416 | 2.366 | 2.217 | 2.221 | 2.777 | 3.236 | 3.220 | Lpar1 | lysophosphatidic acid receptor 1 |
| ENSMUSG00000044951 | 25.045 | 25.993 | 25.469 | 16.545 | 15.767 | 18.618 | 30.539 | 26.533 | 22.460 | Mylk4 | myosin light chain kinase family, member 4 |
| ENSMUSG00000052889 | 2.995 | 3.023 | 2.753 | 1.695 | 1.396 | 1.797 | 3.073 | 2.548 | 2.186 | Prkcb | protein kinase C, beta |
| ENSMUSG00000063594 | 2.132 | 1.843 | 2.462 | 0.941 | 0.827 | 0.849 | 1.544 | 1.760 | 1.641 | Gng8 | guanine nucleotide binding protein (G protein), gamma 8 |
| ENSMUSG00000078566 | 34.586 | 37.697 | 36.498 | 54.670 | 65.994 | 65.632 | 53.892 | 45.577 | 39.138 | Bnip3 | BCL2/adenovirus E1B interacting protein 3 |
| ENSMUSG00000083816 | 0.443 | 0.362 | 0.474 | 0.119 | 0.124 | 0.054 | 0.396 | 0.565 | 0.571 | Gm13033 | prostaglandin-endoperoxide synthase 2 pseudogene |

**Table SVIII. Immune system and signal transduction related genes of DCM mice heart before/after SPM treatment that belonging to profile 0, 1,6, and 7.**

| Gene_ID | CK-1 | CK-2 | CK-3 | DCM-1 | DCM-2 | DCM-3 | SPM-1 | SPM-2 | SPM-3 | symbol | ncbi_descritption |
| --- | --- | --- | --- | --- | --- | --- | --- | --- | --- | --- | --- |
| ENSMUSG00000004709 | 0.276 | 0.156 | 0.178 | 0.134 | 0.113 | 0.084 | 0.286 | 0.326 | 0.330 | Cd244 | CD244 natural killer cell receptor 2B4 |
| ENSMUSG00000015437 | 0.629 | 0.758 | 0.140 | 0.351 | 0.023 | 0.060 | 0.833 | 0.962 | 0.841 | Gzmb | granzyme B |
| ENSMUSG00000015947 | 3.552 | 2.102 | 2.286 | 1.138 | 0.872 | 1.282 | 2.113 | 1.808 | 1.215 | Fcgr1 | Fc receptor, IgG, high affinity I |
| ENSMUSG00000021492 | 0.068 | 0.120 | 0.068 | 0.000 | 0.000 | 0.000 | 0.488 | 0.144 | 0.016 | F12 | coagulation factor XII (Hageman factor) |
| ENSMUSG00000025017 | 3.632 | 3.071 | 2.480 | 2.282 | 1.339 | 1.762 | 4.940 | 3.447 | 2.746 | Pik3ap1 | phosphoinositide-3-kinase adaptor protein 1 |
| ENSMUSG00000026395 | 2.331 | 2.363 | 1.444 | 1.439 | 0.473 | 1.072 | 2.360 | 2.151 | 2.070 | Ptprc | protein tyrosine phosphatase, receptor type, C |
| ENSMUSG00000026579 | 0.167 | 0.123 | 0.090 | 0.132 | 0.026 | 0.108 | 0.274 | 0.220 | 0.197 | F5 | coagulation factor V |
| ENSMUSG00000026715 | 0.027 | 0.124 | 0.000 | 0.000 | 0.026 | 0.012 | 0.987 | 0.474 | 0.006 | Serpinc1 | serine (or cysteine) peptidase inhibitor, clade C (antithrombin), member 1 |
| ENSMUSG00000026874 | 0.031 | 0.103 | 0.000 | 0.000 | 0.000 | 0.000 | 0.280 | 0.179 | 0.023 | Hc | hemolytic complement |
| ENSMUSG00000027009 | 0.349 | 0.434 | 0.397 | 0.382 | 0.080 | 0.290 | 0.450 | 0.598 | 0.563 | Itga4 | integrin alpha 4 |
| ENSMUSG00000027249 | 0.021 | 0.149 | 0.000 | 0.000 | 0.000 | 0.009 | 1.737 | 0.922 | 0.000 | F2 | coagulation factor II |
| ENSMUSG00000027514 | 2.625 | 1.433 | 1.176 | 0.886 | 0.650 | 0.895 | 1.286 | 1.625 | 1.720 | Zbp1 | Z-DNA binding protein 1 |
| ENSMUSG00000028001 | 0.013 | 0.978 | 0.039 | 0.000 | 0.000 | 0.000 | 4.415 | 1.894 | 0.000 | Fga | fibrinogen alpha chain |
| ENSMUSG00000028459 | 1.877 | 1.321 | 0.608 | 0.663 | 0.469 | 0.831 | 2.454 | 1.471 | 0.763 | Cd72 | CD72 antigen |
| ENSMUSG00000030789 | 0.661 | 0.662 | 0.529 | 0.436 | 0.194 | 0.262 | 0.503 | 0.404 | 0.487 | Itgax | integrin alpha X |
| ENSMUSG00000033831 | 0.009 | 0.571 | 0.035 | 0.007 | 0.000 | 0.000 | 3.908 | 1.929 | 0.000 | Fgb | fibrinogen beta chain |
| ENSMUSG00000033860 | 0.077 | 0.727 | 0.000 | 0.000 | 0.000 | 0.000 | 4.586 | 1.948 | 0.146 | Fgg | fibrinogen gamma chain |
| ENSMUSG00000035352 | 2.367 | 1.370 | 1.432 | 1.172 | 0.731 | 0.852 | 4.510 | 2.749 | 2.426 | Ccl12 | chemokine (C-C motif) ligand 12 |
| ENSMUSG00000035373 | 2.338 | 2.534 | 1.586 | 1.603 | 0.245 | 1.429 | 5.637 | 3.412 | 1.825 | Ccl7 | chemokine (C-C motif) ligand 7 |
| ENSMUSG00000035834 | 1.128 | 1.079 | 0.744 | 0.602 | 0.975 | 0.768 | 1.674 | 1.481 | 1.665 | Polr3g | polymerase (RNA) III (DNA directed) polypeptide G |
| ENSMUSG00000036526 | 0.480 | 0.365 | 0.342 | 0.221 | 0.151 | 0.125 | 0.175 | 0.283 | 0.387 | Card11 | caspase recruitment domain family, member 11 |
| ENSMUSG00000037860 | 0.318 | 0.632 | 0.447 | 0.346 | 0.380 | 0.383 | 1.243 | 0.819 | 0.369 | Aim2 | absent in melanoma 2 |
| ENSMUSG00000038224 | 0.107 | 0.148 | 0.000 | 0.000 | 0.000 | 0.013 | 1.050 | 0.573 | 0.000 | Serpinf2 | serine (or cysteine) peptidase inhibitor, clade F, member 2 |
| ENSMUSG00000044583 | 1.322 | 1.178 | 1.313 | 0.950 | 0.764 | 1.344 | 2.632 | 2.321 | 1.908 | Tlr7 | toll-like receptor 7 |
| ENSMUSG00000044827 | 0.608 | 0.632 | 0.398 | 0.350 | 0.303 | 0.426 | 0.929 | 0.748 | 0.483 | Tlr1 | toll-like receptor 1 |
| ENSMUSG00000056130 | 0.286 | 0.465 | 0.500 | 0.183 | 0.014 | 0.037 | 0.460 | 0.242 | 0.122 | Ticam2 | toll-like receptor adaptor molecule 2 |
| ENSMUSG00000059089 | 2.396 | 1.218 | 1.278 | 1.363 | 0.573 | 0.615 | 4.523 | 4.401 | 3.415 | Fcgr4 | Fc receptor, IgG, low affinity IV |
| ENSMUSG00000059481 | 0.000 | 0.448 | 0.000 | 0.009 | 0.000 | 0.000 | 2.961 | 1.242 | 0.012 | Plg | plasminogen |
| ENSMUSG00000063594 | 2.132 | 1.843 | 2.462 | 0.941 | 0.827 | 0.849 | 1.544 | 1.760 | 1.641 | Gng8 | guanine nucleotide binding protein (G protein), gamma 8 |
| ENSMUSG00000066366 | 0.190 | 0.744 | 0.021 | 0.000 | 0.000 | 0.018 | 3.801 | 2.007 | 0.000 | Serpina1a | serine (or cysteine) peptidase inhibitor, clade A, member 1A |
| ENSMUSG00000071178 | 0.626 | 1.469 | 0.114 | 0.086 | 0.390 | 0.325 | 9.690 | 4.457 | 0.198 | Serpina1b | serine (or cysteine) preptidase inhibitor, clade A, member 1B |
| ENSMUSG00000094694 | 0.572 | 0.838 | 0.095 | 0.000 | 0.000 | 0.000 | 0.085 | 1.074 | 1.267 | Ighv1-9 | immunoglobulin heavy variable V1-9 |
| ENSMUSG00000017950 | 0.054 | 0.040 | 0.046 | 0.000 | 0.000 | 0.000 | 0.350 | 0.180 | 0.000 | Hnf4a | hepatic nuclear factor 4, alpha |
| ENSMUSG00000022523 | 1.318 | 0.974 | 1.123 | 0.335 | 0.712 | 0.754 | 1.214 | 1.572 | 2.250 | Fgf12 | fibroblast growth factor 12 |
| ENSMUSG00000025017 | 3.632 | 3.071 | 2.480 | 2.282 | 1.339 | 1.762 | 4.940 | 3.447 | 2.746 | Pik3ap1 | phosphoinositide-3-kinase adaptor protein 1 |
| ENSMUSG00000027009 | 0.349 | 0.434 | 0.397 | 0.382 | 0.080 | 0.290 | 0.450 | 0.598 | 0.563 | Itga4 | integrin alpha 4 |
| ENSMUSG00000027296 | 0.221 | 0.113 | 0.294 | 0.595 | 0.558 | 0.472 | 0.049 | 0.121 | 0.315 | Itpka | inositol 1,4,5-trisphosphate 3-kinase A |
| ENSMUSG00000035352 | 2.367 | 1.370 | 1.432 | 1.172 | 0.731 | 0.852 | 4.510 | 2.749 | 2.426 | Ccl12 | chemokine (C-C motif) ligand 12 |
| ENSMUSG00000036526 | 0.480 | 0.365 | 0.342 | 0.221 | 0.151 | 0.125 | 0.175 | 0.283 | 0.387 | Card11 | caspase recruitment domain family, member 11 |
| ENSMUSG00000042988 | 0.076 | 0.040 | 0.015 | 0.011 | 0.015 | 0.000 | 0.122 | 0.227 | 0.115 | Notum | notum palmitoleoyl-protein carboxylesterase |
| ENSMUSG00000051314 | 0.029 | 0.217 | 0.145 | 0.000 | 0.014 | 0.050 | 0.221 | 0.137 | 0.124 | Ffar2 | free fatty acid receptor 2 |
| ENSMUSG00000056130 | 0.286 | 0.465 | 0.500 | 0.183 | 0.014 | 0.037 | 0.460 | 0.242 | 0.122 | Ticam2 | toll-like receptor adaptor molecule 2 |
| ENSMUSG00000056501 | 0.734 | 1.584 | 1.245 | 1.239 | 2.394 | 1.332 | 0.815 | 0.710 | 0.908 | Cebpb | CCAAT/enhancer binding protein (C/EBP), beta |
| ENSMUSG00000063594 | 2.132 | 1.843 | 2.462 | 0.941 | 0.827 | 0.849 | 1.544 | 1.760 | 1.641 | Gng8 | guanine nucleotide binding protein (G protein), gamma 8 |
| ENSMUSG00000071713 | 1.269 | 1.543 | 0.798 | 1.084 | 0.621 | 0.759 | 2.307 | 1.551 | 1.161 | Csf2rb | colony stimulating factor 2 receptor, beta, low-affinity (granulocyte-macrophage) |
| ENSMUSG00000078592 | 0.267 | 0.294 | 0.267 | 0.553 | 0.588 | 0.543 | 0.239 | 0.031 | 0.127 | Gm4609 | glyceraldehyde-3-phosphate dehydrogenase pseudogene |
| ENSMUSG00000083816 | 0.443 | 0.362 | 0.474 | 0.119 | 0.124 | 0.054 | 0.396 | 0.565 | 0.571 | Gm13033 | prostaglandin-endoperoxide synthase 2 pseudogene |
| ENSMUSG00000094694 | 0.572 | 0.838 | 0.095 | 0.000 | 0.000 | 0.000 | 0.085 | 1.074 | 1.267 | Ighv1-9 | immunoglobulin heavy variable V1-9 |
| ENSMUSG00000101904 | 0.018 | 0.123 | 0.000 | 0.013 | 0.034 | 0.000 | 0.219 | 0.296 | 0.316 | Gm29427 | predicted gene 29427 |

**Table SIX. Venn analysis identified 14 TFs or TFs co-factors that differentially expressed in DCM but reversal regulated by SPM.**

| Gene_ID | CK-1 | CK-2 | CK-3 | DCM-1 | DCM-2 | DCM-3 | SPM-1 | SPM-2 | SPM-3 | symbol | ncbi_descritption | TF_Family | TF_type |
| --- | --- | --- | --- | --- | --- | --- | --- | --- | --- | --- | --- | --- | --- |
| ENSMUSG00000017950 | 0.054 | 0.040 | 0.046 | 0.000 | 0.000 | 0.000 | 0.350 | 0.180 | 0.000 | Hnf4a | hepatic nuclear factor 4, alpha | RXR-like | TF |
| ENSMUSG00000026104 | 6.239 | 3.747 | 4.108 | 2.732 | 2.522 | 3.111 | 3.390 | 3.842 | 4.509 | Stat1 | signal transducer and activator of transcription 1 | STAT | TF |
| ENSMUSG00000002111 | 10.243 | 6.784 | 5.786 | 4.642 | 4.312 | 4.009 | 8.379 | 6.594 | 5.296 | Spi1 | spleen focus forming virus (SFFV) proviral integration oncogene | ETS | TF |
| ENSMUSG00000002325 | 14.107 | 11.954 | 11.424 | 7.405 | 6.276 | 6.922 | 9.050 | 8.752 | 8.960 | Irf9 | interferon regulatory factor 9 | IRF | TF |
| ENSMUSG00000008496 | 0.278 | 0.448 | 0.165 | 0.216 | 0.055 | 0.065 | 0.282 | 0.310 | 0.233 | Pou2f2 | POU domain, class 2, transcription factor 2 | Pou | TF |
| ENSMUSG00000018654 | 1.710 | 1.476 | 1.062 | 1.073 | 0.494 | 0.582 | 1.222 | 1.107 | 1.194 | Ikzf1 | IKAROS family zinc finger 1 | zf-C2H2 | TF |
| ENSMUSG00000024561 | 15.587 | 15.085 | 15.906 | 20.366 | 21.873 | 22.152 | 15.766 | 15.224 | 15.603 | Mbd1 | methyl-CpG binding domain protein 1 | MBD | TF |
| ENSMUSG00000027547 | 0.349 | 0.354 | 0.395 | 0.561 | 0.587 | 0.682 | 0.306 | 0.347 | 0.407 | Sall4 | spalt like transcription factor 4 | zf-C2H2 | TF |
| ENSMUSG00000033249 | 3.442 | 3.823 | 2.826 | 5.024 | 5.665 | 4.689 | 3.869 | 3.829 | 3.380 | Hsf4 | heat shock transcription factor 4 | HSF | TF |
| ENSMUSG00000003283 | 3.373 | 3.178 | 2.285 | 1.468 | 1.017 | 2.202 | 3.173 | 2.898 | 2.734 | Hck | hemopoietic cell kinase | Others | TF_cofactors |
| ENSMUSG00000004698 | 2.915 | 3.690 | 3.966 | 2.286 | 2.127 | 2.526 | 3.186 | 3.200 | 3.369 | Hdac9 | histone deacetylase 9 | Histone deacetylase | TF_cofactors |
| ENSMUSG00000022831 | 13.752 | 11.923 | 10.166 | 9.317 | 6.964 | 7.210 | 12.800 | 10.423 | 9.882 | Hcls1 | hematopoietic cell specific Lyn substrate 1 | Others | TF_cofactors |
| ENSMUSG00000034245 | 12.480 | 10.313 | 11.820 | 13.609 | 15.753 | 14.506 | 11.721 | 10.798 | 10.432 | Hdac11 | histone deacetylase 11 | Histone deacetylase | TF_cofactors |
| ENSMUSG00000049892 | 7.639 | 6.611 | 6.267 | 9.189 | 13.956 | 9.200 | 5.030 | 6.376 | 7.613 | Rasd1 | RAS, dexamethasone-induced 1 | Others | TF_cofactors |
